# Terminal-Selective sp^3^ C–H Borylation of Carbonyl Derivatives by a Di(pyridyl)arylmethane-Ligated Iridium Catalyst

**DOI:** 10.1021/jacs.6c04921

**Published:** 2026-06-16

**Authors:** Shuyao Zhang, Taylor M. Estock, Nathan D. Schley

**Affiliations:** Department of Chemistry, 5718 Vanderbilt University, Nashville, Tennessee 37235, United States

## Abstract

An iridium catalyst based on a 2,2’-dipyridylarylmethane ligand shows high activity at low catalyst loading for terminal *sp^3^* C–H borylation of aliphatic carbonyl derivatives under conditions where the substrate is the limiting reagent. The site selectivity of the process is not directed by the functional group but, instead, by a preference for borylation of unhindered methyl groups over methylene or α-branched methyl groups. The active catalyst species are formed *in situ* from commercial precatalysts and a hydrosilane activator, with structural evidence provided by an isolable complex bearing a κ^3^-metalated ligand. The resulting alkylboronate esters are versatile reagents and are amenable to Pd-catalyzed *B*-alkyl Suzuki cross-coupling.

## Graphical abstract

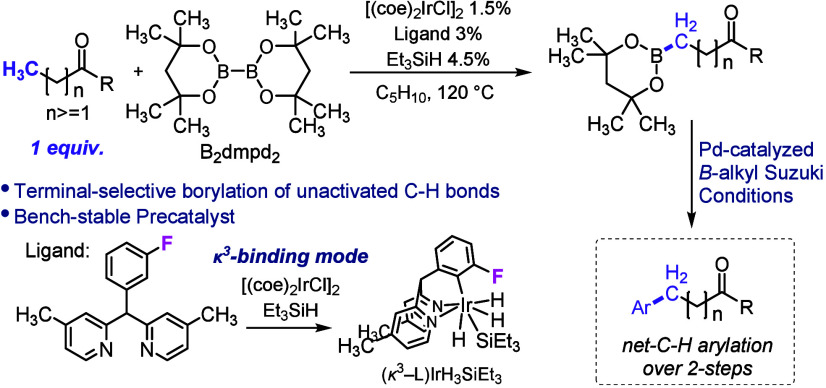

Transition metal catalyzed C–H borylation provides a direct means for the introduction of organoboron functionality into otherwise unactivated hydrocarbon derivatives.^1–5^ The versatility of organoboron compounds has led to their applications in organic synthesis,^6–9^ chemical sensing,^10^ material science^11^ and biochemistry.^12,13^ While *sp* ^2^ C–H borylation has been well developed across many substrate classes,^2,4^ the corresponding *sp* ^3^ C–H borylation reaction is substantially underdeveloped by comparison. In particular, systems for the catalytic C–H borylation of unactivated *sp* ^3^ C–H bonds remain constrained to a few examples with substantial limitations in conditions and scope.^6–14^ For the purposes of this discussion, we define activated *sp* ^3^ C–H bonds as ones where their proximity to a functional group is a driver of C–H borylation reactivity.^15–40^

Previously, our group reported that a dipyridyl­(3-fluorophenyl)­methane ligand enables Ir-catalyzed *sp* ^3^ C–H borylation on aliphatic hydrocarbons with comparatively high turnover numbers and yield (Figure 1A).^8^ In that work, we observed that high rates of alkane borylation under neat conditions where the substrate was also the solvent could be translated to the borylation of small excesses (5 equiv.) of linear alkane derivatives in cyclic hydrocarbon solvent for the first time. Contemporaneously, the Hartwig group identified a phenanthroline derivative which allowed for the borylation of unactivated alkane C–H bonds under conditions where the substrate was the limiting reagent, a major achievement at the time^9^ which has been refined^41^ but not matched by other catalyst systems. While substrate excess may be acceptable for very inexpensive compounds like paraffins, the development of alkane borylation systems which operate with limiting substrate creates opportunities for borylation of molecules of increasing value and complexity.

**Figure 1:**
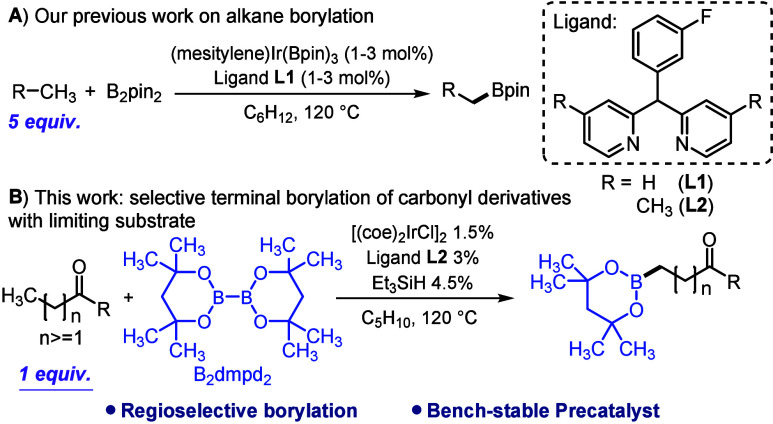
Ir-catalyzed *sp* ^3^ C–H borylation with 2,2’-dipyridylarylmethane ligands.

In this work, we report the development of a catalyst system for the terminal-selective *sp* ^3^ C–H borylation of aliphatic alcohol and carbonyl derivatives that operates effectively under limiting substrate conditions (Figure 1B). This system makes use of a stable and commercially available iridium precatalyst with a simple silane activator and a di­(4-methylpyridyl)­(3-fluorophenyl)­methane ligand (**L2**). The exceedingly challenging transformation described does not benefit from directing effects or substrate activation, instead relying on catalyst-controlled discrimination to functionalize terminal alkyl positions on the limiting substrate in a sea of C–H bonds.

We began by surveying four commercially available diboron reagents using a variation on conditions we reported previously for the C–H borylation of small excesses of substrate.^8^ These initial experiments demonstrated that B_2_dmpd_2_ (dmpd = 2,4-dimethylpentane-2,4-diol) (**2a**) gave considerably higher yields than the benchmark reagent B_2_pin_2_ (pin = pinacol) when a single equivalent of *n*-octane was employed (Figure 2).

**Figure 2:**
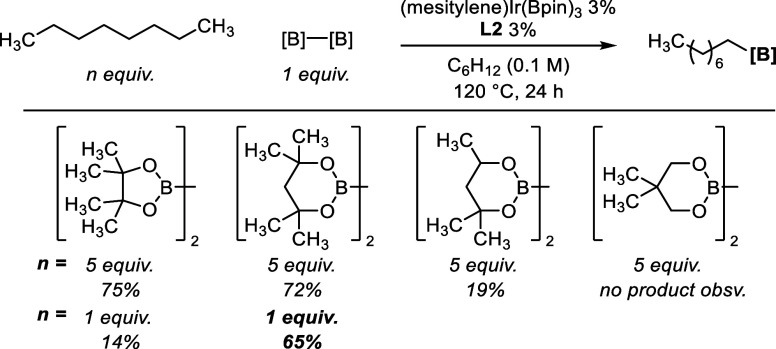
Effect of diboron reagent on alkane C–H borylation. Yields estimated by ^1^H NMR spectroscopy of crude reaction mixtures.

Buoyed by this early success, we examined the C–H borylation of linear esters, which represent considerably more-challenging substrates for aliphatic C–H borylation than alkanes. We expected that esters derived from branched alcohols would drive C–H borylation selectivity to the acid-derived tail, since α-branched alkyl substituents are more challenging to borylate.^12,13^ Tests with several such derivatives failed to give any borylation products (Table 1, entry 1), which was puzzling since the *n*-butanol derived hexanoate ester **1p** (whose CH_3_ groups are equidistant from the carbonyl moiety) underwent C–H borylation in high yield, albeit without selectivity. Substrates like **1t**–**1v** are absent from leading methods^8,9^ for undirected C–H borylation, which speaks to remaining unmet challenges in this field. The isobutyl propionate **1a** did undergo the desired borylation in low yield (12%), which showed that a branched ester could drive C–H borylation toward the *n*-aliphatic tail of the molecule.

**Table 1:**
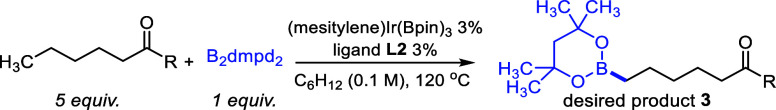

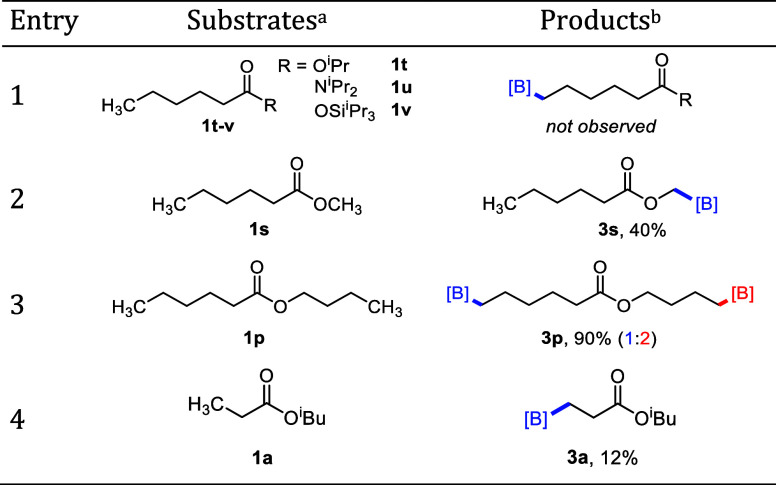
Preliminary Survey of Carbonyl Derivatives

a Conditions: Substrate (5 equiv), **2a** (0.1 mmol), [Ir] (0.003 mmol), **L2** (0.003 mmol), cyclohexane (1 mL), nitrogen, 120 °C.

b Yields determined by crude ^1^H NMR with dimethyl sulfone as internal standard.

A careful search of reaction parameters ultimately identified a set of reaction conditions which allow for the borylation of **1a** to give **3a** in 65% yield under conditions where **1a** is the limiting reagent. Results from alternative conditions highlighting the importance of the reaction variables are summarized in Table 2. Cyclopentane solvent provides a significant advantage over cyclohexane, at the expense of a small increase in competing solvent borylation. Two equivalents of B_2_dmpd_2_ modestly outperforms a single equivalent for substrate **1a**, consistent with the observation that B_2_dmpd_2_ is depleted faster than substrate formation under some conditions (Tables S1–S2). An excess of either substrate or B_2_dmpd_2_ gives higher yields than a stoichiometric ratio (Table S4). The identification of conditions where the substrate can be employed as the limiting reagent is a major advance for this system, since so few others with comparable capability are known for unactivated substrates.^9,41^

**Table 2:**
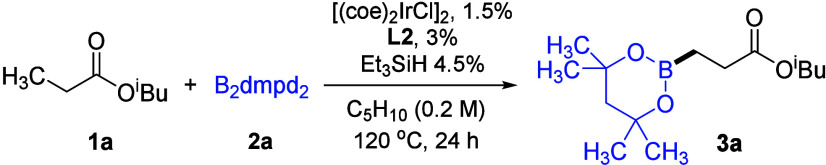
Optimization of Reaction Conditions

Entry	Condition Variation^a^	Yield of **3a** ^b^
1	None	65%
2	Cyclohexane as solvent	47%
3	0.1 M substrate instead of 0.2 M	40%
4	110 °C instead	34%
5	130 °C instead	48%
6	0.2 mmol (1 equiv) B_2_dmpd_2_ instead	48%
7	[(cod)IrOMe]_2_, without Et_3_SiH	53%
8	[(cod)IrOMe]_2_, with Et_3_SiH	10%
9	[(coe)_2_IrCl]_2_ omitted	not obsv.
10	**L2** omitted	not obsv.
11	Et_3_SiH omitted	25%

a Conditions: **1a** (0.2 mmol), **2a** (0.4 mmol), [Ir] (0.003 mmol), **L5** (0.006 mmol), Et_3_SiH (0.009 mmol), cyclopentane (1 mL), nitrogen, 120 °C, 24 h.

b Yields determined by crude ^1^H NMR with dimethyl sulfone as internal standard.

Alongside the selection of reaction conditions, we undertook a study of the iridium precatalyst. In a previous report, we employed (η^*6*^-mesitylene)­Ir­(Bpin)_3_ as the precatalyst.^8^ This iridium reagent has been known to give highly active C–H borylation systems for both *sp* ^*3*^ and *sp* ^*2*^ C–H borylation, but it is accessible only in poor yield.^42^ Since it already bears Bpin-derived boryl ligands, its application alongside B_2_dmpd_2_ leads to mixtures of boronate products, which serves as a further downside to its use. Fortunately, we found that the commercially available precatalyst [(coe)_2_IrCl]_2_ gives a very active catalyst system when combined with triethylsilane. Indeed, our final optimized conditions require only 3% loading of this commercial precatalyst on an iridium basis, comparing favorably to other systems.^9,41^ Low loading is important given the limited terrestrial abundance of iridium, though recent advances in lab-scale iridium recycling offer additional means to offset the use of iridium.^43^

During the exploration of the reaction substrate scope, we found that ca. 40–60% isolated yields of the alkylboronate could be obtained for most substrates we examined. Our yields are in line with those reported in the few examples of alkane C–H borylation using limiting substrate.^9,41^ Alkylboronates can be challenging to purify, which is occasionally noted in the literature,^9^ but can also be inferred from the frequency with which methods for C–H borylation rely on in situ transformations prior to purification for at least a portion of the substrate scope (e.g., to BF_3_K salts or alcohols).^8,9,26^ ^,^ ^41,44^ Given their versatility, we chose to focus on isolation of the pure boronate esters. The relevance of the NMR yield assay is demonstrated in the synthesis of **6b** below (Figure 5A), which can be obtained in higher yield than its parent boronate ester **5d** (Figure 4).

These catalyst conditions are applicable to the selective, terminal C–H borylation of an array of esters and ketones. (Figure 3). Isobutyl esters derived of C_5_ or shorter acids underwent C–H borylation in satisfactory yields (**3a**–**3c**), while longer chain esters were more challenging. The corresponding cyclohexyl esters perform similarly under optimized conditions, giving the products in somewhat lower yields (**3d**–**3f**). Longer chain isobutyl esters undergo competitive ^i^Bu C–H borylation (Figure S4). The pivalate ester **1g** undergoes borylation on the alcohol-derived chain, giving **3g** in good yield. Ketone substrates were also found to be suitable, giving the monoborylated products in moderate yields in the case of **3h** and **3i**, though the site selectivity of **3i** is modest. The higher dialkylketones **1j** and **1k** are found to undergo competitive hydroboration, which leads to somewhat reduced yields of **3j** and **3k**.

**Figure 3:**
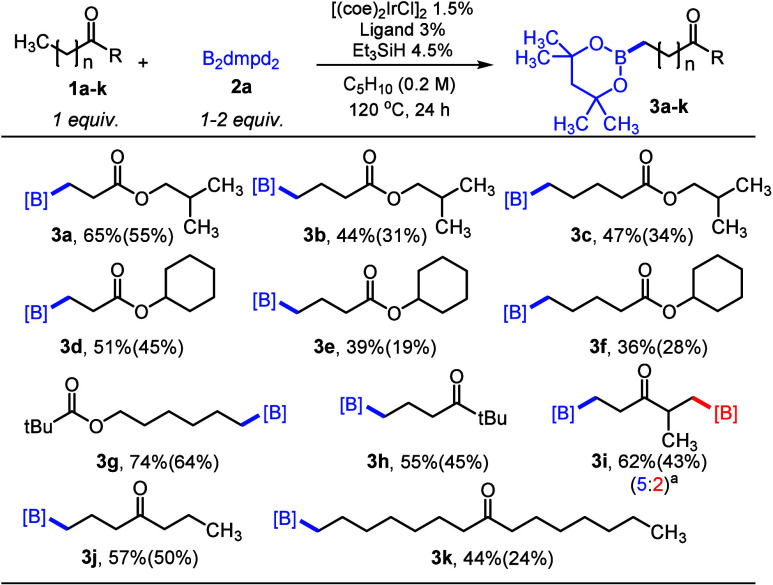
Substrate scope of carbonyl derivatives. NMR yields/(Isolated yields). ^a^Isolated yield of the isomeric mixture.

This catalyst system is suitable for other aliphatic substrates as well (Figure 4). Triisopropylsilyl- (TIPS) protected alcohols up to C_12_ in length undergo C–H borylation in good yields (**5a**–**5d**). A limitation on substrate length is also observed but is more relaxed than the ester case described above. Alkyl ethers and polyethylene glycol (PEG) derivatives are also good substrates (**5e**–**5h**), with substrates **4f** and **4g** each giving differentially end-functionalized products **5f** and **5g** representing unusual linker precursors. Long chain paraffins are tolerated at least up to eicosane (C_20_) (see Figure S4). 1-Fluorododecane gives a slightly reduced yield (**5k**) relative to the parent hydrocarbon (**5j**). In line with previous systems for the C–H borylation of alkanes, aromatic substrates undergo preferential *sp* ^*2*^ C–H borylation, but steric hindrance can divert C–H borylation to terminal aliphatic C–H bonds (**5i**). A selection of other substrates explored can be found in the Supporting Information.

**Figure 4:**
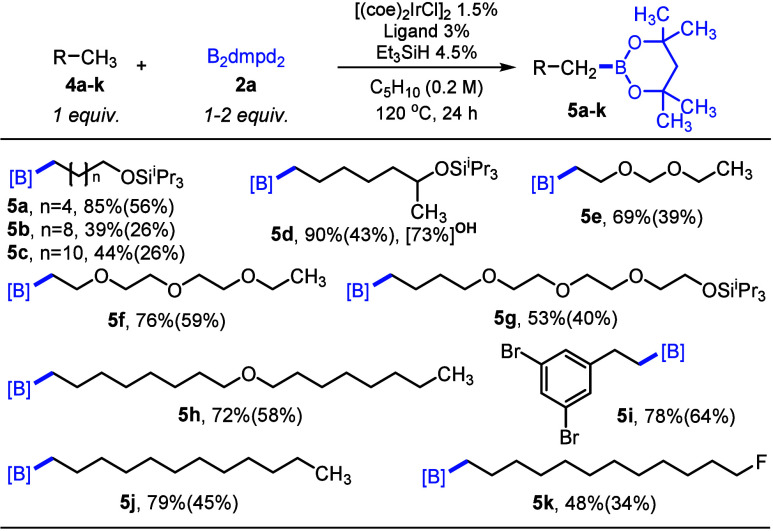
Substrate scope of other aliphatic derivatives. NMR yields/(Isolated yields)/^OH^isolated yield of the alcohol obtained by oxidation of the crude boronate ester.

To demonstrate the utility of the C–H borylation products, we investigated their reactivity in several reactions of alkylboronate esters (Figure 5). They are easily oxidized to the corresponding alcohol, for instance **3a** gives alcohol **6a** in excellent yield (Figure 5A). This oxidation also works well on crude mixtures - a C–H borylation/oxidation sequence involving the intermediacy of **5d** gives the mono-TIPS protected 1,6-heptadiol **6b** in 73% isolated yield. This two-step sequence gives higher yield than was obtained for the first step if **5d** is purified (43%). Boronate esters which do not contain alkyl ester functionality can also be converted to alkyl bromides by the procedure in Figure 5B.^45^

**Figure 5:**
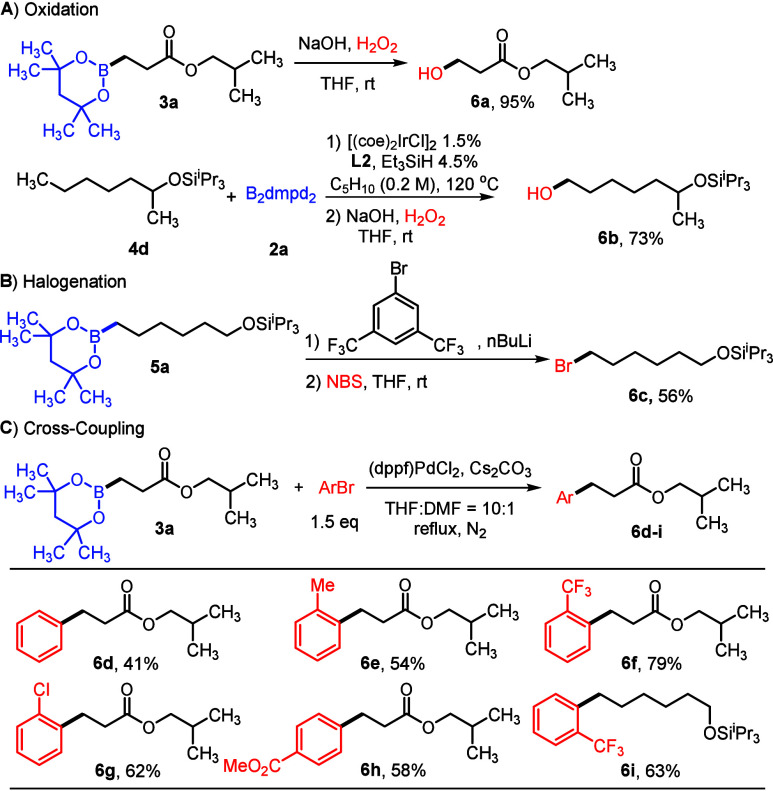
Applications of alkylboronate esters in oxidation, halogenation, and cross-coupling methods. Isolated yields.

Among the most appealing applications of C–H borylation-derived alkylboronate esters is their use as nucleophilic coupling partners in *B*-alkyl Suzuki cross-coupling reactions. We have found that the alkyl-Bdmpd products obtained here are amenable to Pd-catalyzed *B*-alkyl Suzuki cross-coupling with bromoarenes. The resulting products represent a net-terminal arylation of the parent hydrocarbon (Figure 5C).

The triethylsilane activator is an interesting feature of this system. Brookhart has shown that treatment of [(coe)_2_IrCl]_2_ with triethylsilane gives a soluble product with a single hydride resonance in the NMR spectrum at −21 ppm which they posited was the chloro-bridged dimer [(Et_3_Si)_2_IrH_2_(μ-Cl)]_2_.^46^ Our experiments confirm this formulation, with new data that suggests the heavy-atom ligands adopt a distorted tetrahedral arrangement (Figure 6, see the Supporting Information). Treatment of this complex with **L2** gives an isolable complex (**7**) wherein the ligand adopts a κ^*3*^ binding mode resulting from selective metalation *ortho* to the fluorine substituent. The resulting complex is an iridium trihydridosilyl whose limiting formal oxidation states are Ir­(III) [**L2**IrH_2_(σ-HSiEt)] or Ir­(V) [**L2**IrH_3_SiEt_3_] depending on the degree of oxidative addition of the H–SiEt_3_ bond.^47–49^ We hypothesized that dipyridylarylmethane ligands may bind κ^*3*^ via cyclometalation of the 3-fluoroarene in a previous report.^8,50–52^ The preference for C–H metalation of **L2** *ortho* to fluorine is computed to be consistent with the thermodynamic product, as the observed isomer is calculated to be 3.3 kcal/mol below the *para* isomer (Figure S109). Thermodynamic preferences for *o*-F metalation can be large and consequential for reductive elimination rates.^53,54^ This preference may stem from differential destabilization of the C–H and C–M bonds by the fluorine substituent.^55–57^ The observed binding *ortho* to fluorine may explain the requirement for the 3-fluoroaryl substituent in our previously reported system,^8,50^ though a more detailed mechanistic study on a related system is forthcoming. Regardless, the isolation of complex **7** provides a plausible role for Et_3_SiH as aiding in ligand metalation and thus precatalyst activation in this system.

**Figure 6:**
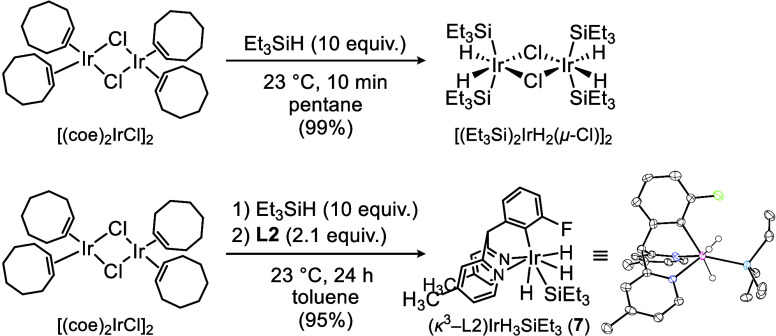
Precatalyst activation studies. ORTEP of **7**.

In summary, we report a terminal-selective *sp* ^*3*^ C–H borylation of carbonyl derivatives through the combination of an iridium silyl precursor and a di­(pyridyl)­arylmethane ligand. Isobutyl esters are found to be protecting groups for carboxylic acid substrates of moderate chain length, driving selective terminal borylation. The resulting catalyst system operates at very low catalyst loading and represents one of very few alkane C–H borylation catalysts capable of operating on unactivated C–H bonds with the substrate as the limiting reagent. The application of the derivatives thus obtained in a *B*-alkyl Suzuki cross-coupling provides additional practical synthetic value for this transformation.

## Supplementary Material





## Notes

Text file of all computed molecule Cartesian coordinates in a format for convenient visualization (XYZ)
Experimental procedures, summaries of computational and experimental results, compound characterization data, and X-ray crystallographic data (PDF)
