# Supplementary material for: Terminal-Selective sp3 C–H Borylation of Carbonyl Derivatives by a Di(pyridyl)arylmethane-Ligated Iridium Catalyst
Source: J Am Chem Soc. 2026 Jun 16;148(25):25336–41. doi: 10.1021/jacs.6c04921 (PMC13339159; doi:10.1021/jacs.6c04921)
Supplement: Supplementary file 2 [file ja6c04921_si_002.pdf]

Supporting Information

Terminal-Selective sp³ C–H Borylation of Carbonyl Derivatives by a Di(pyridyl)arylmethane-Ligated Iridium Catalyst

Shuyao Zhang, Taylor M. Estock, and Nathan D. Schley*

Department of Chemistry, Vanderbilt University, Nashville, Tennessee 37235 United States

**I.**	**General Information**	**1**
**II.**	**Synthesis and Characterization**	**2**
**III.**	**General Procedures**	**7**
**IV.**	**Condition Optimization and Substrate Scope**	**9**
**V.**	**Compound Characterization**	**12**
**VI.**	**NMR Spectral Data**	**30**
**VII.**	**X-ray Crystallographic Data**	**133**
**VIII.**	**Computational Methods**	**136**
**IX.**	**References**	**138**

I. General Information

General Considerations. All manipulations were carried out using standard vacuum, Schlenk, cannula, or glovebox techniques under N_2 unless otherwise specified. Tetrahydrofuran, dichloromethane, pentane, toluene and diethyl ether were degassed with argon and dried over activated alumina using a solvent purification system. All other reagents are broadly available and were used as received. Unless otherwise stated, the culture tubes used for the procedures below are 8 mL capacity, 100 mm long, and accept a 13-415 threaded cap (for instance, Fisher catalog number 14-957-76A).

Spectroscopy. ^{1}H, $^{13}C\{^{1}H\}$, ^{19}F, and ^{29}Si NMR spectra were recorded on Bruker NMR spectrometers at ambient temperature unless otherwise noted. ^{1}H, and $^{13}C\{^{1}H\}$ and chemical shifts are referenced to residual solvent signals. ^{19}F and ^{29}Si NMR were externally referenced against benzene solutions of fluorobenzene (-113.11 ppm)[1] and tetramethylsilane (0 ppm) respectively.[2]

Mass Spectrometry. High resolution mass spectrometry was conducted at the Mass Spectrometry Research Center (MSRC) at Vanderbilt University. Solutions of purified products were diluted into a solvent and analyzed by ESI using an Orbitrap mass analyzer. M+H or M+Na ions were not detected for most alkyl boronate products that lacked Lewis-basic functionality (e.g. hydrocarbon derived or $^{i}Pr_3Si$-ether derivatives).

For these cases, data from low resolution GC-MS (EI, Agilent 7970A/5973) are included instead. In most cases Bdmpd and Bpin esters fragment to give highly abundant M-CH_3 ion signals, but the molecular ion is reported when observed.

Elemental Analysis. Elemental analysis of **(κ^3–L2)IrH_3(SiEt_3)** is of the bulk sample for which the yield is reported. No additional purification operations are carried out prior to packaging for analysis, but samples are dried under vacuum to remove residual or co-crystalized solvent. Elemental analyses were performed at the University of Rochester CENTC Elemental Analysis Facility. That facility acknowledges support from the National Science Foundation under NSF CHE-0650456.

II. Synthesis and Characterization

Organometallic starting materials. (η^6-mesitylene)(tris-pinacolboryl)iridium [(η^6-mesitylene)Ir(Bpin)$_3$ was freshly prepared according to a published procedure][3] and stored at -35 °C. Chlorobis(cyclooctene)iridium(I) dimer [(coe)$_2$IrCl]$_2$, Bis(1,5-cyclooctadiene)diiridium(I) dimethoxide [(cod)IrOMe]$_2$, and Pd(dppf)Cl_2 were purchased from chemical vendors and used as received.

Ligand synthesis. 2,2'-di(4-methylpyridyl)-3-fluorophenylmethane (**L2**) was synthesized from 2-(3-fluorobenzyl)-4-picoline[4] according to a reported variation[5] of a method for the preparation of triarylmethanes.[6]

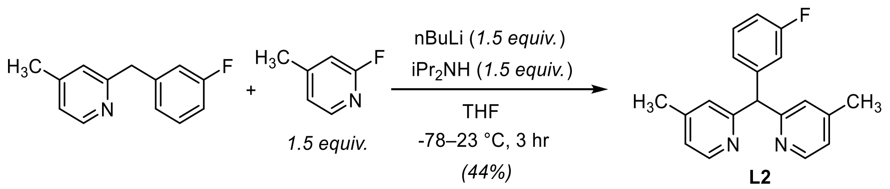

A 50 mL Schlenk flask was charged with ^{i}Pr$_2$NH (0.266 mL, 1.87 mmol, 1.5 equiv.) in 20 mL THF under an atmosphere of nitrogen and was cooled to -78 °C. nBuLi (0.857 mL, 1.87 mmol, 1.5 equiv., 2.5 M in hexanes) was added dropwise over 15 min to give a lithium diisopropylamide solution. The resulting solution was allowed to warm to 0 °C while stirring for 30 min. 2-(3-fluorobenzyl)-4-picoline (0.25 g, 1.25 mmol, 1 equiv.) and 2.5 mL THF were added to a separate Schlenk flask under nitrogen and cooled to 0 °C. The LDA solution was transferred dropwise via cannula to the fluorobenzyl picoline solution, and the resulting mixture allowed to warm to ambient temperature with stirring for 45 min. 2-fluoro-4-methylpyridine (0.161 mL, 1.87 mmol, 1.5 equiv.) was then added dropwise via syringe, and the reaction allowed to stir at room temperature for 3 hours at room temperature. The mixture was quenched by slow addition of deionized water and the organic layer extracted with dichloromethane. The aqueous phase was washed with

three portions of dichloromethane and the combined organic layers were washed with brine and dried over Na_2SO_4 followed by concentration in vacuo. The resulting mixture was purified by column chromatography to give the product as a colorless solid. Yield: 0.160 g (44%)

^{1}H NMR ($CDCl_3$, 400 MHz): δ 8.44 (d, J = 5.0 Hz, 2H), 7.27 (td, J = 7.9, 6.3 Hz, 1H), 7.09 (s, 2H), 7.06 (d, J = 7.9 Hz, 1H), 7.00 (dt, J = 1.7, 9.6 Hz, 1H), 6.98 (d, J = 5.1 Hz, 2H), 6.90 (td, J = 2.5, 8.5 Hz, 1H), 5.75 (s, 1H), 2.31 (s, 6H).

^{13}C NMR (151 MHz, $CDCl_3$) δ 163.03 (d, *J* = 245.2 Hz), 161.48, 149.42, 147.92, 144.46 (d, *J* = 7.3 Hz), 129.95 (d, *J* = 8.3 Hz), 125.21 (d, *J* = 2.8 Hz), 124.98, 122.94, 116.47 (d, *J* = 21.8 Hz), 113.74 (d, *J* = 21.1 Hz), 61.19, 21.30.

^{19}F NMR ($CDCl_3$, 376 MHz): δ -113.1.

GCMS m/z (EI, 70 eV): 292.1 $(C_{19}H_{17}N_2F)^+$ $[M]^+$

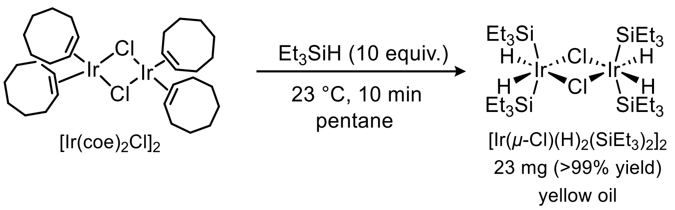

[Ir(*μ*-Cl)(H)$_2$(SiEt$_3$)$_2$]$_2$ This compound was prepared according to a modified literature procedure.[5] In an inert-atmosphere glove box, an oven-dried glass vial equipped with a magnetic stirbar was charged with $[(coe)_2IrCl]_2$ (22.4 mg, 25 μmol, 1 equiv.) and dry pentane (1.00 ml, 25.0 mmolar) to give a heterogeneous orange solution. Et_3SiH (40 μL, 250 μmol, 10 equiv.) was added and the mixture quickly became yellow and homogeneous. After stirring for 10 minutes at room temperature, the solution was placed under vacuum for 1h to afford the pure product as a temperature sensitive yellow oil. Yield: 23 mg (>99%) X-ray quality crystals were grown from a concentrated solution of *n*-pentane at -35 °C. The NMR spectra agree with reported data.[7] We have reassigned this compound as the geometry shown above based on a low quality small-molecule X-ray diffraction study. Briefly, the low-melting compound was crystallized at -35 °C and crystals were handled cold. The crystals underwent a destructive phase change at 223 K, so X-ray analysis was conducted on crystals mounted and held at 253 K. Extensive disorder and poor high-angle diffraction limited the quality of the dataset, but the compound crystalizes in Pbca with unit cell dimensions of 13.7118, 12.9884, 21.642, 90°, 90°, 90°. The Si-Ir-Si angle is *ca.* 103-117° and the geometry of the $IrCl_2Si_2$ fragment is approximately tetrahedral. The atomic coordinates determined in this manner were used as a starting point for a DFT computation of the structure, see Figure S110.

^{1}H NMR (600 MHz, C_6D_6): δ 1.15-1.00 (m, 60H, SiCH_2CH_3), -21.1 (s, 4H, Ir-*H*)

^{13}C NMR (150 MHz, C_6D_6): δ 12.05, 8.78.

^{29}Si NMR (99 MHz, C_6D_6): δ 45.9.

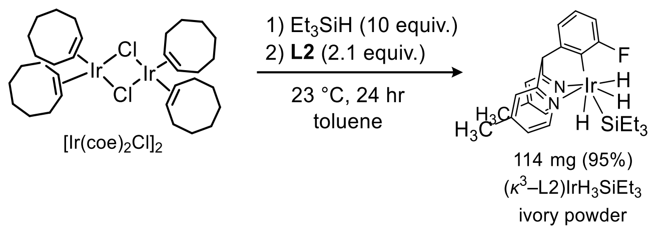

(κ^3–L2)$IrH_3(SiEt_3)$ (7) In an inert-atmosphere glove box, an oven-dried glass vial equipped with a magnetic stirbar was charged with $[(coe)_2IrCl]_2$ (89.6 mg, 100 μmol, 1 equiv.) and dry toluene (2.00 ml, 50.0 mmolar) to give a heterogeneous orange solution. Et_3SiH (160 μL, 1.00 mmol, 10 equiv.) was added and the mixture quickly became yellow and homogeneous. After stirring for 5 minutes at room temperature, Ligand **L2** (61.4 mg, 210 μmol, 2.1 equiv.) was added at once and the reaction was left to stir for 24h at room temperature. The solution gradually turns opaque and light solid begins precipitating. The next day, the volatiles were removed under vacuum. The residual off-white powder was washed with ten 0.5 mL portions of cold pentane to remove an orange impurity, then dried at reduced pressure. Yield: 0.114 g (95%) X-ray quality crystals were grown from a concentrated solution of diethyl ether at -35 °C. The solid can be stored in air for months without apparent degradation, but the material is air sensitive in solution. Elemental Analysis for $C_{25}H_{34}FIrN_2Si$: C, 49.89; H, 5.69; N, 4.65. Found C, 49.674; H, 5.443; N, 4.623.

^{1}H NMR (600 MHz, C_6D_6) δ 8.57 (d, *J* = 5.6 Hz, 2H, pyridyl C-H), 7.30 (dd, *J* = 6.6, 1.5 Hz, 1H, phenyl C-H), 7.00-6.93 (m, 2H, phenyl C-H), 6.73 (s, 2H, pyridyl C-H), 5.73 (dd, *J* = 5.5, 2.5 Hz, 2H, pyridyl C-H), 5.22 (s, 1H, py$_2$Ar*CH*), 1.69 (q, *J* = 7.8 Hz, 6H, Si*CH$_2$*CH$_3$), 1.57 (s, 6H, py-C*H$_3$*), 1.55 (t, J = 7.7 Hz, 9H, SiCH$_2$*CH$_3$*), -5.39 (s, 1H, Ir-*H*), -18.3 (s, 2H, Ir-*H*)

^{13}C NMR (151 MHz, C_6D_6) δ 169.67 (d, *J* = 235.5 Hz), 156.93, 155.86, 148.95, 141.92 (d, *J* = 53.6 Hz), 139.06 (d, *J* = 17.7 Hz), 124.49, 123.94 (d, *J* = 7.2 Hz), 122.98, 121.54 (d, *J* = 2.8 Hz), 111.63 (d, *J* = 31.5 Hz), 70.05, 19.93, 16.72, 9.08.

^{19}F NMR (471 MHz, C_6D_6) δ -82.7 (s)

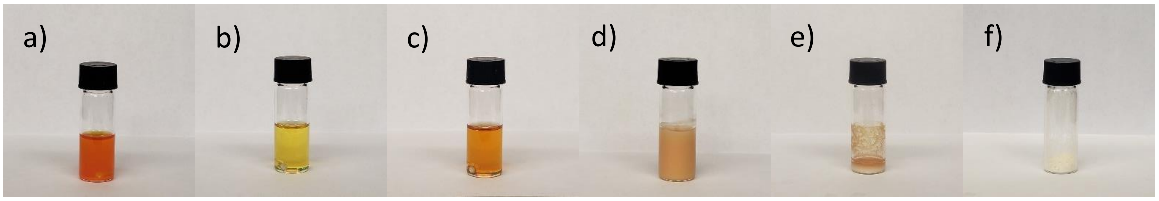

Figure S1. Synthesis of (κ^3–L2)$IrH_3(SiEt_3)$. a) Initial heterogeneous solution of $[(coe)_2IrCl]_2$ in toluene, b) 1 minute after silane addition c) 1 minute after ligand addition d) 24 hr after ligand addition e) Crude product after concentration under vacuum f) Pure product after workup.

Substrate synthesis. All ester substrates and TIPS-protected alcohol substrates were synthesized and characterized via the synthetic procedure listed below. Other reagents and building blocks were all purchased from chemical vendors and used as received.

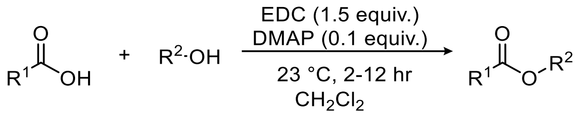

Preparation of ester substrates (1a-g): To an oven-dried Erlenmeyer flask was added carboxylic acid (10 mmol, 1 eq.), alcohol (10 mmol, 1 eq.), DMAP (1 mmol, 0.1 eq.) and dichloromethane (50 mL). EDC (2.88 g, 15 mmol, 1.5 eq.) was then added during stirring. The reaction was stirred at room temperature for 2-12 hr. After the reaction finished (determined by TLC), the reaction was quenched with brine, extracted with dichloromethane, dried over Na_2SO_4, concentrated under vacuum. The crude product was purified by column chromatography on silica gel to obtain the desired product **1**.

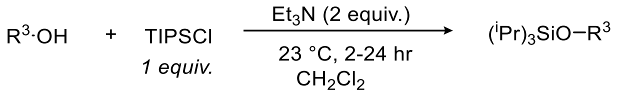

Preparation of TIPS protected alcohol (4a-d): To an oven-dried Erlenmeyer flask was added alcohol (10 mmol, 1 eq.), Et_3N (2.8 mL, 20 mmol, 2 eq.) and dichloromethane (50 mL). TIPSCl (1.93 g, 10 mmol, 1 eq.) was then added during stirring. The reaction was stirred at room temperature for 2-24 h. After the reaction finished (determined by TLC), the reaction was quenched with brine, extracted with dichloromethane, dried over Na_2SO_4, concentrated under vacuum and purified by column chromatography on silica gel to obtain the desired product **4**.

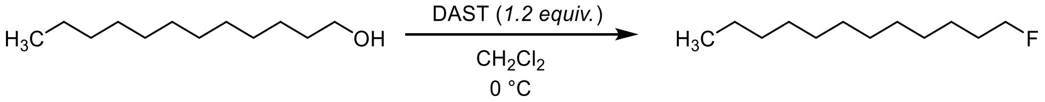

Preparation of 1-Fluorododecane (4k): To an oven-dried round-bottom flask was added 1-dodecanol (10 mmol, 1 eq.) and methylene chloride (50 mL). DAST (12 mmol, 1.2 eq.) was added dropwise at 0 °C under N_2 atmosphere. The reaction was stirred at room temperature for 1 h. After the reaction finished (confirmed by TLC), the reaction was quenched with saturated $NaHCO_3$ solution and extracted with methylene chloride. The combined organic layer was successively washed with water and brine, dried over Na_2SO_4, concentrated under vacuum and purified by column chromatography on silica gel to obtain the desired product **4k**.

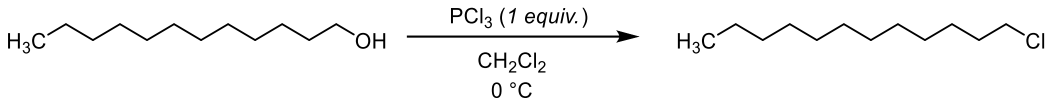

Preparation of 1-Chlorododecane (4q): To an oven-dried round-bottom flask was added 1-dodecanol (10 mmol, 1 eq.) and methylene chloride (50 mL). PCl_3 (10 mmol, 1 eq.) was added dropwise at 0 °C under N_2 atmosphere. The reaction was stirred at room temperature for 12 h. After the reaction finished (confirmed by TLC), the reaction was quenched with saturated $NaHCO_3$ solution and extracted with methylene chloride. The combined organic layer was successively washed with water and brine, dried over Na_2SO_4, concentrated under vacuum and purified by column chromatography on silica gel to obtain the desired product **4q**.

III. General Procedures

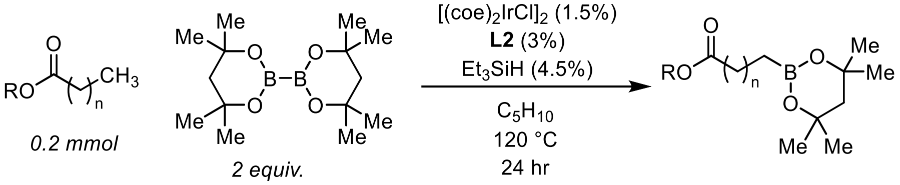

Procedure for the borylation of ester substrates (Figure 3). In an inert atmosphere glovebox, to a 8 mL screw cap glass culture tube with a stir bar was added $[(coe)_2IrCl]_2$ (2.7 mg, 0.003 mmol, 0.015 eq.), **L2** (1.8 mg, 0.006 mmol, 0.03 eq.), Et_3SiH (1.5 uL, 0.009 mmol, 0.045 eq.), B_2dmpd_2 (112.8 mg, 0.4 mmol, 2 eq.), ester substrate **1a-g** (0.2 mmol, 1 eq.) and cyclopentane (1 mL). The tube was capped with a PTFE-lined cap, removed from the glovebox and was heated in a 120 °C oil bath with stirring for 24 h. The crude mixture was then cooled to room temperature, concentrated in vacuo, and purified by column chromatography on silica gel (see below) to obtain the desired products **3a-g**.

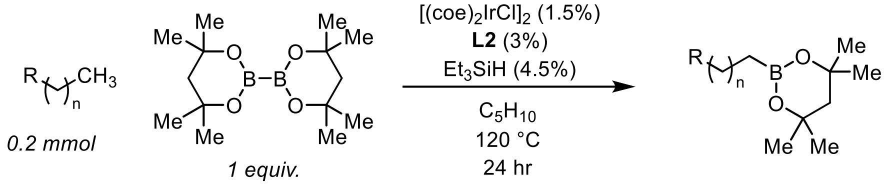

Procedure for the borylation of other substrates (Figures 3 & 4). In an inert atmosphere glovebox, to a 10 mL screw cap glass culture tube with a stir bar was added $[(coe)_2IrCl]_2$ (2.7 mg, 0.003 mmol, 0.015 eq.), **L2** (1.8 mg, 0.006 mmol, 0.03 eq.), Et_3SiH (1.5 uL, 0.009 mmol, 0.045 eq.), B_2dmpd_2 (56.4 mg, 0.2 mmol, 1 eq.), substrate **1h-k** or **4e-k** (0.2 mmol, 1 eq.) and cyclopentane (1 mL). The tube was capped well with a PTFE-lined cap, removed from the glovebox and was heated in a 120 °C oil bath with stirring for 24 h. The crude mixture was then cooled to room temperature, concentrated in vacuo, and purified by column chromatography on silica gel (see below) to obtain the desired product **3h-k** or **5e-k**.

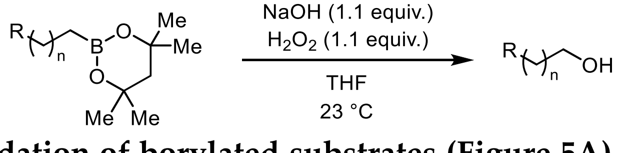

Procedure for the oxidation of borylated substrates (Figure 5A). To a 4 mL vial with a stir bar was added borylated substrate **3** or **5** (0.1 mmol, 1 eq.), NaOH (4.4 mg, 0.11 mmol, 1.1 eq.) and THF (1 mL). 30% H_2O_2 (11.1 uL, 0.11 mmol, 1.1 eq.) was then added while stirring at room temperature. After full consumption (determined by TLC), the reaction was quenched with saturated sodium thiosulfate solution, extracted with EtOAc, dried over Na_2SO_4, concentrated in vacuo and purified by column chromatography on silica gel to obtain the desired product **6a-b**.

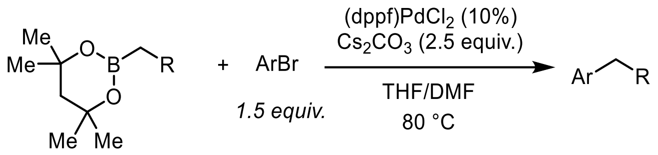

Procedure for B-alkyl Suzuki cross-coupling reactions of Bdmpd boronate esters. (Figure 5C). In an inert atmosphere glovebox, to an 8 mL screw cap glass culture tube with a stir bar was added (dppf)$PdCl_2$ (7.3 mg, 0.01 mmol, 0.1 eq), Cs_2CO_3 (81.5 mg, 0.25 mmol, 2.5 eq.), the bromoarene coupling partner (0.15 mmol, 1.5 eq.), THF (1 mL) and DMF (0.1 mL). **3a** or **5a** (0.1 mmol, 1 eq.) was then added after stirring. The tube was capped well with a PTFE-lined cap, removed from the glovebox and was heated in a 75 °C oil bath with stirring for 12-24 h. The crude mixture was then cooled to room temperature, concentrated under vacuum, and purified by column chromatography on silica gel to obtain the desired product **6d-i**.

Procedure for the preparative TLC separation necessary for nonpolar substrates.
In certain cases, particularly involving nonpolar substrates, there are impurities that are difficult to separate by column chromatography. In these cases, a second purification step involving preparative-TLC was required to obtain high purity. Below is a general procedure for the preparative TLC purification of products that do not absorb UV light.

1. Concentrate, load, and elute the mixed fractions obtained by column chromatography on glass-backed silica gel plates (250 μm).
2. Once eluted, dry the plate with N_2 flow, then estimate the position of the substrate band by placing a light source behind the plate and looking for the change in refractive index (see example below, left).

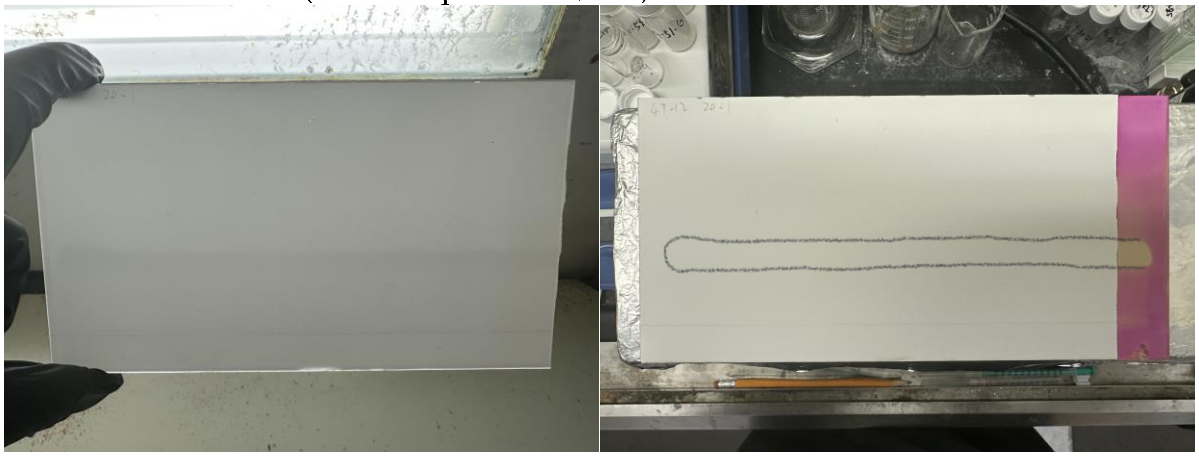

3. Cut a slice from one side of the plate and stain with $KMnO_4$ solution to confirm the probable product band location on the plate.
4. Scrape off the silica and extract the product.

IV. Condition Optimization and Substrate Scope

Preliminary optimization studies

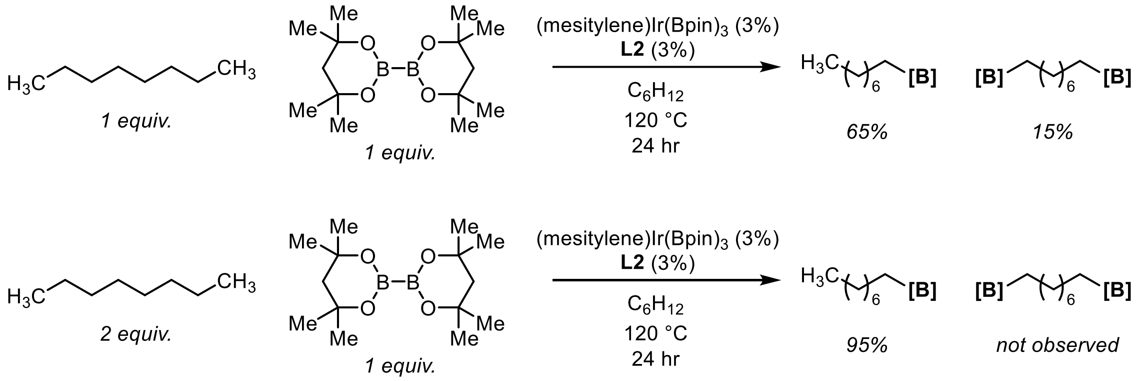

Figure S2. Effect of B_2dmpd_2/*n*-octane ratio on n-octane C-H borylation selectivity.

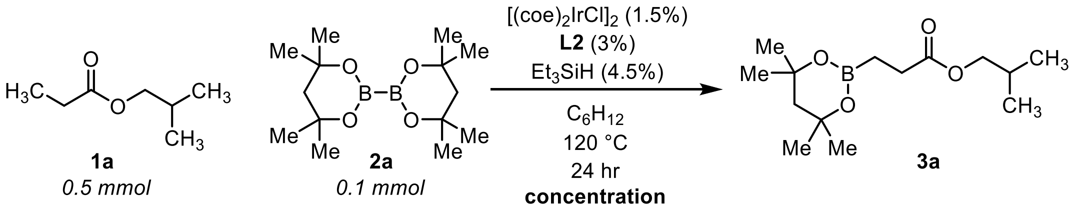

Entry	Condition Variation	Yield of **3a**	Note
1	As above	8%	-
2	0.05 M concentration	< 5%	-
3	0.2 M concentration	11%	-
4	0.2 mmol B_2dmpd_2	< 5%	-
5	0.2 mmol **1a**	10%	-
6	0.1 mmol **1a**	17%	B_2dmpd_2 consumed
**7**	**0.1 mmol 1a, 0.2 M concentration**	**20%**	**B_2dmpd_2 consumed**

Table S1. Optimization studies on carbonyl derivatives - Effect of concentration

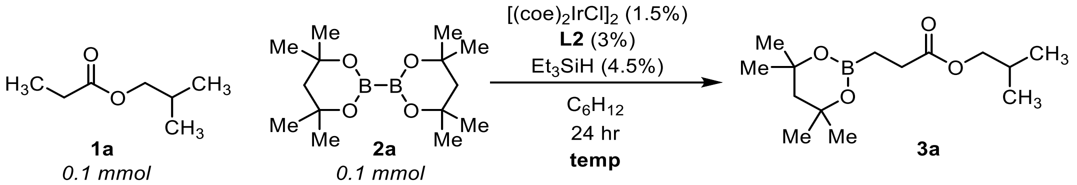

Entry	Condition Variation	Yield of **3a**	Note
1	120 °C	20%	B_2dmpd_2 consumed
2	130 °C	< 5%	B_2dmpd_2 consumed
**3**	**110 °C**	**25%**	**B_2dmpd_2 consumed**
4	100 °C	22%	B_2dmpd_2 consumed
5	80 °C	20%	-

Table S2. Effect of reaction temperature

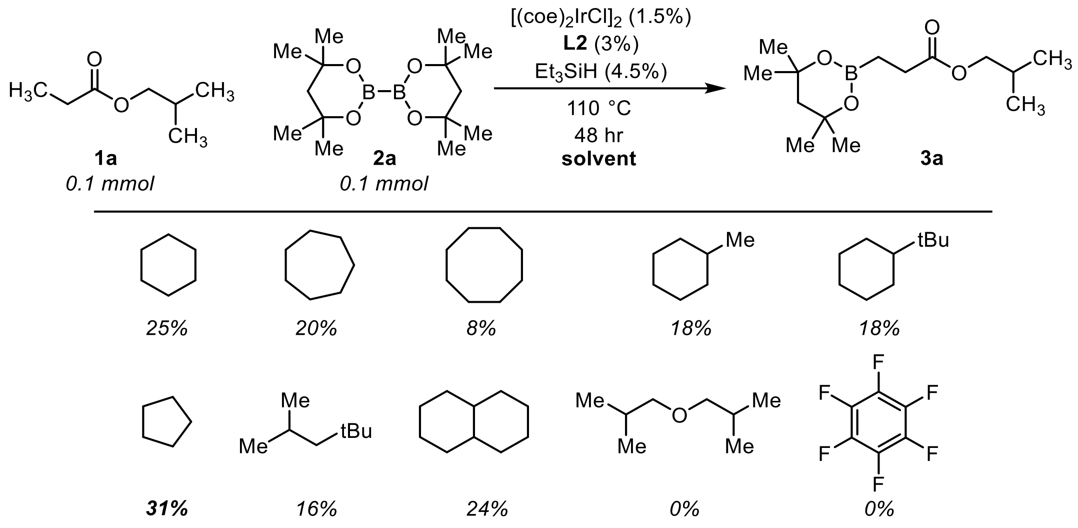

Figure S3. Comparison of reaction solvent in ester borylation.

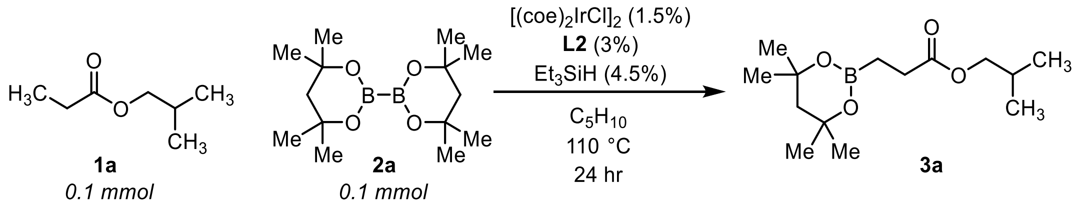

Entry	Condition Variation	Yield of **3a**	Note
1	As above (0.2 M)	31%	B_2dmpd_2 consumed
2	100 °C	26%	-
3	80 °C	<5%	-
**4**	**120 °C**	**50%**	**B_2dmpd_2 consumed**
5	120 °C, 0.33 M	45%	B_2dmpd_2 consumed

Table S3. Refinement after switching to cyclopentane solvent

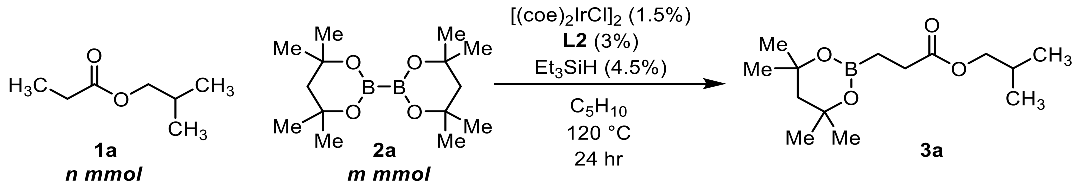

Entry	Substrate (**1a**)	B_2dmpd_2 (**2a**)	**1a** : **2a**	Yield of **3a**
1	0.2 mmol	0.6 mmol	1 : 3	62%
2	0.2 mmol	0.4 mmol	1 : 2	60%
3	0.2 mmol	0.2 mmol	1 : 1	44%
4	0.4 mmol	0.2 mmol	2 : 1	52%
5	0.6 mmol	0.2 mmol	3 : 1	58%

Table S4. Effect of changing the substrate/B_2dmpd_2 ratio near optimized conditions. Yields of **3a** are determined by GC-FID relative to a calibrated internal standard.

Additional substrates explored[a]

The additional substrates below were found to be amenable to alkane C-H borylation under optimized conditions, but isolated yields were not obtained. They are included here for reference.

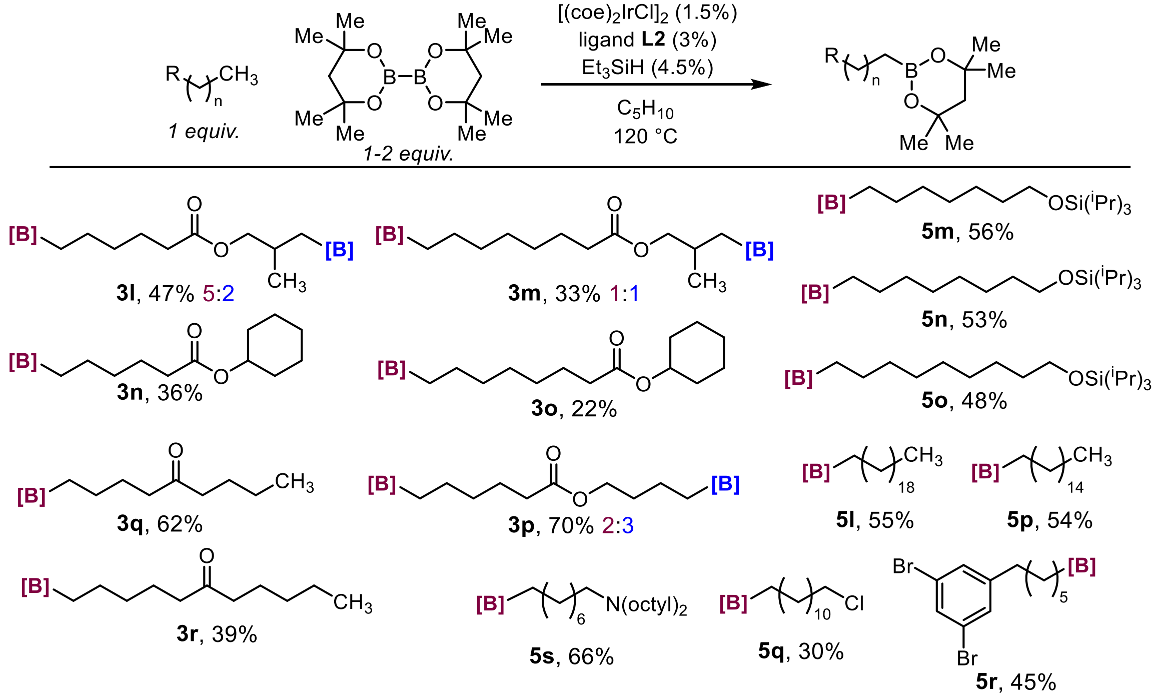

Figure S4. Additional substrates explored which underwent C-H borylation, but for which optimized, isolated yields were not obtained. [a]Yields and selectivity determined by NMR – products isolated in analytical quantities only.

V. Compound Characterization

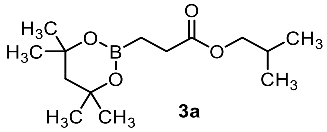

Isobutyl 3-(4,4,6,6-tetramethyl-1,3,2-dioxaborinan-2-yl)propionate (3a)

3a was prepared according to the general procedure for the borylation of ester substrates. The product was isolated as a colorless oil. Yield: 55%.

^{1}H NMR (600 MHz, $CDCl_3$) δ 3.82 (d, *J* = 6.7 Hz, 2H), 2.38 (t, *J* = 7.6 Hz, 2H), 1.91 (dp, *J* = 13.4, 6.7 Hz, 1H), 1.79 (s, 2H), 1.30 (s, 12H), 0.97 – 0.86 (m, 8H).

^{13}C NMR (151 MHz, $CDCl_3$) δ 175.41, 70.38, 70.22, 48.77, 31.71, 29.11, 27.76, 19.16, 10.33 (br).

HRMS m/z (ESI) calcd. for $C_{14}H_{28}BO_4^+$ $(M+H)^+$ 271.2075, found 271.2075.

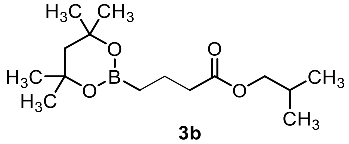

Isobutyl 4-(4,4,6,6-tetramethyl-1,3,2-dioxaborinan-2-yl)butanoate (3b)

3b was prepared according to the general procedure for the borylation of ester substrates. The product was isolated as a colorless oil. Yield: 31%.

^{1}H NMR (600 MHz, $CDCl_3$) δ 3.84 (d, *J* = 6.6 Hz, 2H), 2.30 (t, *J* = 7.7 Hz, 2H), 1.91 (dp, *J* = 13.4, 6.7 Hz, 1H), 1.79 (s, 2H), 1.70 (p, *J* = 7.7 Hz, 2H), 1.31 (s, 12H), 0.92 (d, *J* = 6.7 Hz, 6H), 0.69 (t, *J* = 7.8 Hz, 2H).

^{13}C NMR (151 MHz, $CDCl_3$) δ 174.27, 70.28, 70.21, 48.81, 36.75, 32.04, 31.80, 27.74, 20.08, 19.12, 15.30 (br).

HRMS m/z (ESI) calcd. for $C_{15}H_{30}BO_4^+$ $(M+H)^+$ 285.2232, found 285.2232.

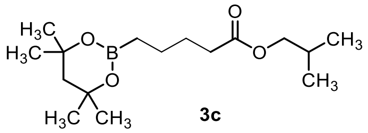

Isobutyl 5-(4,4,6,6-tetramethyl-1,3,2-dioxaborinan-2-yl)pentanoate (3c)

3c was prepared according to the general procedure for the borylation of ester substrates. The product was isolated as a colorless oil. Yield: 34%.

^{1}H NMR (600 MHz, $CDCl_3$) δ 3.84 (d, *J* = 6.7 Hz, 2H), 2.30 (t, *J* = 7.7 Hz, 2H), 1.92 (hept, *J* = 6.8 Hz, 1H), 1.78 (s, 2H), 1.65 – 1.57 (m, 2H), 1.39 (p, *J* = 7.6 Hz, 2H), 1.31 (s, 12H), 0.92 (d, *J* = 6.7 Hz, 6H), 0.67 (t, *J* = 7.8 Hz, 2H).

^{13}C NMR (151 MHz, $CDCl_3$) δ 173.22, 69.27, 69.18, 47.79, 33.42, 30.81, 30.78, 26.71, 26.68, 22.96, 18.10. 14.20 (br).

HRMS m/z (ESI) calcd. for $C_{16}H_{32}BO_4^+$ $(M+H)^+$ 299.2388, found 299.2389.

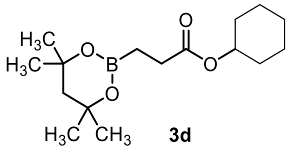

3d

Cyclohexyl 3-(4,4,6,6-tetramethyl-1,3,2-dioxaborinan-2-yl)propanoate (3d)
3d was prepared according to the general procedure for the borylation of ester substrates. The product was isolated as a colorless oil. Yield: 45%.
^1H NMR (600 MHz, $CDCl_3$) δ 4.72 (td, *J* = 9.1, 4.0 Hz, 1H), 2.34 (t, *J* = 7.5 Hz, 2H), 1.84 – 1.80 (m, 2H), 1.79 (s, 2H), 1.71 (dq, *J* = 8.6, 4.8 Hz, 2H), 1.56 – 1.48 (m, 1H), 1.43 – 1.32 (m, 3H), 1.30 (s, 12H), 1.27 – 1.21 (m, 2H), 0.92 (t, *J* = 7.5 Hz, 2H).

^{13}C NMR (151 MHz, $CDCl_3$) δ 174.77, 72.02, 70.36, 48.77, 31.82, 31.72, 31.58, 29.48, 25.47, 23.79, 10.52 (br).

HRMS m/z (ESI) calcd. for $C_{16}H_{30}BO_4^+$ $(M+H)^+$ 297.2232, found 297.2235.

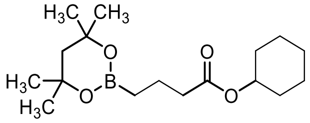

3e

Cyclohexyl 4-(4,4,6,6-tetramethyl-1,3,2-dioxaborinan-2-yl)butanoate (3e)
3e was prepared according to the general procedure for the borylation of ester substrates. The product was isolated as a colorless oil. Yield: 19%.
^1H NMR (600 MHz, $CDCl_3$) δ 4.74 (td, *J* = 9.1, 3.9 Hz, 1H), 2.30 – 2.23 (m, 2H), 1.85 – 1.80 (m, 2H), 1.79 (s, 2H), 1.75 – 1.64 (m, 4H), 1.57 – 1.49 (m, 1H), 1.45 – 1.33 (m, 4H), 1.31 (s, 12H), 1.29 – 1.26 (m, 1H), 0.69 (t, *J* = 7.9 Hz, 2H).

^{13}C NMR (151 MHz, $CDCl_3$) δ 173.67, 72.07, 70.27, 48.82, 37.14, 31.86, 31.81, 31.69, 25.46, 23.76, 20.13, 15.15 (br).

HRMS m/z (ESI) calcd. for $C_{17}H_{32}BO_4^+$ $(M+H)^+$ 311.2388, found 311.2388.

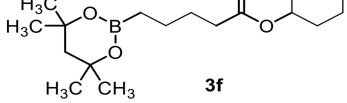

3f

Cyclohexyl 5-(4,4,6,6-tetramethyl-1,3,2-dioxaborinan-2-yl)pentanoate (3f)
3f was prepared according to the general procedure for the borylation of ester substrates. The product was isolated as a colorless oil. Yield: 28%.

^{1}H NMR (600 MHz, $CDCl_3$) δ 4.74 (td, *J* = 9.1, 3.9 Hz, 1H), 2.26 (t, *J* = 7.7 Hz, 2H), 1.86 – 1.80 (m, 2H), 1.79 (s, 2H), 1.74 – 1.68 (m, 2H), 1.64 – 1.50 (m, 4H), 1.42 – 1.34 (m, 6H), 1.31 (s, 12H), 0.67 (t, *J* = 7.8 Hz, 2H).

^{13}C NMR (151 MHz, $CDCl_3$) δ 173.64, 72.14, 70.20, 48.82, 34.83, 31.82, 31.67, 29.72, 27.79, 25.45, 23.95, 23.76, 15.40 (br).

HRMS m/z (ESI) calcd. for $C_{18}H_{34}BO_4^+$ $(M+H)^+$ 325.2545, found 325.2545.

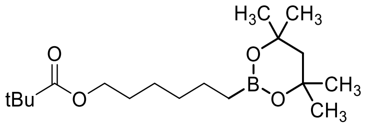

3g

6-(4,4,6,6-tetramethyl-1,3,2-dioxaborinan-2-yl)hexyl pivalate (3g)

3g was prepared according to the general procedure for the borylation of ester substrates. The product was isolated as a colorless oil. Yield: 64%.

^{1}H NMR (600 MHz, $CDCl_3$) δ 4.03 (t, *J* = 6.7 Hz, 2H), 1.79 (s, 2H), 1.64 – 1.55 (m, 4H), 1.38 – 1.33 (m, 4H), 1.32 (s, 12H), 1.19 (d, *J* = 1.0 Hz, 9H), 0.65 (t, *J* = 7.7 Hz, 2H).

^{13}C NMR (151 MHz, $CDCl_3$) δ 177.67, 69.11, 63.55, 47.81, 37.71, 30.98, 30.80, 28.69, 27.59, 26.20, 24.76, 23.19, 14.61 (br).

HRMS m/z (ESI) calcd. for $C_{18}H_{36}BO_4^+$ $(M+H)^+$ 327.2701, found 327.2702.

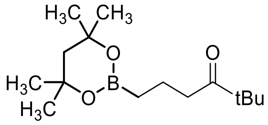

3h

2,2-dimethyl-6-(4,4,6,6-tetramethyl-1,3,2-dioxaborinan-2-yl)hexan-3-one (3h)

3h was prepared according to the general procedure for the borylation of other substrates. The product was isolated as a colorless oil. Yield: 45%.

^{1}H NMR (600 MHz, $CDCl_3$) δ 2.47 (t, *J* = 7.5 Hz, 2H), 1.79 (s, 2H), 1.62 (p, *J* = 7.7 Hz, 2H), 1.31 (s, 12H), 1.12 (s, 9H), 0.65 (t, *J* = 7.8 Hz, 2H).

^{13}C NMR (151 MHz, $CDCl_3$) δ 215.57, 69.22, 47.78, 43.04, 37.86, 30.81, 28.69, 25.35, 18.07. $-CH_2-B$ not observed.

HRMS m/z (ESI) calcd. for $C_{15}H_{30}BO_3^+$ $(M+H)^+$ 269.2283, found 269.2283.

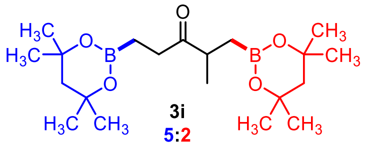

3i
5:2

2- and 4-methyl-1-(4,4,6,6-tetramethyl-1,3,2-dioxaborinan-2-yl)pentan-3-one (3i)

3i was prepared according to the general procedure for the borylation of other substrates.

The product was isolated as a colorless oil. Yield: 43% (5:2 mixture of two isomers).
^{1}H NMR (600 MHz, $CDCl_3$) δ 2.69 (h, *J* = 7.1 Hz, 1H), 2.61 (hept, *J* = 6.9 Hz, 1H), 2.53 (t, *J* = 6.9 Hz, 2H), 2.48 (qd, *J* = 7.2, 1.8 Hz, 2H), 1.78 (s, 2H), 1.77 (s, 2H), 1.30 – 1.27 (m, 17H), 1.08 (d, *J* = 6.9 Hz, 86H), 1.03 (t, *J* = 7.3 Hz, 3H), 1.01 – 0.97 (m, 1H), 0.82 (t, *J* = 6.9 Hz, 2H), 0.72 (dd, *J* = 15.8, 6.6 Hz, 1H).

^{13}C NMR (151 MHz, $CDCl_3$) δ 215.42, 215.12, 69.44, 69.28, 47.77, 47.75, 41.23, 39.31, 34.53, 32.52, 30.77, 30.74, 30.72, 28.75, 18.07, 17.56, 8.35 (br), 6.99.

HRMS m/z (ESI) calcd. for $C_{13}H_{26}BO_3^+$ $(M+H)^+$ 241.1970, found 241.1970.

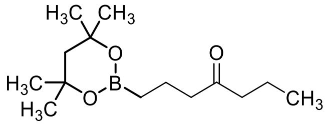

3j

1-(4,4,6,6-tetramethyl-1,3,2-dioxaborinan-2-yl)heptan-4-one (3j)

3j was prepared according to the general procedure for the borylation of other substrates. The product was isolated as a colorless oil. Yield: 50%.
^{1}H NMR (600 MHz, $CDCl_3$) δ 2.37 (q, *J* = 7.3 Hz, 4H), 1.79 (s, 2H), 1.64 (p, *J* = 7.7 Hz, 2H), 1.58 (p, *J* = 7.4 Hz, 2H), 1.31 (s, 12H), 0.90 (t, *J* = 7.4 Hz, 3H), 0.65 (t, *J* = 7.8 Hz, 2H).

^{13}C NMR (151 MHz, $CDCl_3$) δ 212.20, 70.28, 48.80, 45.37, 44.73, 31.81, 29.71, 19.04, 17.36, 15.18 (br), 13.81.

HRMS m/z (ESI) calcd. for $C_{14}H_{28}BO_3^+$ $(M+H)^+$ 255.2126, found 255.2126.

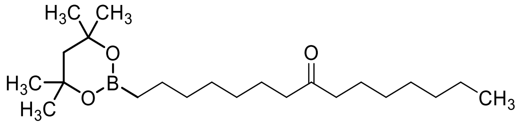

3k

1-(4,4,6,6-tetramethyl-1,3,2-dioxaborinan-2-yl)pentadecan-8-one (3k)

3k was prepared according to the general procedure for the borylation of other substrates. The product was isolated as a colorless oil. Yield: 24%.
^{1}H NMR (600 MHz, $CDCl_3$) δ 2.37 (td, *J* = 7.5, 2.6 Hz, 4H), 1.79 (s, 2H), 1.55 (dh, *J* = 11.0, 3.7 Hz, 2H), 1.36 – 1.33 (m, 2H), 1.32 (s, 12H), 1.30 – 1.23 (m, 18H), 0.90 – 0.85 (m, 3H), 0.63 (t, *J* = 7.7 Hz, 2H).

^{13}C NMR (151 MHz, $CDCl_3$) δ 211.91, 70.12, 48.84, 42.89, 42.83, 32.26, 31.83, 31.69, 31.59, 29.29, 29.25, 29.14, 29.10, 24.30, 23.98, 23.92, 22.62, 14.08. -CH_2-B not observed.

HRMS m/z (ESI) calcd. for $C_{22}H_{44}BO_3^+$ $(M+H)^+$ 367.3378, found 367.3377.

Note:
Compounds **3l**-**3r** were isolated only in sufficient quantity to obtain spectroscopic support for their synthesis. NMR yields are given in Figure S4 but the products were not isolated in preparative quantities, so isolated yields are not reported.

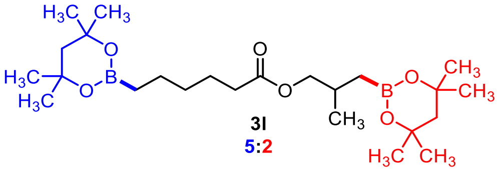

3l
5:2

Isobutyl 6-(4,4,6,6-tetramethyl-1,3,2-dioxaborinan-2-yl)hexanoate and 2-methyl-3-(4,4,6,6-tetramethyl-1,3,2-dioxaborinan-2-yl)propyl hexanoate (3l)

3l was prepared according to the general procedure for the borylation of ester substrates. NMR spectroscopic data is included below, but an isolated yield was not obtained. Crude NMR yield: 47% (5:2 mixture of two isomers).

^{1}H NMR (600 MHz, $CDCl_3$) δ 3.94 – 3.80 (m, 1H), 3.84 (d, *J* = 6.6 Hz, 2H), 2.29 (t, *J* = 7.6 Hz, 3H), 2.05 – 1.98 (m, 1H), 1.92 (hept, *J* = 6.7 Hz, 1H), 1.80 (s, 1H), 1.78 (s, 2H), 1.65 – 1.59 (m, 3H), 1.40 – 1.34 (m, 3H), 1.32 (s, 5H), 1.31 (s, 12H), 0.92 (d, *J* = 6.7 Hz, 6H), 0.88 (q, *J* = 7.1 Hz, 3H), 0.65 (t, *J* = 7.7 Hz, 2H).

^{13}C NMR (151 MHz, $CDCl_3$) δ 174.22, 174.18, 71.04, 70.43, 70.39, 70.22, 48.89, 34.55, 34.52, 32.00, 31.89, 31.87, 31.43, 29.78, 29.23, 27.80, 25.06, 24.83, 24.01, 22.77, 22.40, 19.24, 19.19, 14.20, 15.59 (br), 14.00.

HRMS m/z (ESI) calcd. for $C_{17}H_{34}BO_4^+$ $(M+H)^+$ 313.2545, found 313.2545.

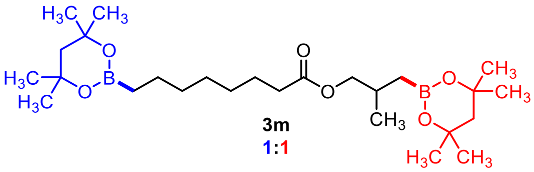

3m
1:1

Isobutyl 8-(4,4,6,6-tetramethyl-1,3,2-dioxaborinan-2-yl)octanoate and 2-methyl-3-(4,4,6,6-tetramethyl-1,3,2-dioxaborinan-2-yl)propyl octanoate (3m)

3m was prepared according to the general procedure for the borylation of ester substrates. NMR spectroscopic data is included below, but an isolated yield was not obtained. Crude NMR yield: 33% (1:1 mixture of two isomers).

^{1}H NMR (600 MHz, $CDCl_3$) δ 3.93 (dd, *J* = 10.5, 5.6 Hz, 1H), 3.84 (d, *J* = 6.7 Hz, 2H), 3.81 (dd, *J* = 10.5, 7.5 Hz, 1H), 2.29 (td, *J* = 7.5, 1.9 Hz, 4H), 2.01 (dh, *J* = 13.3, 6.6 Hz, 1H), 1.92 (hept, *J* = 6.8 Hz, 1H), 1.79 (s, 2H), 1.78 (s, 2H), 1.61 (pd, *J* = 7.1, 3.8 Hz, 6H), 1.32 (d, *J* = 2.5 Hz, 24H), 1.30 – 1.26 (m, 12H), 0.93 (dd, *J* = 6.7, 4.5 Hz, 9H), 0.89 – 0.85 (m, 3H), 0.76 (dd, *J* = 15.4, 5.7 Hz, 1H), 0.63 (t, *J* = 7.7 Hz, 2H), 0.54 (dd, *J* = 15.4, 8.7 Hz, 1H).

^{13}C NMR (151 MHz, $CDCl_3$) δ 174.11, 174.08, 70.97, 70.36, 70.35, 70.11, 48.84, 48.82, 34.49, 34.46, 32.22, 31.87, 31.83, 31.80, 31.68, 29.17, 29.15, 28.95, 27.73, 25.10, 25.08, 24.28, 22.61, 20.17 (br), 19.18, 19.11, 15.86 (br), 14.07.

HRMS m/z (ESI) calcd. for $C_{19}H_{38}BO_4^+$ $(M+H)^+$ 341.2858, found 341.2857.

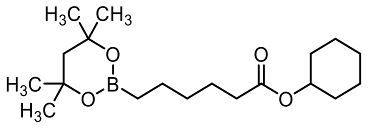

3n

Cyclohexyl 6-(4,4,6,6-tetramethyl-1,3,2-dioxaborinan-2-yl)hexanoate (3n)

3n was prepared according to the general procedure for the borylation of ester substrates. NMR spectroscopic data is included below, but an isolated yield was not obtained. Crude NMR yield: 36%.

^1H NMR (600 MHz, $CDCl_3$) δ 4.74 (td, *J* = 9.1, 3.9 Hz, 1H), 2.25 (t, *J* = 7.7 Hz, 2H), 1.82 (ddd, *J* = 10.2, 4.8, 2.5 Hz, 2H), 1.78 (s, 2H), 1.74 – 1.68 (m, 2H), 1.61 (p, *J* = 7.5 Hz, 2H), 1.43 – 1.20 (m, 10H), 1.31 (s, 12H), 0.64 (t, *J* = 7.7 Hz, 2H).

^{13}C NMR (151 MHz, $CDCl_3$) δ 173.53, 72.21, 70.15, 48.83, 34.89, 32.05, 31.92, 31.82, 31.68, 25.43, 25.07, 23.96, 23.79,15.70 (br).

HRMS m/z (ESI) calcd. for $C_{19}H_{36}BO_4^+$ $(M+H)^+$ 339.2701, found 339.2704.

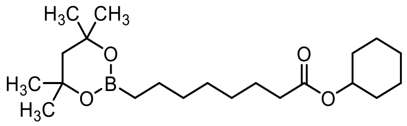

3o

Cyclohexyl 8-(4,4,6,6-tetramethyl-1,3,2-dioxaborinan-2-yl)octanoate (3o)

3o was prepared according to the general procedure for the borylation of ester substrates. NMR spectroscopic data is included below, but an isolated yield was not obtained. Crude NMR yield: 22%.

^1H NMR (600 MHz, $CDCl_3$) δ 4.75 (td, *J* = 9.0, 3.9 Hz, 1H), 2.26 (t, *J* = 7.6 Hz, 2H), 1.85 – 1.80 (m, 2H), 1.79 (s, 2H), 1.74 – 1.68 (m, 2H), 1.63 – 1.58 (m, 2H), 1.55 – 1.51 (m, 2H), 1.43 – 1.33 (m, 6H), 1.32 (s, 12H), 1.30 – 1.26 (m, 6H), 0.64 (t, *J* = 7.7 Hz, 2H).

^{13}C NMR (151 MHz, $CDCl_3$) δ 173.47, 72.25, 70.11, 48.85, 34.85, 32.23, 31.83, 31.67, 29.71, 29.17, 29.13, 25.43, 25.18, 24.29, 23.77. -CH_2-B not observed.

HRMS m/z (ESI) calcd. for $C_{21}H_{40}BO_4^+$ $(M+H)^+$ 367.3014, found 367.3018.

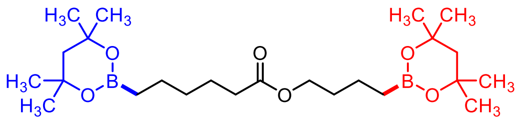

3p
2:3

Butyl 6-(4,4,6,6-tetramethyl-1,3,2-dioxaborinan-2-yl)hexanoate and 4-(4,4,6,6-tetramethyl-1,3,2-dioxaborinan-2-yl)butyl hexanoate (3p)

3p was prepared according to the general procedure for the borylation of other substrates. NMR spectroscopic data is included below, but an isolated yield was not obtained. Crude NMR yield: 70% (2:3 ratio of two isomers).

^{1}H NMR (600 MHz, $CDCl_3$) δ 4.05 (td, *J* = 6.7, 3.1 Hz, 2H), 2.28 (td, *J* = 7.7, 3.0 Hz, 2H), 1.79 (s, 2H), 1.78 (s, 2H), 1.65 – 1.57 (m, 4H), 1.45 – 1.34 (m, 4H), 1.31 (d, *J* = 1.8 Hz, 12H), 1.30 – 1.27 (m, 2H), 0.93 (t, *J* = 7.4 Hz, 3H), 0.89 (t, *J* = 7.1 Hz, 3H), 0.68 (t, *J* = 7.8 Hz, 2H), 0.64 (t, *J* = 7.7 Hz, 2H).

^{13}C NMR (151 MHz, $CDCl_3$) δ 174.15, 174.05, 70.21, 70.13, 64.53, 64.03, 48.79, 34.46, 34.40, 31.89, 31.79, 31.56, 31.32, 31.19, 30.70, 29.69, 24.94, 24.70, 23.91, 22.68, 22.31, 20.72, 19.14, 15.44 (br), 14.11, 13.91, 13.71.

HRMS m/z (ESI) calcd. for $C_{17}H_{34}BO_4^+$ (M+H)$^+$ 313.2545, found 313.2548.

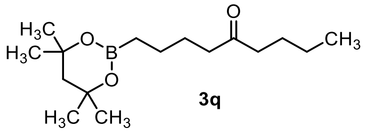

3q

1-(4,4,6,6-tetramethyl-1,3,2-dioxaborinan-2-yl)nonan-5-one (3q)

3q was prepared according to the general procedure for the borylation of other substrates. NMR spectroscopic data is included below, but an isolated yield was not obtained. Crude NMR yield: 62%.

^{1}H NMR (600 MHz, $CDCl_3$) δ 2.38 (td, *J* = 7.5, 3.6 Hz, 4H), 1.79 (s, 2H), 1.55 (dtd, *J* = 15.2, 7.5, 5.3 Hz, 4H), 1.35 (p, *J* = 7.7 Hz, 2H), 1.31 (s, 12H), 1.31 – 1.28 (m, 2H), 0.90 (t, *J* = 7.4 Hz, 3H), 0.66 (t, *J* = 7.8 Hz, 2H).

^{13}C NMR (151 MHz, $CDCl_3$) δ 212.10, 70.21, 48.82, 42.92, 42.42, 31.82, 26.63, 26.02, 24.06, 22.40, 13.89. $-CH_2$-B not observed.

HRMS m/z (ESI) calcd. for $C_{16}H_{32}BO_3^+$ (M+H)$^+$ 283.2439, found 283.2442.

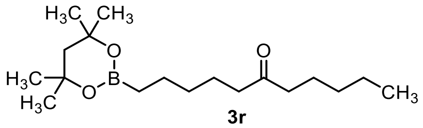

3r

1-(4,4,6,6-tetramethyl-1,3,2-dioxaborinan-2-yl)undecan-6-one (3r)

3r was prepared according to the general procedure for the borylation of other substrates. NMR spectroscopic data is included below, but an isolated yield was not obtained. Crude NMR yield: 39%.

^{1}H NMR (600 MHz, $CDCl_3$) δ 2.37 (td, *J* = 7.6, 4.6 Hz, 4H), 1.79 (s, 2H), 1.55 (q, *J* = 7.4 Hz, 8H), 1.39 – 1.33 (m, 2H), 1.31 (s, 12H), 1.30 – 1.27 (m, 2H), 0.88 (t, *J* = 7.2 Hz, 3H), 0.64 (t, *J* = 7.8 Hz, 2H).

^{13}C NMR (151 MHz, $CDCl_3$) δ 211.98, 70.16, 48.83, 42.95, 42.70, 32.02, 31.83, 31.48, 29.71, 24.08, 23.93, 23.59, 22.48, 13.94. -CH_2-B not observed.

HRMS m/z (ESI) calcd. for $C_{18}H_{36}BO_3^+$ $(M+H)^+$ 311.2752, found 311.2753.

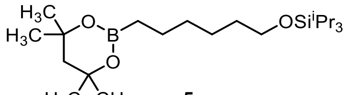

5a

Triisopropyl((6-(4,4,6,6-tetramethyl-1,3,2-dioxaborinan-2-yl)hexyl)oxy)silane (5a)

5a was prepared according to the general procedure for the borylation of other substrates. The product was isolated as a colorless oil. Yield: 56%.

^{1}H NMR (600 MHz, $CDCl_3$) δ 3.65 (t, *J* = 6.8 Hz, 2H), 1.79 (s, 2H), 1.57 – 1.49 (m, 2H), 1.38 – 1.25 (m, 6H), 1.32 (s, 12H), 1.01 – 1.07 (m, 21H), 0.64 (t, *J* = 7.7 Hz, 2H).

^{13}C NMR (151 MHz, $CDCl_3$) δ 70.10, 63.66, 48.85, 33.12, 32.31, 31.83, 25.72, 24.38, 18.05, 15.69 (br), 12.04.

GCMS m/z (EI, 70 eV): 383.3 $(C_{21}H_{44}BO_3Si)^+$ $[M-CH_3]^+$

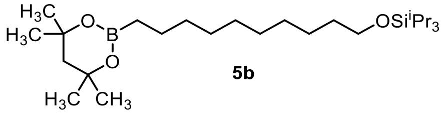

5b

Triisopropyl((10-(4,4,6,6-tetramethyl-1,3,2-dioxaborinan-2-yl)decyl)oxy)silane (5b)

5b was prepared according to the general procedure for the borylation of other substrates. The product was isolated as a colorless oil. Yield: 26%.

^{1}H NMR (600 MHz, $CDCl_3$) δ 3.66 (t, *J* = 6.7 Hz, 2H), 1.79 (s, 2H), 1.56 – 1.49 (m, 2H), 1.32 (s, 12H), 1.30 – 1.23 (m, 14 H), 1.01 – 1.08 (d, *J* = 4.8 Hz, 21H), 0.64 (t, *J* = 7.8 Hz, 2H).

^{13}C NMR (151 MHz, $CDCl_3$) δ 70.09, 63.59, 48.86, 33.10, 32.45, 31.84, 29.72, 29.67, 29.54, 25.86, 24.39, 18.05, 15.85 (br), 12.04.

GCMS m/z (EI, 70 eV): 439.4 $(C_{25}H_{52}BO_3Si)^+$ $[M-CH_3]^+$

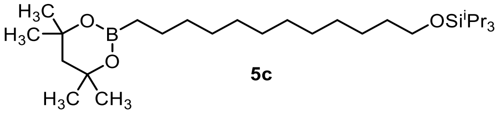

Triisopropyl((12-(4,4,6,6-tetramethyl-1,3,2-dioxaborinan-2-yl)dodecyl)oxy)silane (5c)
5c was prepared according to the general procedure for the borylation of other substrates. The product was isolated as a colorless oil. Yield: 26%.
^{1}H NMR (600 MHz, $CDCl_3$) δ 3.66 (t, *J* = 6.7 Hz, 2H), 1.79 (s, 2H), 1.56 – 1.48 (m, 2H), 1.32 (s, 12H), 1.29 – 1.22 (m, 18H), 1.01 – 1.07 (m, 21H), 0.64 (t, *J* = 7.8 Hz, 2H).

^{13}C NMR (151 MHz, $CDCl_3$) δ 70.09, 63.55, 48.86, 33.08, 32.48, 31.83, 29.71, 29.69, 29.65, 29.61, 29.58, 29.52, 25.84, 24.40, 18.05, 15.78 (br), 12.04.

GCMS m/z (EI, 70 eV): 467.5 $(C_{27}H_{56}BO_3Si)^+$ $[M-CH_3]^+$

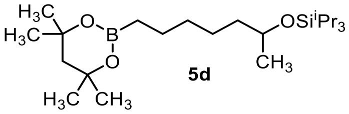

Triisopropyl((7-(4,4,6,6-tetramethyl-1,3,2-dioxaborinan-2-yl)heptan-2-yl)oxy)silane
5d was prepared according to the general procedure for the borylation of other substrates. The product was isolated as a colorless oil. Yield: 43%.
^{1}H NMR (600 MHz, $CDCl_3$) δ 3.89 (h, *J* = 6.1 Hz, 1H), 1.79 (s, 2H), 1.53 – 1.47 (m, 2H), 1.32 (s, 12H), 1.30 – 1.25 (m, 6H), 1.14 (d, *J* = 6.0 Hz, 3H), 1.05 (s, 21H), 0.64 (t, *J* = 7.8 Hz, 2H).

^{13}C NMR (151 MHz, $CDCl_3$) δ 68.51, 63.04, 39.91, 32.83, 29.71, 25.97, 25.13, 23.50, 18.18, 18.15, 12.50. $-CH_2-B$ not observed.

GCMS m/z (EI, 70 eV): 397.4 $(C_{22}H_{46}BO_3Si)^+$ $[M-CH_3]^+$

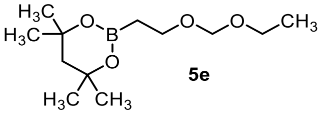

2-(2-(ethoxymethoxy)ethyl)-4,4,6,6-tetramethyl-1,3,2-dioxaborinane (5e)
5e was prepared according to the general procedure for the borylation of other substrates. The product was isolated as a colorless oil. Yield: 39%.
^{1}H NMR (600 MHz, $CDCl_3$) δ 4.67 (s, 2H), 3.64 (t, *J* = 8.0 Hz, 2H), 3.60 (q, *J* = 7.1 Hz, 2H), 1.80 (s, 2H), 1.31 (s, 12H), 1.22 (t, *J* = 7.1 Hz, 3H), 1.06 (t, *J* = 8.1 Hz, 2H).

^{13}C NMR (151 MHz, $CDCl_3$) δ 93.81, 69.41, 64.32, 61.82, 47.81, 30.75, 28.69, 16.22 (br), 14.17.

HRMS m/z (ESI) calcd. for $C_{12}H_{26}BO_4^+$ $(M+H)^+$ 245.1919, found 245.1901.

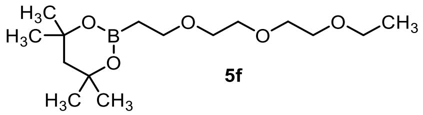

5f

2-(2-(2-(2-ethoxyethoxy)ethoxy)ethyl)-4,4,6,6-tetramethyl-1,3,2-dioxaborinane (5f)

5f was prepared according to the general procedure for the borylation of other substrates. The product was isolated as a colorless oil. Yield: 59%.

^{1}H NMR (600 MHz, $CDCl_3$) δ 3.66 – 3.61 (m, 4H), 3.60 – 3.54 (m, 6H), 3.52 (q, *J* = 7.0 Hz, 2H), 1.79 (s, 2H), 1.31 (s, 12H), 1.20 (t, *J* = 7.1 Hz, 3H), 1.06 (t, *J* = 8.1 Hz, 2H).

^{13}C NMR (151 MHz, $CDCl_3$) δ 70.66, 70.62, 70.38, 69.86, 69.54, 68.63, 66.64, 48.83, 32.04, 31.77, 17.22 (br), 15.16.

HRMS m/z (ESI) calcd. for $C_{15}H_{32}BO_5^+$ $(M+H)^+$ 303.2337, found 303.2341.

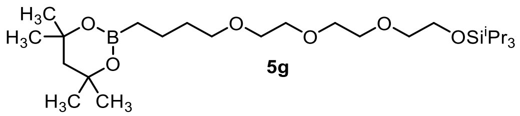

5g

3,3-diisopropyl-2-methyl-17-(4,4,6,6-tetramethyl-1,3,2-dioxaborinan-2-yl)-4,7,10,13-tetraoxa-3-silaheptadecane (5g)

5g was prepared according to the general procedure for the borylation of other substrates with some modification. In an inert atmosphere glovebox, to a 8 mL screw cap glass culture tube with a stir bar was added $[(coe)_2IrCl]_2$ (2.7 mg, 0.003 mmol, 0.015 eq.), **L2** (1.8 mg, 0.006 mmol, 0.03 eq.), Et_3SiH (1.5 uL, 0.009 mmol, 0.045 eq.), B_2dmpd_2 (56.4 mg, 0.2 mmol, 1 eq.), and cyclopentane (1 mL). The tube was capped well with a septum cap, removed from the glovebox and was heated in a 120 °C oil bath with stirring for 30 min. After 30 minutes, the substrate **4g** was taken up in 0.5 mL cyclopentane and added through the septum using a syringe. (We find that this substrate leads to precipitate formation and catalyst poisoning if present for precatalyst activation.) The reaction was further stirred while heating for 24 hr. The crude mixture was then concentrated in vacuo and purified by column chromatography on silica gel to obtain the desired product as a colorless oil. Yield: 40%.

^{1}H NMR (600 MHz, $CDCl_3$) δ 3.83 (t, *J* = 5.7 Hz, 2H), 3.70 – 3.61 (m, 6H), 3.60 – 3.54 (m, 4H), 3.44 (t, *J* = 7.0 Hz, 2H), 1.78 (s, 2H), 1.57 (p, *J* = 7.2 Hz, 2H), 1.42 – 1.36 (m, 2H), 1.31 (s, 12H), 1.07 – 1.00 (m, 21H), 0.66 (t, *J* = 7.9 Hz, 2H).

^{13}C NMR (151 MHz, $CDCl_3$) δ 72.72, 71.60, 70.83, 70.68, 70.66, 70.15, 69.97, 62.94, 48.83, 32.18, 31.83, 29.71, 20.77, 17.97, 15.52 (br), 11.96.

HRMS m/z (ESI) calcd. for $C_{26}H_{56}BO_6Si^+$ $(M+H)^+$ 503.3934, found 503.3935.

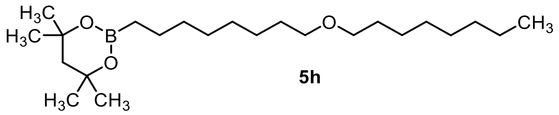

5h

4,4,6,6-tetramethyl-2-(8-(octyloxy)octyl)-1,3,2-dioxaborinane (5h)

5h was prepared according to the general procedure for the borylation of other substrates. The product was isolated as a colorless oil. Yield: 58%.

^{1}H NMR (600 MHz, $CDCl_3$) δ 3.38 (td, *J* = 6.8, 1.5 Hz, 4H), 1.79 (s, 2H), 1.58 – 1.52 (m, 4H), 1.32 (s, 12H), 1.30 – 1.22 (m, 20H), 0.88 (t, *J* = 7.1 Hz, 3H), 0.64 (t, *J* = 7.7 Hz, 2H).

^{13}C NMR (151 MHz, $CDCl_3$) δ 71.03, 70.97, 70.09, 48.85, 32.41, 31.86, 31.84, 29.80, 29.71, 29.52, 29.49, 29.37, 29.29, 26.22, 24.36, 22.67, 15.60 (br), 14.12.

HRMS m/z (ESI) calcd. for $C_{23}H_{48}BO_3^+$ $(M+H)^+$ 383.3691, found 383.3696.

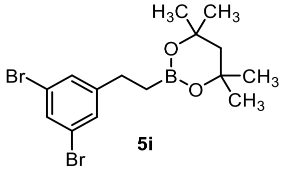

5i

2-(3,5-dibromophenethyl)-4,4,6,6-tetramethyl-1,3,2-dioxaborinane (5i)

5i was prepared according to the general procedure for the borylation of other substrates. The product was isolated as a colorless oil. Yield: 64%.

^{1}H NMR (600 MHz, $CDCl_3$) δ 7.43 (s, 1H), 7.31 (s, 2H), 2.64 (t, *J* = 7.8 Hz, 2H), 1.77 (s, 2H), 1.29 (s, 12H), 0.99 (t, *J* = 7.7 Hz, 3H).

^{13}C NMR (151 MHz, $CDCl_3$) δ 149.22, 130.79, 130.27, 122.38, 70.54, 48.77, 31.79, 31.77, 29.84, 16.97 (br).

GCMS m/z (EI, 70 eV): 404.0 $(C_{15}H_{21}BBr_2O_2)^+$ $[M]^+$

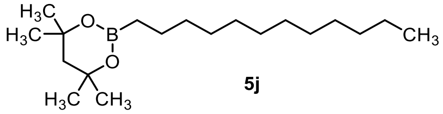

5j

2-dodecyl-4,4,6,6-tetramethyl-1,3,2-dioxaborinane (5j)

5j was prepared according to the general procedure for the borylation of other substrates. The product was isolated as a colorless oil. Yield: 45%.

^{1}H NMR (600 MHz, $CDCl_3$) δ 1.79 (s, 2H), 1.32 (s, 12H), 1.25 (s, 20H), 0.88 (t, *J* = 7.1 Hz, 3H), 0.64 (t, *J* = 7.8 Hz, 2H).

^{13}C NMR (151 MHz, $CDCl_3$) δ 70.09, 48.86, 32.47, 32.40, 31.94, 31.84, 29.72, 29.69, 29.58, 29.47, 29.39, 29.37, 24.40, 22.70, 15.74 (br), 14.14.

GCMS m/z (EI, 70 eV): 310.3 $(C_{19}H_{39}BO_2)^+$ $[M]^+$, 295.1 $(C_{18}H_{36}BO_2)^+$ $[M-CH_3]^+$

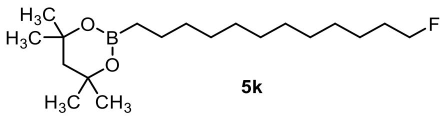

2-(12-fluorododecyl)-4,4,6,6-tetramethyl-1,3,2-dioxaborinane (5k)
5k was prepared according to the general procedure for the borylation of other substrates. The product was isolated as a colorless oil. Yield: 34%.
^{1}H NMR (500 MHz, $CDCl_3$) δ 4.43 (dt, *J* = 47.3, 6.2 Hz, 2H), 1.79 (s, 2H), 1.68 (dt, *J* = 24.7, 7.6 Hz, 2H), 1.32 (s, 18H), 1.25 (s, 12H), 0.64 (t, *J* = 7.8 Hz, 2H).

^{13}C NMR (126 MHz, $CDCl_3$) δ 84.85, 83.55, 70.00, 48.77, 32.36, 31.74, 30.41, 30.25, 29.56, 29.48, 29.46, 29.18, 25.07, 25.03, 24.29. -CH_2-B not observed.

^{19}F NMR (471 MHz, $CDCl_3$) δ -217.92.
GCMS m/z (EI, 70 eV): 328.3 $(C_{19}H_{38}BFO_2)^+$ $[M]^+$, 313.2 $(C_{18}H_{35}BFO_2)^+$ $[M-CH_3]^+$

Note:
Compounds **5l**-**5r** were isolated only in sufficient quantity to obtain spectroscopic support for their synthesis. NMR yields are given in Figure S4 but the products were not isolated in preparative quantities, so isolated yields are not reported.

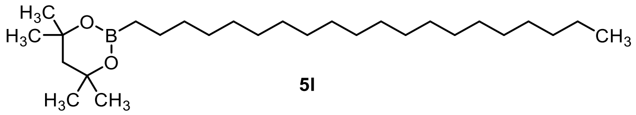

2-icosyl-4,4,6,6-tetramethyl-1,3,2-dioxaborinane (5l)
5l was prepared from eicosane according to the general procedure for the borylation of other substrates. NMR spectroscopic data is included below, but an isolated yield was not obtained. Crude NMR yield: 55%.
^{1}H NMR (600 MHz, $CDCl_3$) δ 1.79 (s, 2H), 1.32 (s, 12H), 1.25 (s, 36H), 0.88 (t, *J* = 7.1 Hz, 3H), 0.64 (t, *J* = 7.7 Hz, 2H).

^{13}C NMR (151 MHz, $CDCl_3$) δ 69.07, 53.03, 47.83, 31.42, 30.85, 30.82, 30.60, 28.69, 28.62, 28.57, 28.31, 26.70, 26.65, 25.72, 23.35, 21.66, 15.89 (br), 13.10.

GCMS m/z (EI, 70 eV): 422.5 $(C_{27}H_{55}BO_2)^+$ $[M]^+$, 407.4 $(C_{26}H_{52}BO_2)^+$ $[M-CH_3]^+$

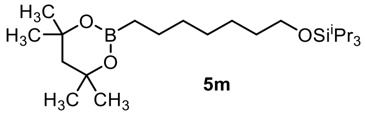

Triisopropyl((7-(4,4,6,6-tetramethyl-1,3,2-dioxaborinan-2-yl)heptyl)oxy)silane (5m)
5m was prepared according to the general procedure for the borylation of other substrates. NMR spectroscopic data is included below, but an isolated yield was not obtained. Crude NMR yield: 56%.

^{1}H NMR (600 MHz, $CDCl_3$) δ 3.66 (t, *J* = 6.8 Hz, 2H), 1.79 (s, 2H), 1.53 (p, *J* = 6.6 Hz, 2H), 1.38 – 1.33 (m, 2H), 1.32 (s, 12H), 1.30 – 1.26 (m, 6H), 1.05 (d, *J* = 4.7 Hz, 21H), 0.64 (t, *J* = 7.7 Hz, 2H).

^{13}C NMR (151 MHz, $CDCl_3$) δ 70.10, 63.58, 48.85, 33.12, 32.46, 31.92, 31.83, 29.41, 25.81, 24.34, 18.05, 16.01 (br), 12.04.

GCMS m/z (EI, 70 eV): 397.3 $(C_{22}H_{46}BO_3Si)^+$ $[M-CH_3]^+$

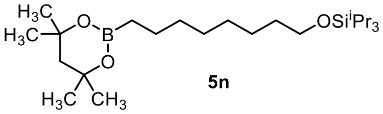

5n

Triisopropyl((8-(4,4,6,6-tetramethyl-1,3,2-dioxaborinan-2-yl)octyl)oxy)silane (5n)
5n was prepared according to the general procedure for the borylation of other substrates. NMR spectroscopic data is included below, but an isolated yield was not obtained. Crude NMR yield: 53%.
^{1}H NMR (600 MHz, $CDCl_3$) δ 3.66 (t, *J* = 6.7 Hz, 2H), 1.79 (s, 2H), 1.55 – 1.50 (m, 2H), 1.35 (dd, *J* = 4.5, 3.3 Hz, 2H), 1.32 (s, 12H), 1.27 (t, *J* = 2.9 Hz, 8H), 1.05 (d, *J* = 4.9 Hz, 21H), 0.64 (t, *J* = 7.8 Hz, 2H).

^{13}C NMR (151 MHz, $CDCl_3$) δ 70.09, 63.58, 48.86, 33.10, 32.44, 31.84, 29.60, 29.52, 25.88, 24.38, 22.73, 18.05, 12.04. -CH_2-B not observed.

GCMS m/z (EI, 70 eV): 411.4 $(C_{23}H_{48}BO_3Si)^+$ $[M-CH_3]^+$

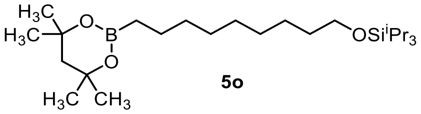

5o

Triisopropyl((9-(4,4,6,6-tetramethyl-1,3,2-dioxaborinan-2-yl)nonyl)oxy)silane (5o)
5o was prepared according to the general procedure for the borylation of other substrates. NMR spectroscopic data is included below, but an isolated yield was not obtained. Crude NMR yield: 48%.
^{1}H NMR (600 MHz, $CDCl_3$) δ 3.66 (t, *J* = 6.8 Hz, 2H), 1.79 (s, 2H), 1.54 – 1.49 (m, 2H), 1.32 (s, 12H), 1.31 – 1.26 (m, 12H), 1.05 (d, *J* = 4.9 Hz, 21H), 0.64 (t, *J* = 7.8 Hz, 2H).

^{13}C NMR (151 MHz, $CDCl_3$) δ 70.09, 63.59, 48.86, 33.10, 32.45, 31.84, 29.72, 29.67, 29.54, 25.86, 24.39, 18.05, 12.04. -CH_2-B not observed.

GCMS m/z (EI, 70 eV): 425.4 $(C_{24}H_{50}BO_3Si)^+$ $[M-CH_3]^+$

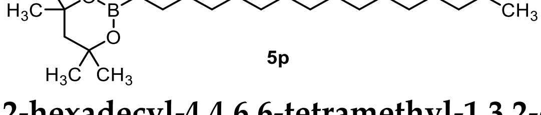

5p

2-hexadecyl-4,4,6,6-tetramethyl-1,3,2-dioxaborinane (5p)

5p was prepared according to the general procedure for the borylation of other substrates. NMR spectroscopic data is included below, but an isolated yield was not obtained. Crude NMR yield: 54%.

^{1}H NMR (600 MHz, $CDCl_3$) δ 1.79 (s, 2H), 1.32 (s, 12H), 1.25 (s, 28H), 0.88 (t, *J* = 7.1 Hz, 3H), 0.64 (t, *J* = 7.7 Hz, 2H).

^{13}C NMR (151 MHz, $CDCl_3$) δ 70.09, 48.86, 32.48, 31.94, 31.84, 29.72, 29.69, 29.67, 29.58, 29.38, 24.40, 22.71, 14.14. -CH_2-B not observed.

GCMS m/z (EI, 70 eV): 366.4 $(C_{23}H_{47}BO_2)^+$ $[M]^+$, 351.3 $(C_{22}H_{44}BO_2)^+$ $[M-CH_3]^+$

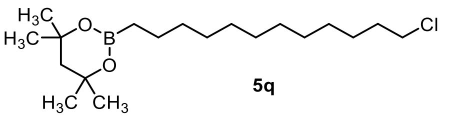

5q

2-(12-chlorododecyl)-4,4,6,6-tetramethyl-1,3,2-dioxaborinane (5q)

5q was prepared according to the general procedure for the borylation of other substrates. NMR spectroscopic data is included below, but an isolated yield was not obtained. Crude NMR yield: 30%.

^{1}H NMR (600 MHz, $CDCl_3$) δ 3.53 (t, *J* = 6.8 Hz, 2H), 1.79 (s, 2H), 1.78 – 1.73 (m, 2H), 1.44 – 1.38 (m, 2H), 1.32 (s, 12H), 1.25 (s, 16H), 0.64 (t, *J* = 7.8 Hz, 2H).

^{13}C NMR (151 MHz, $CDCl_3$) δ 69.06, 68.23, 47.82, 44.19, 31.65, 31.42, 30.85, 30.80, 30.78, 28.68, 28.61, 28.57, 28.53, 28.46, 28.03, 27.89, 25.88, 23.35. -CH_2-B not observed.

GCMS m/z (EI, 70 eV): 344.2 $(C_{19}H_{38}BClO_2)^+$ $[M]^+$, 329.2 $(C_{18}H_{35}BClO_2)^+$ $[M-CH_3]^+$

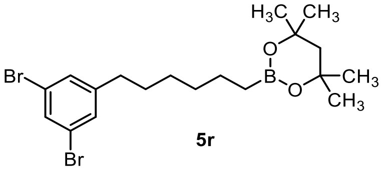

5r

2-(6-(3,5-dibromophenyl)hexyl)-4,4,6,6-tetramethyl-1,3,2-dioxaborinane (5r)

5r was prepared according to the general procedure for the borylation of other substrates. NMR spectroscopic data is included below, but an isolated yield was not obtained. Crude NMR yield: 45%.

^{1}H NMR (600 MHz, $CDCl_3$) δ 7.47 (s, 1H), 7.25 (s, 2H), 2.53 (t, *J* = 7.8 Hz, 2H), 1.79 (s, 2H), 1.56 (q, *J* = 7.4 Hz, 2H), 1.35 (t, *J* = 7.2 Hz, 2H), 1.32 (s, 12H), 1.29 (dd, *J* = 6.7, 3.7 Hz, 4H), 0.64 (t, *J* = 7.7 Hz, 2H).

^{13}C NMR (151 MHz, $CDCl_3$) δ 147.05, 131.22, 130.29, 122.64, 70.15, 48.84, 35.40, 32.10, 31.84, 31.00, 29.72, 29.00, 24.19. -CH_2-B not observed.

GCMS m/z (EI, 70 eV): 460.1 $(C_{19}H_{29}BBr_2O_2)^+$ $[M]^+$, 443.1 $(C_{18}H_{26}BBr_2O_2)^+$ $[M-CH_3]^+$

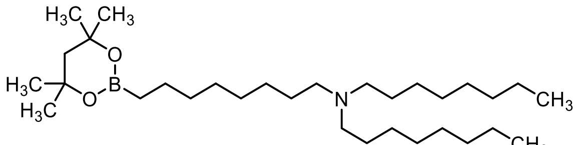

5s

N,N-dioctyl-8-(4,4,6,6-tetramethyl-1,3,2-dioxaborinan-2-yl)octan-1-amine (5s)

5s was prepared according to the general procedure for the borylation of other substrates. NMR spectroscopic data is included below, but an isolated yield was not obtained. Crude NMR yield: 66%.

^{1}H NMR (600 MHz, $CDCl_3$) δ 2.40 (t, *J* = 7.8 Hz, 6H), 1.78 (s, 2H), 1.46 – 1.38 (m, 6H), 1.32 (s, 12H), 1.29 – 1.22 (m, 30H), 0.88 (t, *J* = 7.0 Hz, 6H), 0.64 (t, *J* = 7.8 Hz, 2H).

^{13}C NMR (151 MHz, $CDCl_3$) δ 69.07, 53.03, 47.83, 31.42, 30.85, 30.82, 30.60, 28.69, 28.62, 28.57, 28.31, 26.70, 26.65, 25.72, 23.35, 21.66, 14.83 (br), 13.10.

HRMS m/z (ESI) calcd. for $C_{31}H_{65}BNO_2^+$ $(M+H)^+$ 494.5103, found 494.5114.

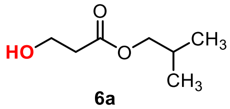

6a

Isobutyl 3-hydroxypropanoate (6a)

6a was prepared according to the general procedure for the oxidation of borylated substrates. The product was isolated as a colorless oil. Yield: 95%.

^{1}H NMR (600 MHz, $CDCl_3$) δ 3.90 (d, *J* = 6.7 Hz, 2H), 3.87 (t, *J* = 5.6 Hz, 2H), 2.58 (t, *J* = 5.6 Hz, 2H), 1.94 (dp, *J* = 13.4, 6.8 Hz, 1H), 0.93 (d, *J* = 6.7 Hz, 6H).

^{13}C NMR (151 MHz, $CDCl_3$) δ 173.04, 70.83, 58.33, 36.71, 27.67, 19.06.

HRMS m/z (ESI) calcd. for $C_7H_{15}O_3^+$ $(M+H)^+$ 147.1016, found 169.0861.

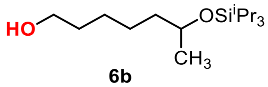

6b

6-((triisopropylsilyl)oxy)heptan-1-ol (6b)

6b was prepared according to a combination of two general procedures. First, **5d** was prepared according to the general procedure for the borylation of other substrates. Once finished, the reaction was cooled to room temperature and concentrated in vacuo to remove solvent. Then, the mixture was flushed through a silica plug with 10:1 ratio of hexane and EtOAc to remove any metal residue. The crude solution was then concentrated in vacuo and used as starting material in the general procedure for the oxidation of borylated substrates. The product was isolated as a colorless oil. Yield: 73%.

^{1}H NMR (600 MHz, $CDCl_3$) δ 3.92 (h, *J* = 6.1 Hz, 1H), 3.64 (t, *J* = 6.6 Hz, 2H), 1.61 – 1.55 (m, 2H), 1.54 – 1.39 (m, 2H), 1.39 – 1.32 (m, 4H), 1.15 (d, *J* = 6.1 Hz, 3H), 1.05 (s, 21H).

^{13}C NMR (151 MHz, $CDCl_3$) δ 68.51, 63.04, 39.91, 32.83, 29.71, 25.97, 25.13, 23.50, 18.18, 12.50.

HRMS m/z (ESI) calcd. for $C_{16}H_{37}O_2Si^+$ $(M+H)^+$ 289.2557, found 289.2559.

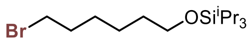

6c

((6-bromohexyl)oxy)triisopropylsilane (6c)

6c was prepared by a variation on a reported procedure.[8] To a 20 mL vial with a stir bar was added 3,5-Bis(trifluoromethyl)bromobenzene (44.0 mg, 0.15 mmol, 1.5 eq.) and THF (1 mL). nBuLi (60 uL, 0.15 mmol, 1.5 eq.) was slowly added while stirring under -78 °C. After stirring for 1 h, **5a** (39.9 mg, 0.1 mmol, 1 eq) in 1 mL THF was added. The reaction was stirring for another 1 h while -78 ˚C was removed. Next, NBS (33.9 mg, 0.15 mmol, 1.5 eq.) was added, and the reaction was stirring at room temperature until completion (determined by TLC). Upon completion, the reaction was quenched with saturated sodium thiosulfate solution, extracted with EA, dried over Na_2SO_4, concentrated in vacuo and purified by column chromatography on silica gel to obtain the desired product **6c** as a colorless oil. Yield: 56%. The spectral data for this known compound matches previous reports.[8]

^{1}H NMR (400 MHz, $CDCl_3$) δ 3.68 (t, *J* = 6.4 Hz, 2H), 3.41 (t, *J* = 6.9 Hz, 2H), 1.87 (p, *J* = 6.9 Hz, 2H), 1.60 – 1.33 (m, 6H), 1.09 – 1.00 (m, 21H).

^{13}C NMR (151 MHz, $CDCl_3$) δ 63.37, 34.10, 32.99, 32.93, 28.18, 25.21, 18.19, 12.16.

GCMS m/z (EI, 70 eV): 293.1, 295.1 $(C_{12}H_{26}BrOSi)^+$ $[M\text{-}iPr]^+$.

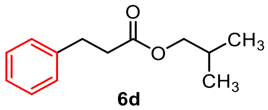

6d

Isobutyl 3-phenylpropanoate (6d)

6d was prepared according to the general procedure for the cross-coupling reaction. The product was isolated as a colorless oil. Yield: 41%.

^{1}H NMR (600 MHz, $CDCl_3$) δ 7.30 – 7.27 (m, 2H), 7.22 – 7.17 (m, 3H), 3.85 (d, *J* = 6.7 Hz, 2H), 2.96 (t, *J* = 7.9 Hz, 2H), 2.66 – 2.63 (m, 2H), 1.90 (dp, *J* = 13.4, 6.8 Hz, 1H), 0.90 (d, *J* = 6.7 Hz, 6H).

^{13}C NMR (151 MHz, $CDCl_3$) δ 68.51, 63.04, 39.91, 32.83, 29.71, 25.97, 25.13, 23.50, 18.18, 18.15, 12.50.

HRMS m/z (ESI) calcd. for $C_{13}H_{19}O_2^+$ $(M+H)^+$ 207.1380, found 207.1384.

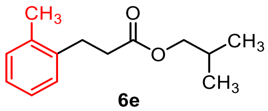

6e

Isobutyl 3-(o-tolyl)propanoate (6e)

6e was prepared according to the general procedure for the cross-coupling reaction. The product was isolated as a colorless oil. Yield: 54%.

^{1}H NMR (600 MHz, $CDCl_3$) δ 7.13 (tt, *J* = 6.3, 2.9 Hz, 4H), 3.87 (d, *J* = 6.7 Hz, 2H), 2.98 – 2.92 (m, 2H), 2.63 – 2.57 (m, 2H), 2.33 (s, 3H), 1.92 (hept, *J* = 6.7 Hz, 1H), 0.91 (d, *J* = 6.7 Hz, 6H).

^{13}C NMR (151 MHz, $CDCl_3$) δ 173.16, 138.68, 135.97, 130.29, 128.47, 126.39, 126.12, 70.65, 34.64, 28.40, 27.71, 19.26, 19.08.

HRMS m/z (ESI) calcd. for $C_{14}H_{21}O_2^+$ $(M+H)^+$ 221.1536, found 221.1537.

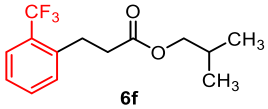

6f

Isobutyl 3-(2-(trifluoromethyl)phenyl)propanoate (6f)

6f was prepared according to the general procedure for the cross-coupling reaction. The product was isolated as a colorless oil. Yield: 79%.

^{1}H NMR (600 MHz, $CDCl_3$) δ 7.63 (d, *J* = 7.8 Hz, 1H), 7.47 (t, *J* = 7.7 Hz, 1H), 7.36 (d, *J* = 7.8 Hz, 1H), 7.31 (t, *J* = 7.7 Hz, 1H), 3.88 (d, *J* = 6.7 Hz, 2H), 3.14 (t, *J* = 8.0 Hz, 2H), 2.67 – 2.61 (m, 2H), 1.91 (dp, *J* = 13.4, 6.8 Hz, 1H), 0.91 (d, *J* = 6.7 Hz, 6H).

^{13}C NMR (126 MHz, $CDCl_3$) δ 172.52, 139.22, 131.84, 130.80, 128.49 (q, *J* = 30.1 Hz), 126.36, 126.02 (q, *J* = 5.8 Hz), 125.45 (q, *J* = 214.2 Hz), 70.65, 35.72, 27.76, 27.59, 18.95.

^{19}F NMR (471 MHz, $CDCl_3$) δ -59.84.

HRMS m/z (ESI) calcd. for $C_{14}H_{18}F_3O_2^+$ $(M+H)^+$ 275.1253, found 275.1270.

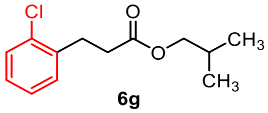

6g

Isobutyl 3-(2-chlorophenyl)propanoate (6g)

6g was prepared according to the general procedure for the cross-coupling reaction. The product was isolated as a colorless oil. Yield: 62%.

^{1}H NMR (600 MHz, $CDCl_3$) δ 7.34 (dd, *J* = 7.5, 1.7 Hz, 1H), 7.25 (dd, *J* = 7.2, 2.0 Hz, 1H), 7.17 (dtd, *J* = 17.0, 7.4, 1.7 Hz, 2H), 3.86 (d, *J* = 6.6 Hz, 2H), 3.10 – 3.04 (m, 2H), 2.69 – 2.63 (m, 2H), 1.90 (hept, *J* = 6.7 Hz, 1H), 0.90 (d, *J* = 6.7 Hz, 6H).

^{13}C NMR (151 MHz, $CDCl_3$) δ 172.82, 138.12, 133.97, 130.43, 129.57, 127.82, 126.90, 70.68, 34.00, 29.02, 27.69, 19.07.

HRMS m/z (ESI) calcd. for $C_{13}H_{17}ClO_2Na^+$ $(M+Na)^+$ 263.0809, found 263.0815.

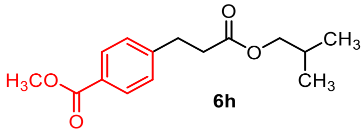

6h

Methyl 4-(3-isobutoxy-3-oxopropyl)benzoate (6h)

6h was prepared according to the general procedure for the cross-coupling reaction. The product was isolated as a white solid. Yield: 58%.
^{1}H NMR (600 MHz, $CDCl_3$) δ 7.95 (d, *J* = 8.4 Hz, 2H), 7.27 (d, *J* = 8.5 Hz, 2H), 3.90 (s, 3H), 3.84 (d, *J* = 6.7 Hz, 2H), 3.01 (t, *J* = 7.7 Hz, 2H), 2.66 (t, *J* = 7.7 Hz, 2H), 1.89 (hept, *J* = 6.7 Hz, 1H), 0.89 (d, *J* = 6.7 Hz, 6H).

^{13}C NMR (151 MHz, $CDCl_3$) δ 172.62, 167.03, 145.99, 129.85, 128.35, 128.28, 70.74, 52.04, 35.37, 30.95, 27.68, 19.04.

HRMS m/z (ESI) calcd. for $C_{15}H_{21}O_4^+$ $(M+H)^+$ 265.1434, found 265.1435.

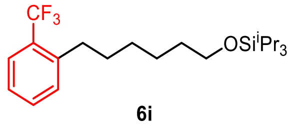

6i

Triisopropyl((6-(2-(trifluoromethyl)phenyl)hexyl)oxy)silane (6i)
6i was prepared according to the general procedure for the cross-coupling reaction. The product was isolated as a pale yellow oil. Yield: 63%.
^{1}H NMR (500 MHz, $CDCl_3$) δ 7.60 (d, *J* = 7.9 Hz, 1H), 7.45 (t, *J* = 7.1 Hz, 1H), 7.32 (d, *J* = 7.7 Hz, 1H), 7.27 (d, *J* = 8.5 Hz, 1H), 3.68 (t, *J* = 6.6 Hz, 2H), 2.76 (t, *J* = 8.1 Hz, 2H), 1.63 (p, *J* = 7.6 Hz, 2H), 1.58 – 1.52 (m, 2H), 1.41 (tt, *J* = 6.9, 2.6 Hz, 4H), 1.06 (d, *J* = 4.5 Hz, 21H).

^{13}C NMR (126 MHz, $CDCl_3$) δ 141.67, 131.49, 130.84, 128.25 (q, *J* = 29.5 Hz), 125.74 (q, *J* = 5.8 Hz), 125.56, 123.50, 63.30, 32.83, 32.53, 31.69, 29.43, 25.56, 17.95, 11.94.

^{19}F NMR (471 MHz, $CDCl_3$) δ -59.66.

GCMS m/z (EI, 70 eV): 359.2 $(C_{19}H_{30}F_3OSi)^+$ $[M-iPr]^+$

VI. NMR Spectral Data

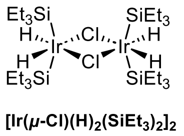

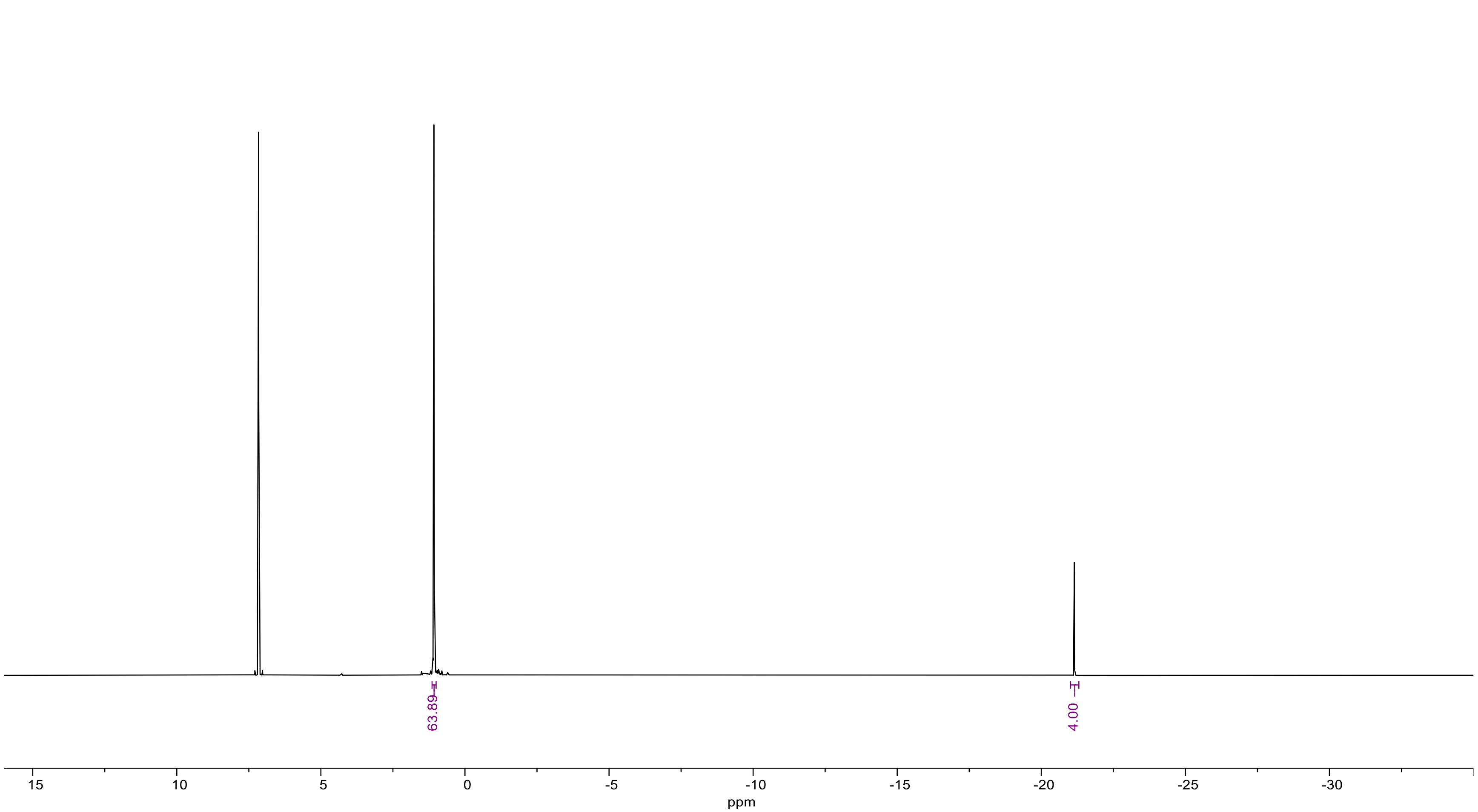

Figure S5. ^{1}H NMR Spectrum of **[Ir(*μ*-Cl)(H)$_2$(SiEt$_3$)$_2$]$_2$** (600 MHz, C_6D_6).

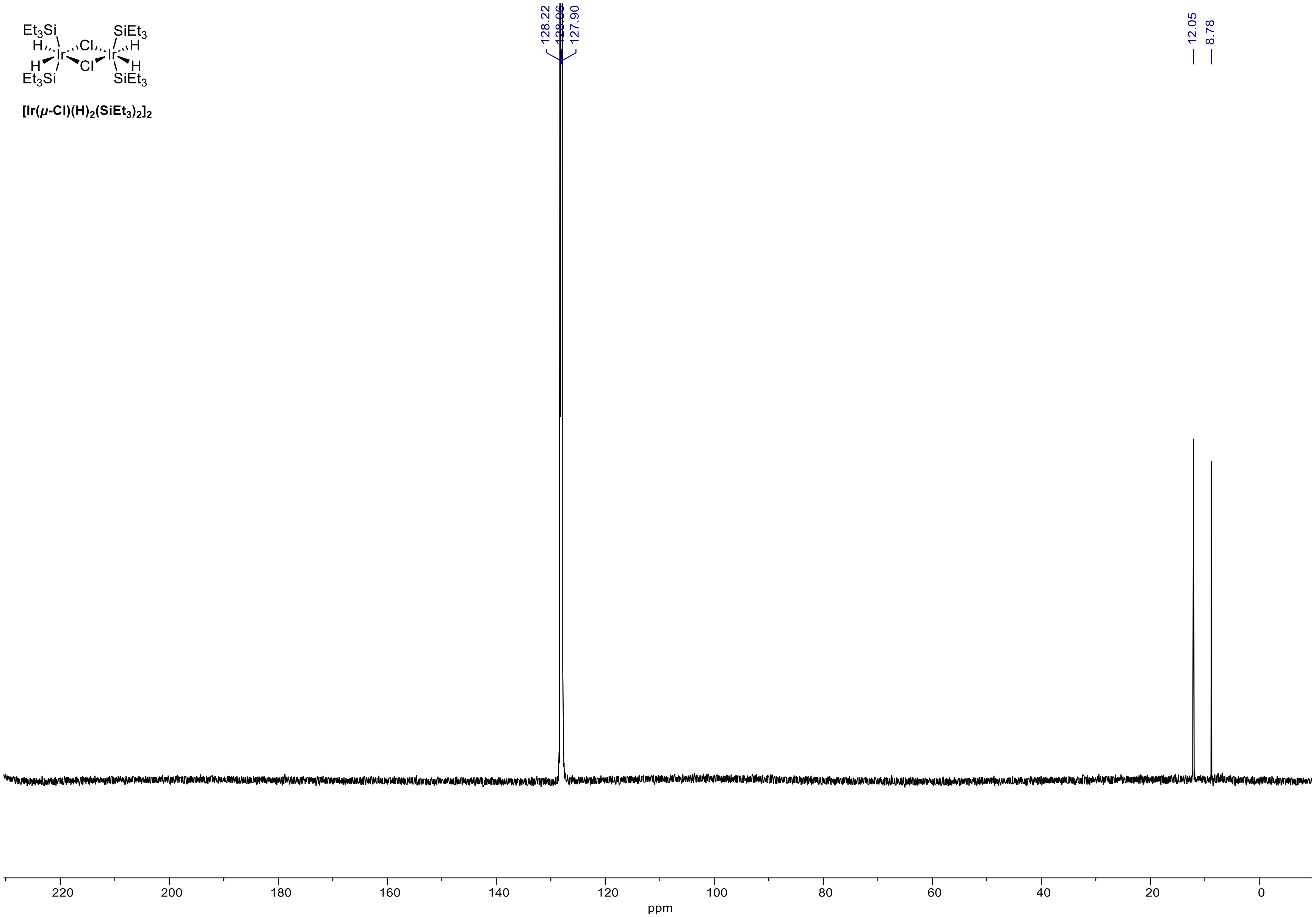

Figure S6. $^{13}C\{^{1}H\}$ NMR Spectrum of **[Ir(*μ*-Cl)(H)$_2$(SiEt$_3$)$_2$]$_2$** (150 MHz, C_6D_6).

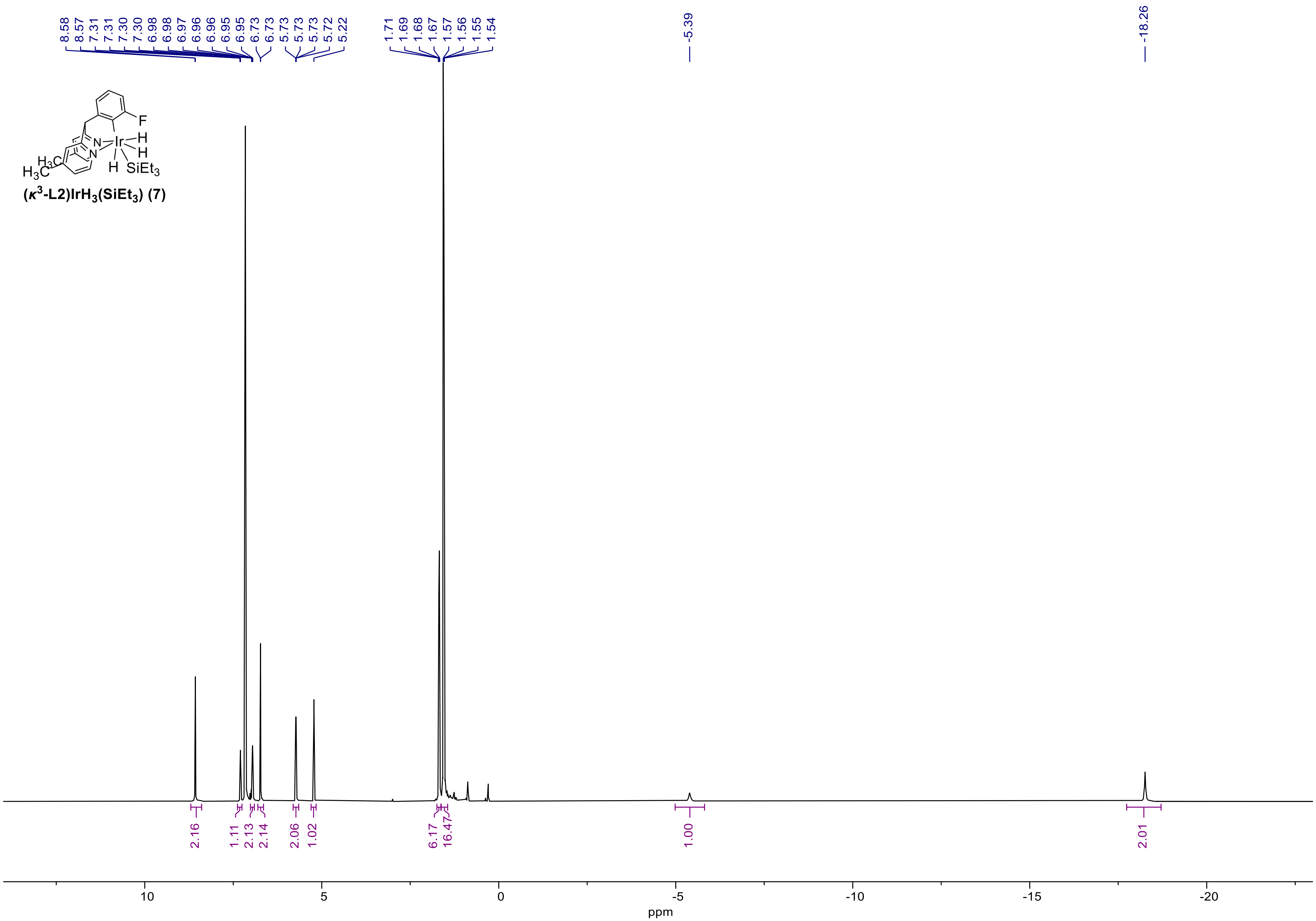

Figure S7. ^{1}H NMR Spectrum of **(κ[3]-L2)IrH$_3$(SiEt$_3$)** (**7**) (600 MHz, C_6D_6).

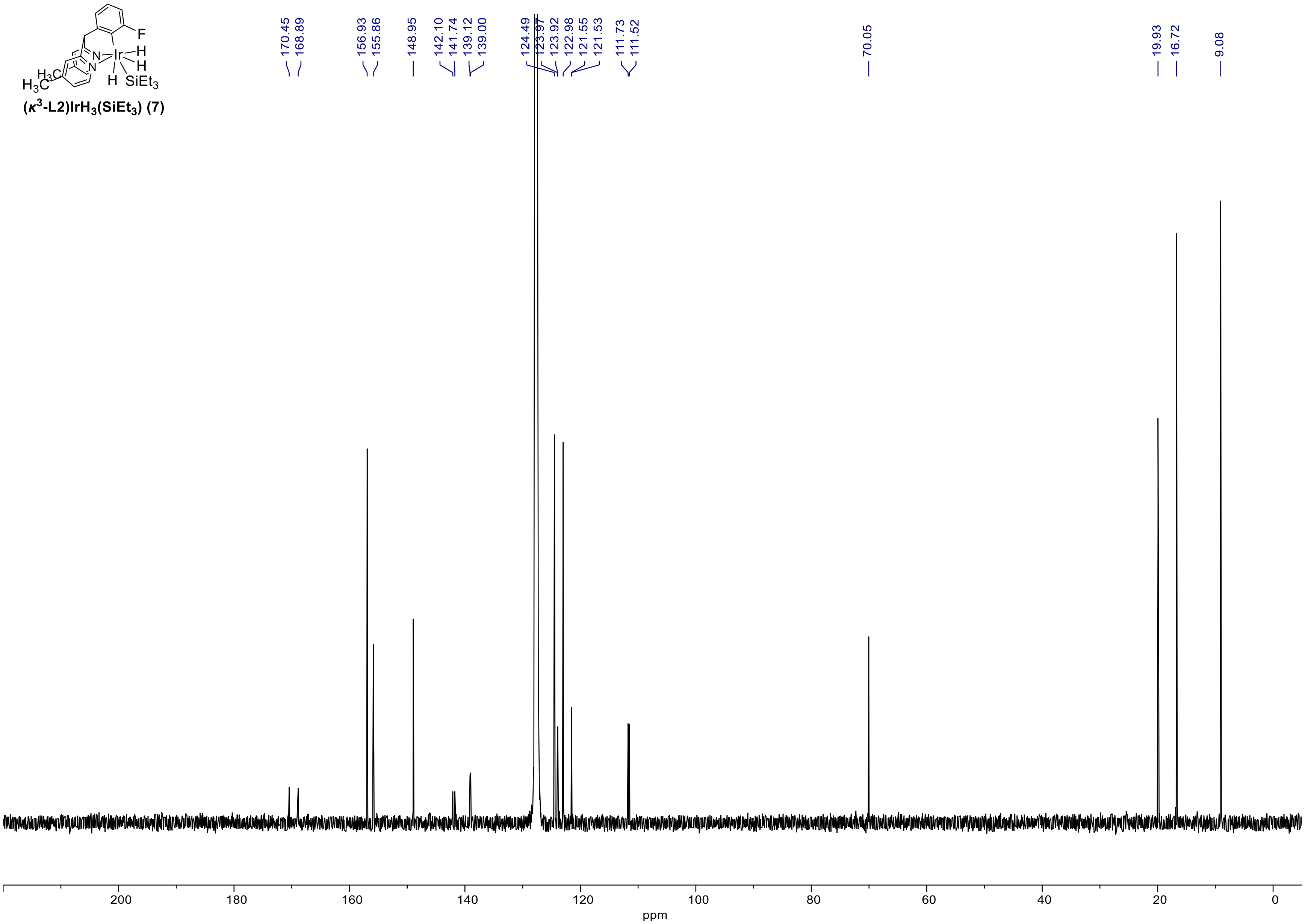

Figure S8. $^{13}C\{^1H\}$ NMR Spectrum of **(κ^3-L2)IrH$_3$(SiEt$_3$)** (7) (150 MHz, C_6D_6).

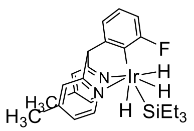

(κ^3-L2)IrH$_3$(SiEt$_3$) (7)

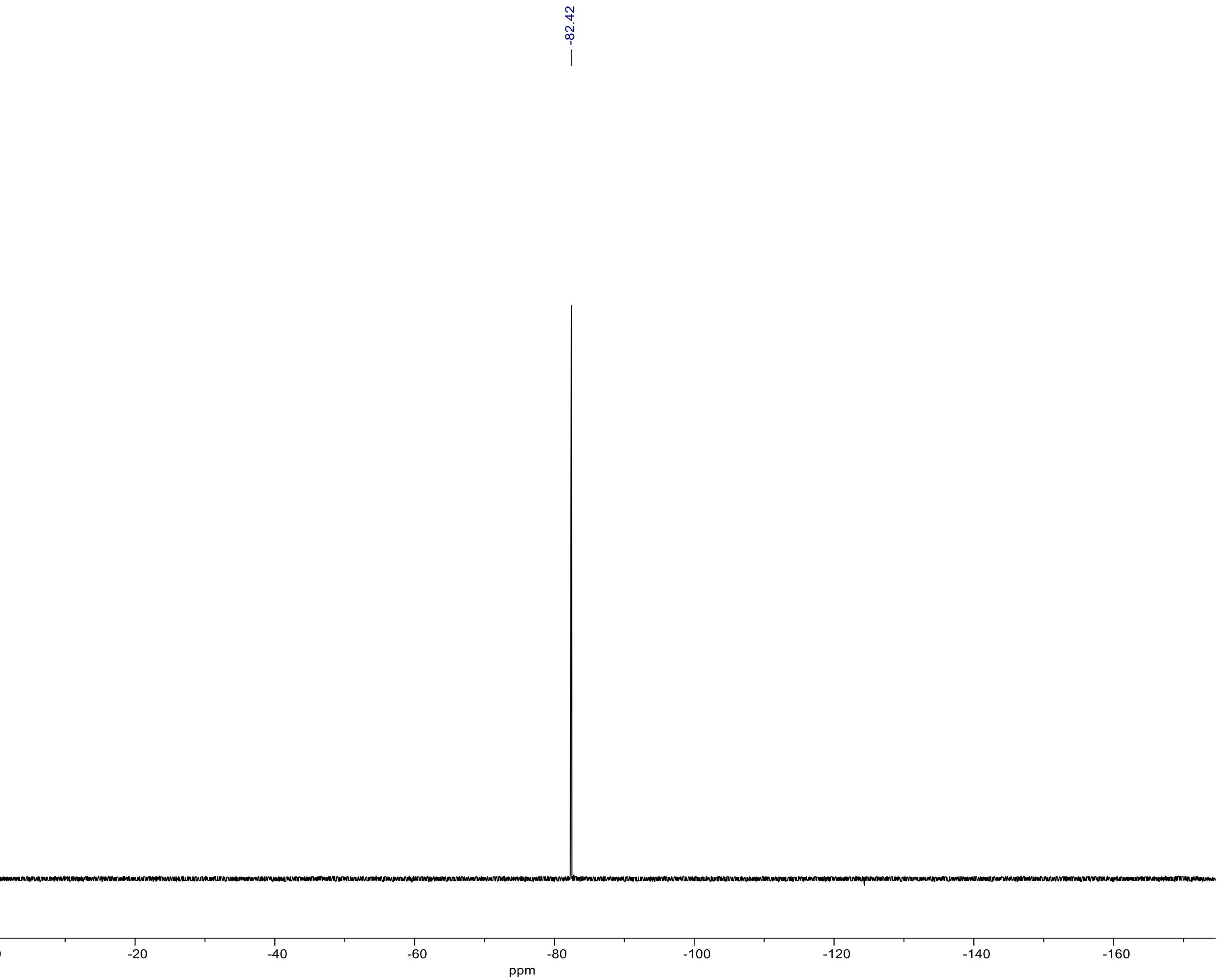

Figure S9. ^{19}F NMR Spectrum of **(κ^3-L2)IrH$_3$(SiEt$_3$)** (**7**) (471 MHz, C_6D_6).

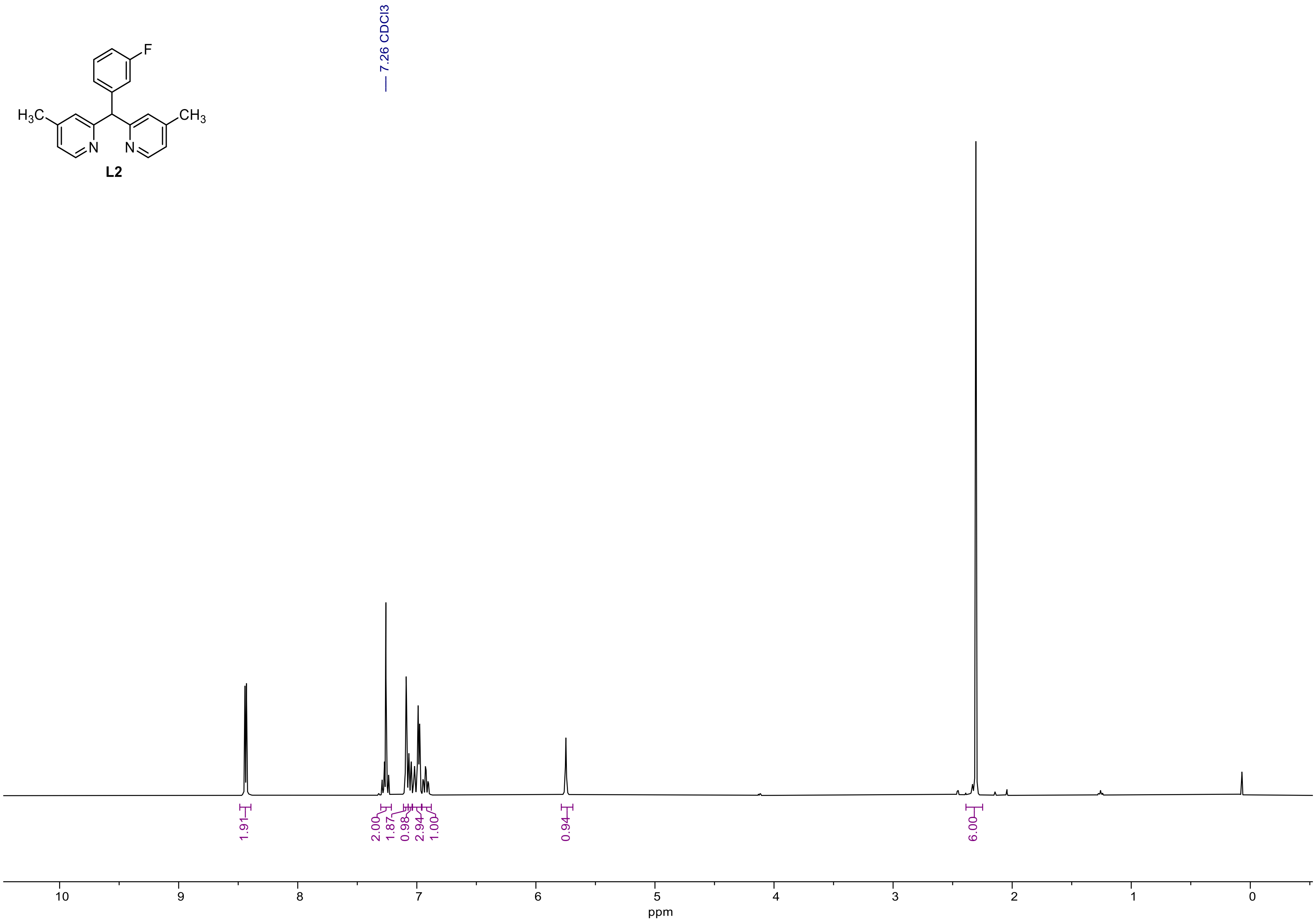

Figure S10. ^{1}H NMR Spectrum of **L2** (400 MHz, $CDCl_3$).

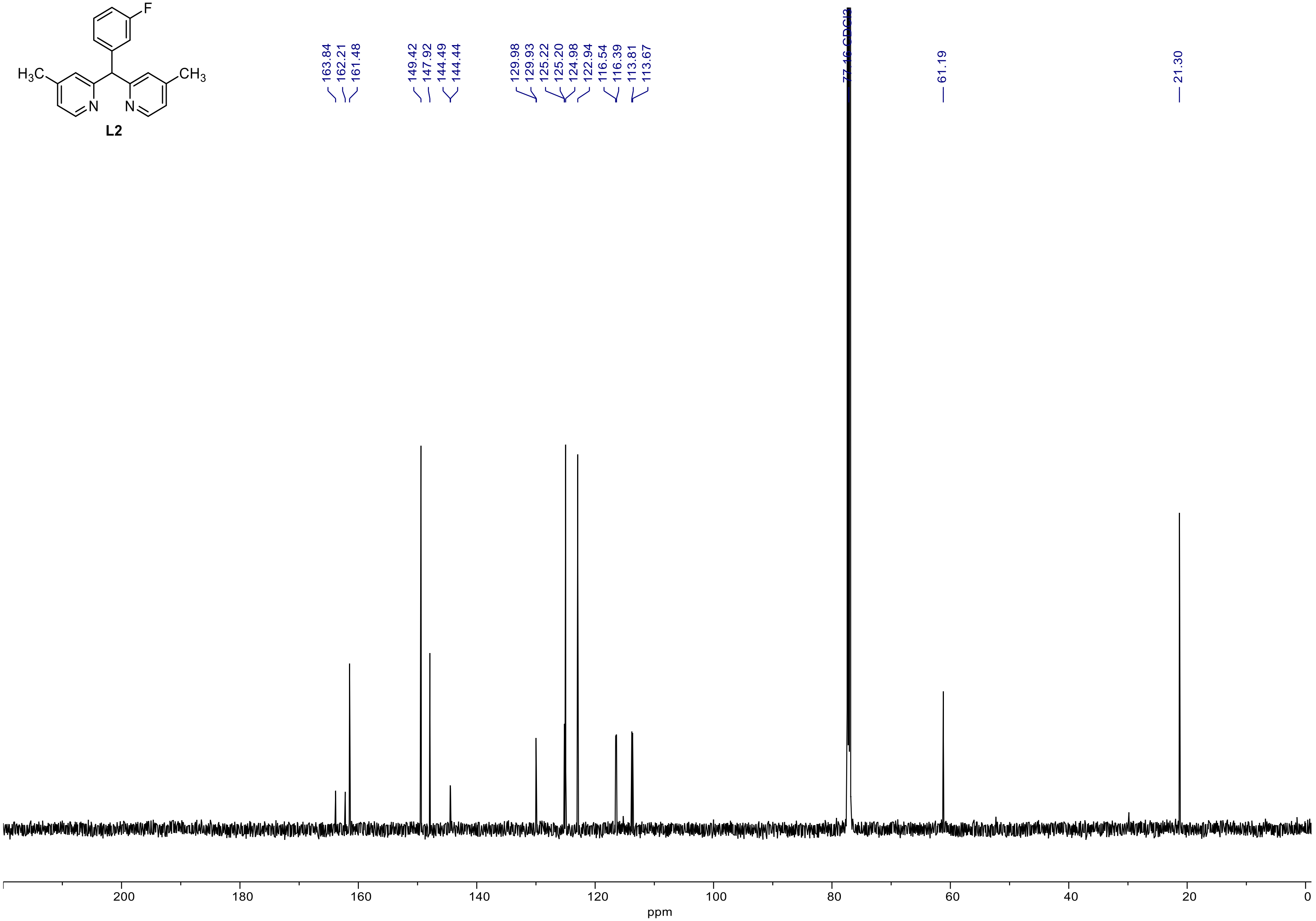

Figure S11. $^{13}C\{^{1}H\}$ NMR Spectrum of **L2** (150 MHz, $CDCl_3$).

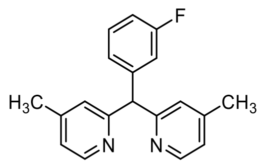

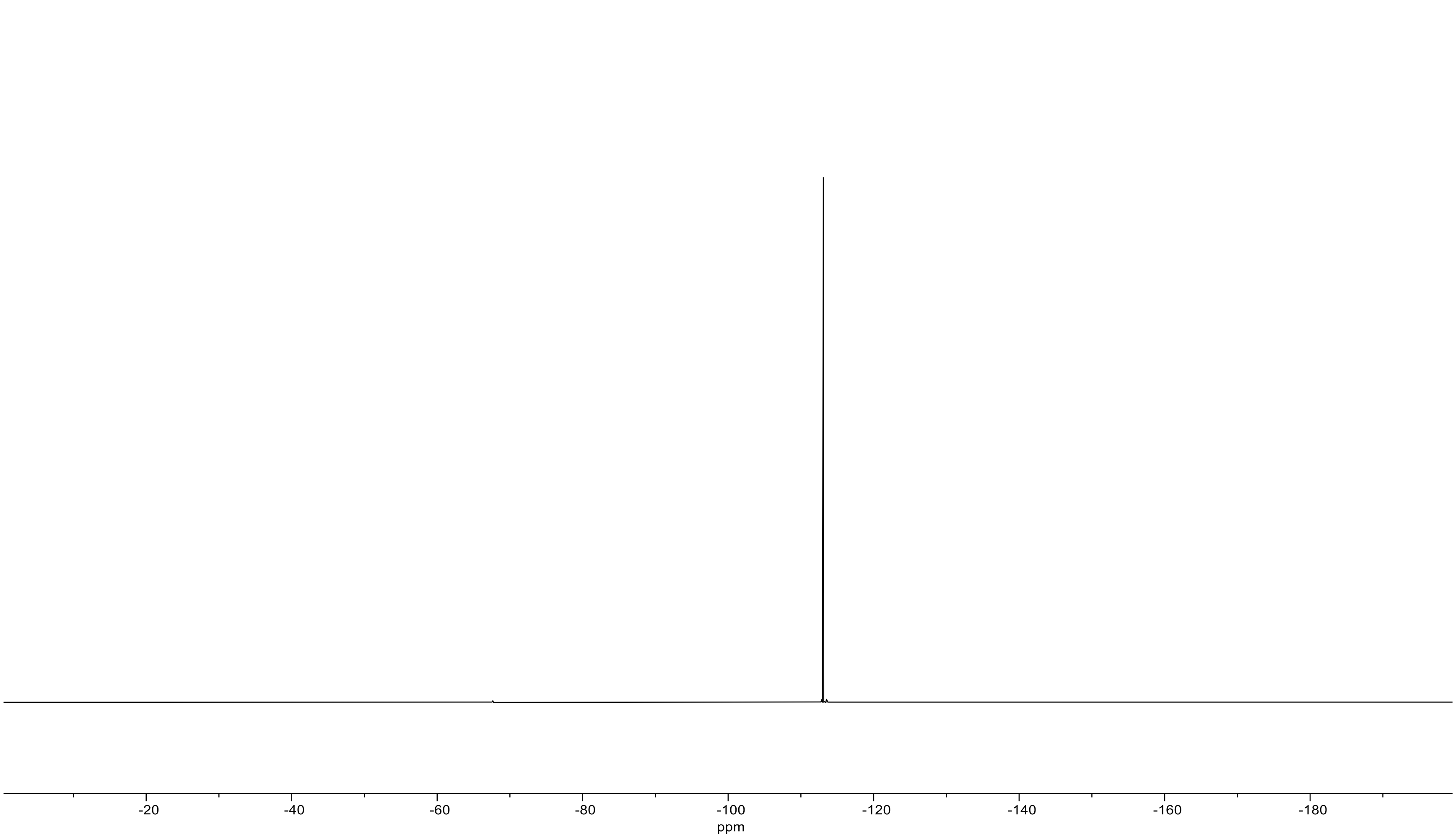

Figure S12. ^{19}F NMR Spectrum of **L2** (376 MHz, $CDCl_3$).

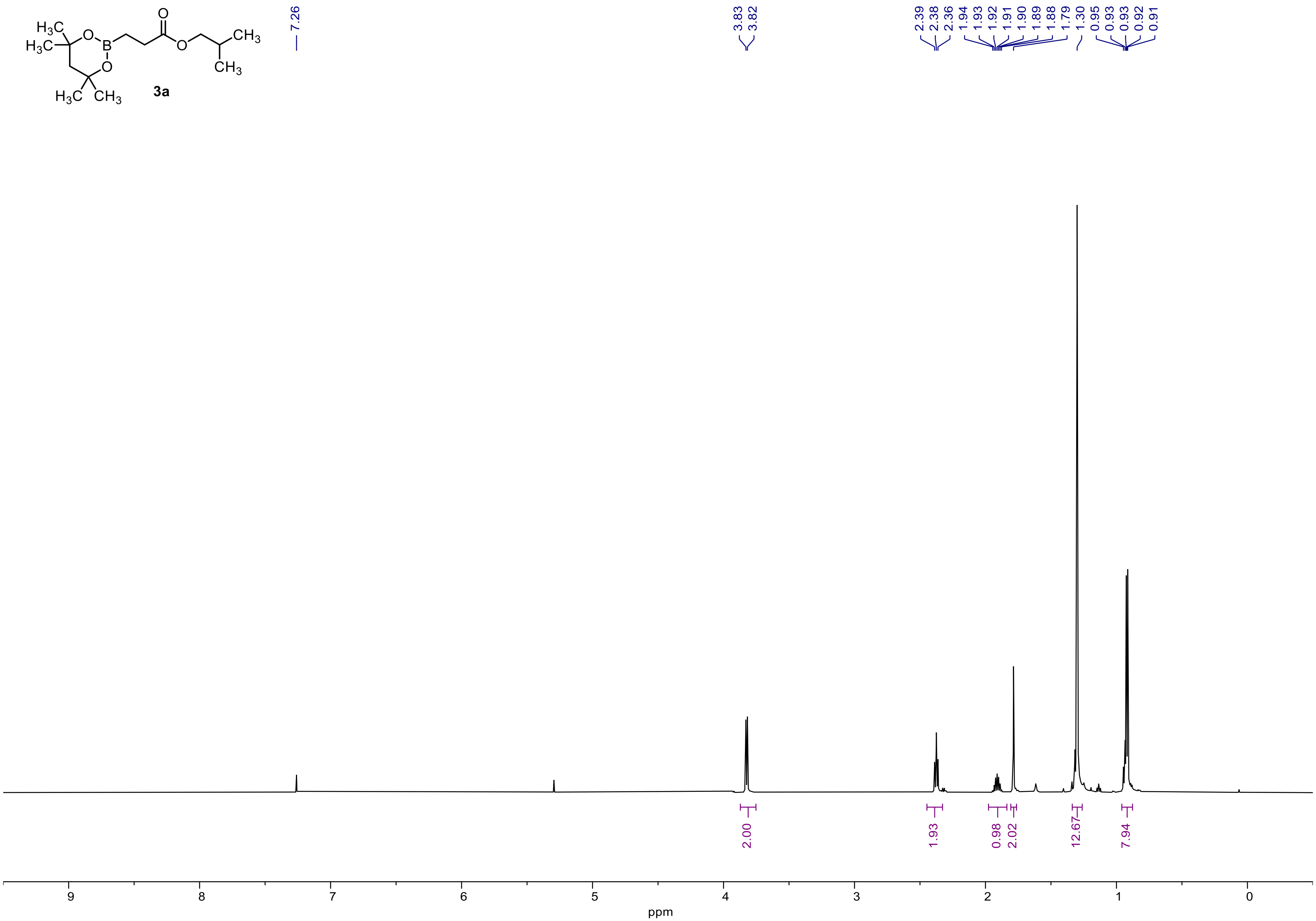

Figure S13. ¹H NMR Spectrum of **3a** (600 MHz, $CDCl_3$).

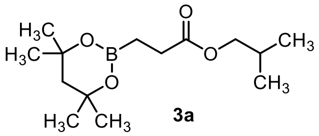

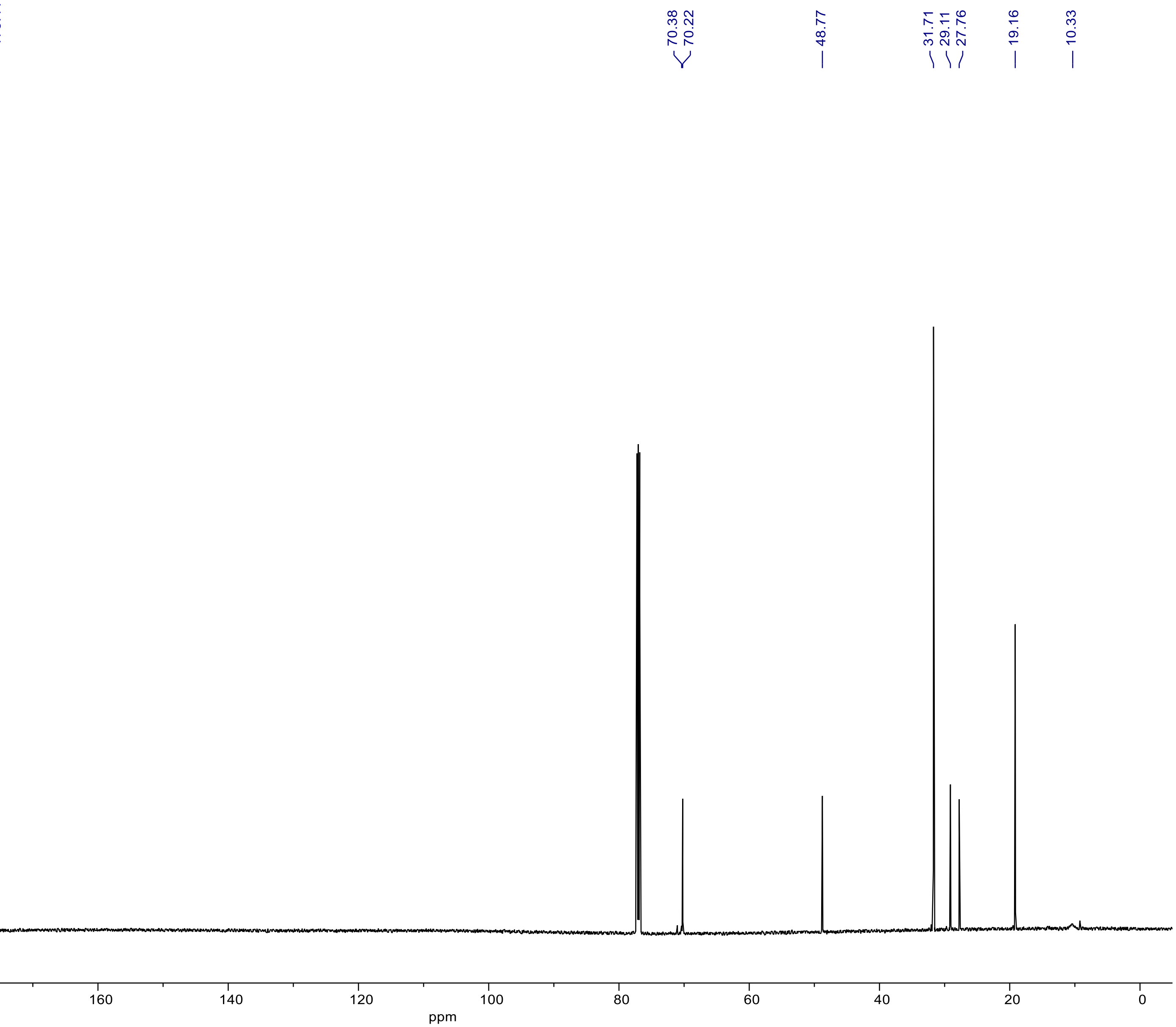

Figure S14. $^{13}C\{^{1}H\}$ NMR Spectrum of **3a** (150 MHz, $CDCl_3$).

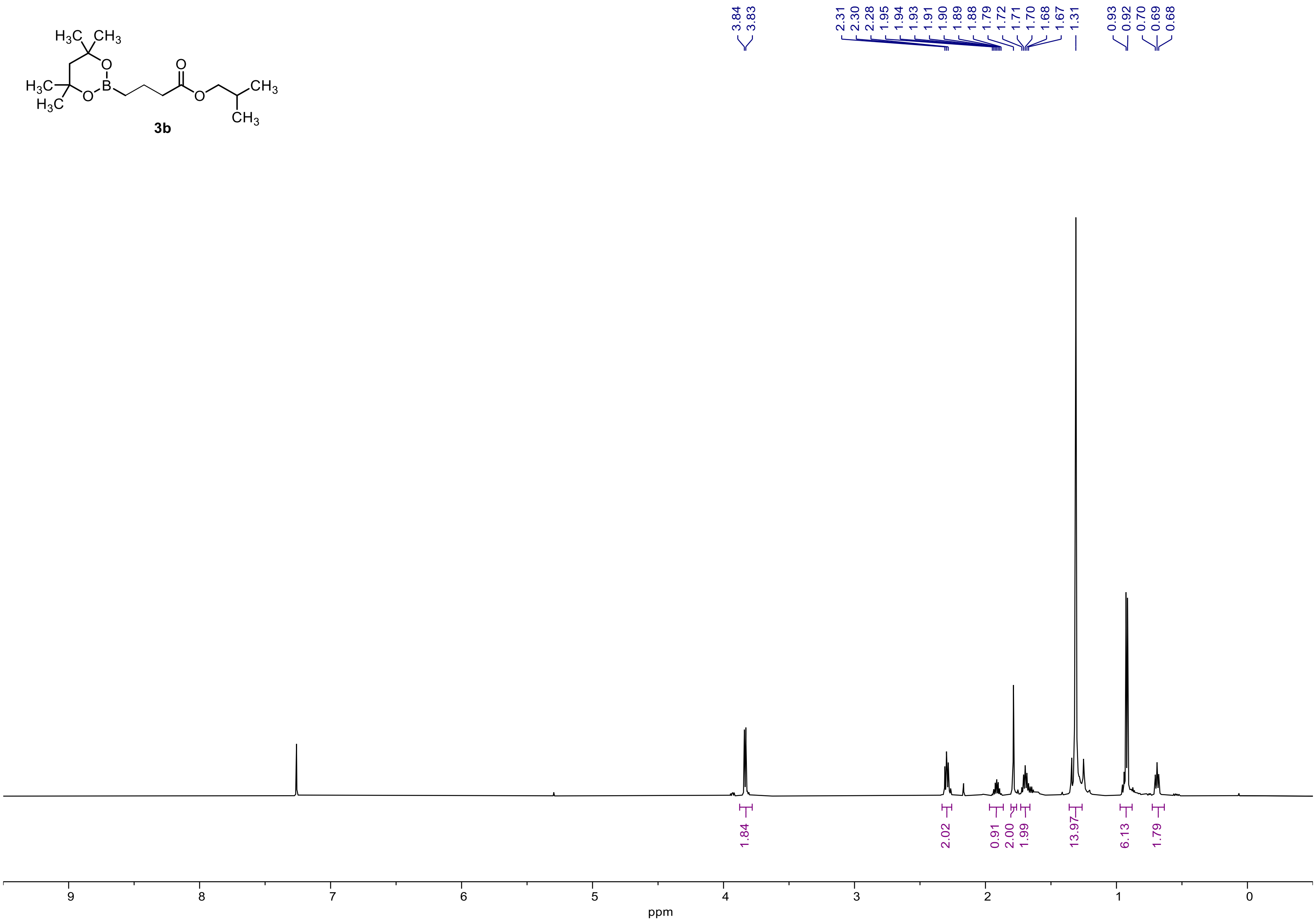

Figure S15. ^{1}H NMR Spectrum of **3b** (600 MHz, $CDCl_3$).

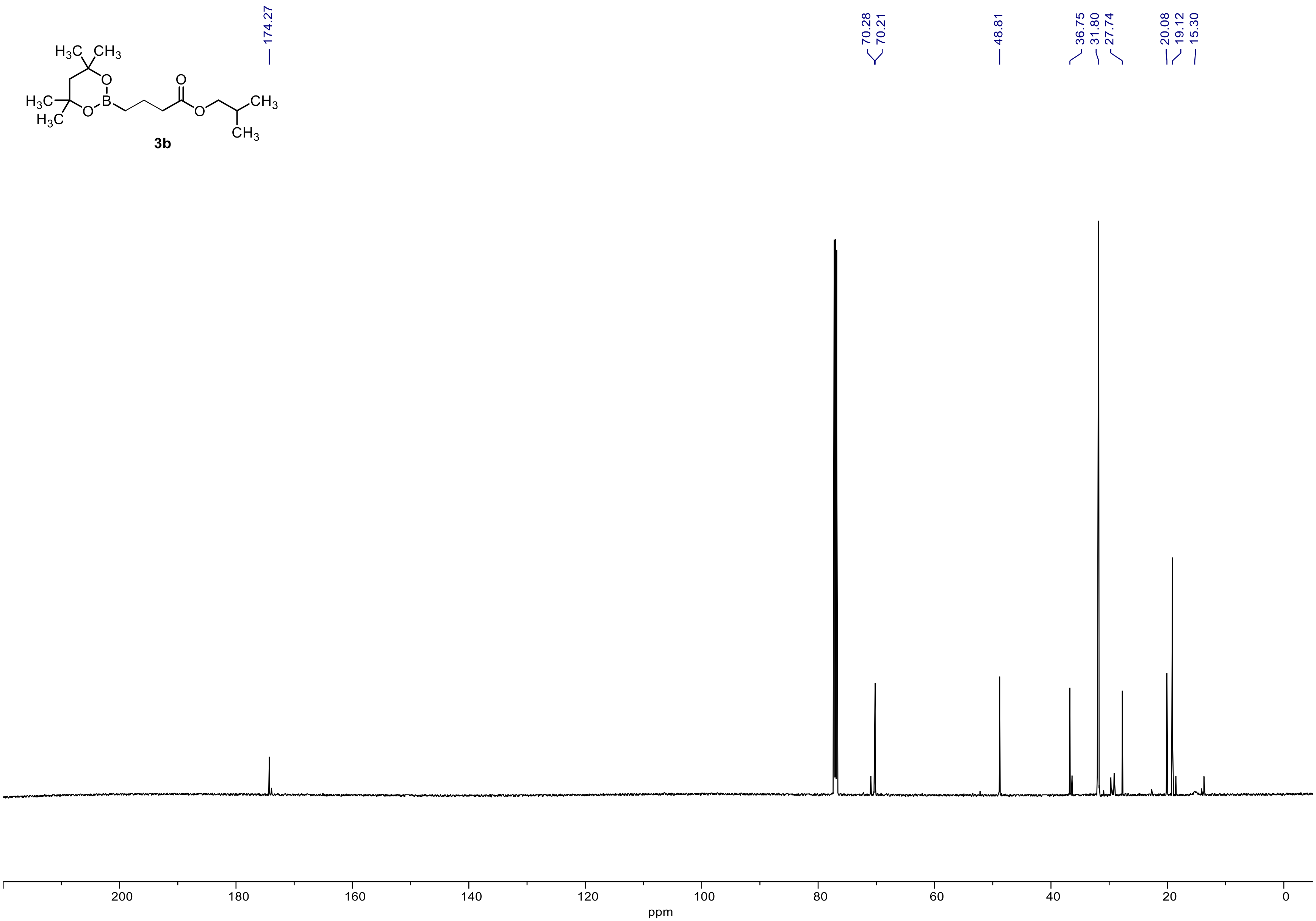

Figure S16. $^{13}C\{^{1}H\}$ NMR Spectrum of **3b** (150 MHz, $CDCl_3$).

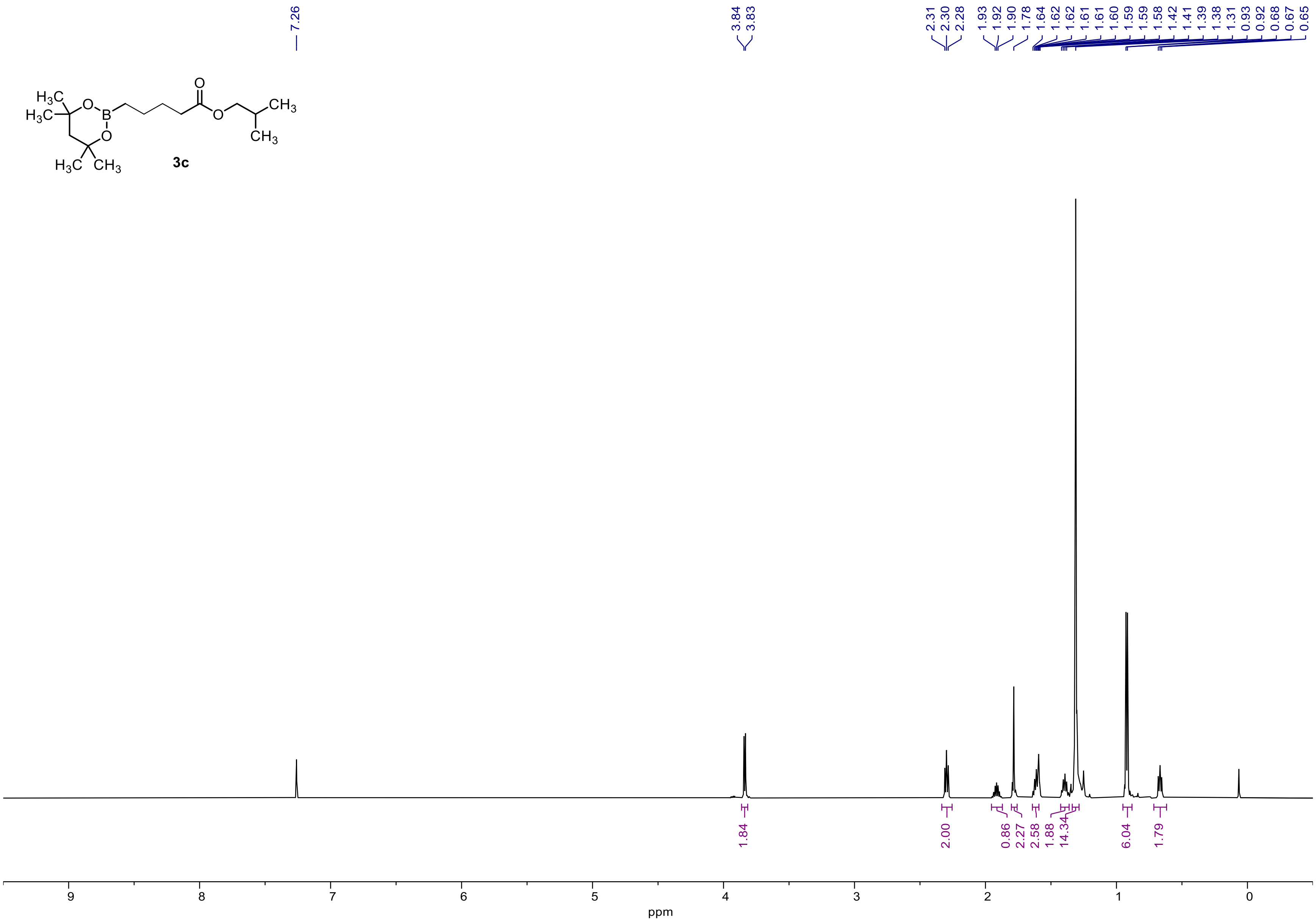

Figure S17. ^{1}H NMR Spectrum of **3c** (600 MHz, $CDCl_3$).

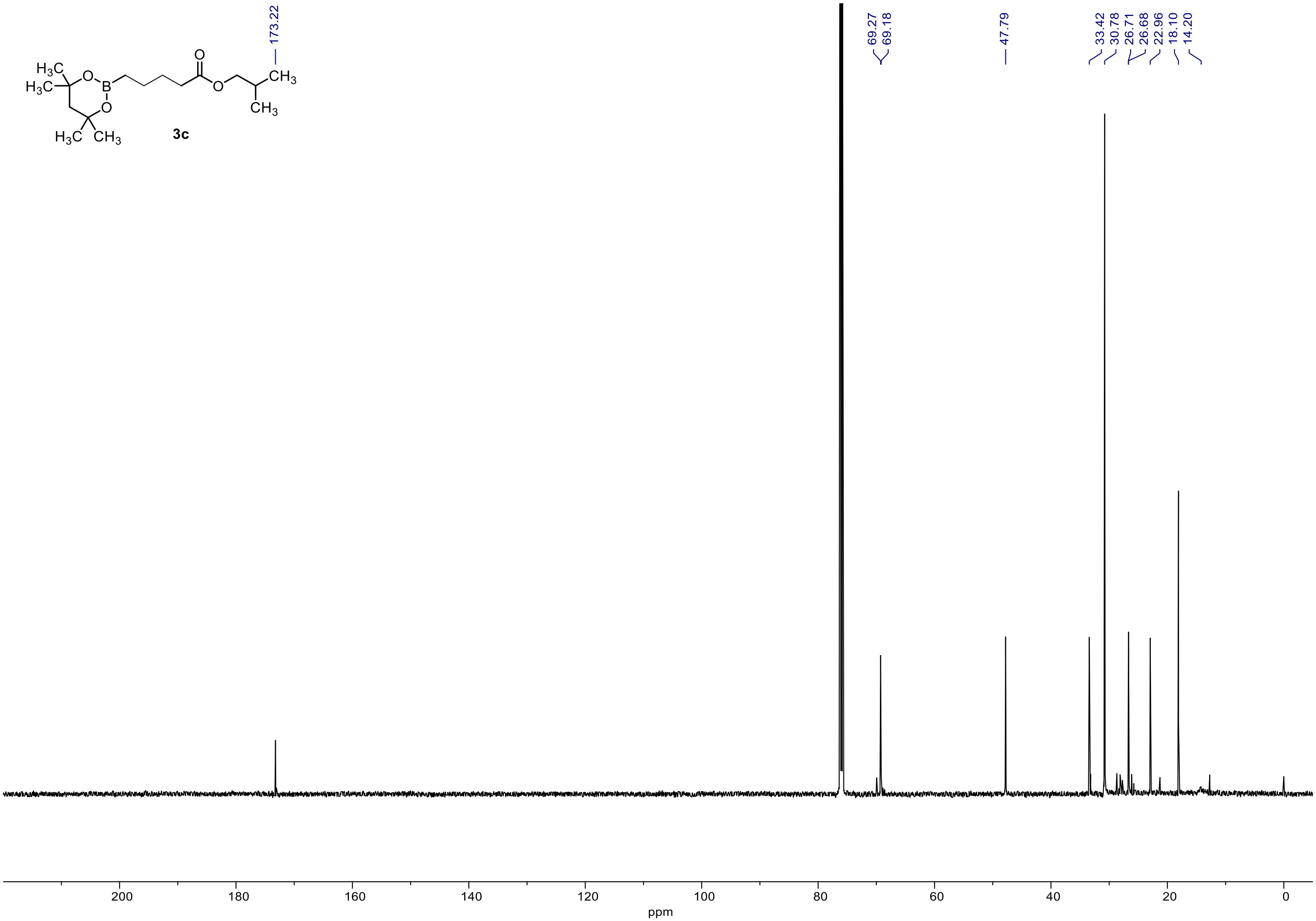

Figure S18. $^{13}C\{^{1}H\}$ NMR Spectrum of **3c** (150 MHz, $CDCl_3$).

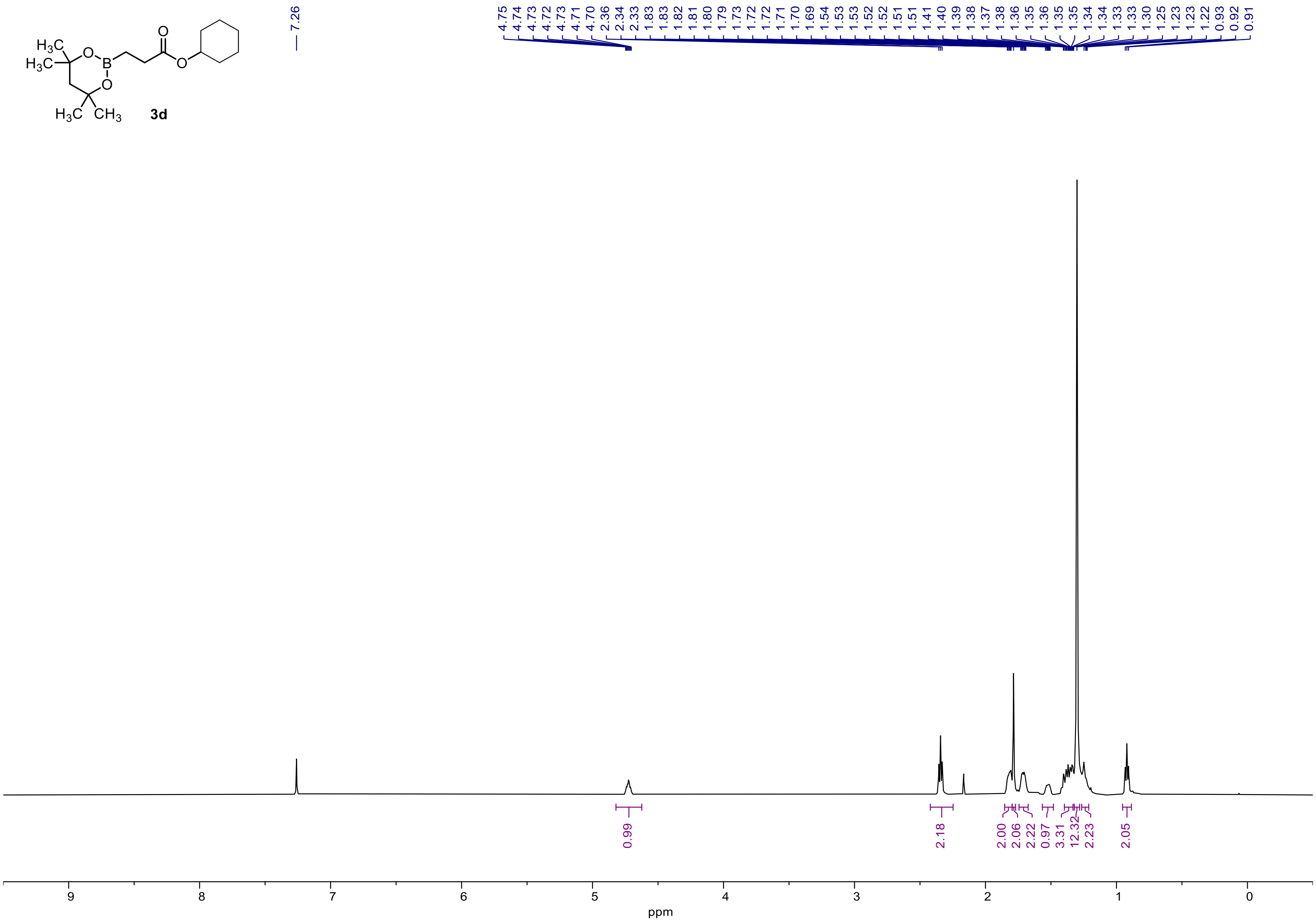

Figure S19. ^{1}H NMR Spectrum of **3d** (600 MHz, $CDCl_3$).

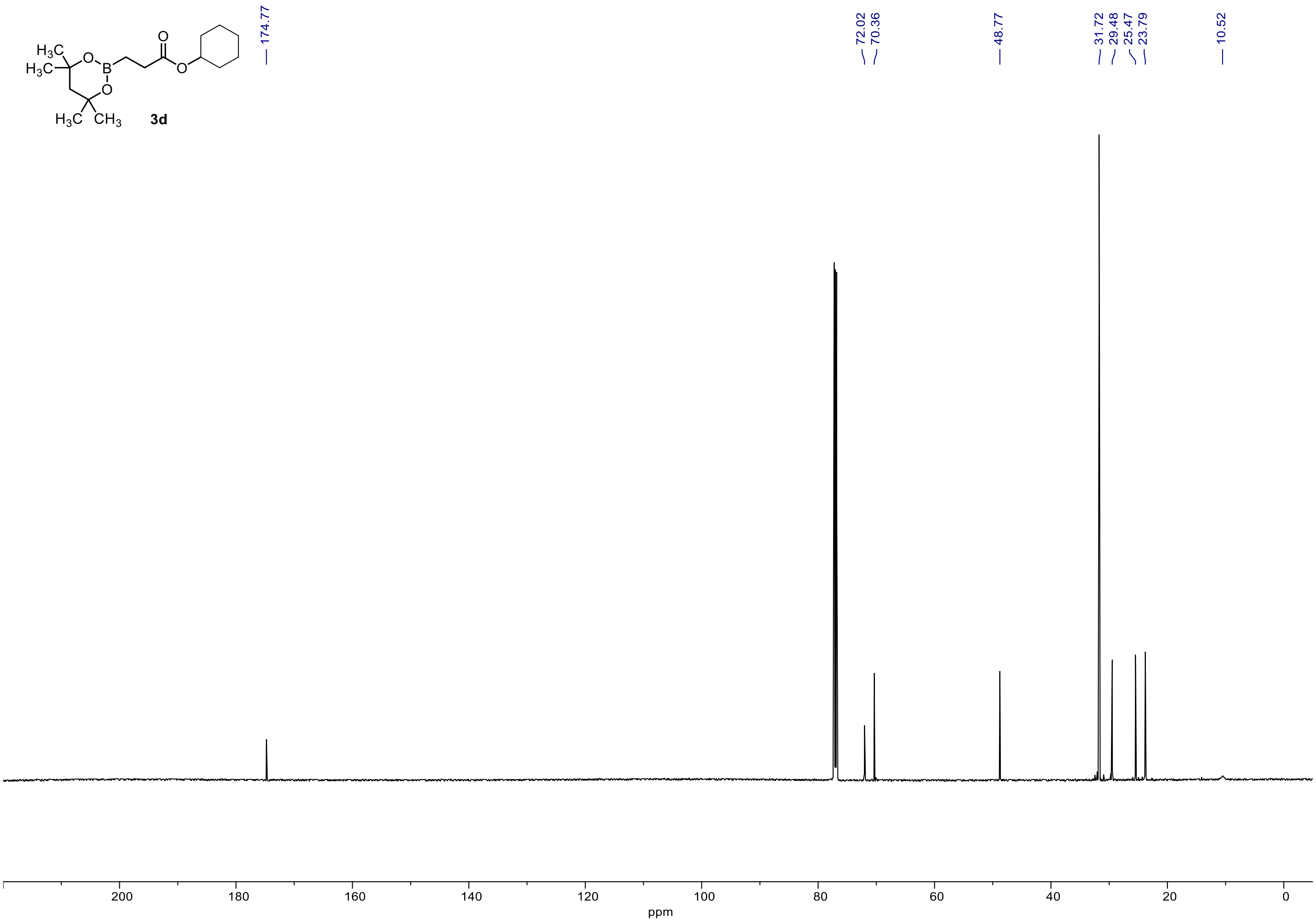

Figure S20. $^{13}C\{^{1}H\}$ NMR Spectrum of **3d** (150 MHz, $CDCl_3$).

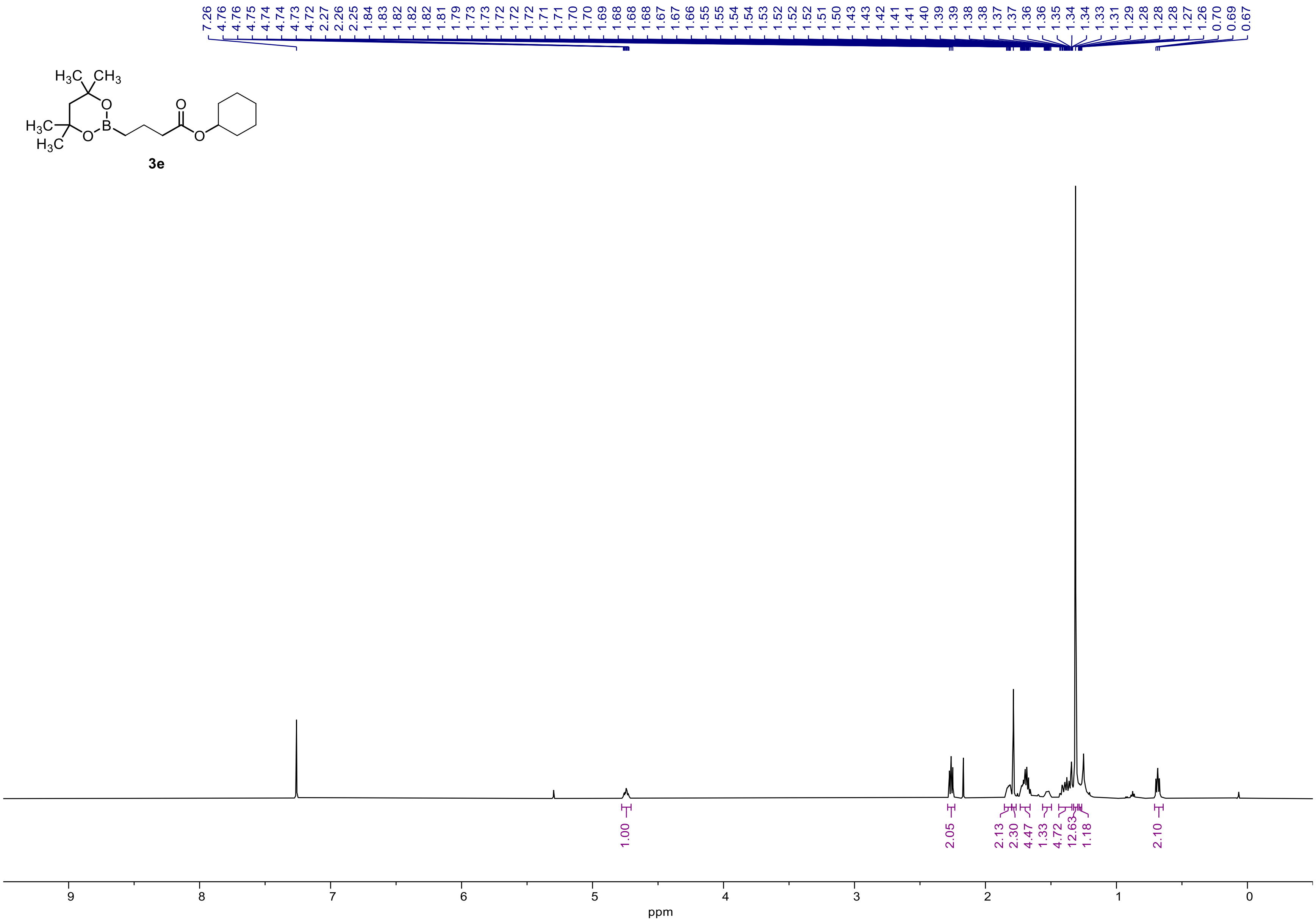

Figure S21. ¹H NMR Spectrum of **3e** (600 MHz, $CDCl_3$).

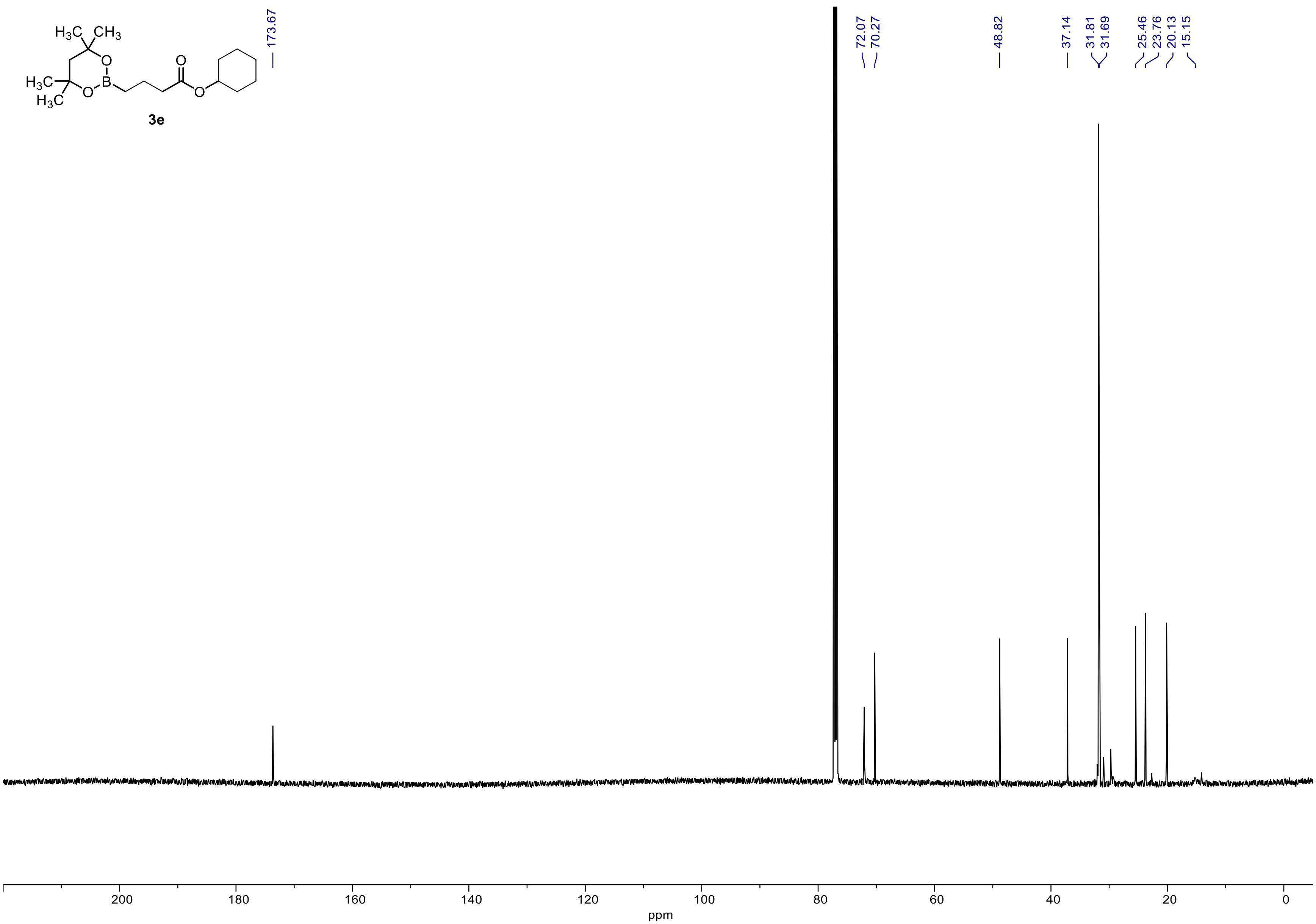

Figure S22. $^{13}C\{^{1}H\}$ NMR Spectrum of **3e** (150 MHz, $CDCl_3$).

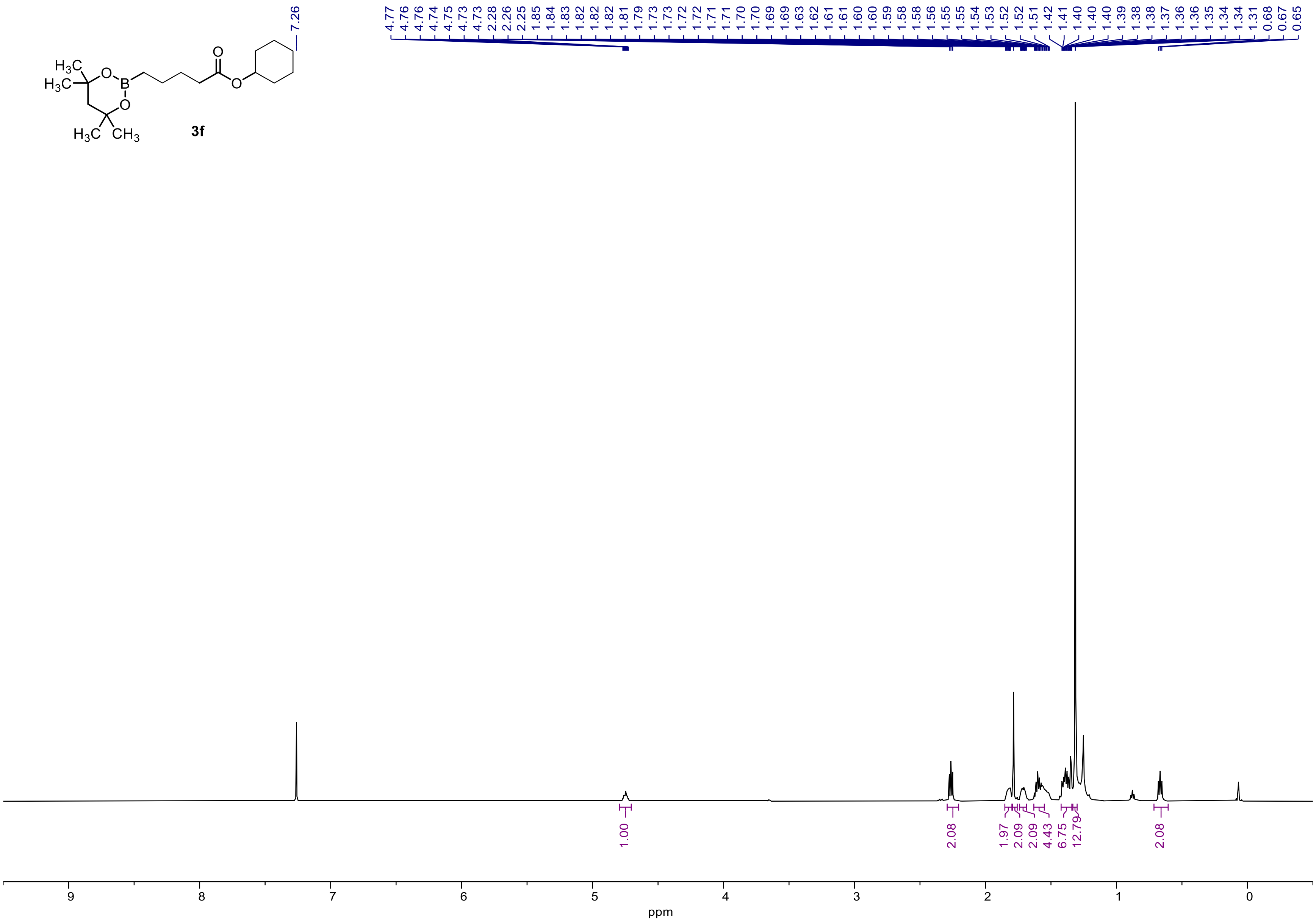

Figure S23. ^{1}H NMR Spectrum of **3f** (600 MHz, $CDCl_3$).

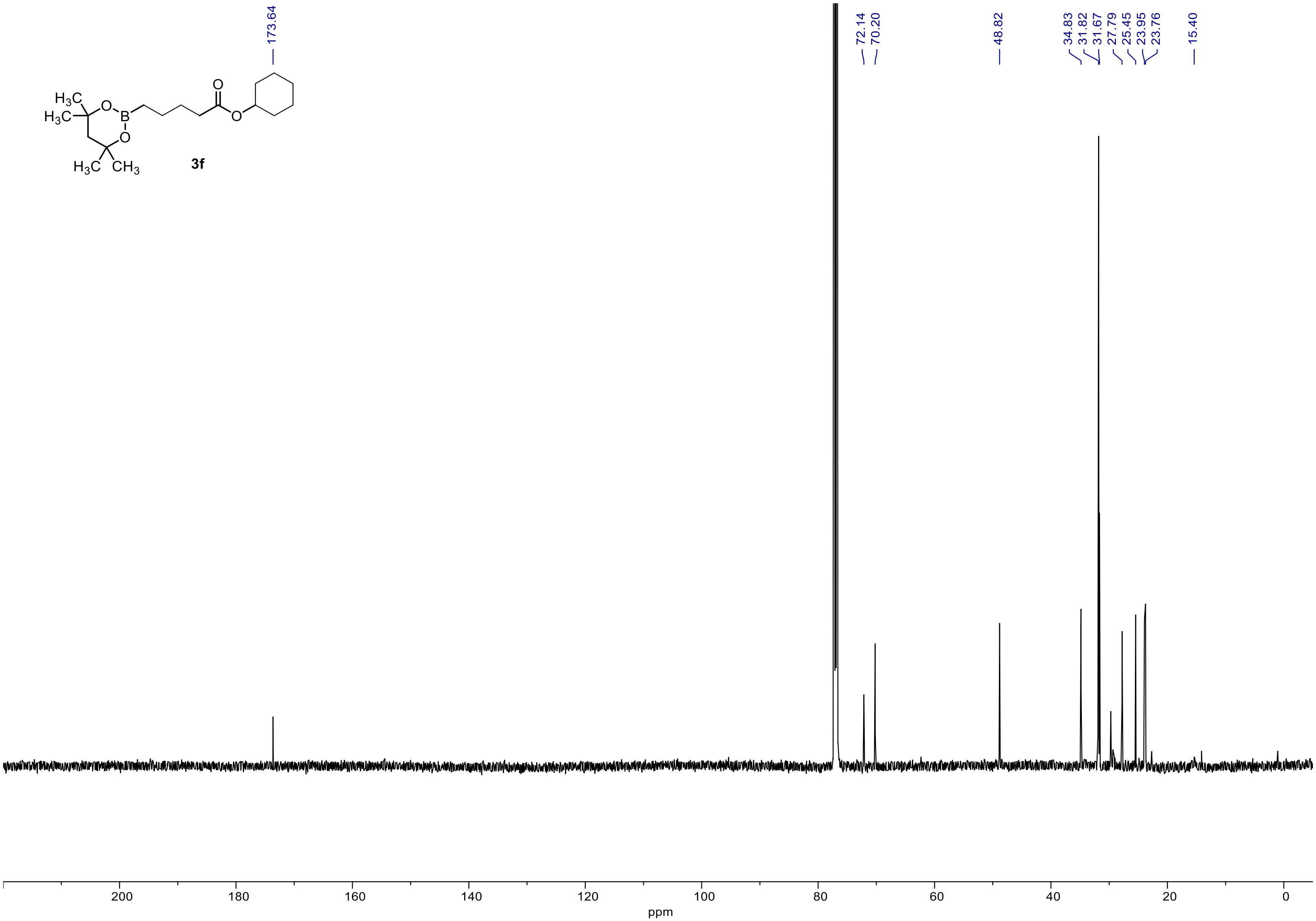

Figure S24. $^{13}C\{^{1}H\}$ NMR Spectrum of **3f** (150 MHz, $CDCl_3$).

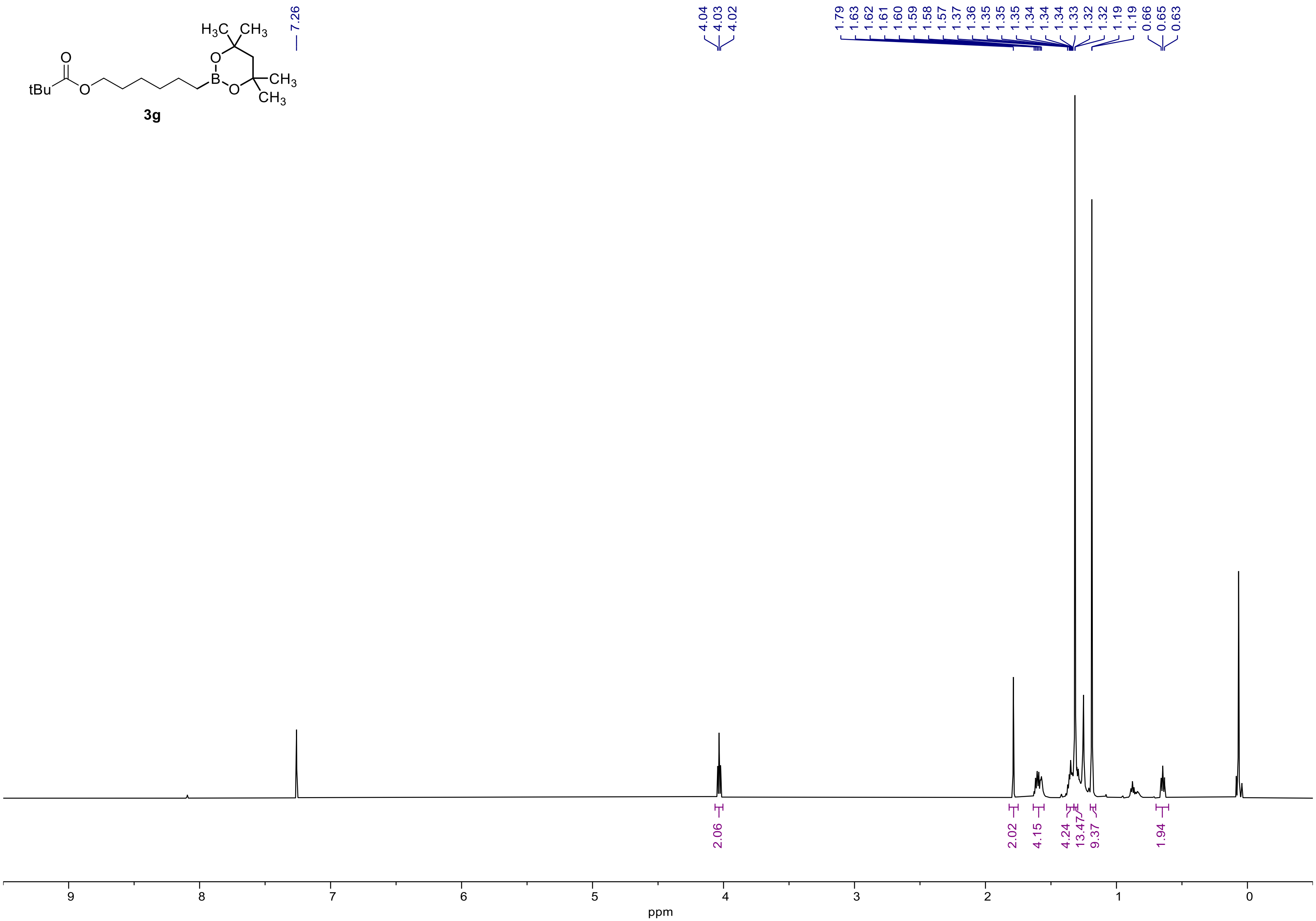

Figure S25. ^{1}H NMR Spectrum of **3g** (600 MHz, $CDCl_3$).

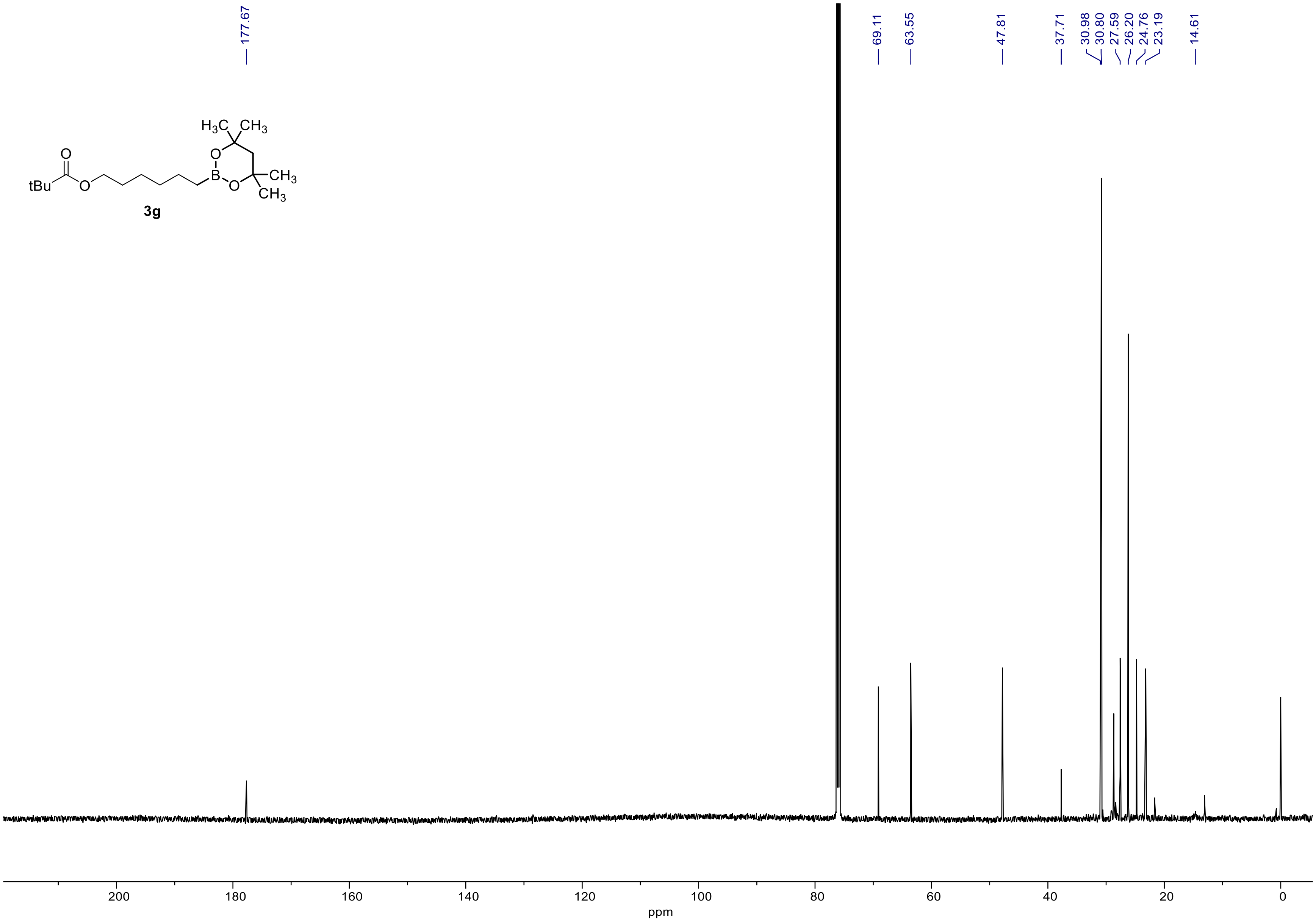

Figure S26. $^{13}C\{^{1}H\}$ NMR Spectrum of **3g** (150 MHz, $CDCl_3$).

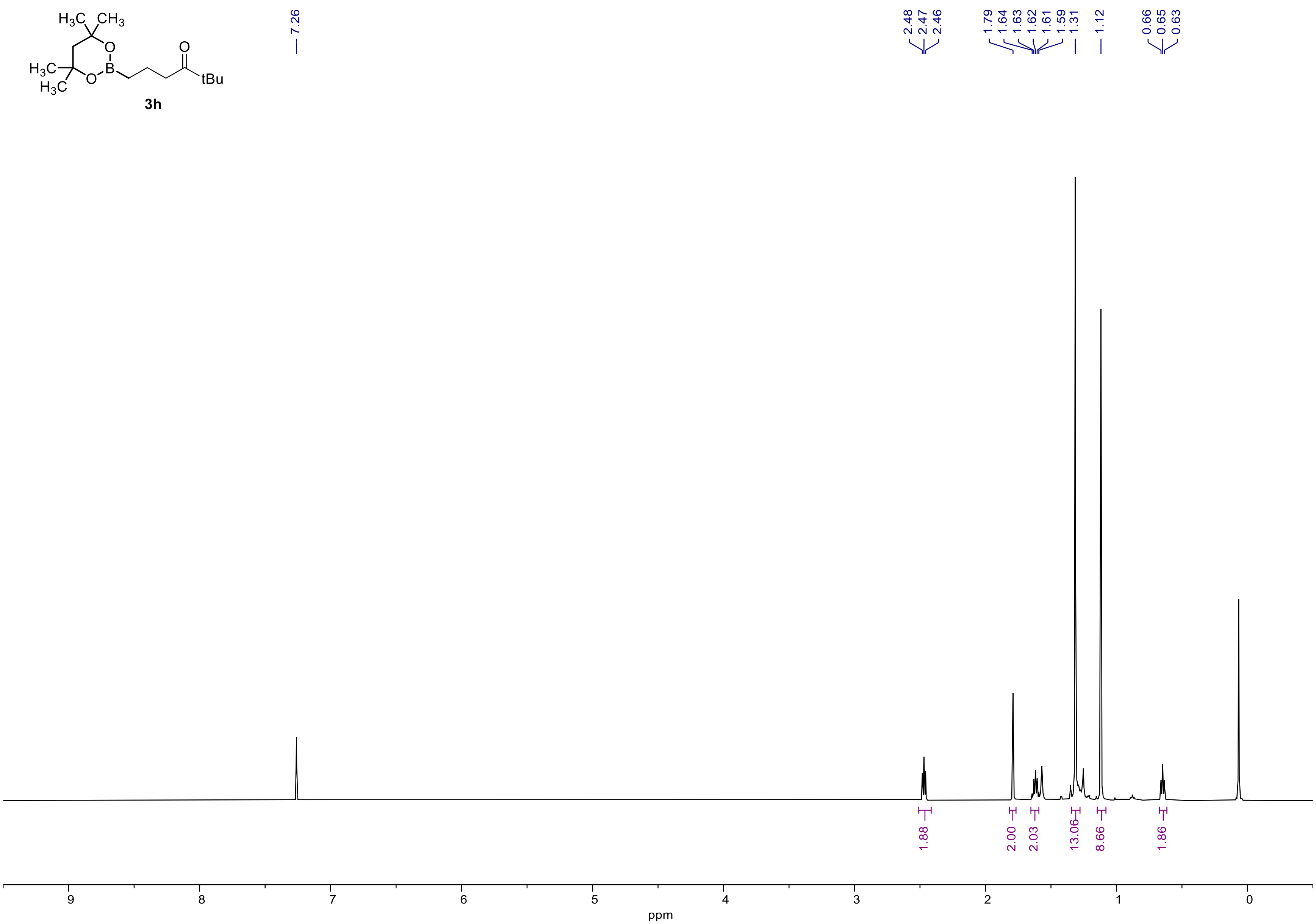

Figure S27. ^{1}H NMR Spectrum of **3h** (600 MHz, $CDCl_3$).

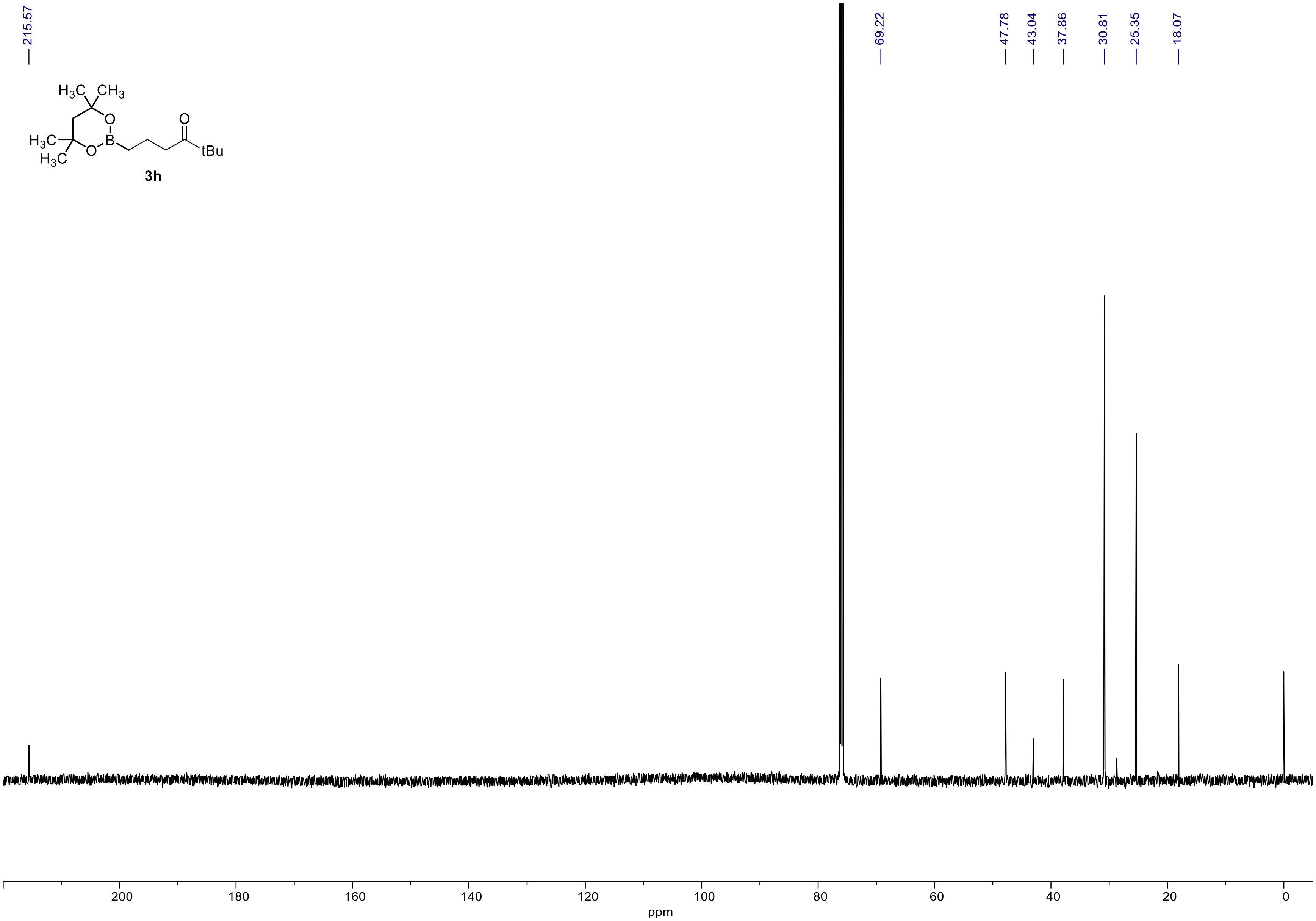

Figure S28. $^{13}C\{^{1}H\}$ NMR Spectrum of **3h** (150 MHz, $CDCl_3$).

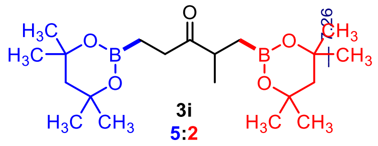

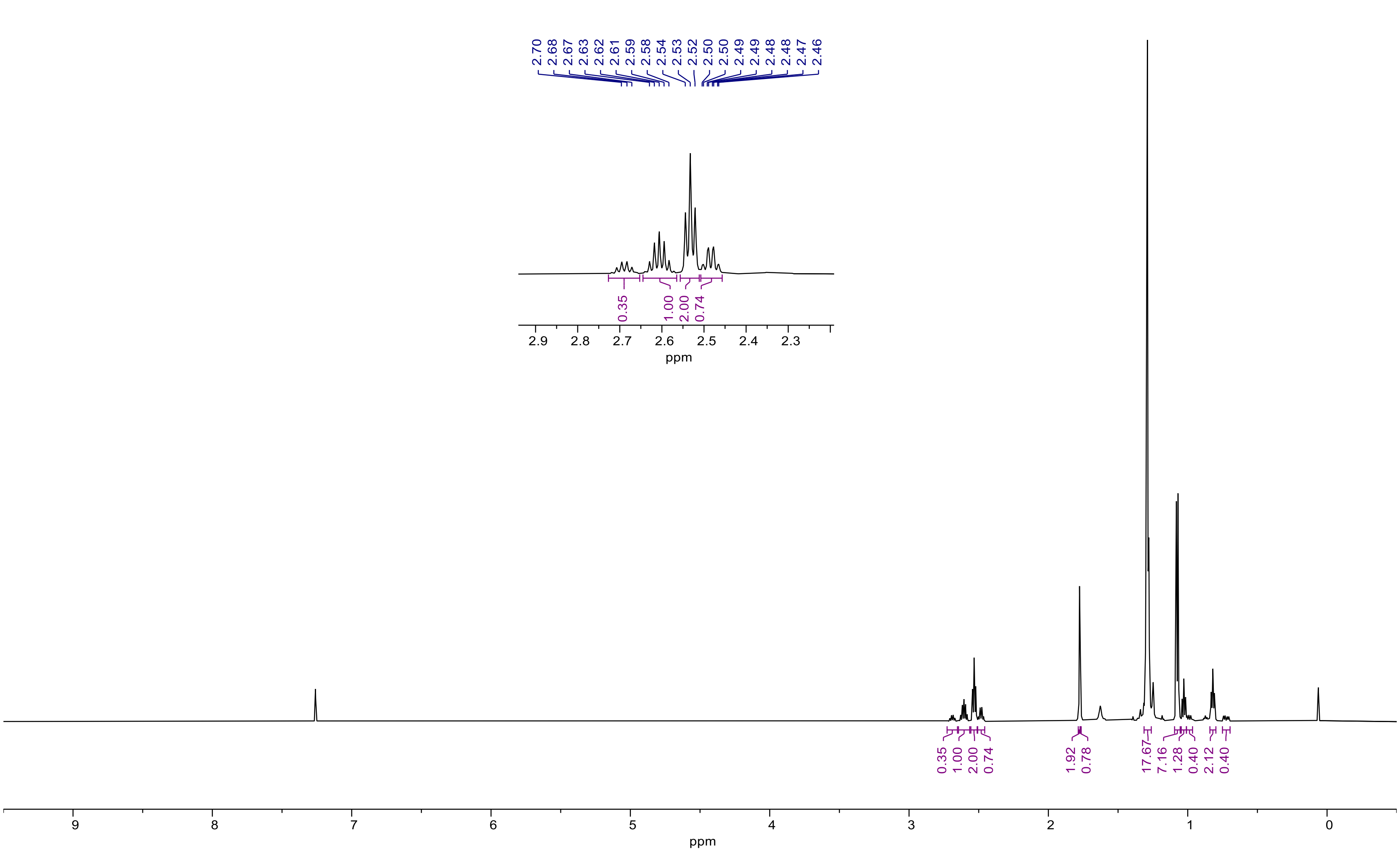

Figure S29. ^{1}H NMR Spectrum of **3i** (600 MHz, CDCl3).

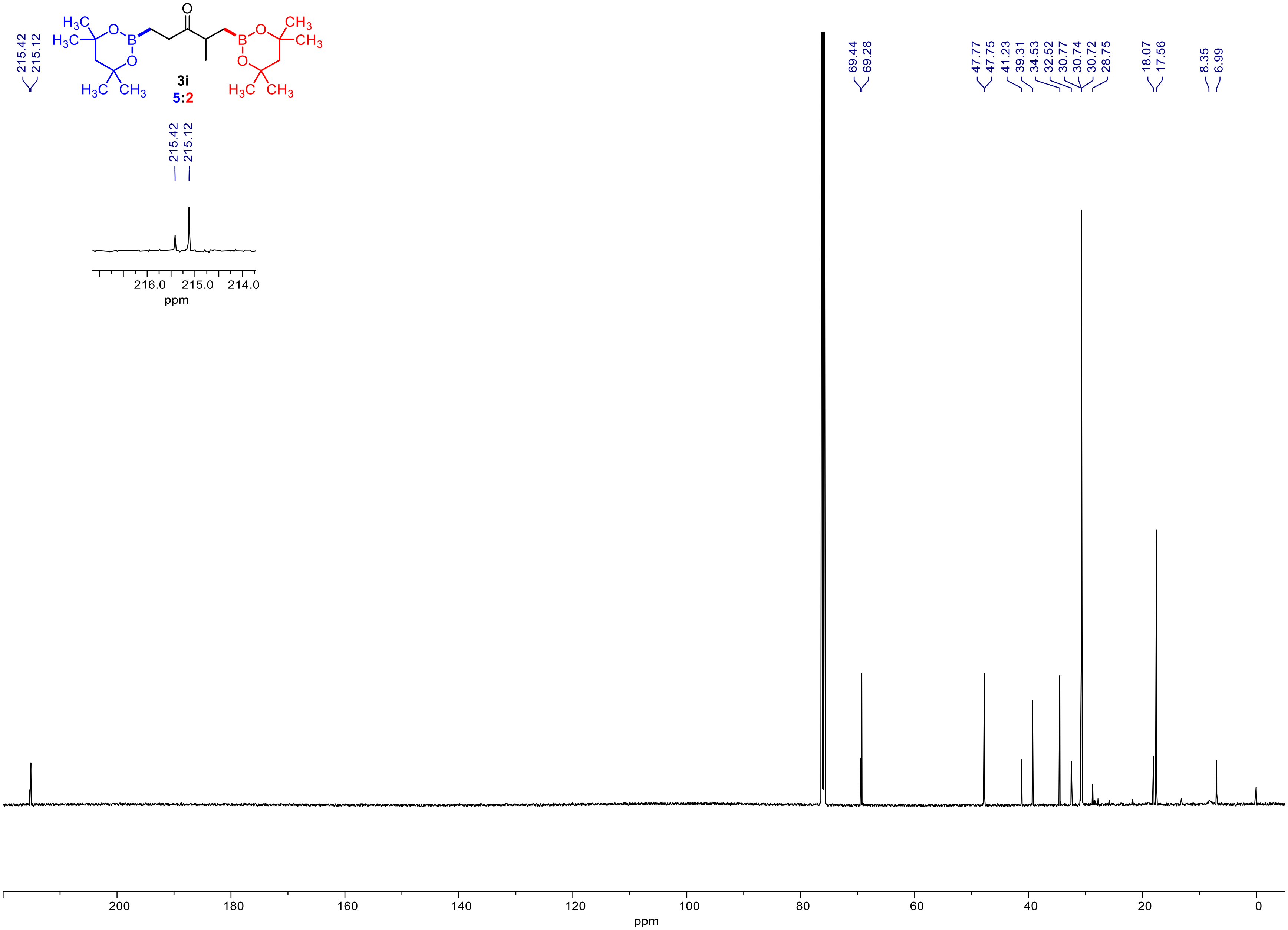

Figure S30. $^{13}C\{^{1}H\}$ NMR Spectrum of **3i** (150 MHz, $CDCl_3$).

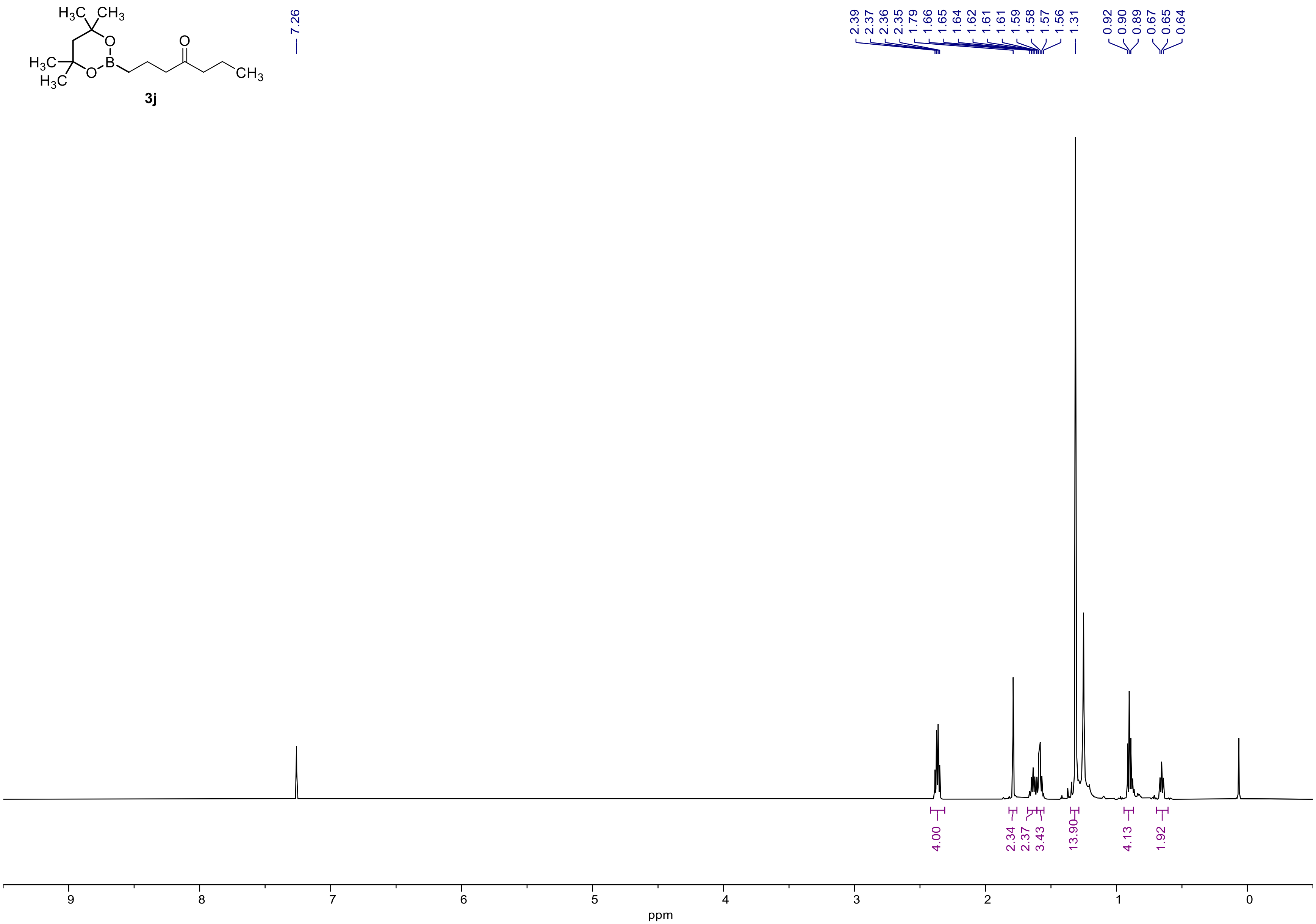

Figure S31. ^{1}H NMR Spectrum of **3j** (600 MHz, $CDCl_3$).

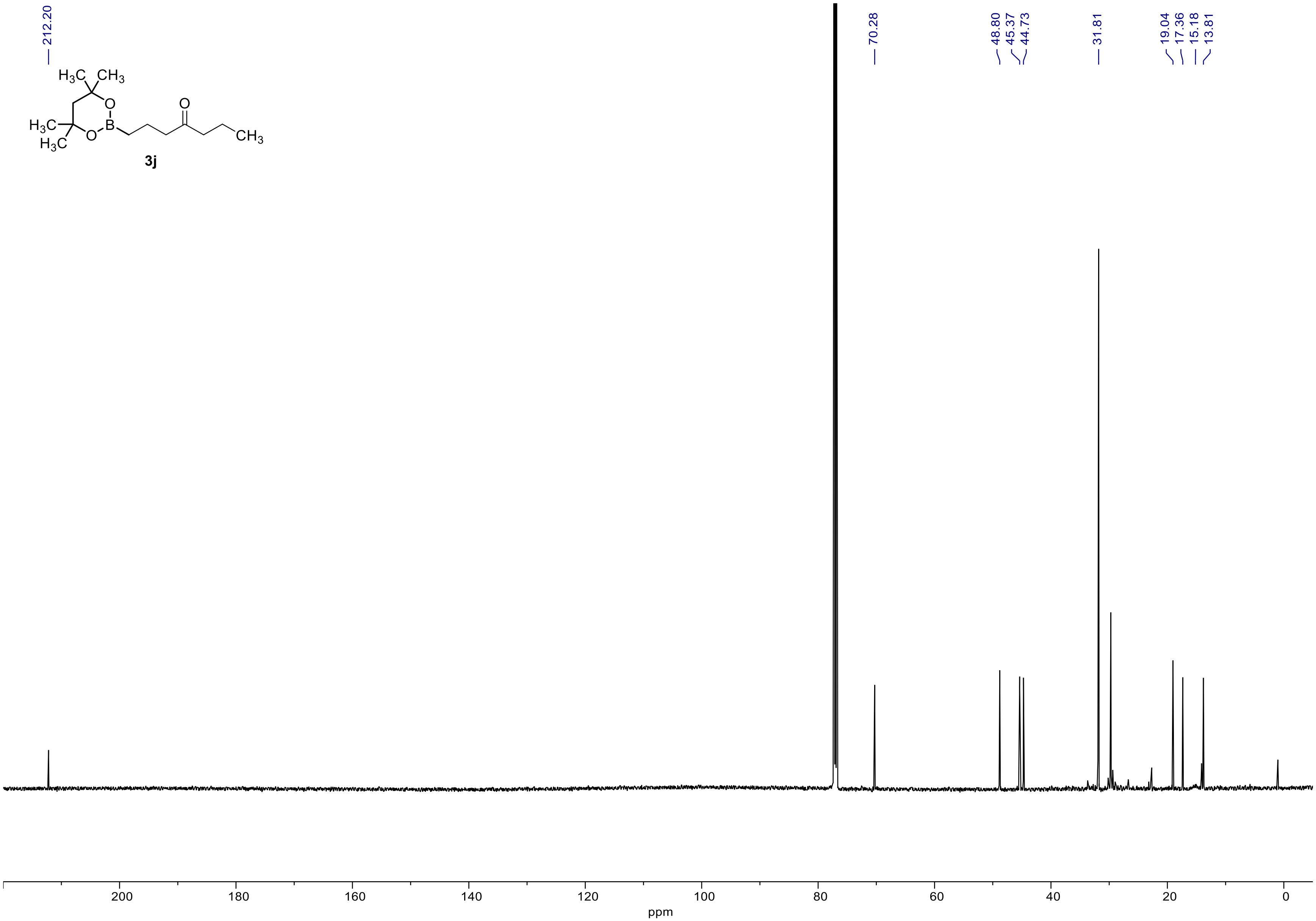

Figure S32. $^{13}C\{^{1}H\}$ NMR Spectrum of **3j** (150 MHz, $CDCl_3$).

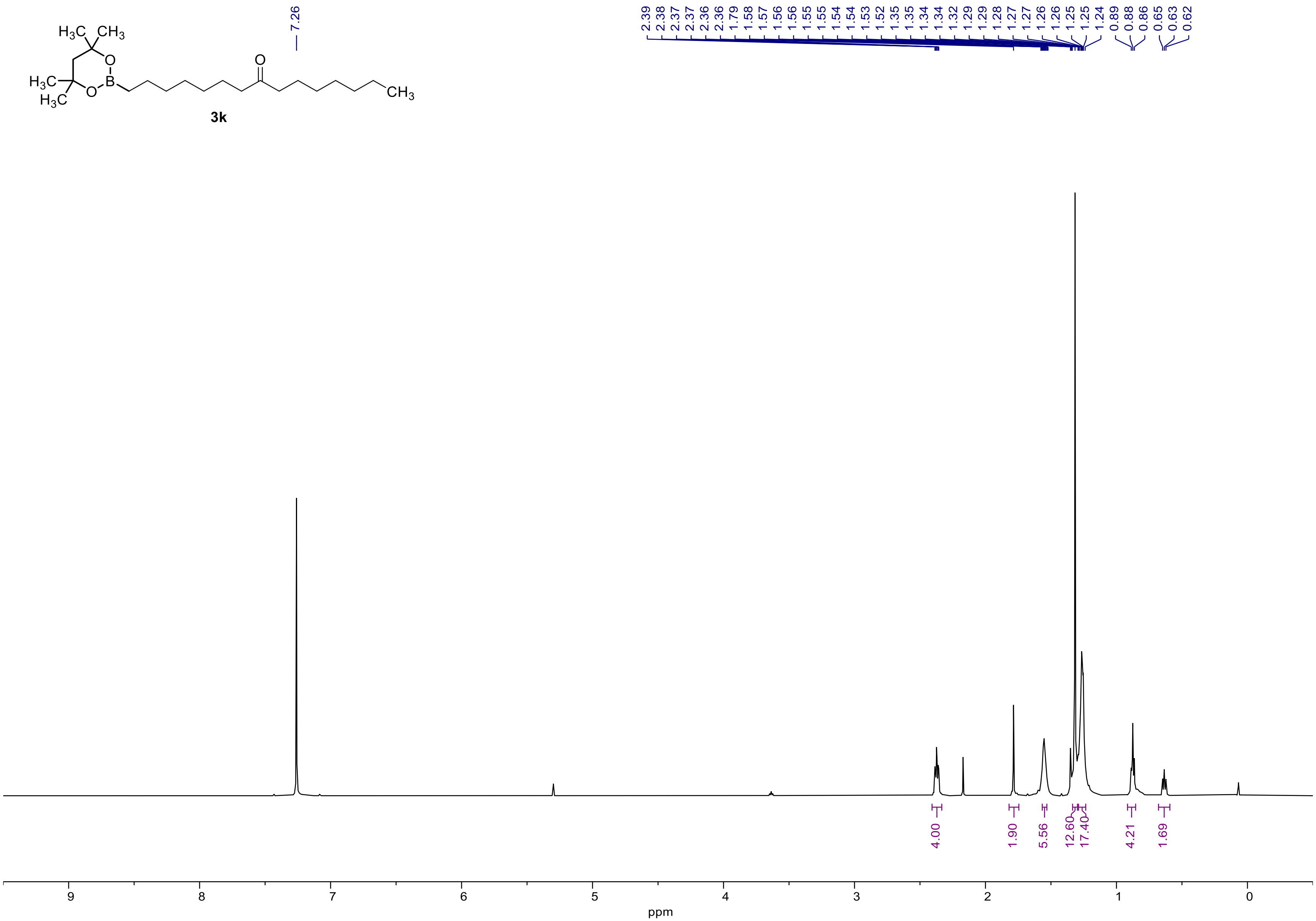

Figure S33. ^{1}H NMR Spectrum of **3k** (600 MHz, $CDCl_3$).

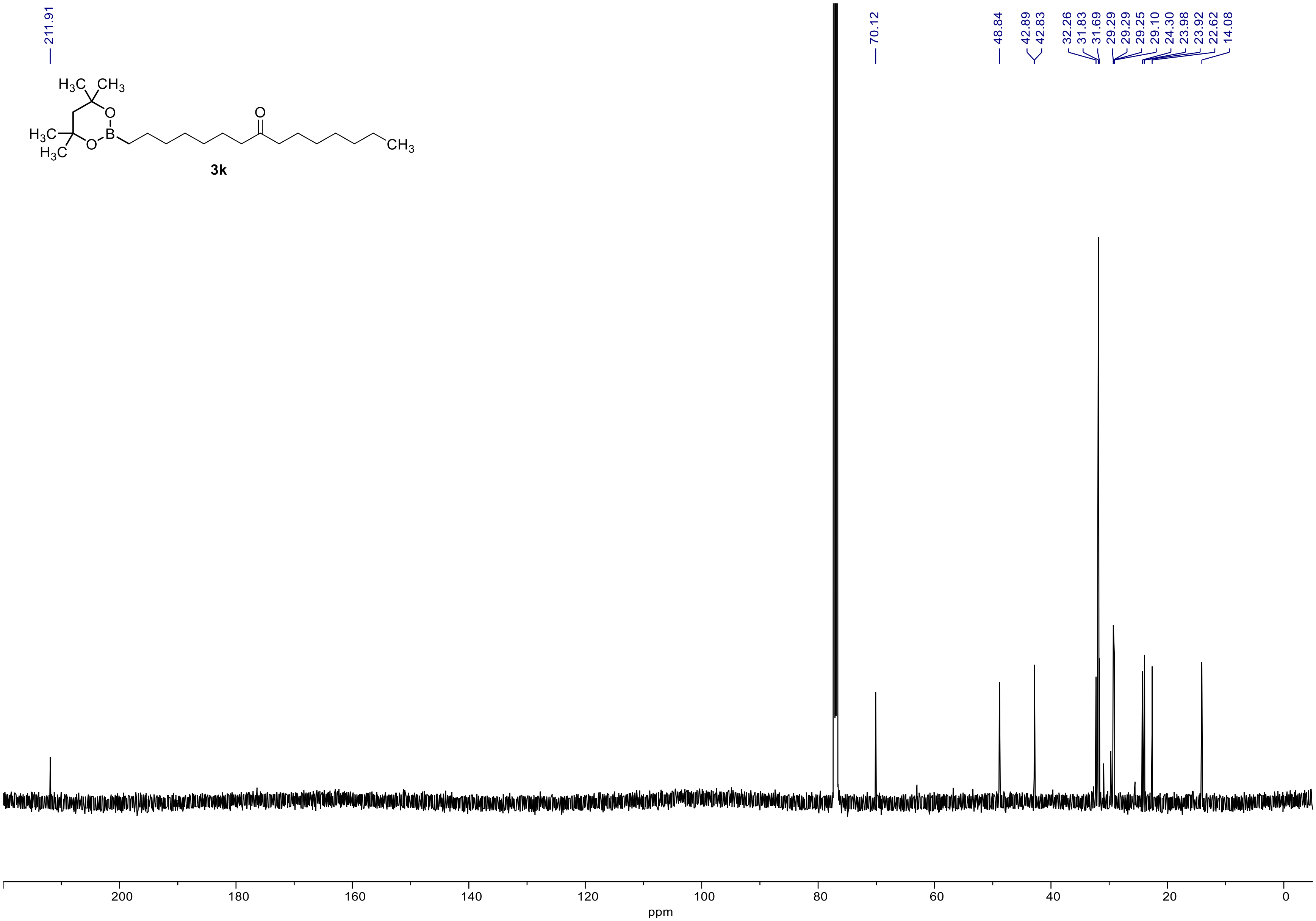

Figure S34. $^{13}C\{^{1}H\}$ NMR Spectrum of **3k** (150 MHz, $CDCl_3$).

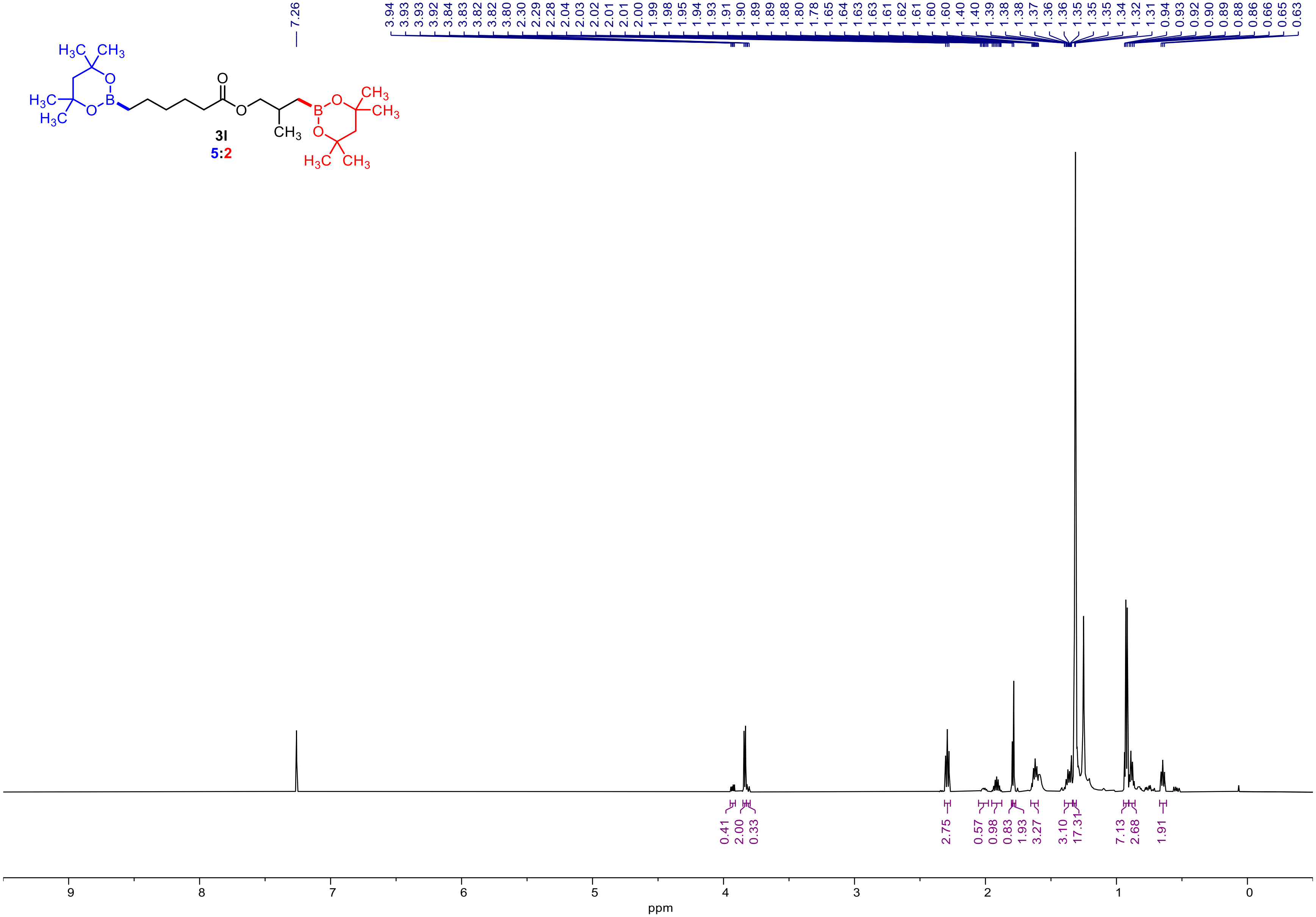

Figure S35. ^{1}H NMR Spectrum of **3l** (600 MHz, $CDCl_3$).

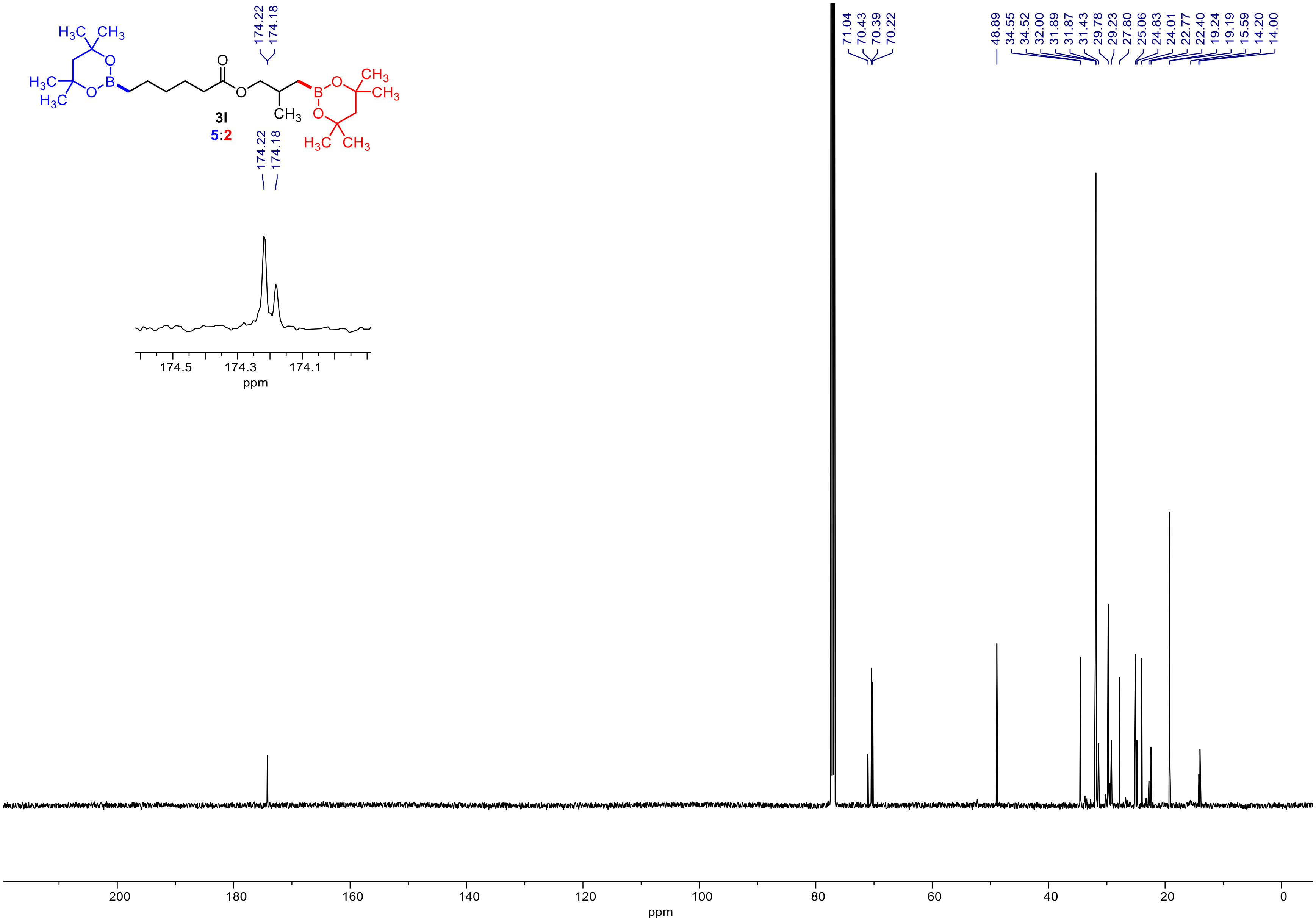

Figure S36. $^{13}C\{^{1}H\}$ NMR Spectrum of **3l** (150 MHz, $CDCl_3$).

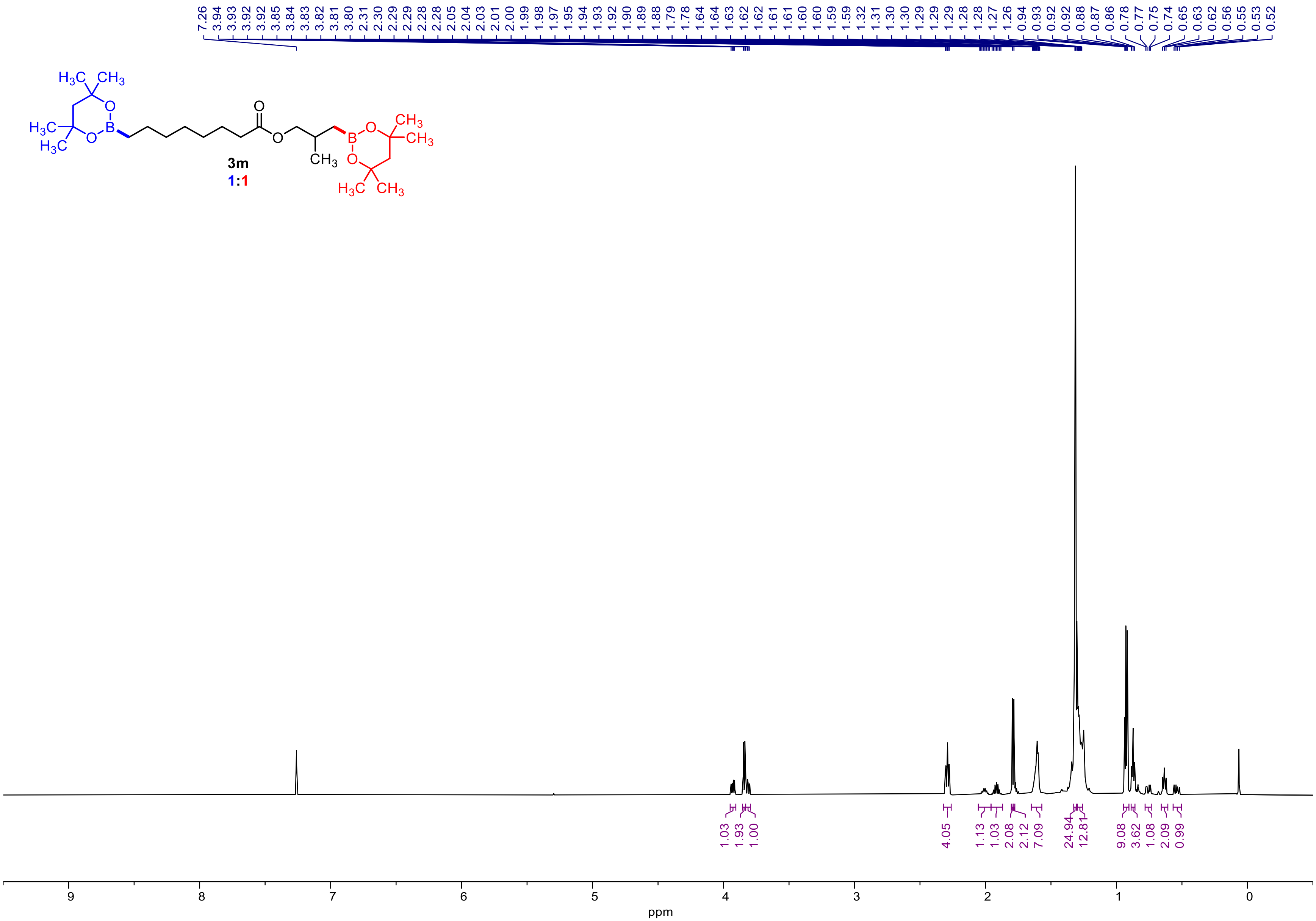

Figure S37. ¹H NMR Spectrum of **3m** (600 MHz, $CDCl_3$).

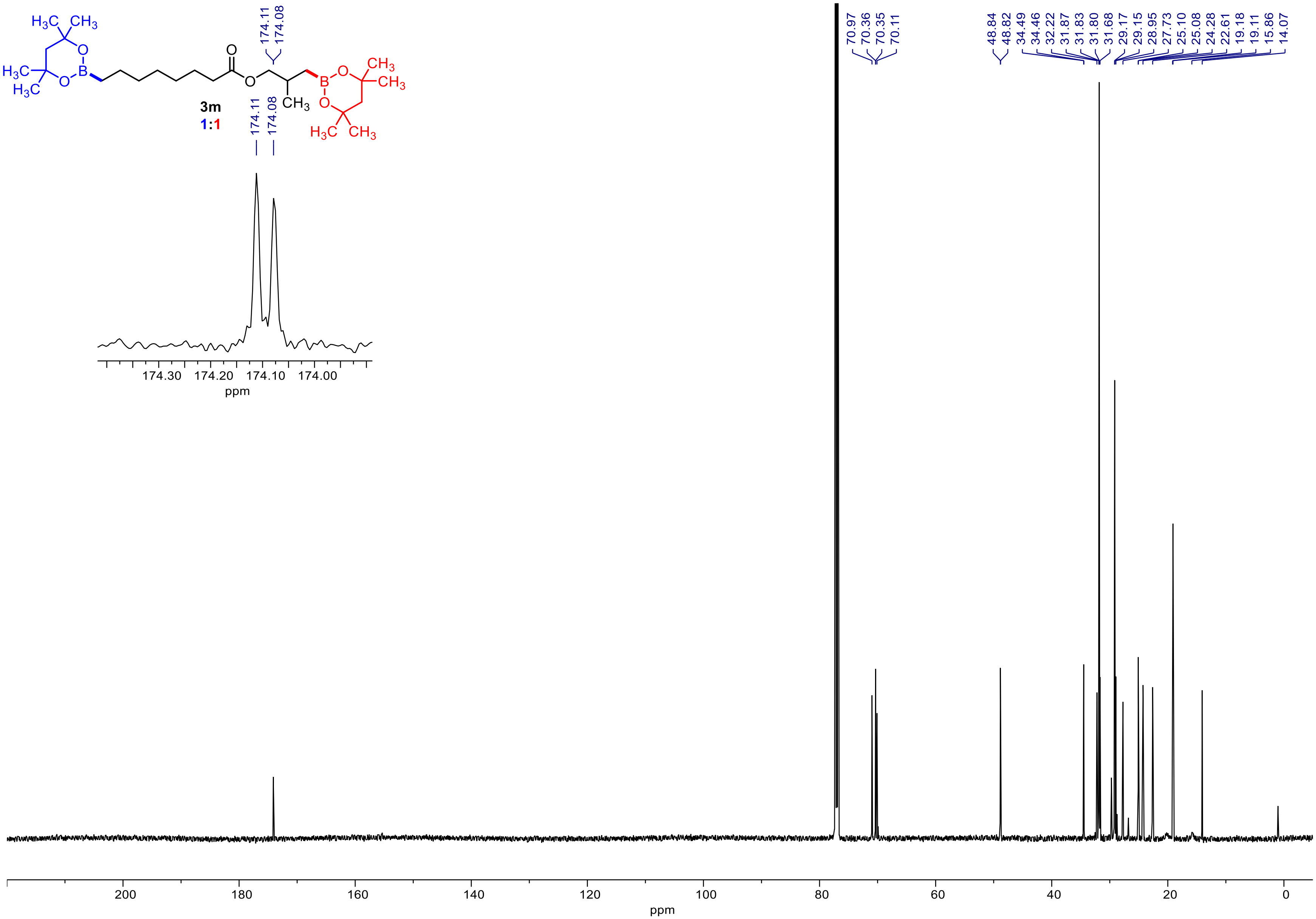

Figure S38. $^{13}C\{^{1}H\}$ NMR Spectrum of **3m** (150 MHz, $CDCl_3$).

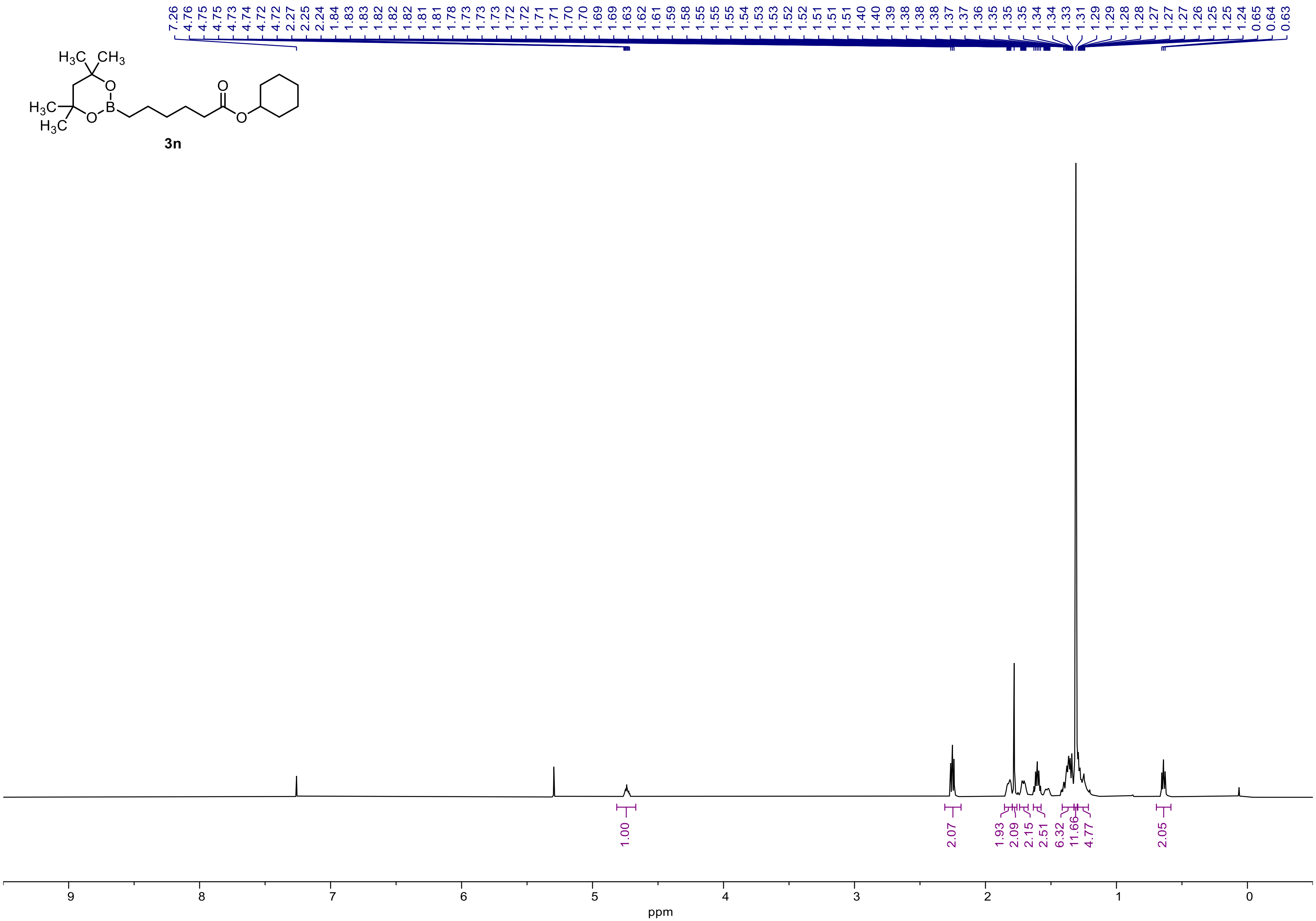

Figure S39. ^{1}H NMR Spectrum of **3n** (600 MHz, $CDCl_3$).

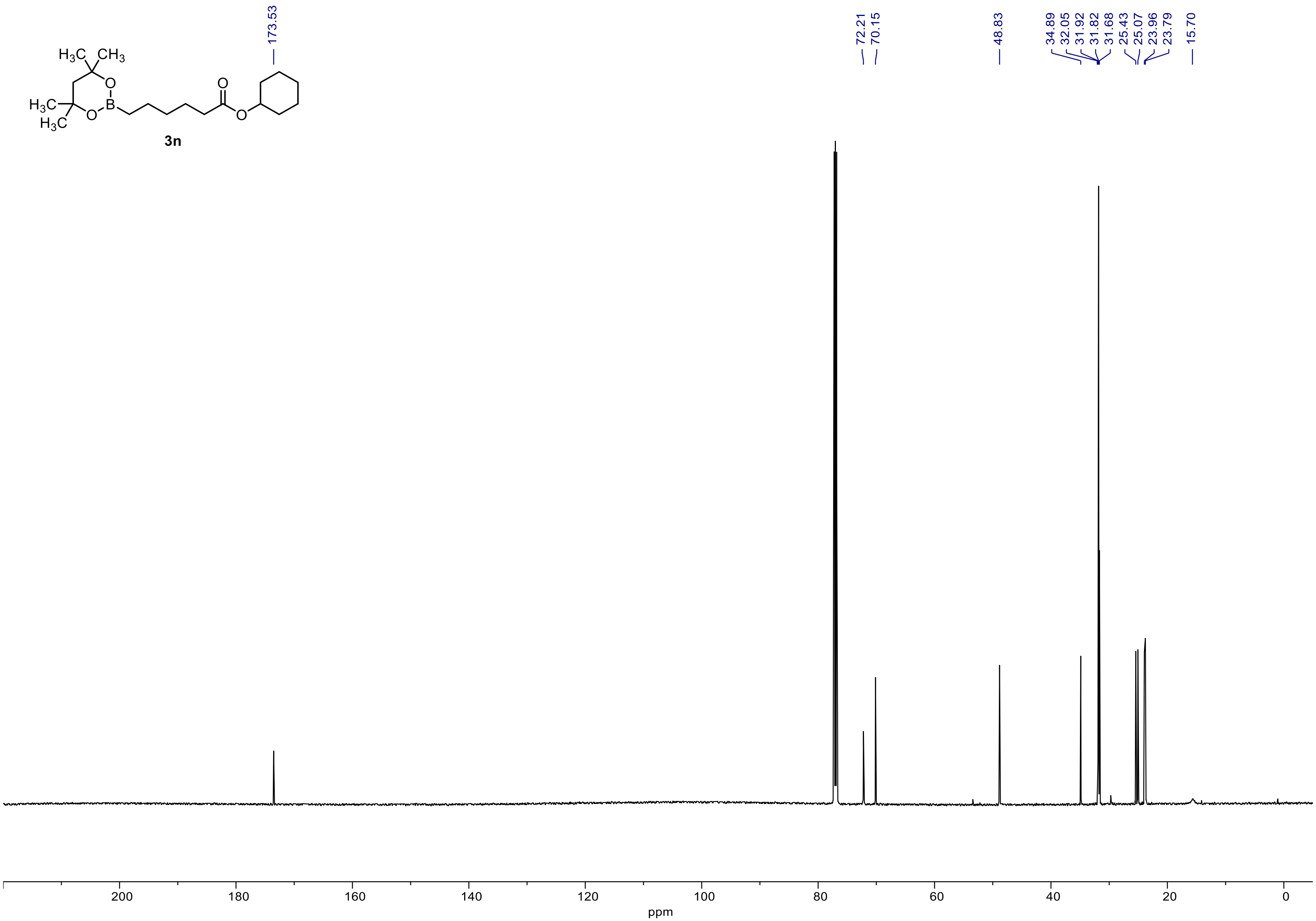

Figure S40. $^{13}C\{^{1}H\}$ NMR Spectrum of **3n** (150 MHz, $CDCl_3$).

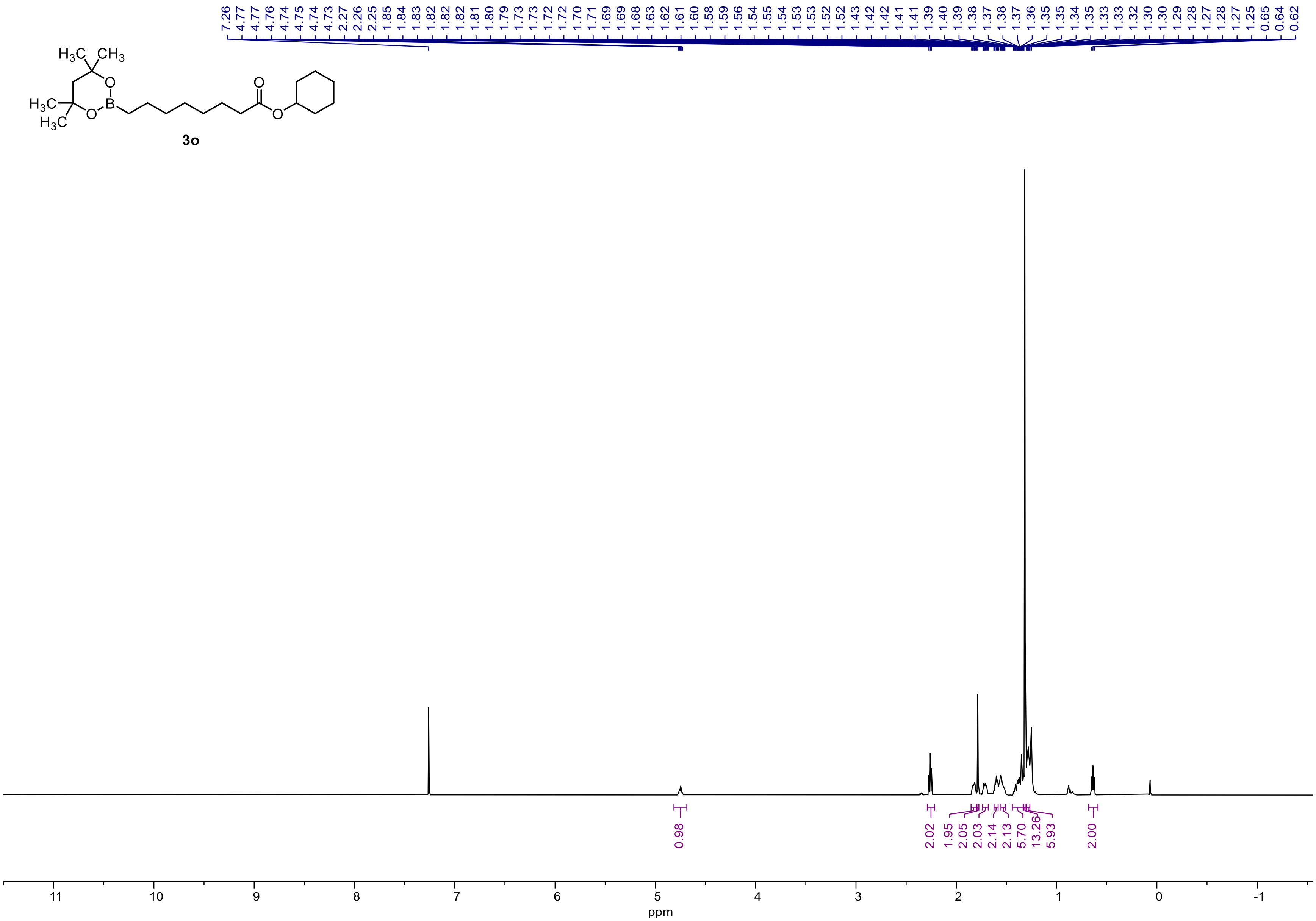

Figure S41. ^{1}H NMR Spectrum of **3o** (600 MHz, $CDCl_3$).

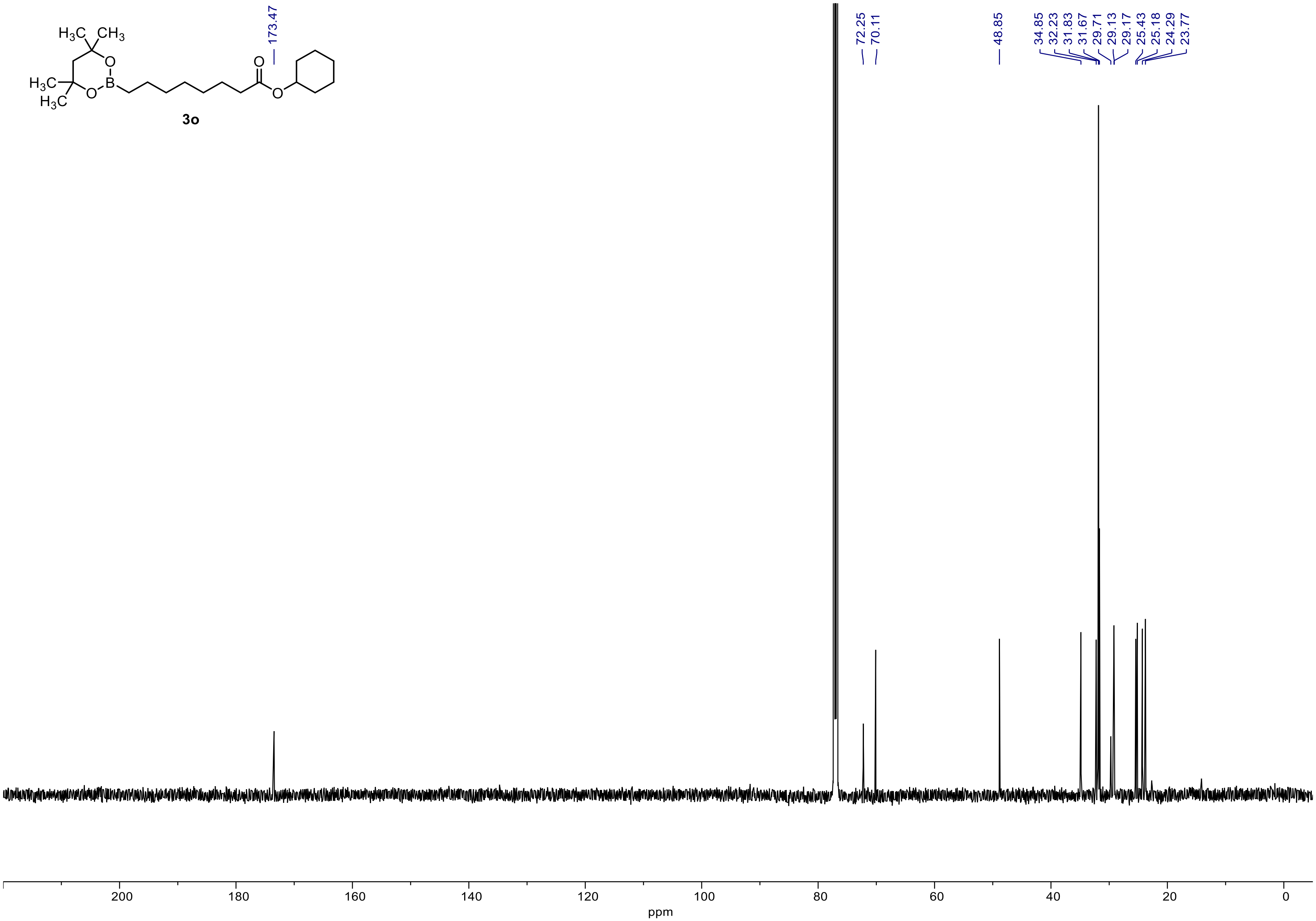

Figure S42. $^{13}C\{^{1}H\}$ NMR Spectrum of **3o** (150 MHz, $CDCl_3$).

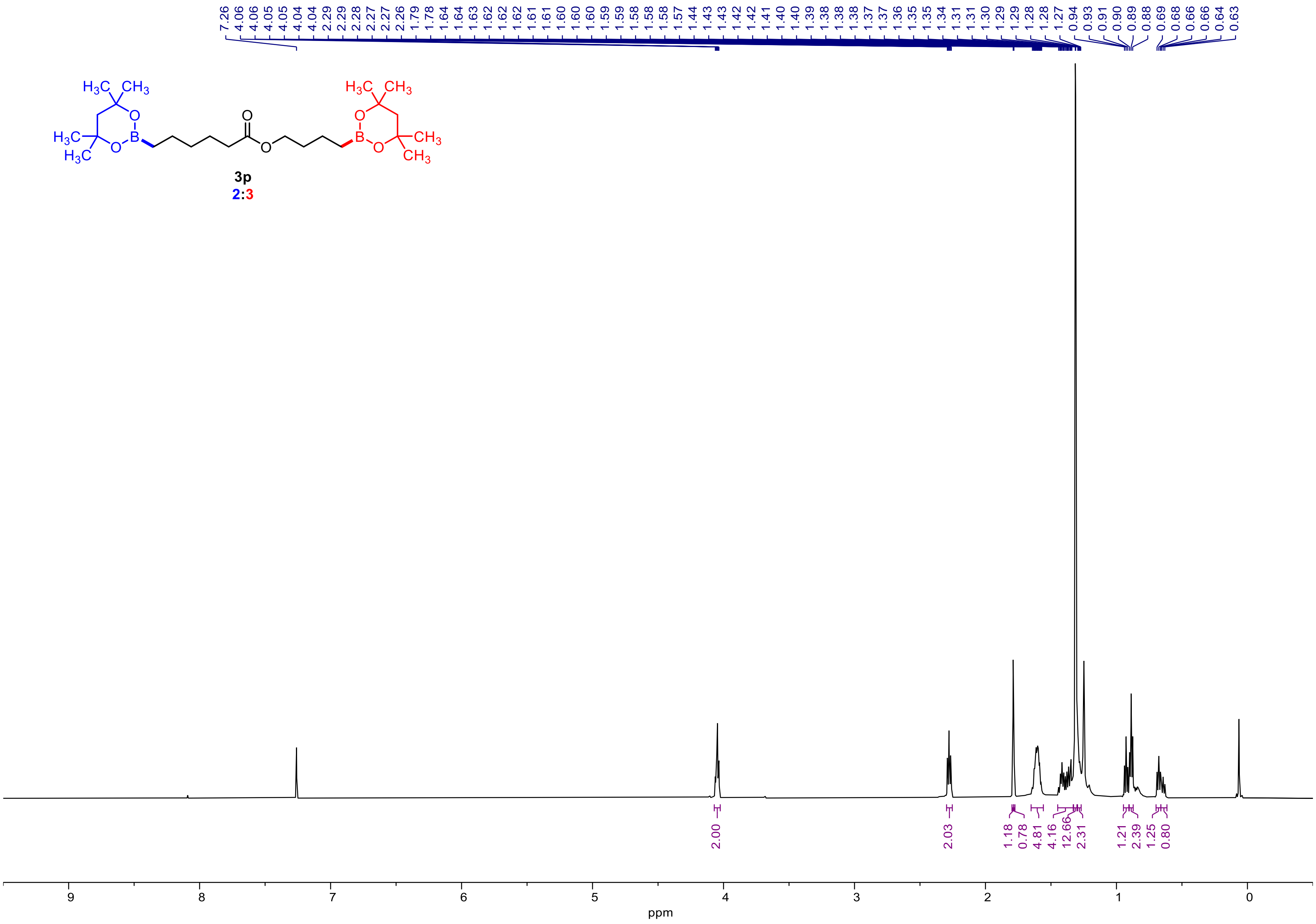

Figure S43. ¹H NMR Spectrum of **3p** (600 MHz, $CDCl_3$).

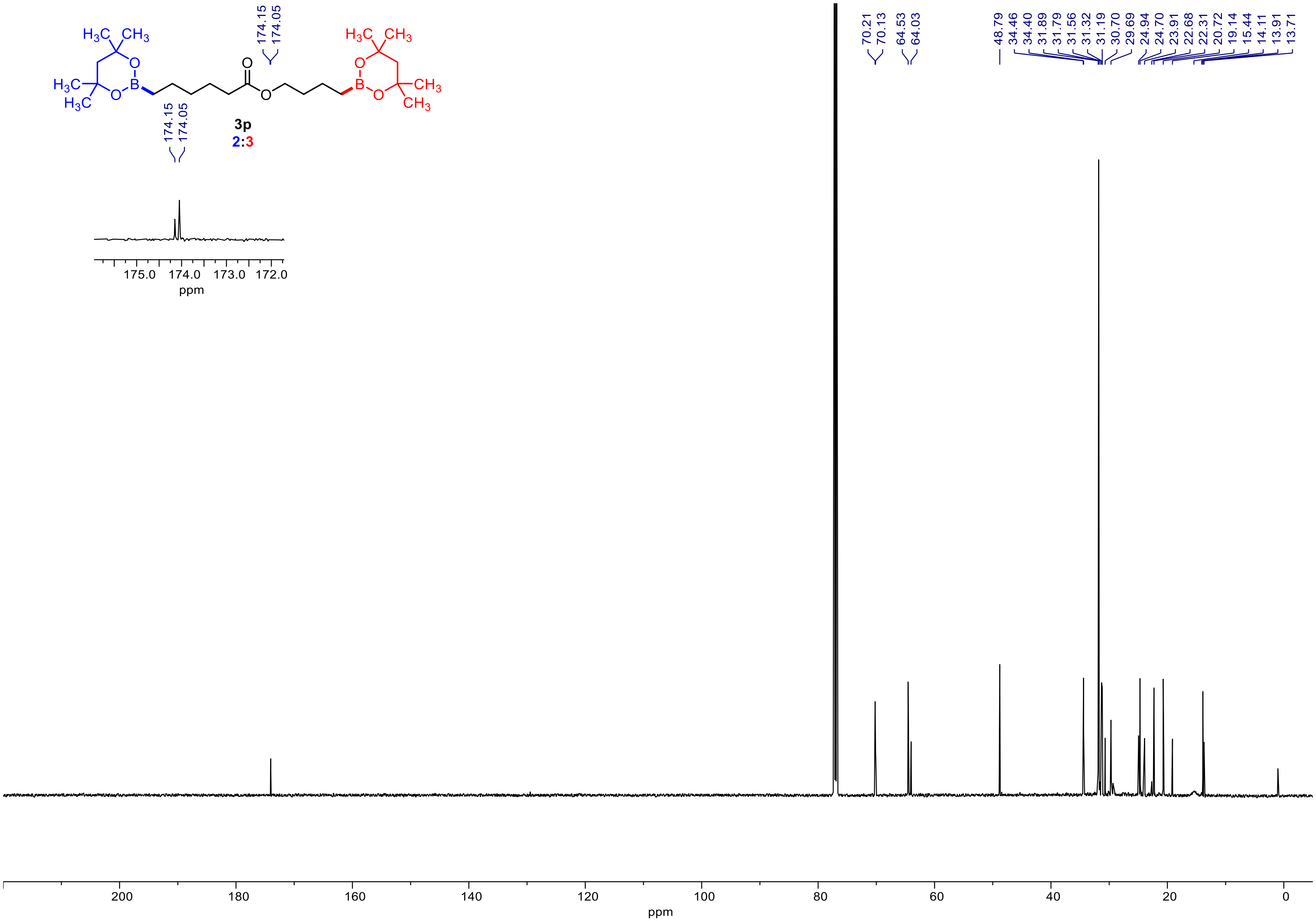

Figure S44. $^{13}C\{^{1}H\}$ NMR Spectrum of **3p** (150 MHz, $CDCl_3$).

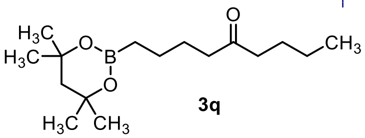

3q

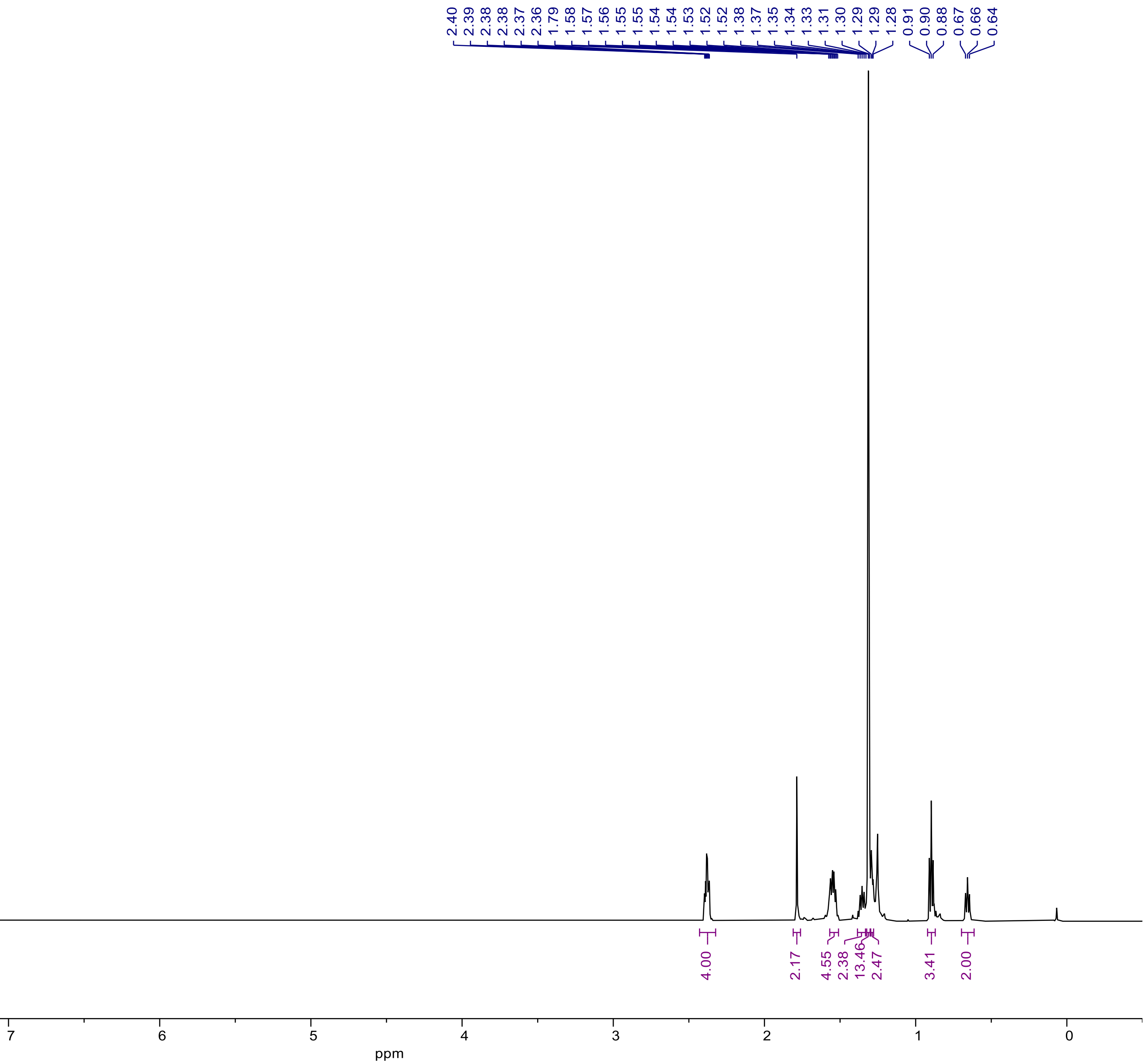

Figure S45. ¹H NMR Spectrum of **3q** (600 MHz, $CDCl_3$).

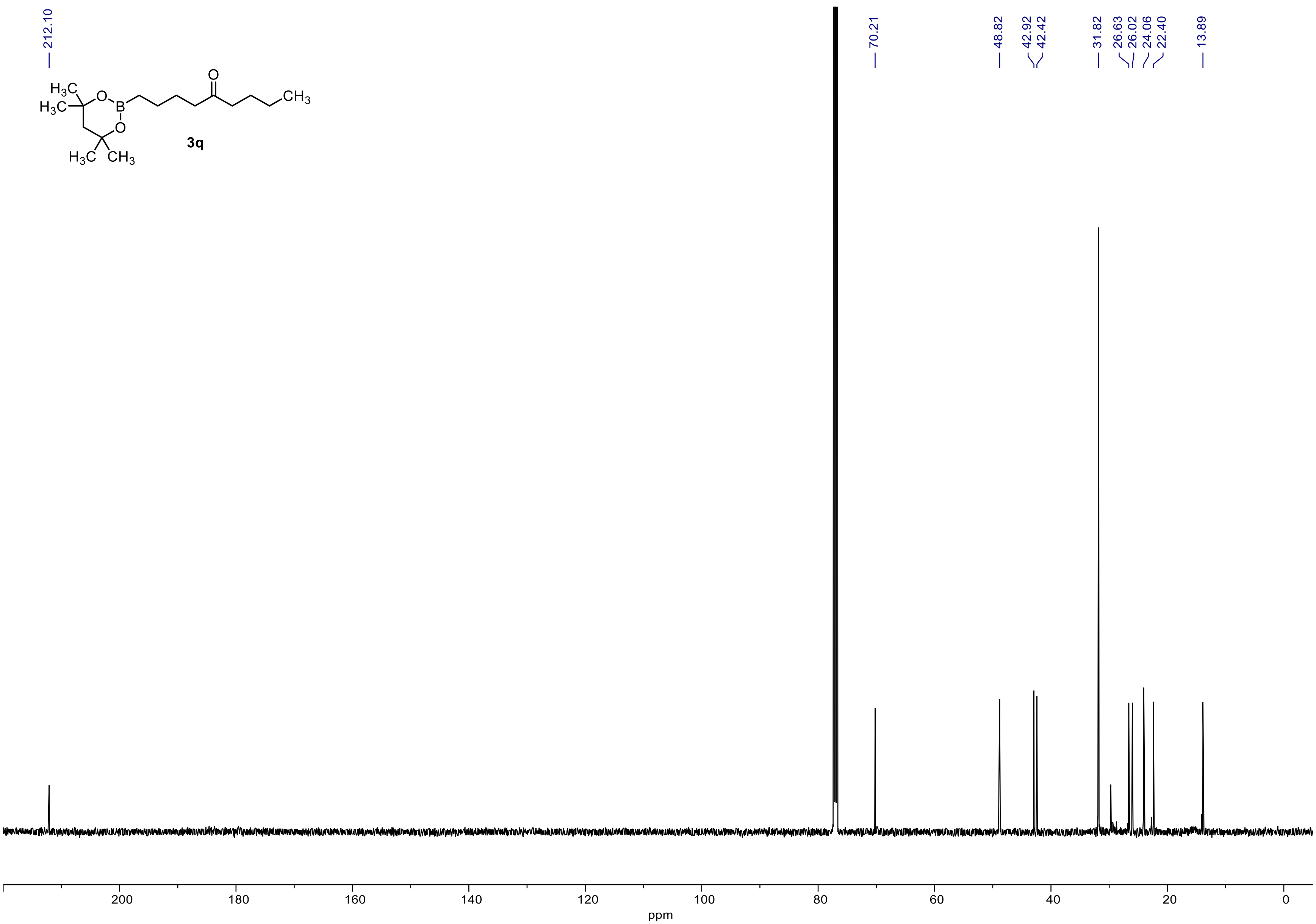

Figure S46. $^{13}C\{^{1}H\}$ NMR Spectrum of **3q** (150 MHz, $CDCl_3$).

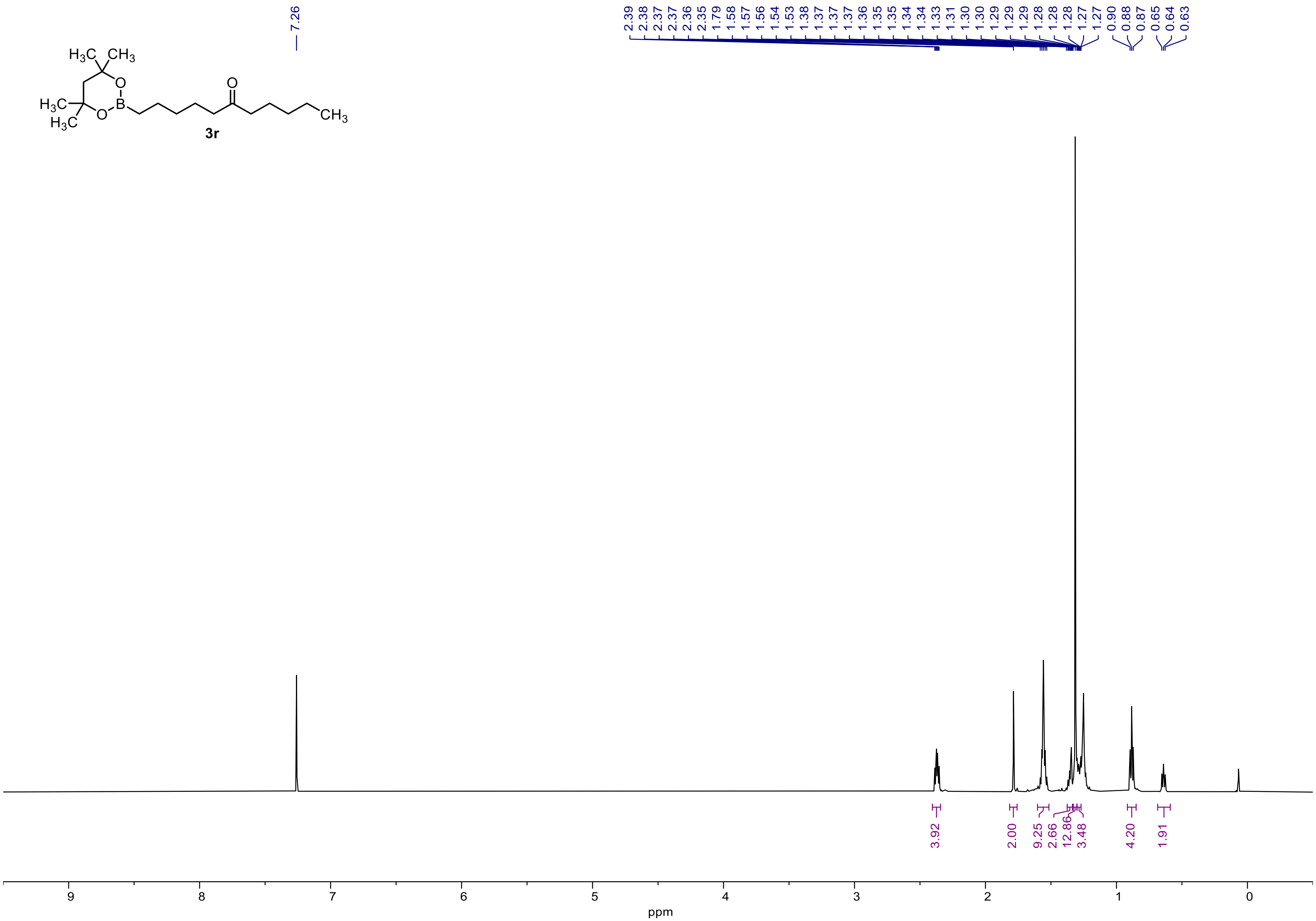

Figure S47. ^{1}H NMR Spectrum of **3r** (600 MHz, $CDCl_3$).

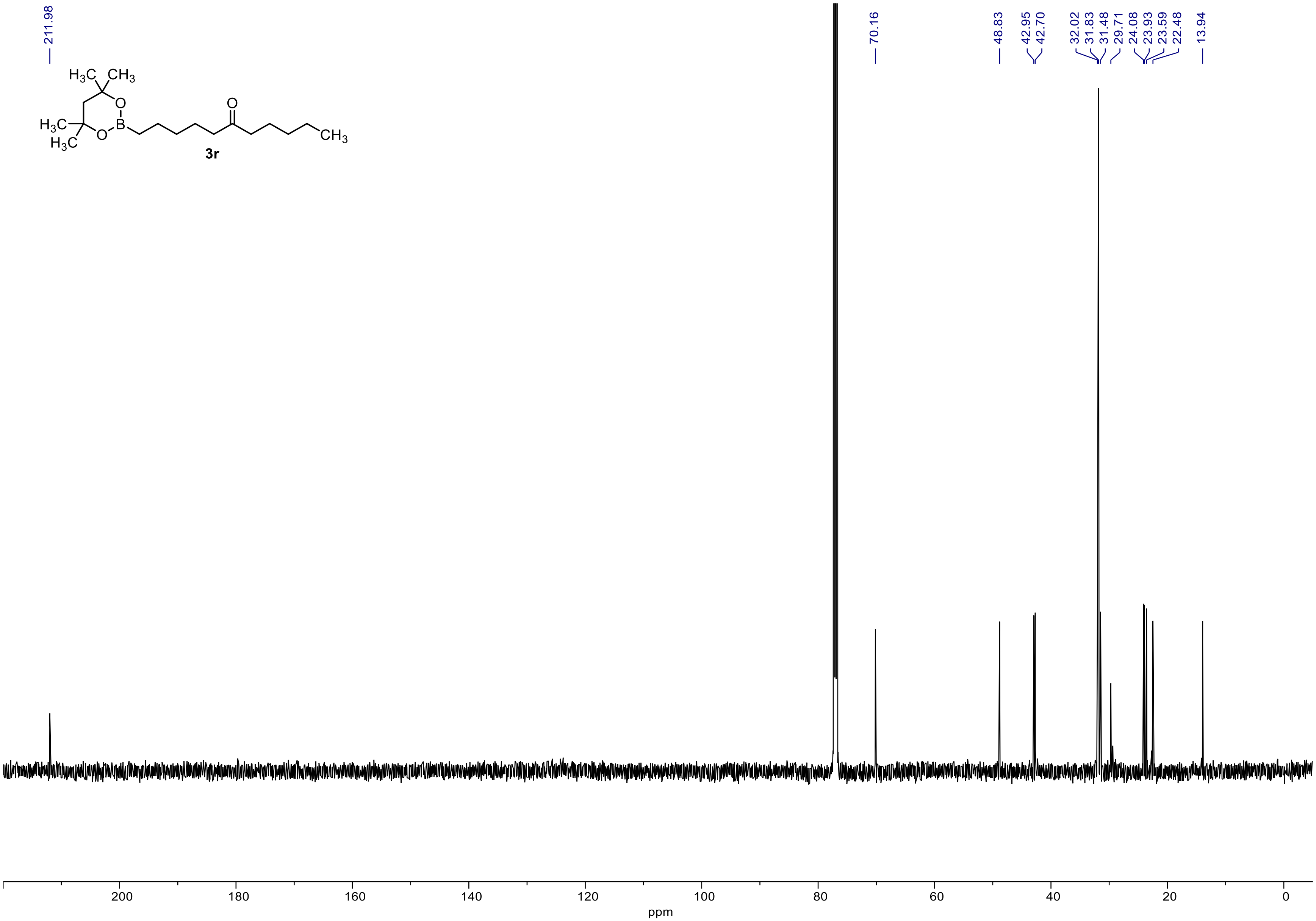

Figure S48. $^{13}C\{^{1}H\}$ NMR Spectrum of **3r** (150 MHz, $CDCl_3$).

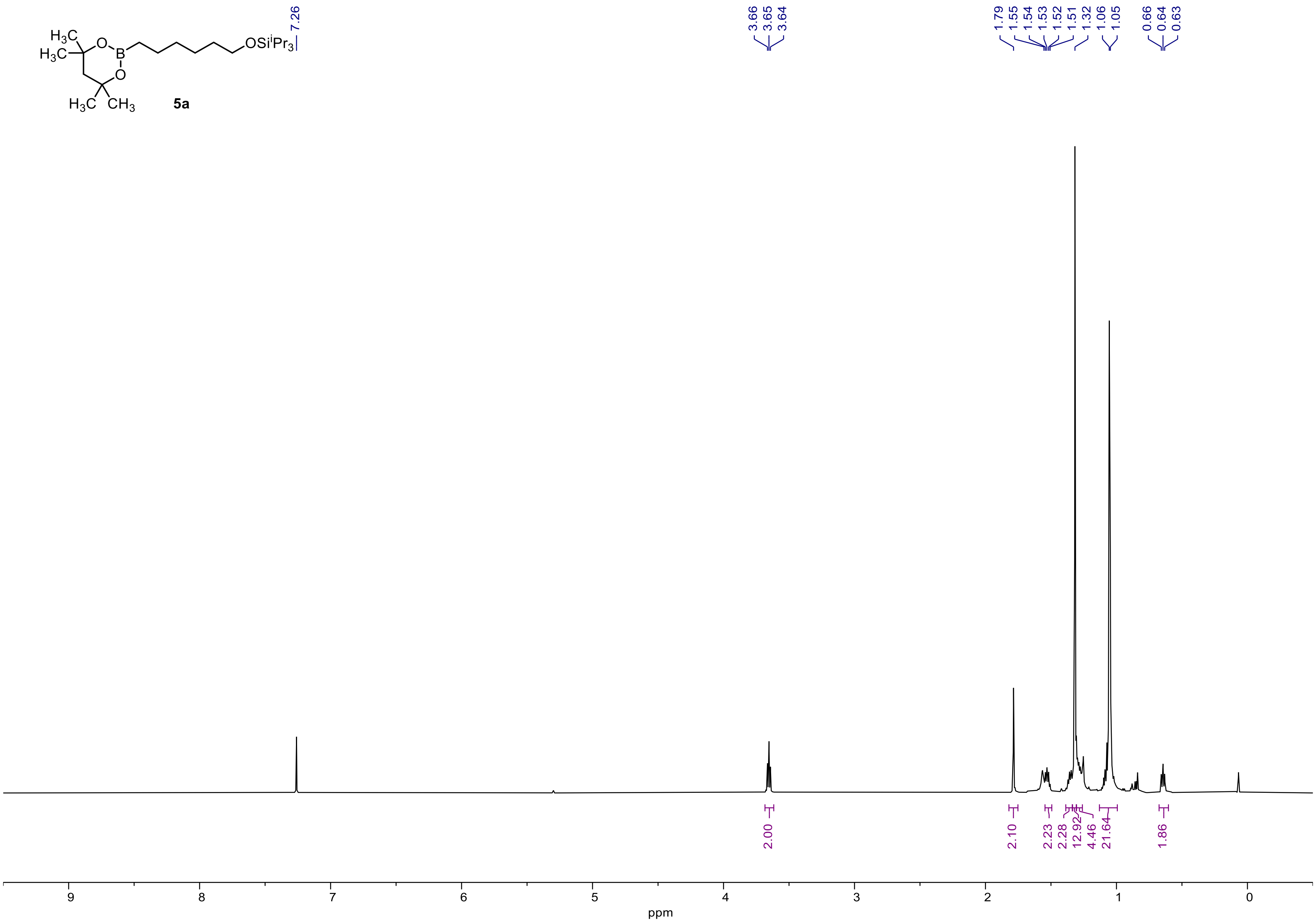

Figure S49. ^{1}H NMR Spectrum of **5a** (600 MHz, $CDCl_3$).

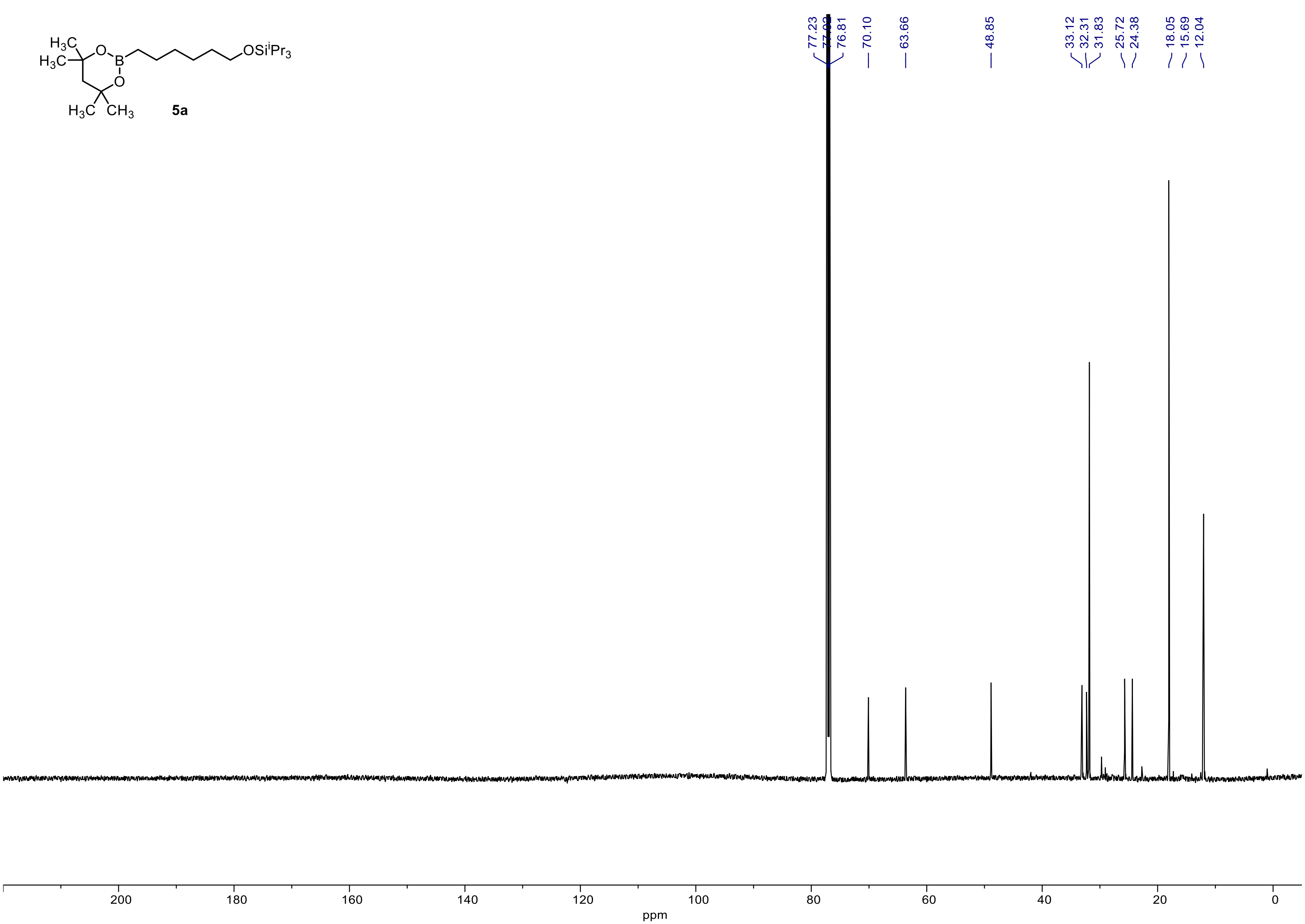

Figure S50. $^{13}C\{^1H\}$ NMR Spectrum of **5a** (150 MHz, $CDCl_3$).

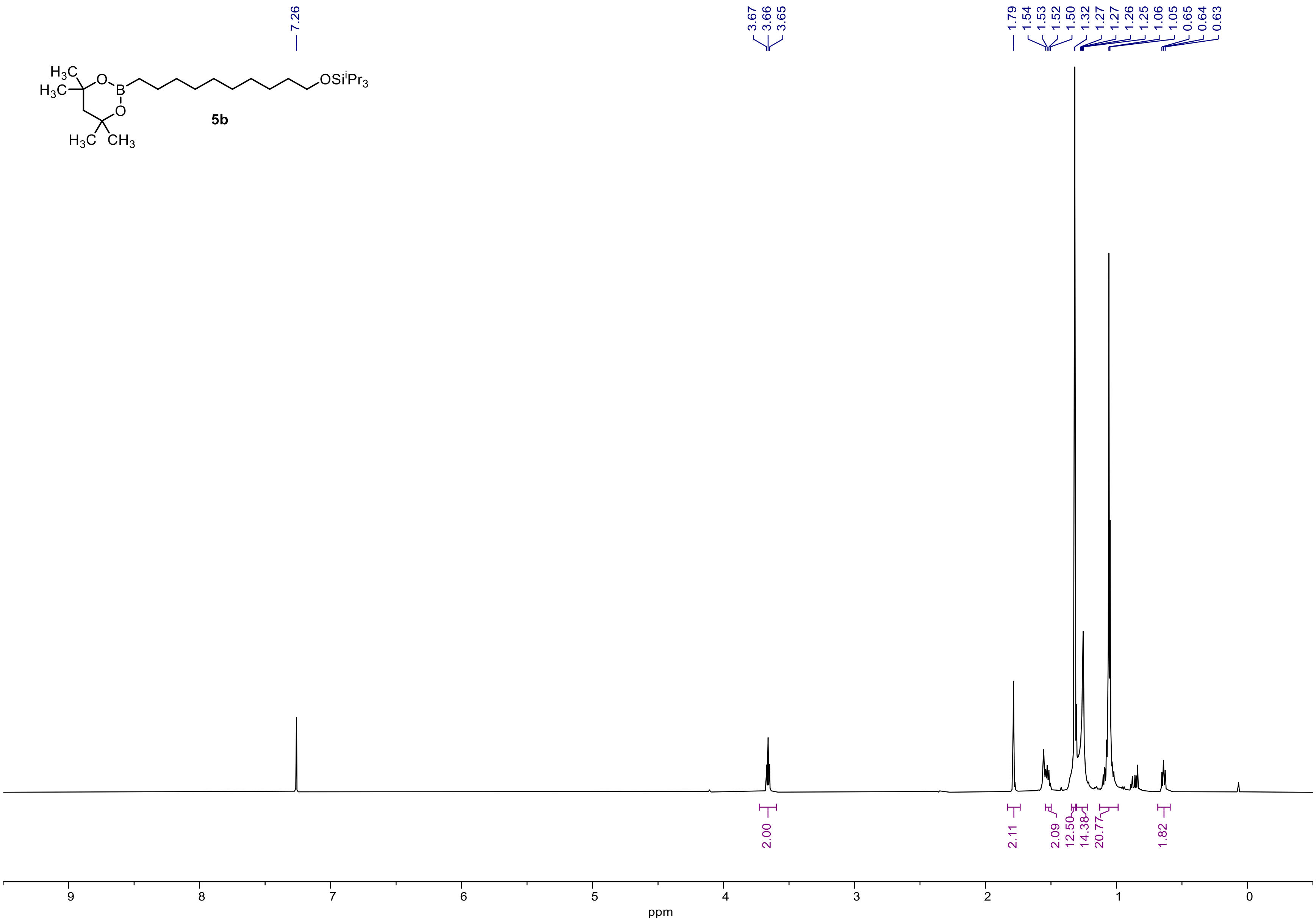

Figure S51. ^{1}H NMR Spectrum of **5b** (600 MHz, $CDCl_3$).

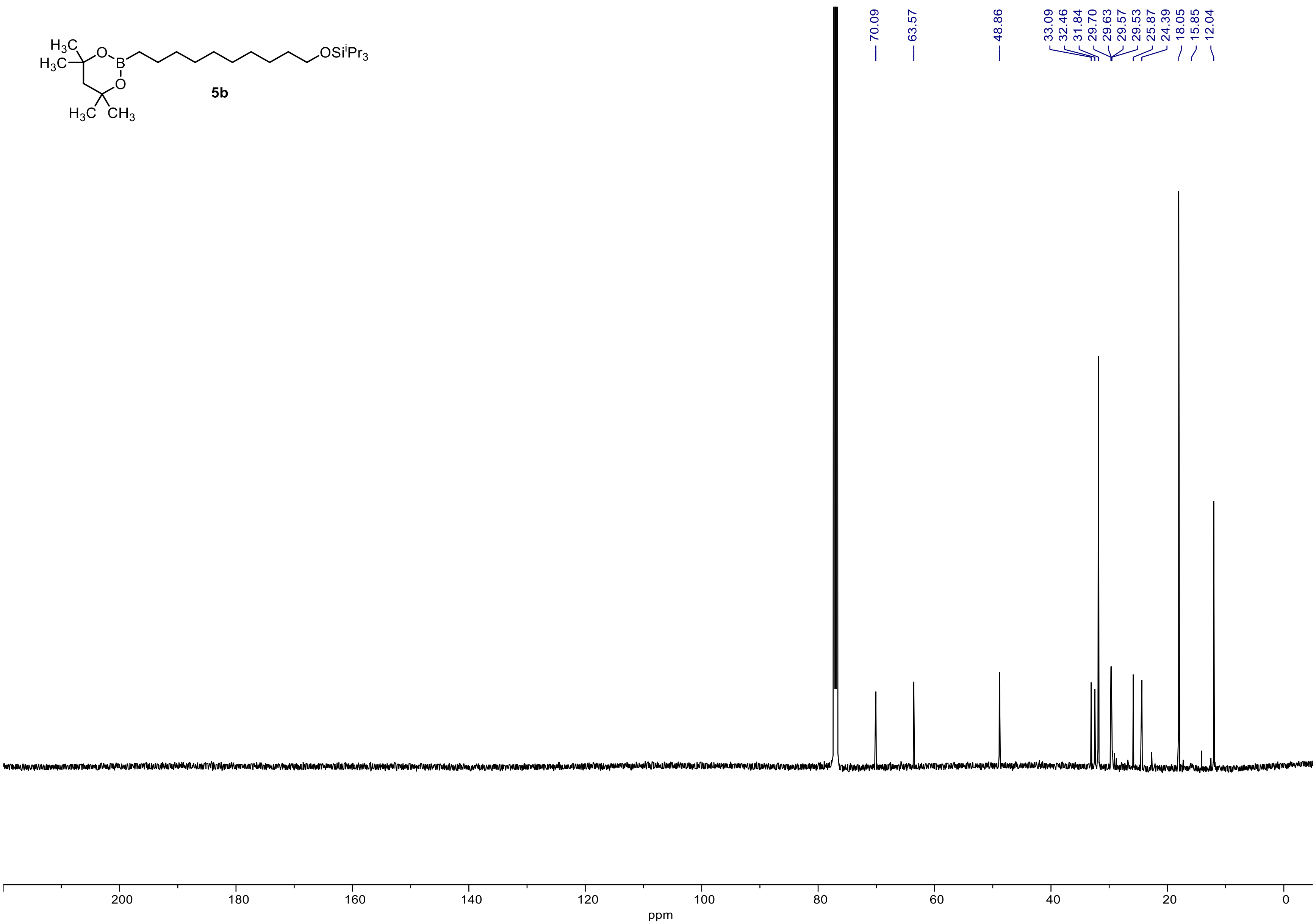

Figure S52. $^{13}C\{^{1}H\}$ NMR Spectrum of **5b** (150 MHz, $CDCl_3$).

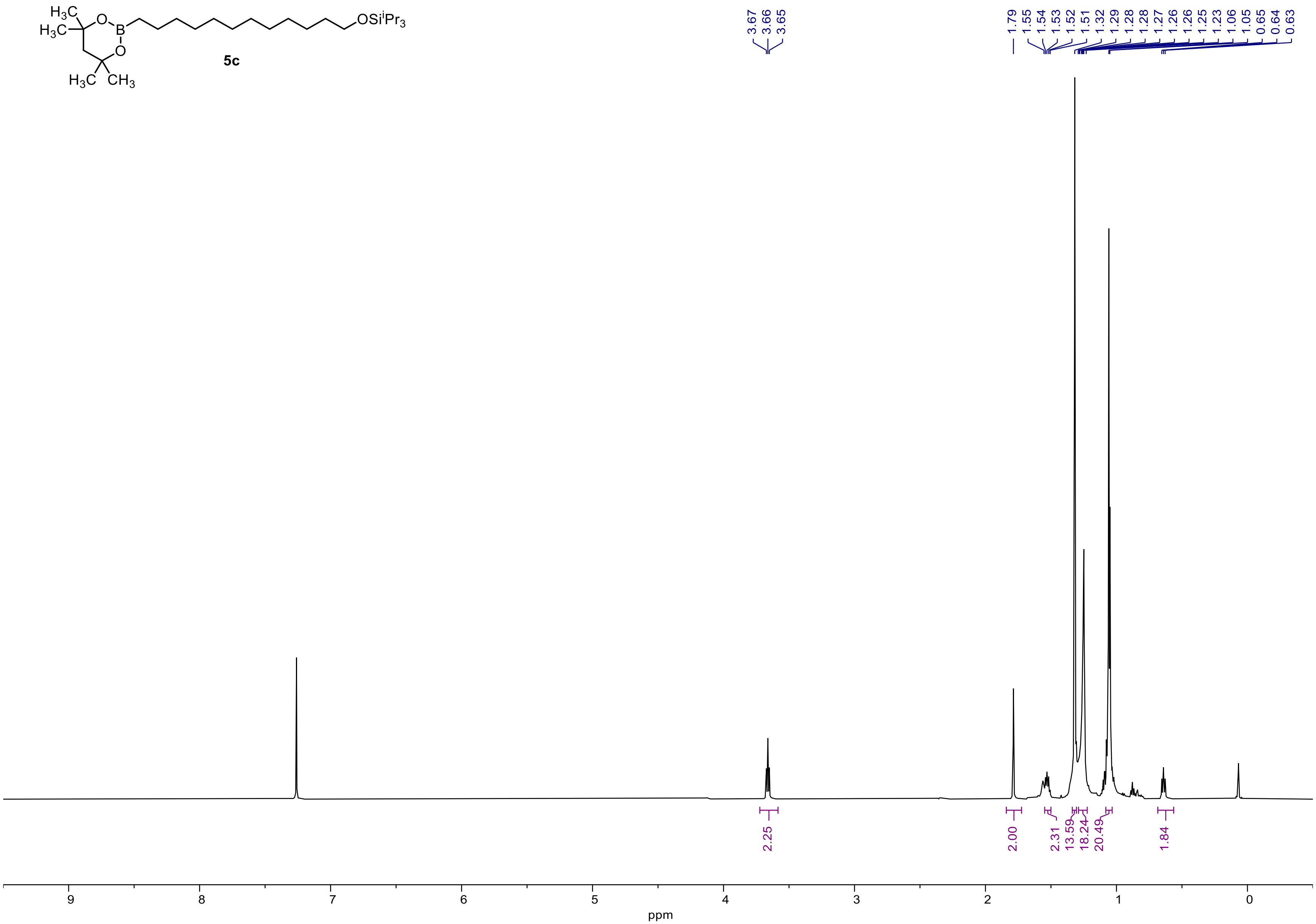

Figure S53. ^{1}H NMR Spectrum of **5c** (600 MHz, $CDCl_3$).

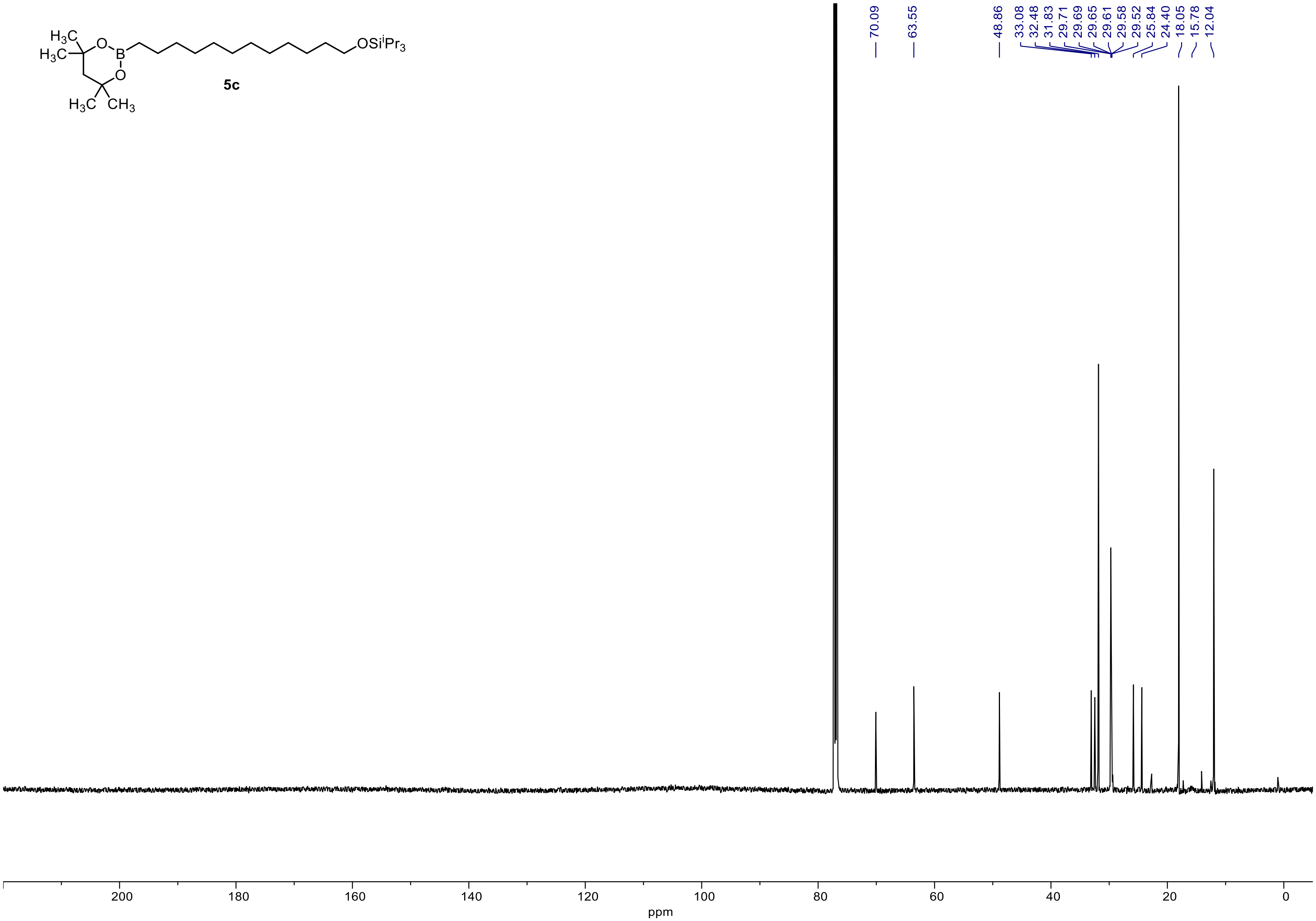

Figure S54. $^{13}C\{^{1}H\}$ NMR Spectrum of **5c** (150 MHz, $CDCl_3$).

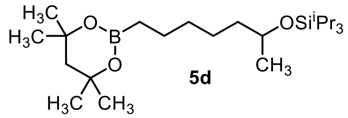

5d

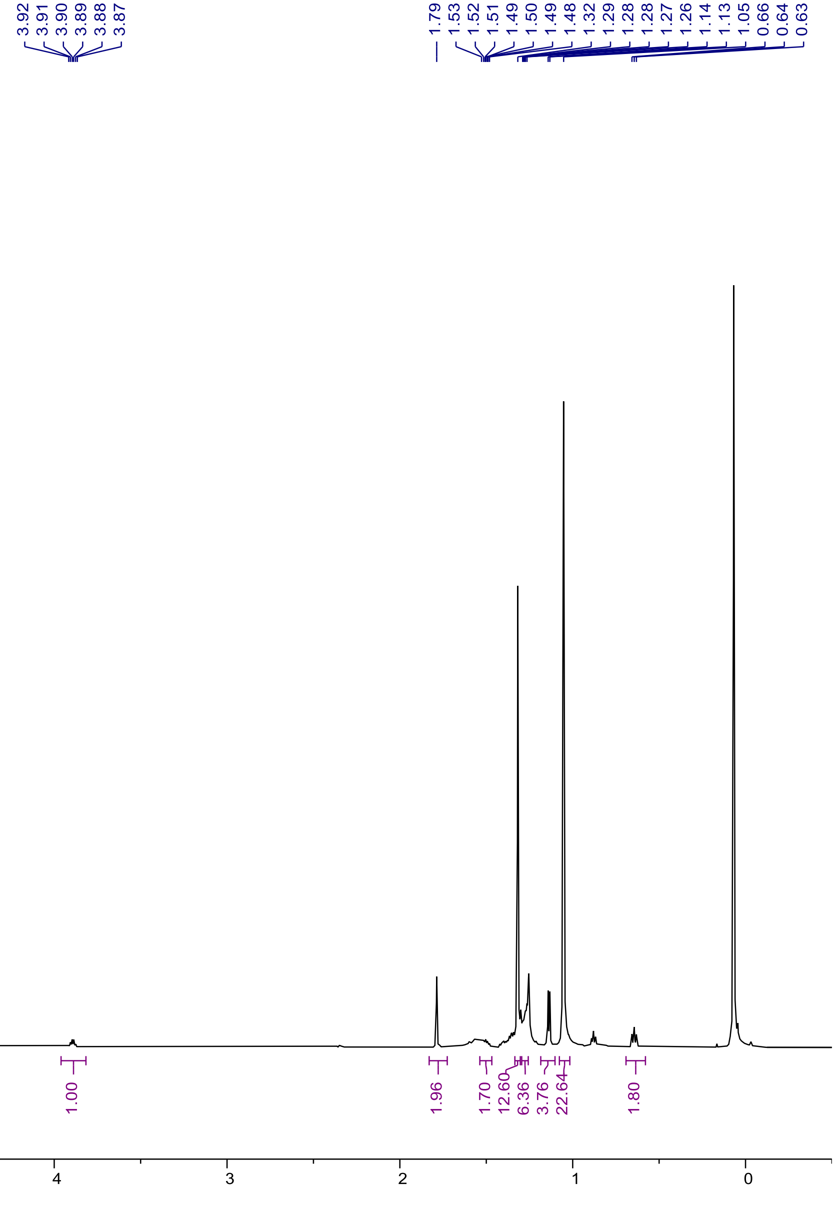

Figure S55. ^{1}H NMR Spectrum of **5d** (600 MHz, $CDCl_3$).

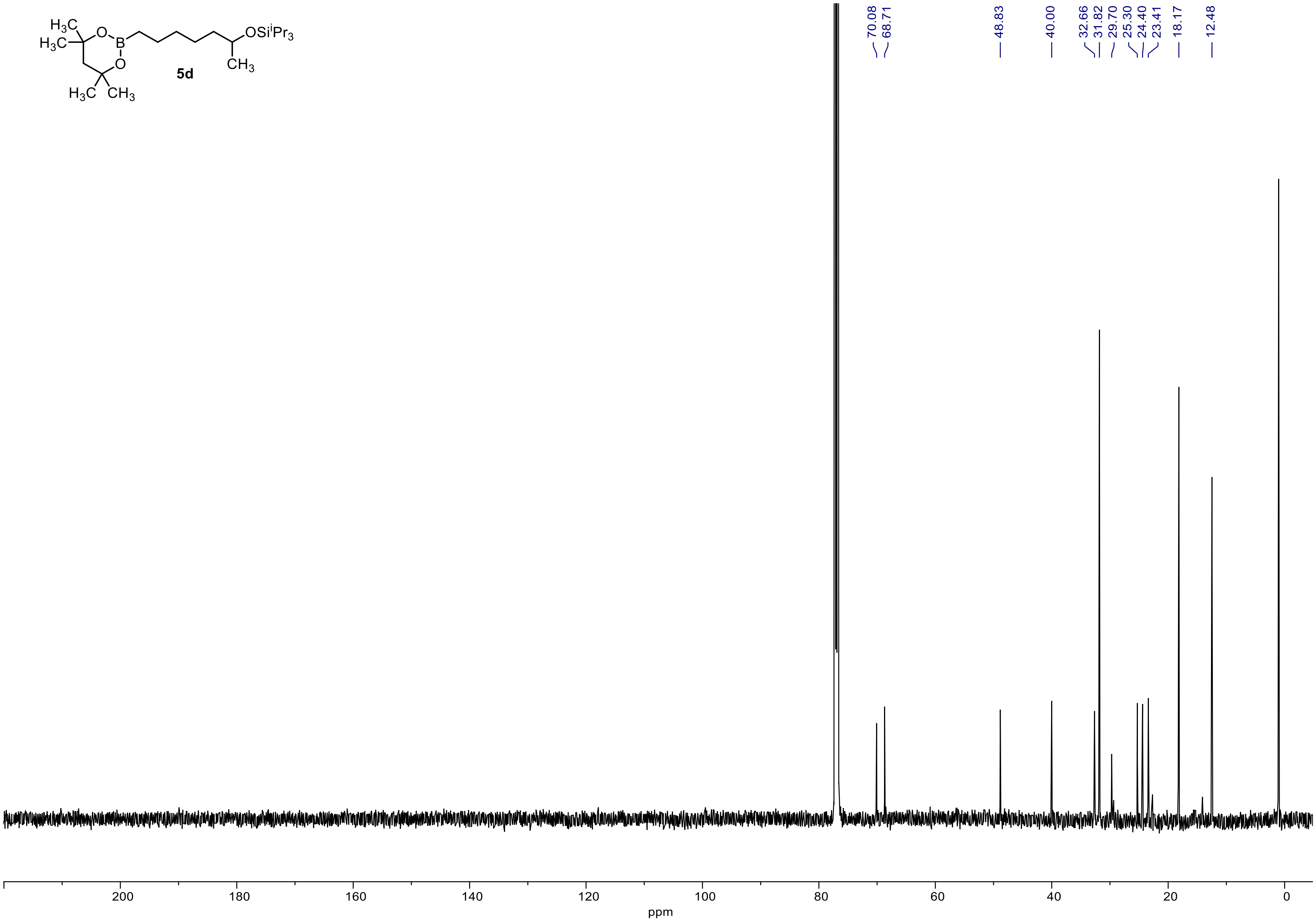

Figure S56. $^{13}C\{^{1}H\}$ NMR Spectrum of **5d** (150 MHz, $CDCl_3$).

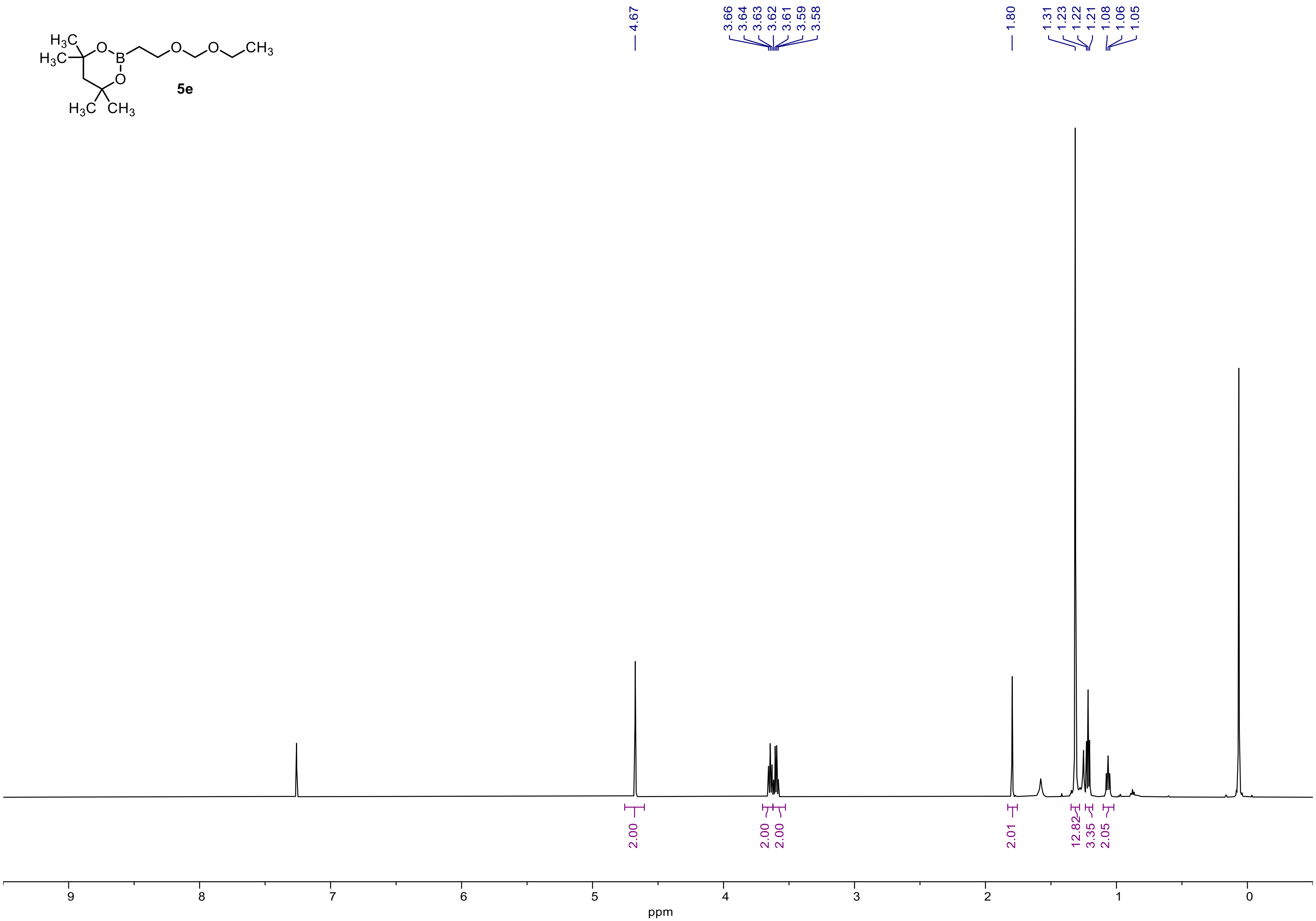

Figure S57. ^{1}H NMR Spectrum of **5e** (600 MHz, $CDCl_3$).

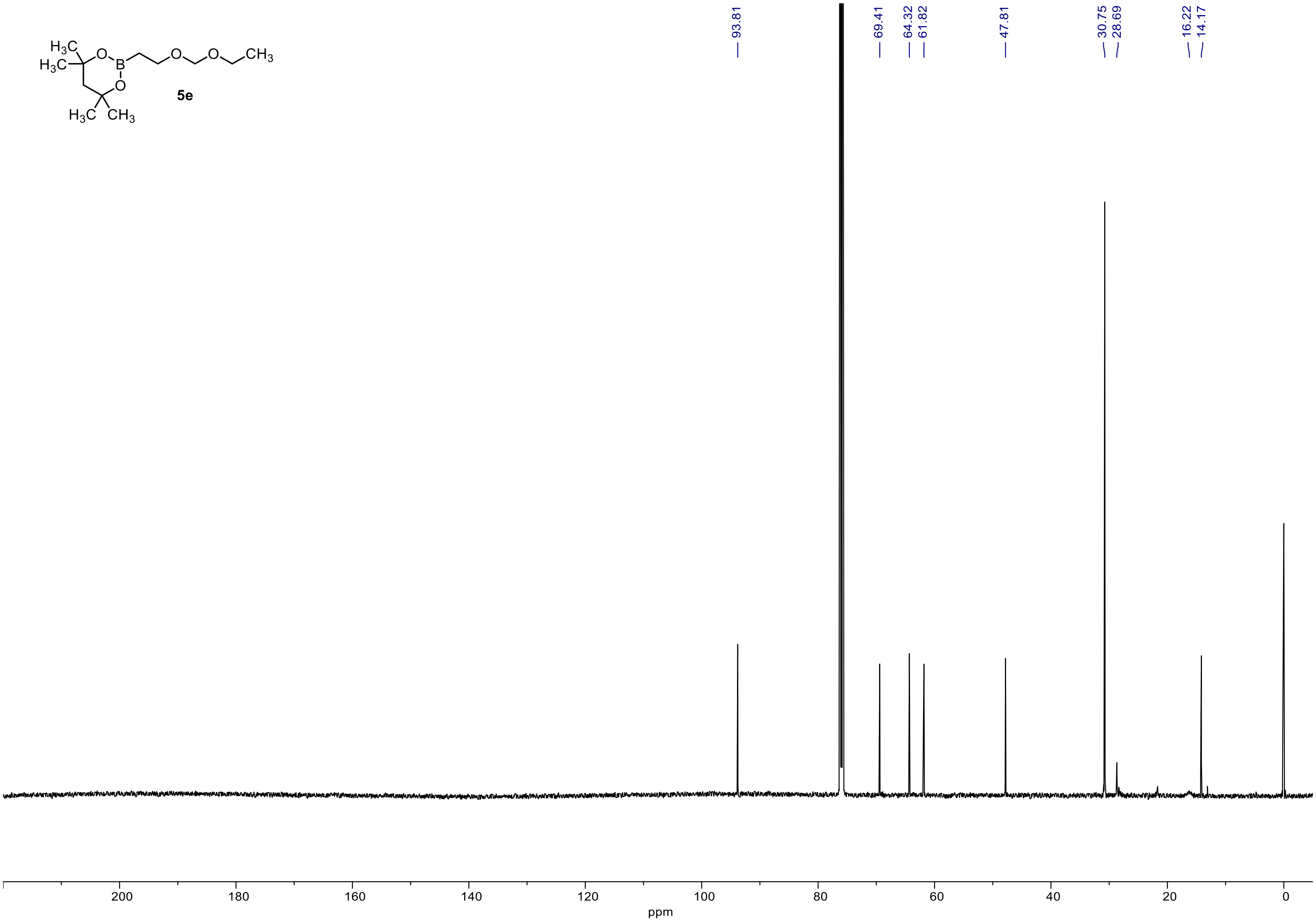

Figure S58. $^{13}C\{^{1}H\}$ NMR Spectrum of **5e** (150 MHz, $CDCl_3$).

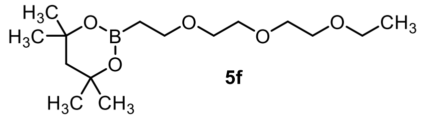

5f

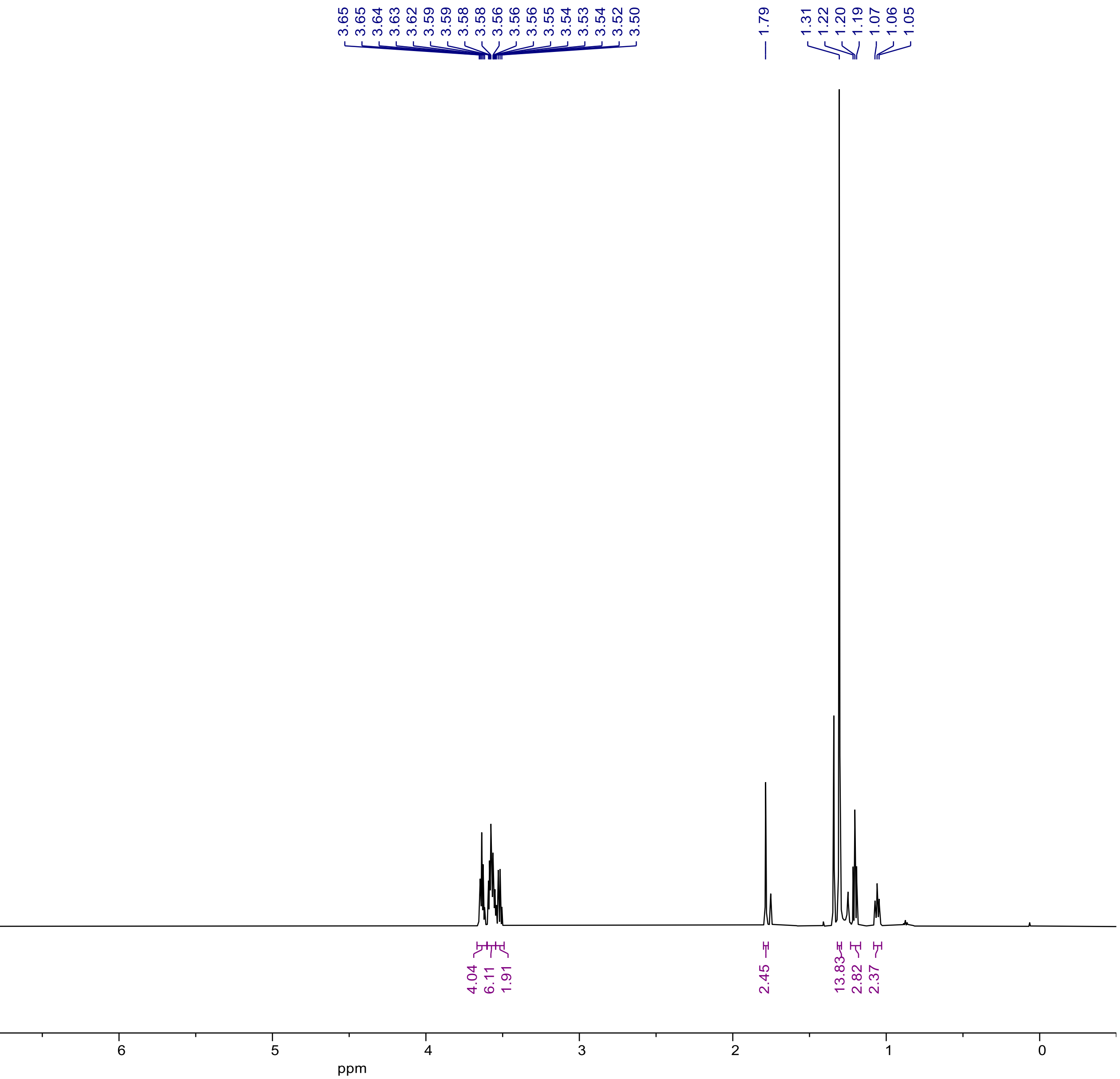

Figure S59. ^{1}H NMR Spectrum of **5f** (600 MHz, $CDCl_3$).

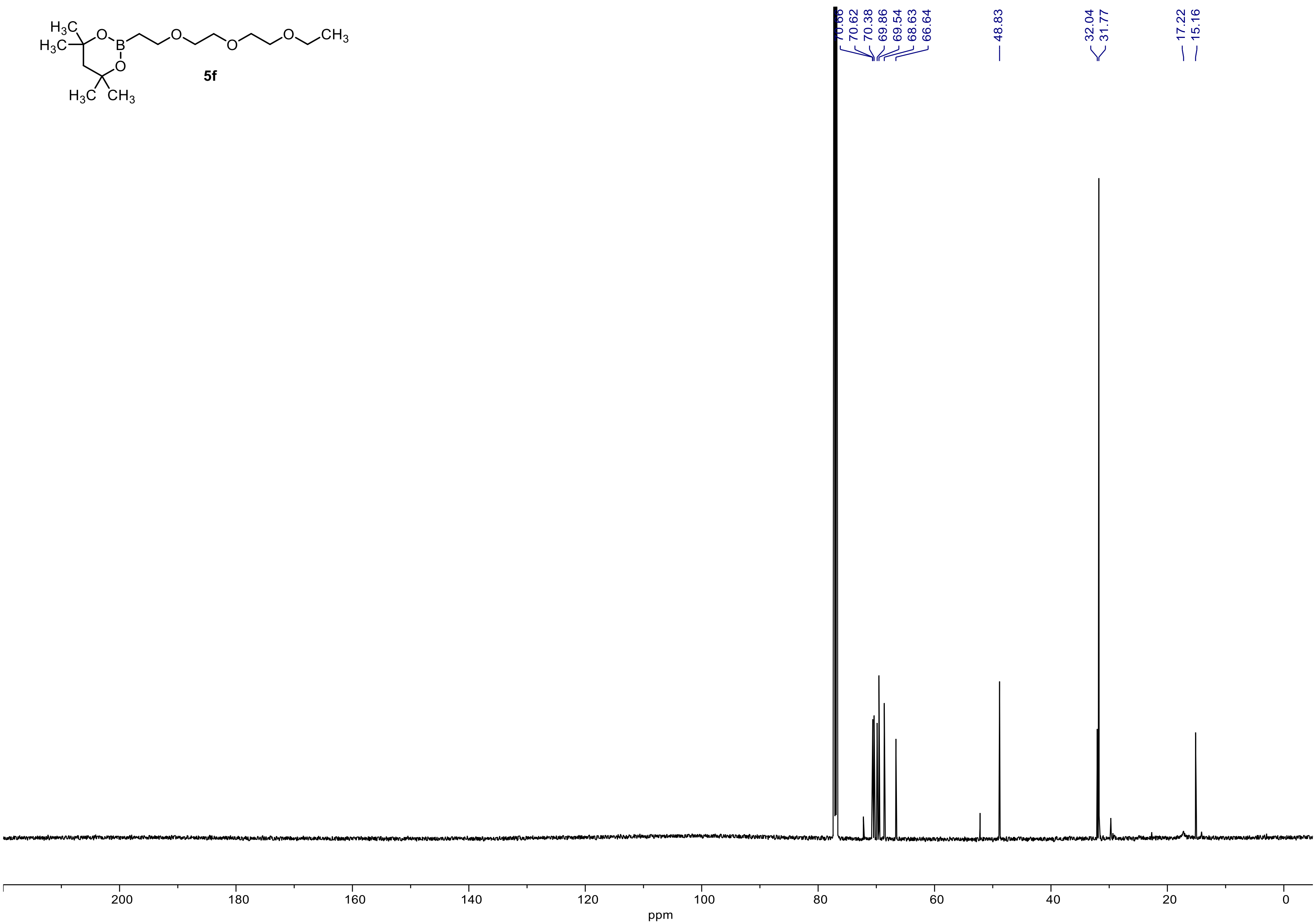

Figure S60. $^{13}C\{^{1}H\}$ NMR Spectrum of **5f** (150 MHz, $CDCl_3$).

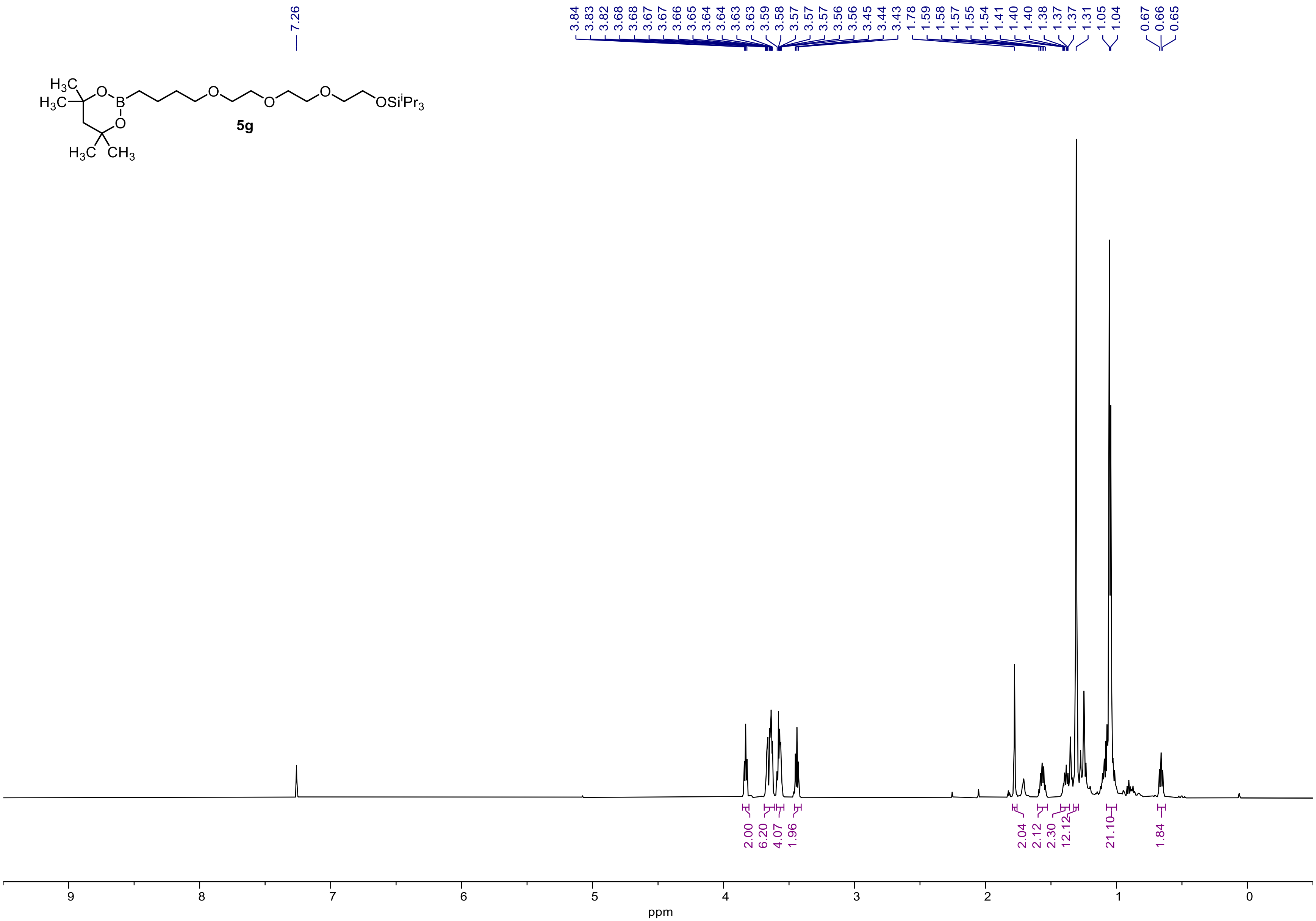

Figure S61. ^{1}H NMR Spectrum of **5g** (600 MHz, $CDCl_3$).

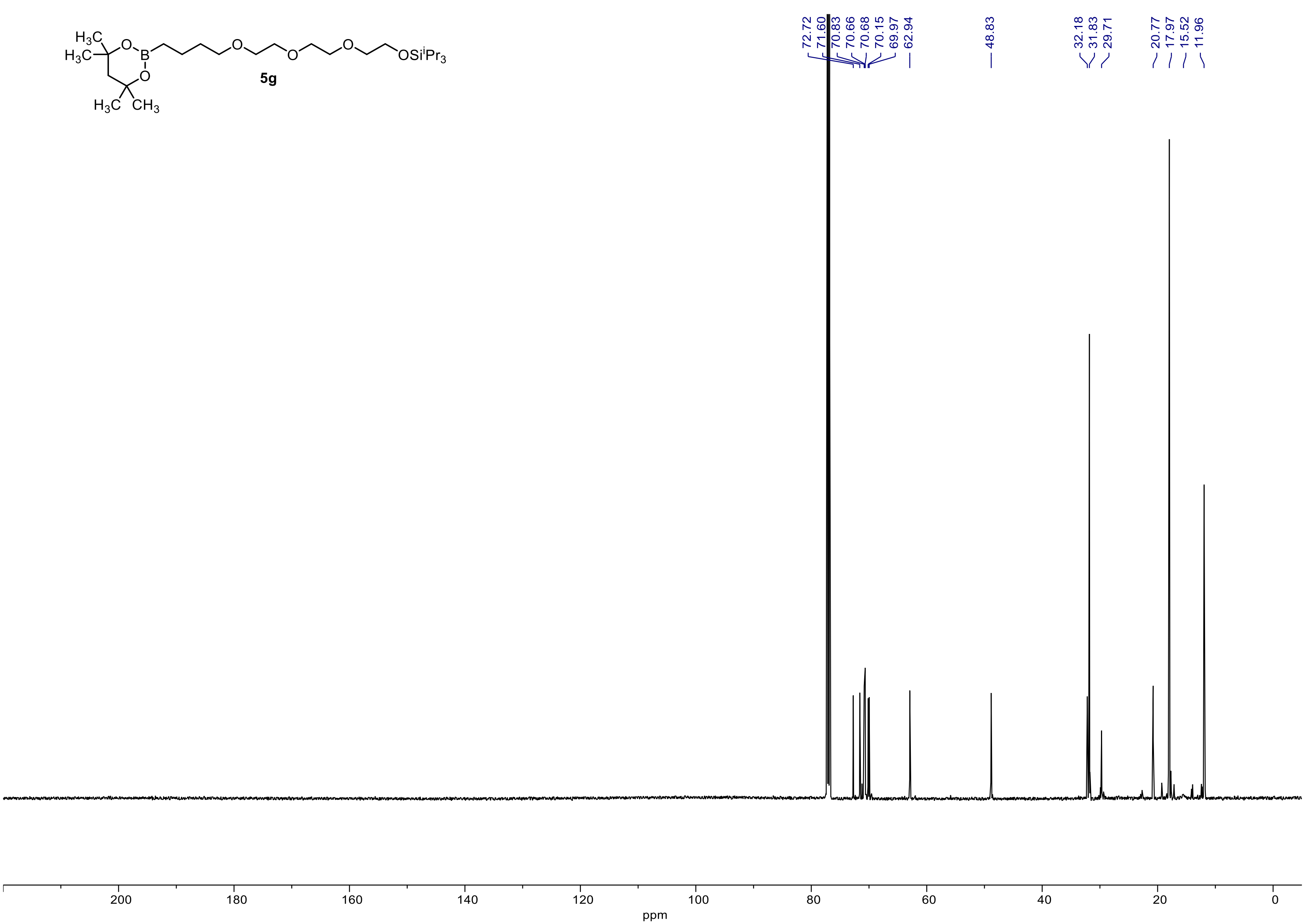

Figure S62. $^{13}C\{^1H\}$ NMR Spectrum of **5g** (150 MHz, $CDCl_3$).

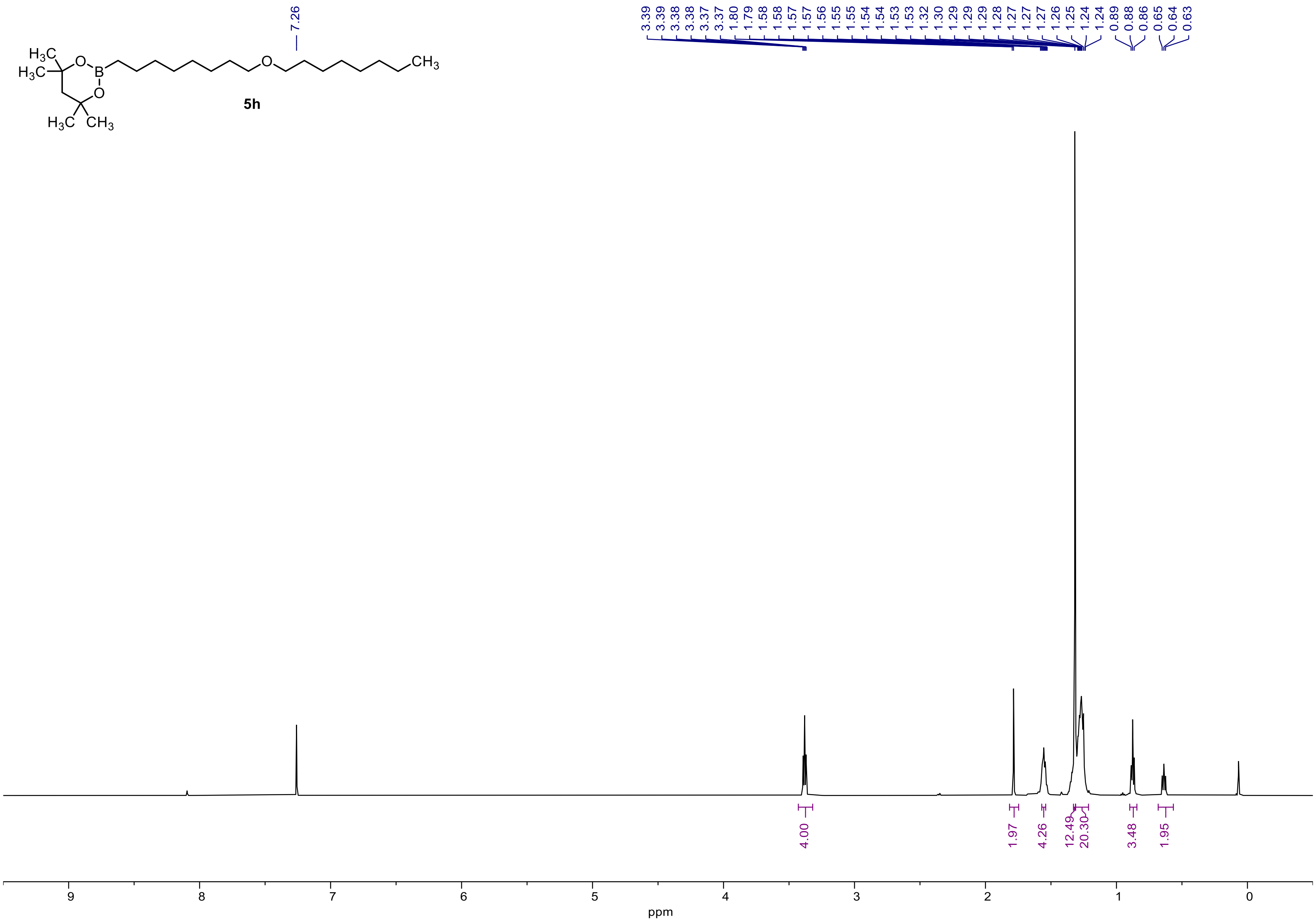

Figure S63. ^{1}H NMR Spectrum of **5h** (600 MHz, $CDCl_3$).

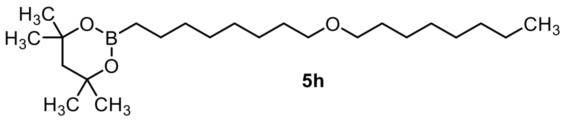

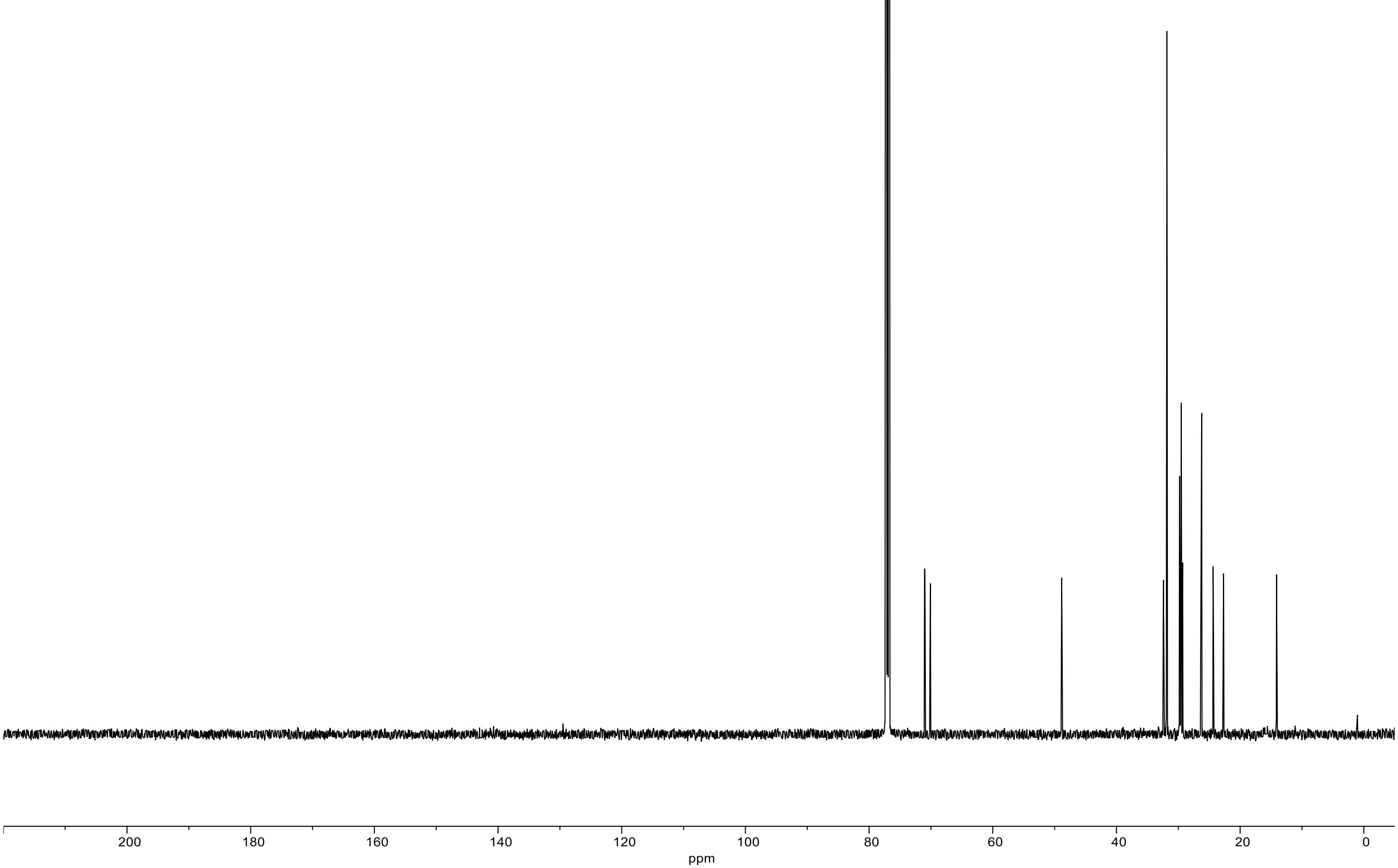

Figure S64. $^{13}C\{^{1}H\}$ NMR Spectrum of **5h** (150 MHz, $CDCl_3$).

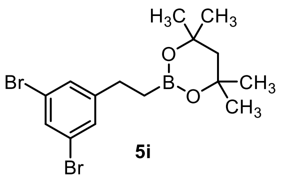

5i

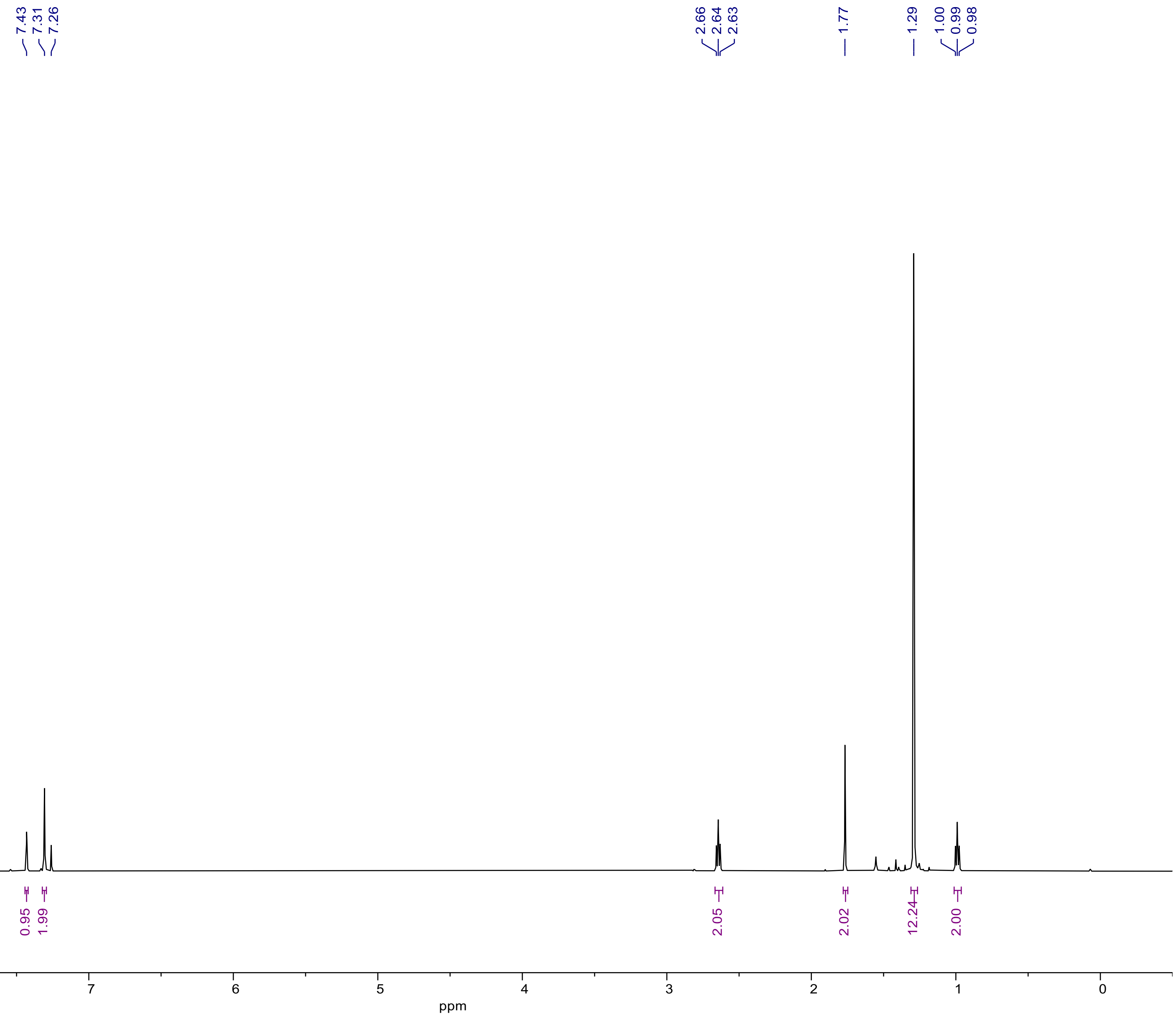

Figure S65. ^{1}H NMR Spectrum of **5i** (600 MHz, CDCl3).

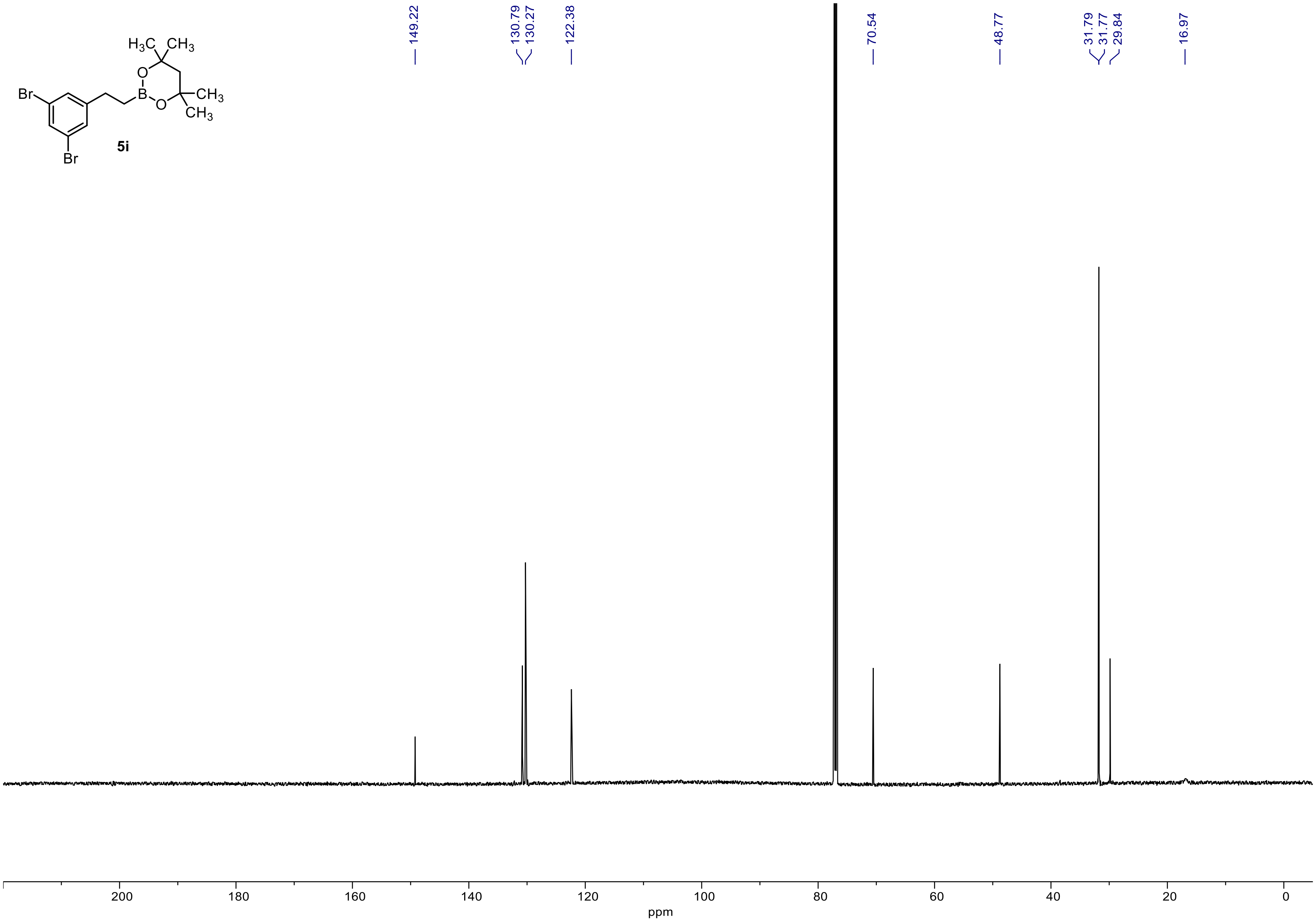

Figure S66. $^{13}C\{^{1}H\}$ NMR Spectrum of **5i** (150 MHz, $CDCl_3$).

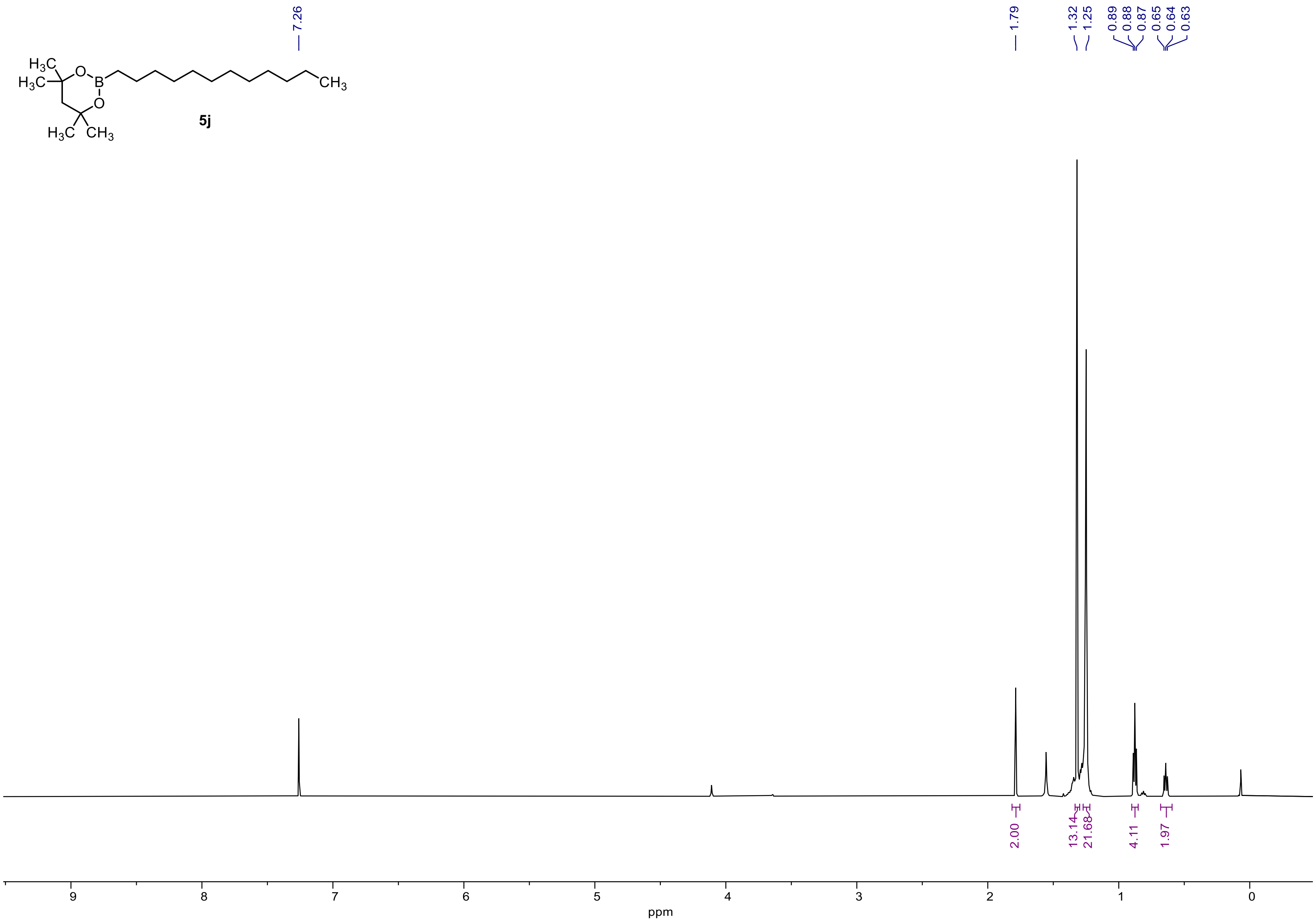

Figure S67. ^{1}H NMR Spectrum of **5j** (600 MHz, $CDCl_3$).

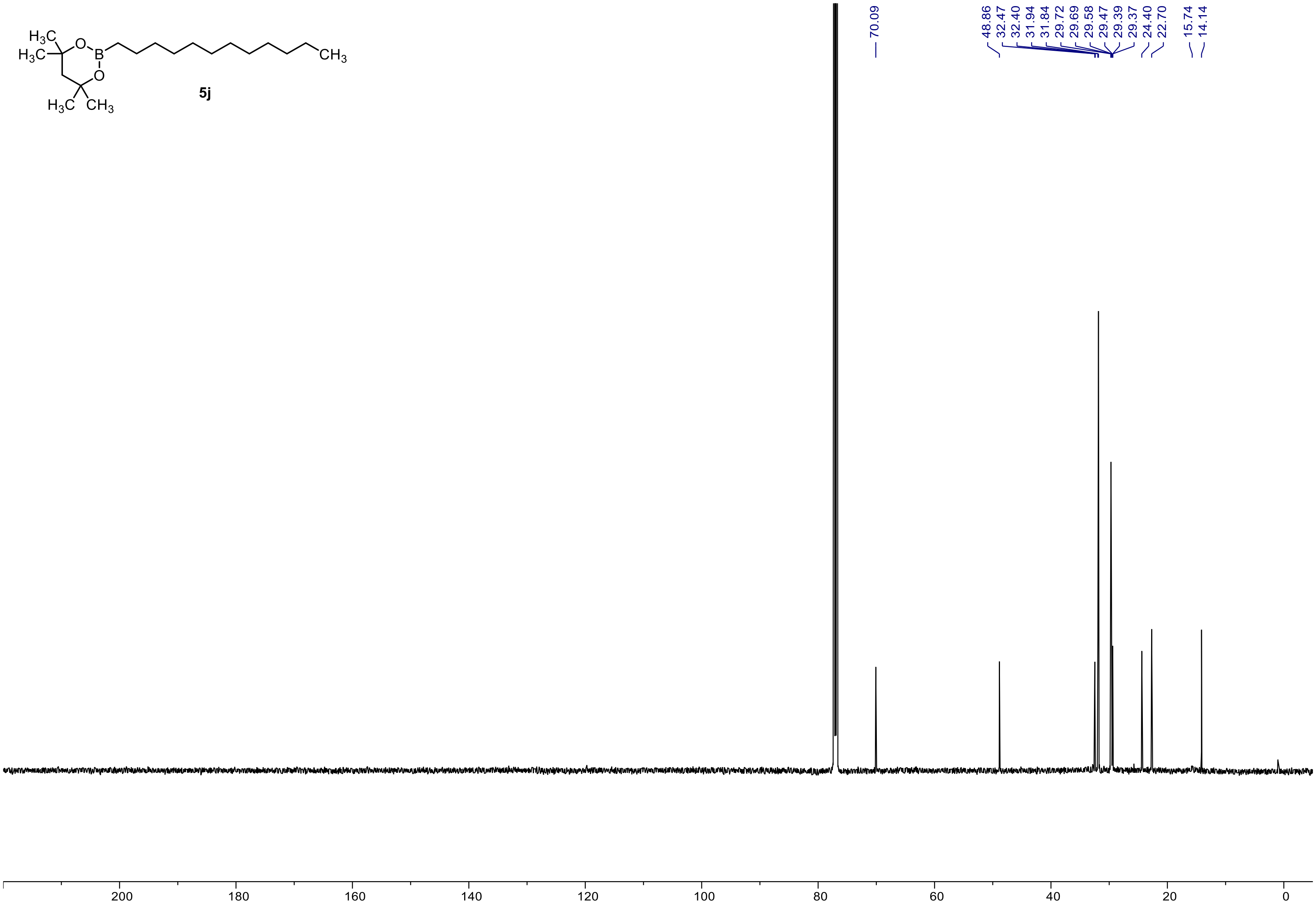

Figure S68. $^{13}C\{^1H\}$ NMR Spectrum of **5j** (150 MHz, $CDCl_3$).

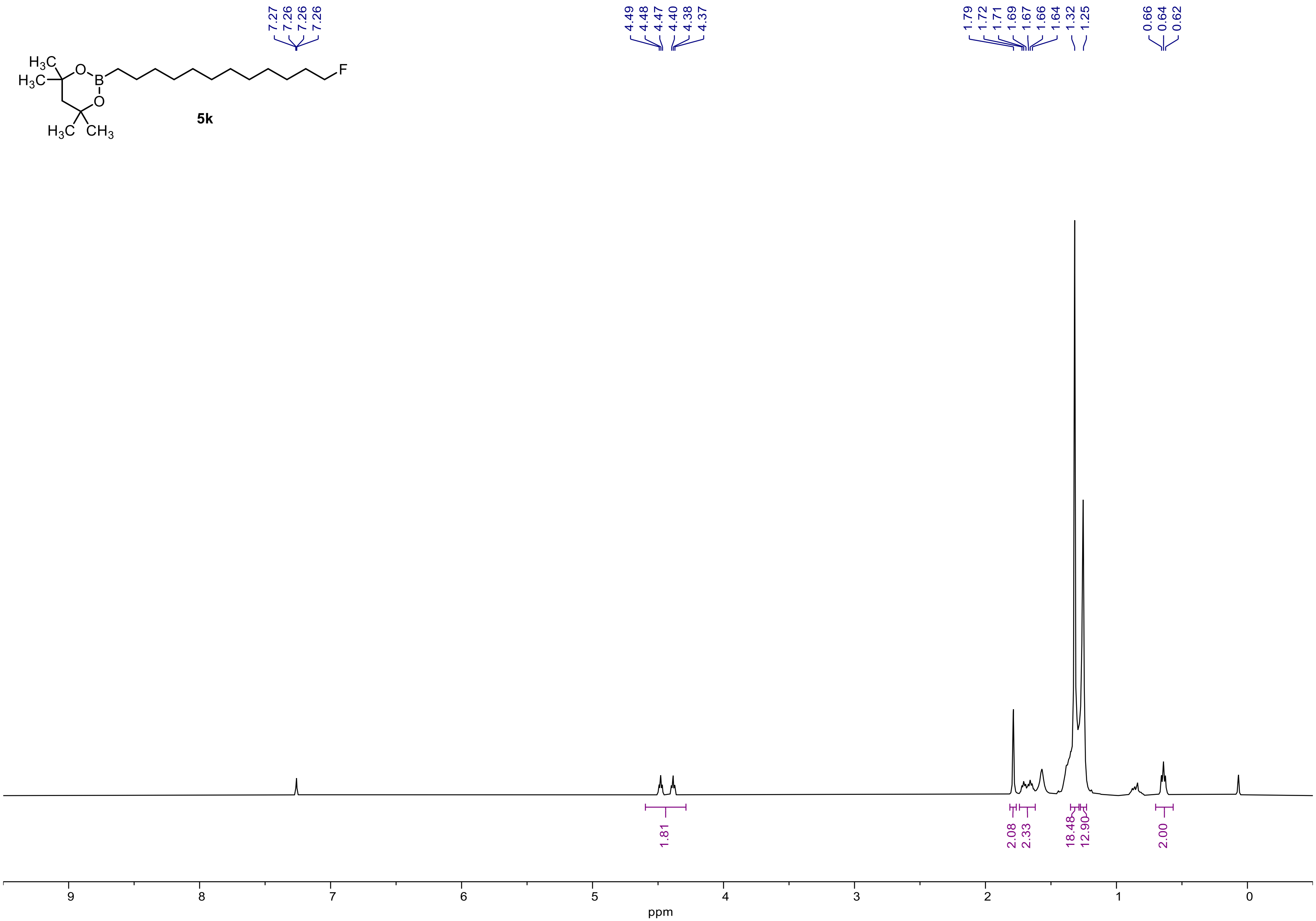

Figure S69. ^{1}H NMR Spectrum of **5k** (600 MHz, $CDCl_3$).

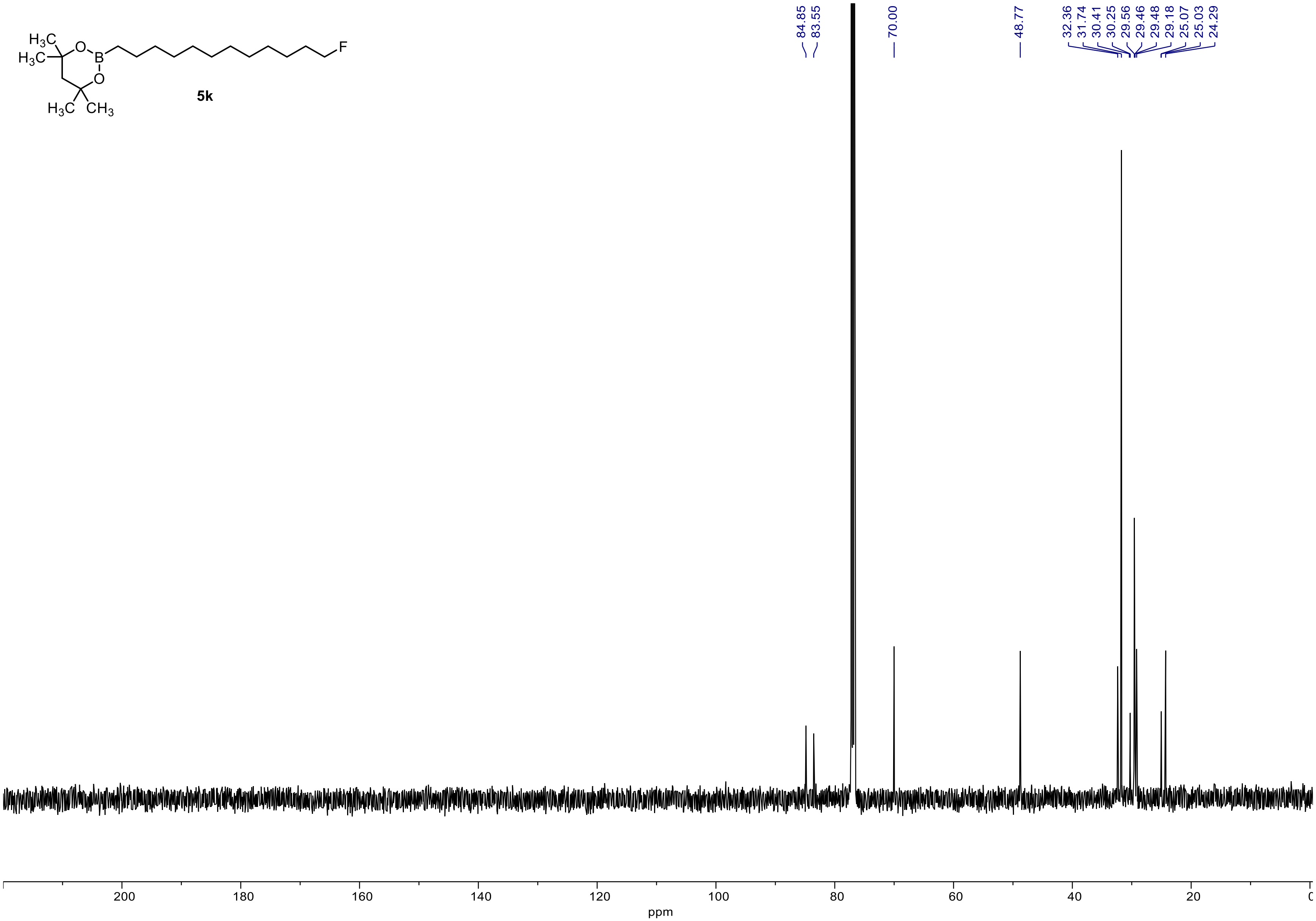

Figure S70. $^{13}C\{^{1}H\}$ NMR Spectrum of **5k** (150 MHz, $CDCl_3$).

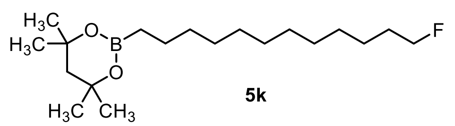

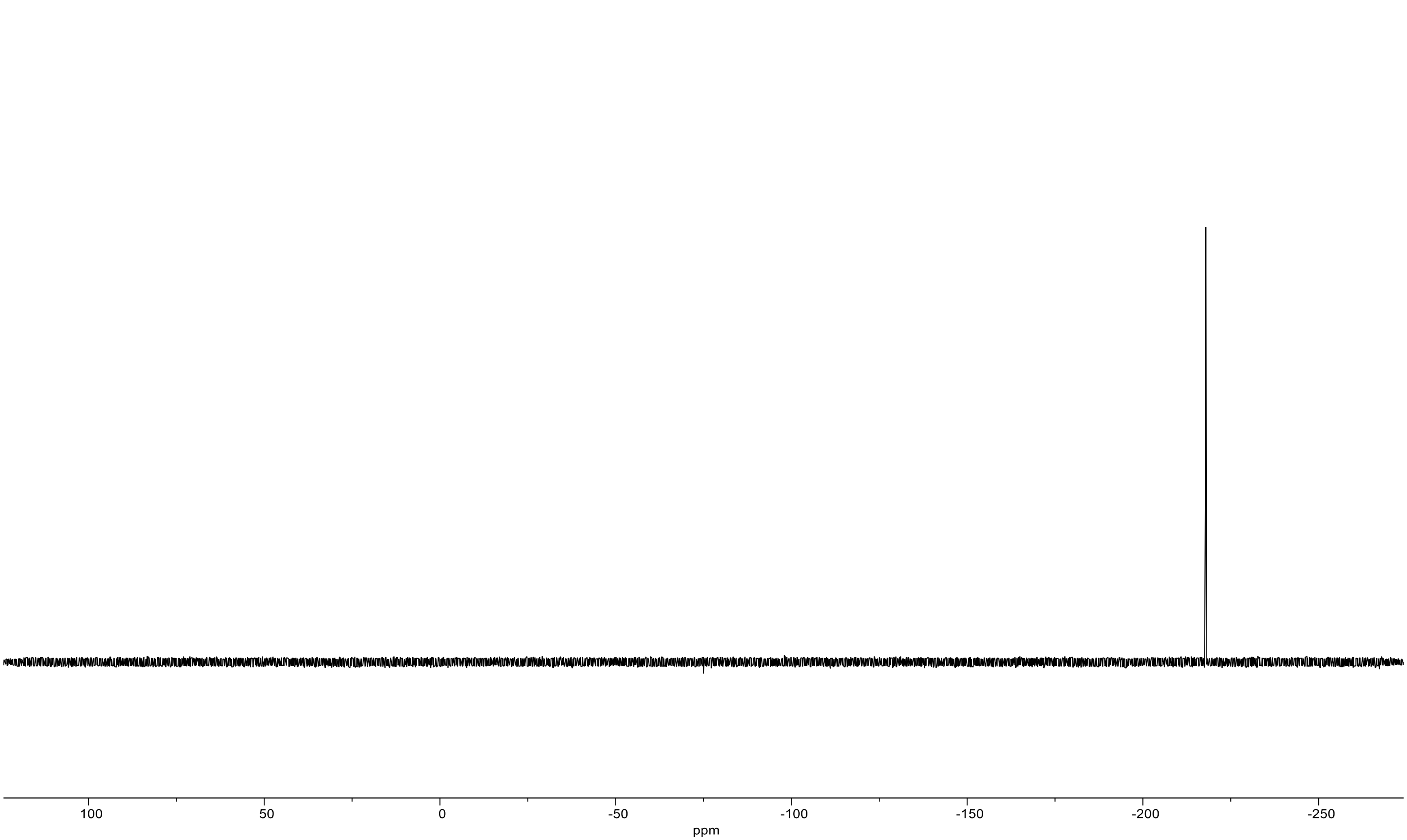

Figure S71. ^{19}F NMR Spectrum of **5k** (471 MHz, $CDCl_3$).

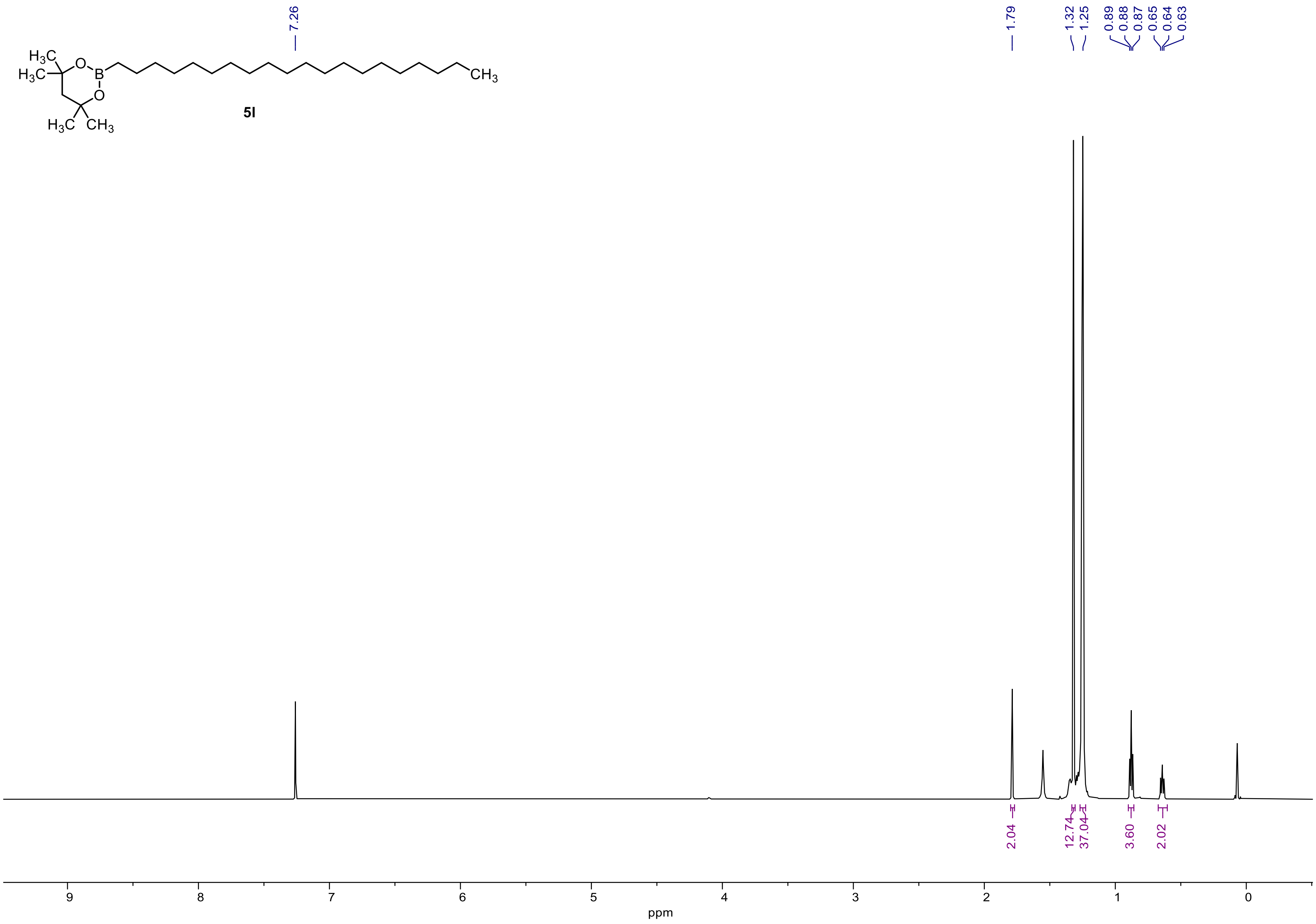

Figure S72. ^{1}H NMR Spectrum of **5l** (600 MHz, $CDCl_3$).

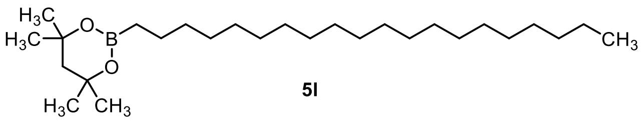

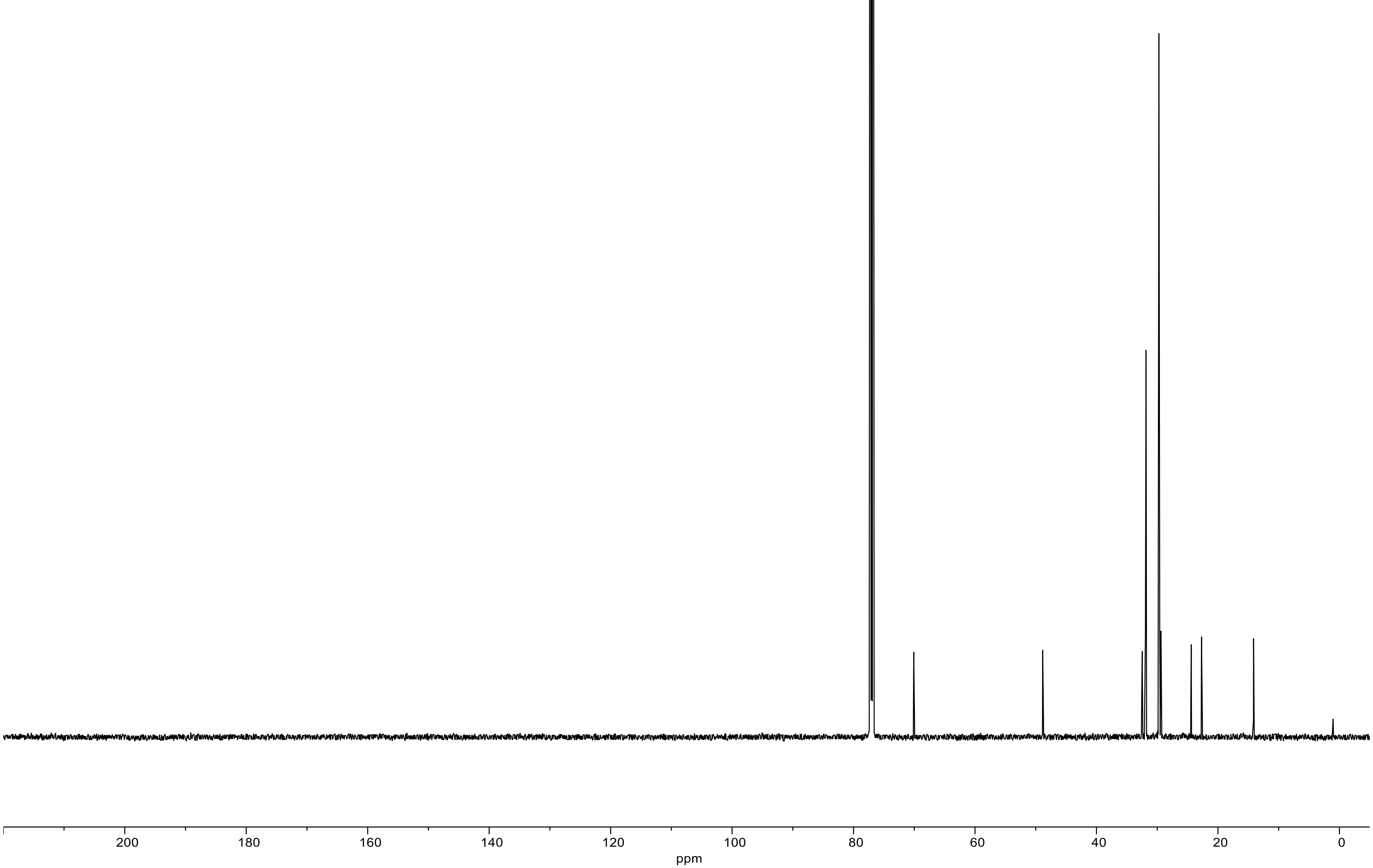

Figure S73. $^{13}C\{^{1}H\}$ NMR Spectrum of **5l** (150 MHz, $CDCl_3$).

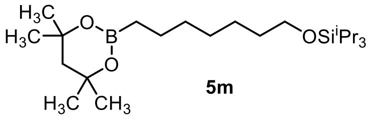

5m

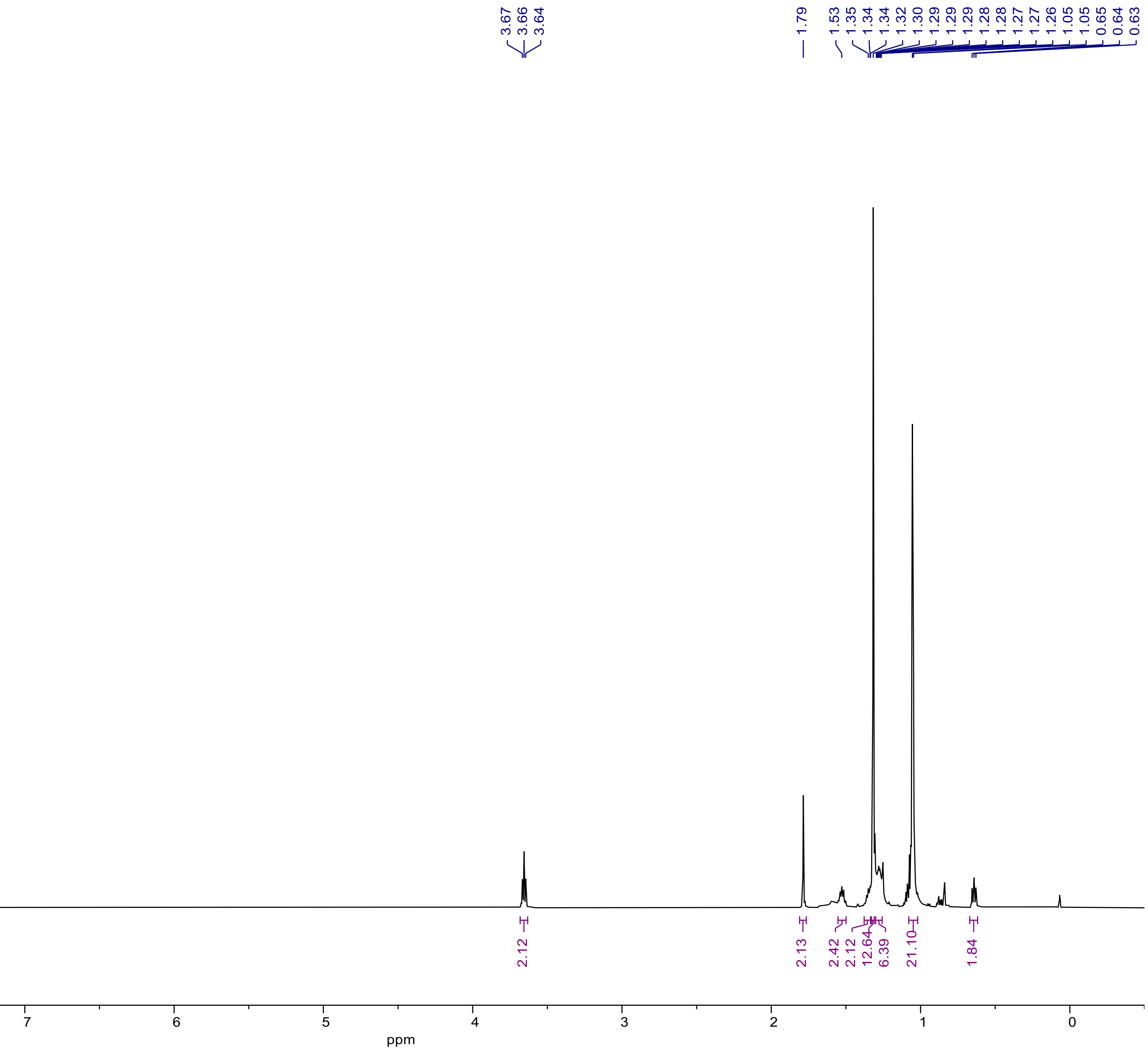

Figure S74. ^{1}H NMR Spectrum of **5m** (600 MHz, $CDCl_3$).

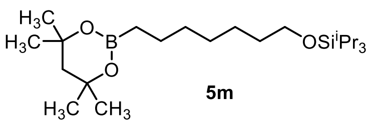

5m

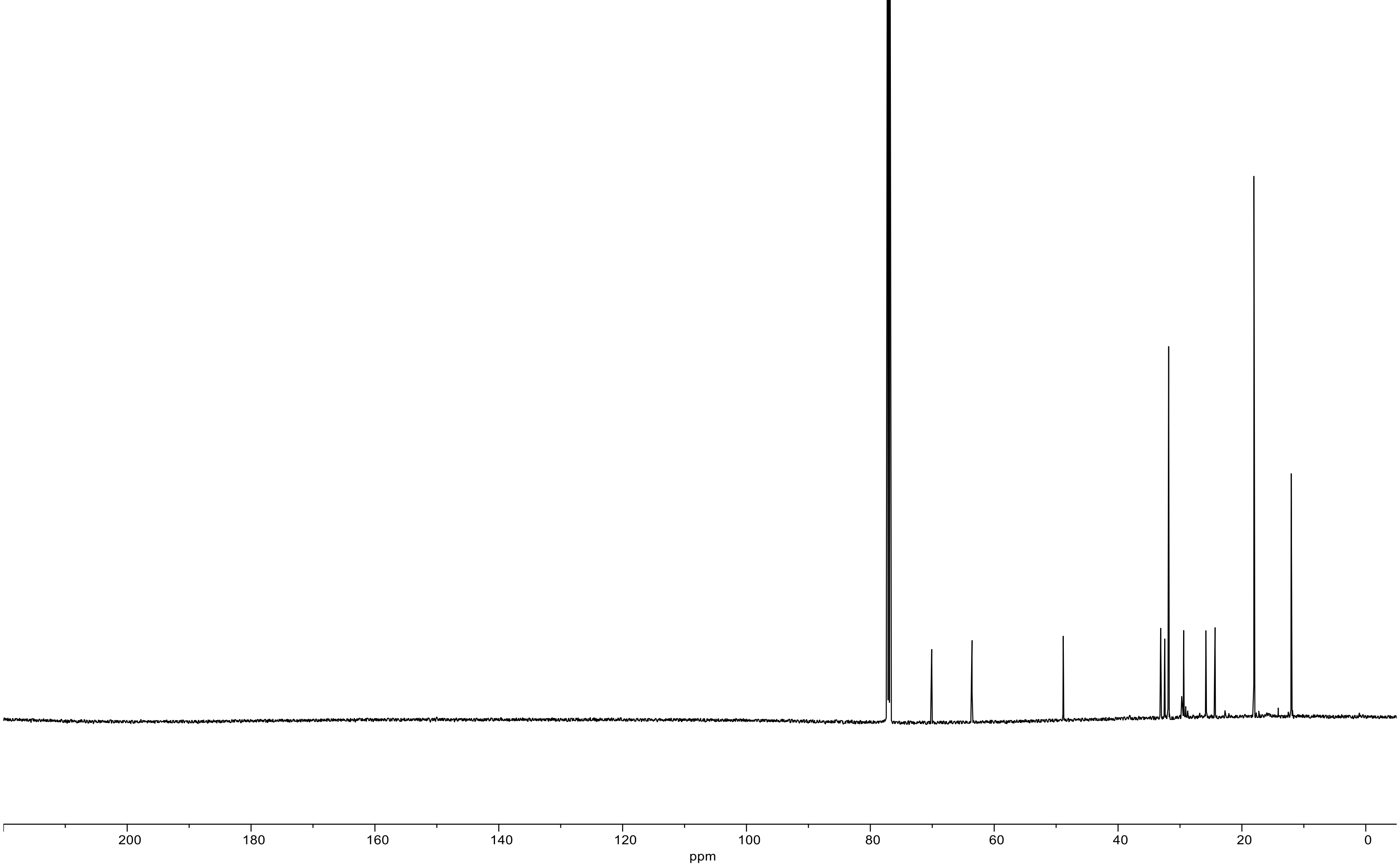

Figure S75. $^{13}C\{^{1}H\}$ NMR Spectrum of **5m** (150 MHz, $CDCl_3$).

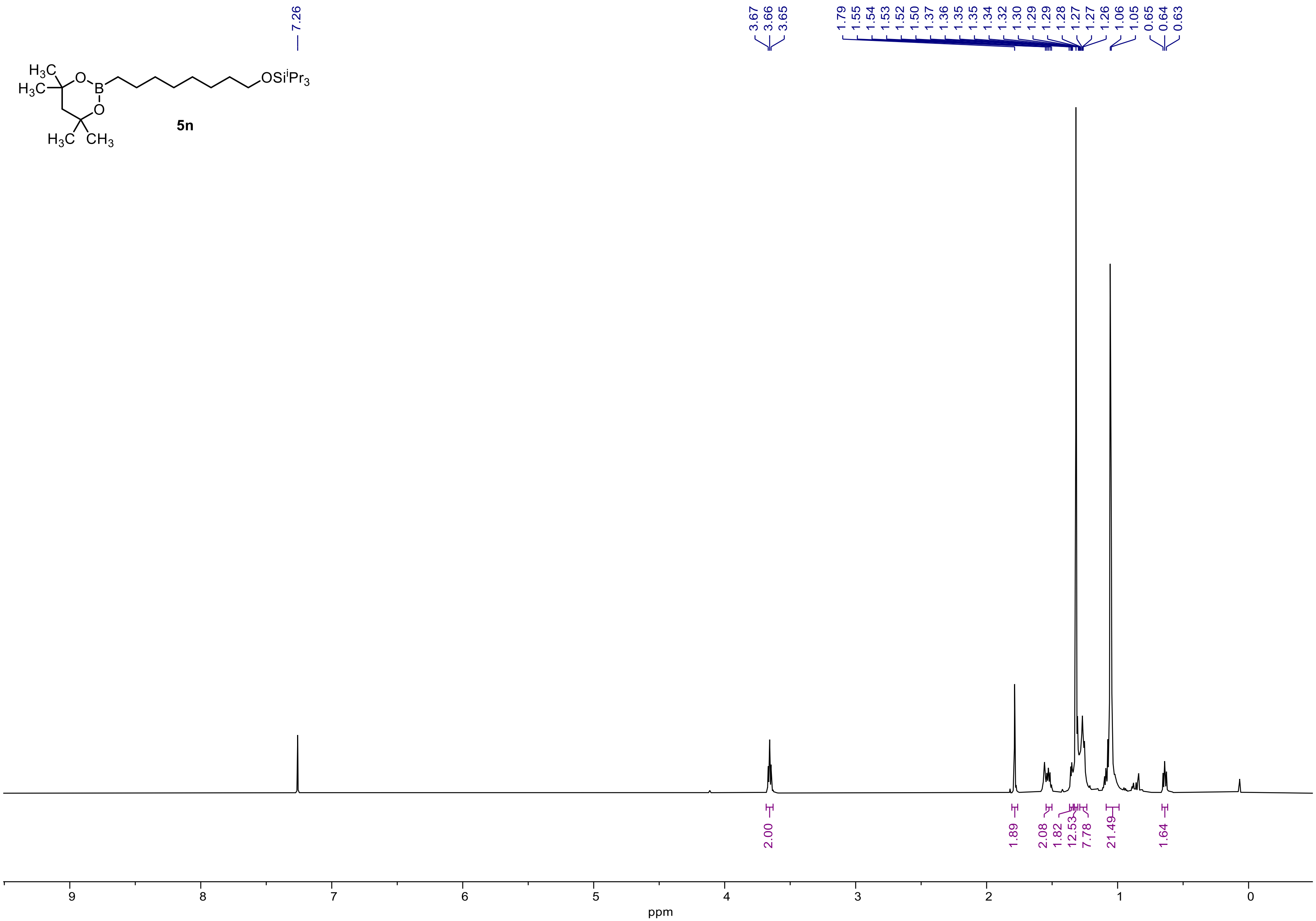

Figure S76. ^{1}H NMR Spectrum of **5n** (600 MHz, $CDCl_3$).

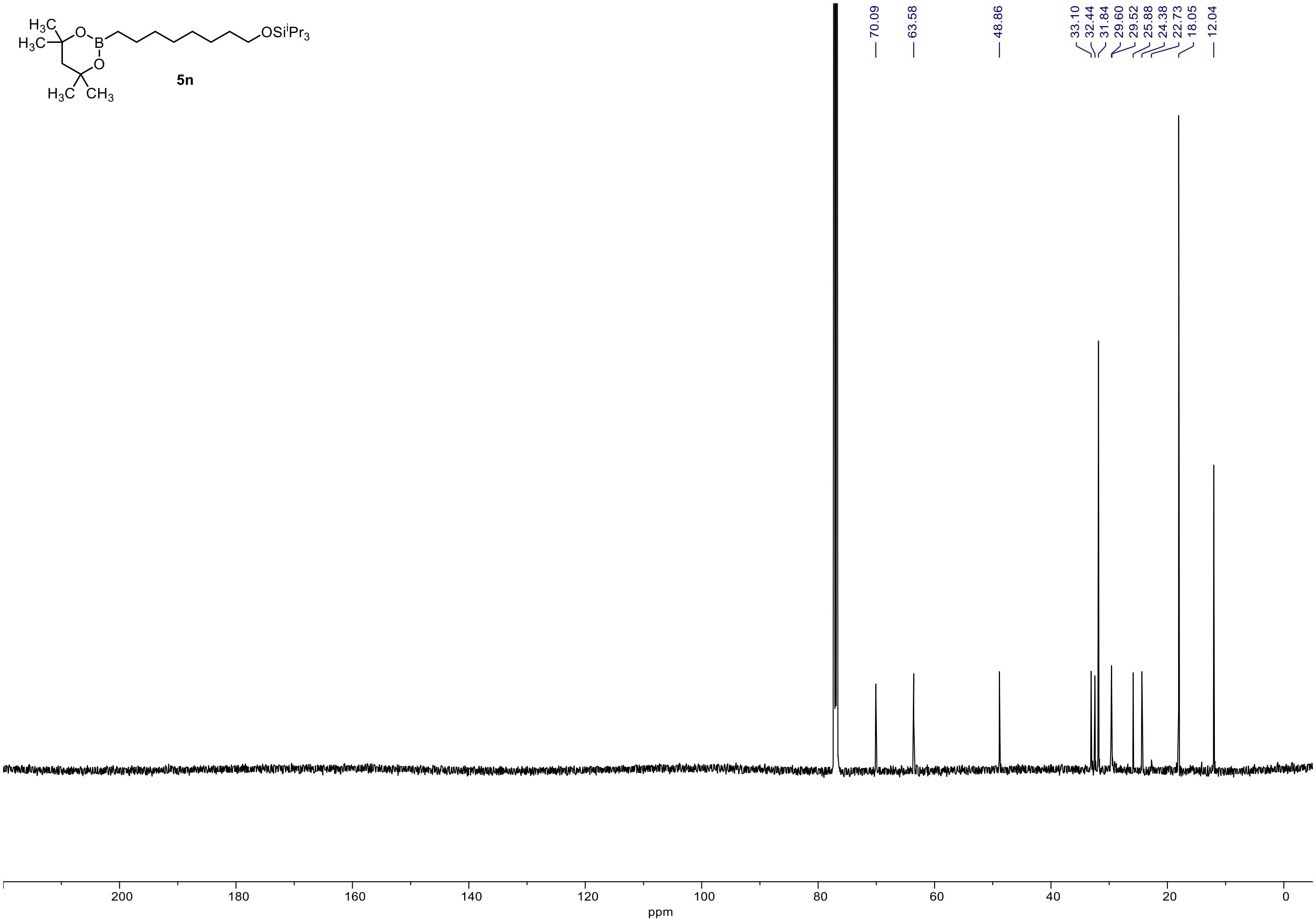

Figure S77. $^{13}C\{^1H\}$ NMR Spectrum of **5n** (150 MHz, $CDCl_3$).

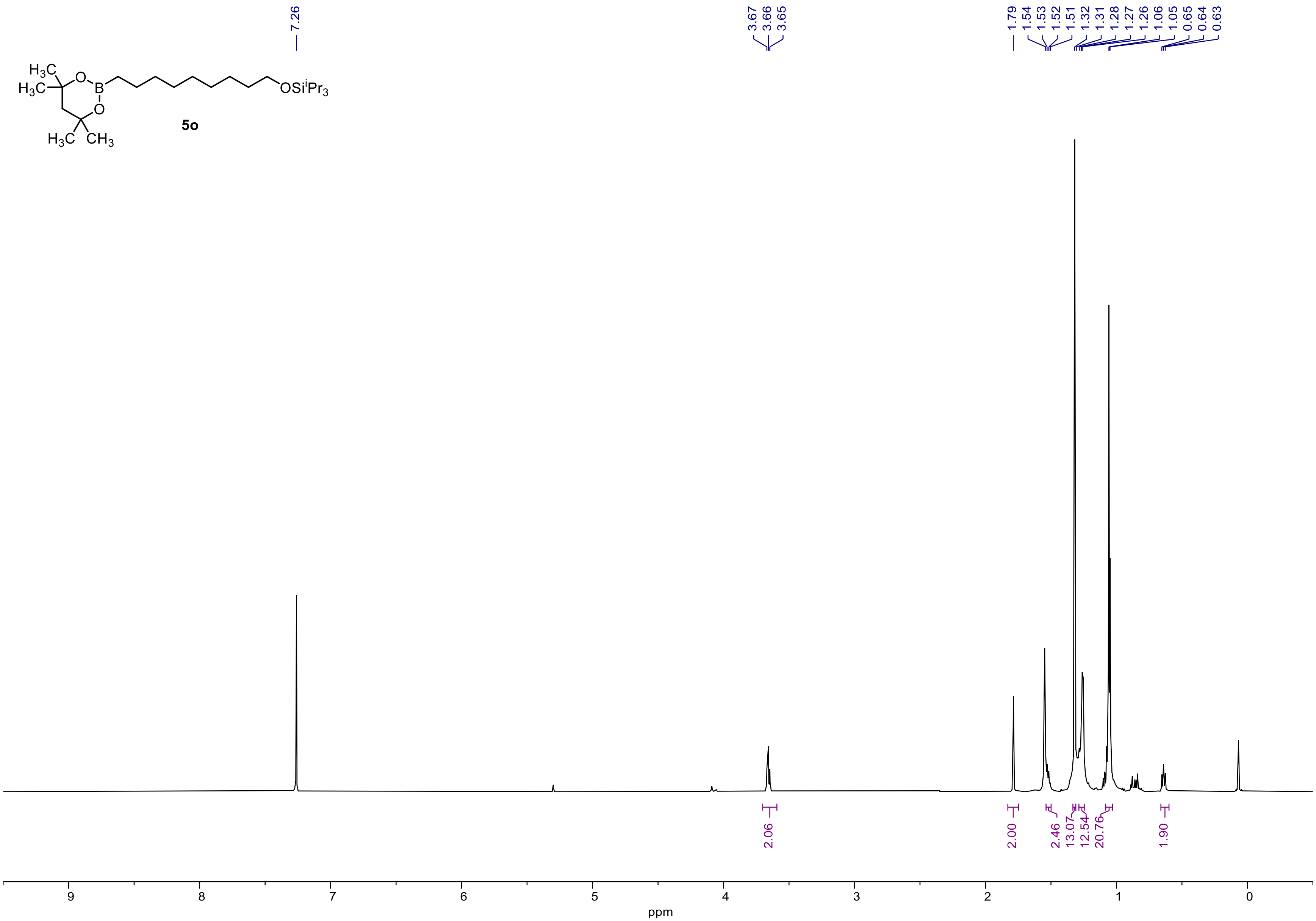

Figure S78. ^{1}H NMR Spectrum of **5o** (600 MHz, $CDCl_3$).

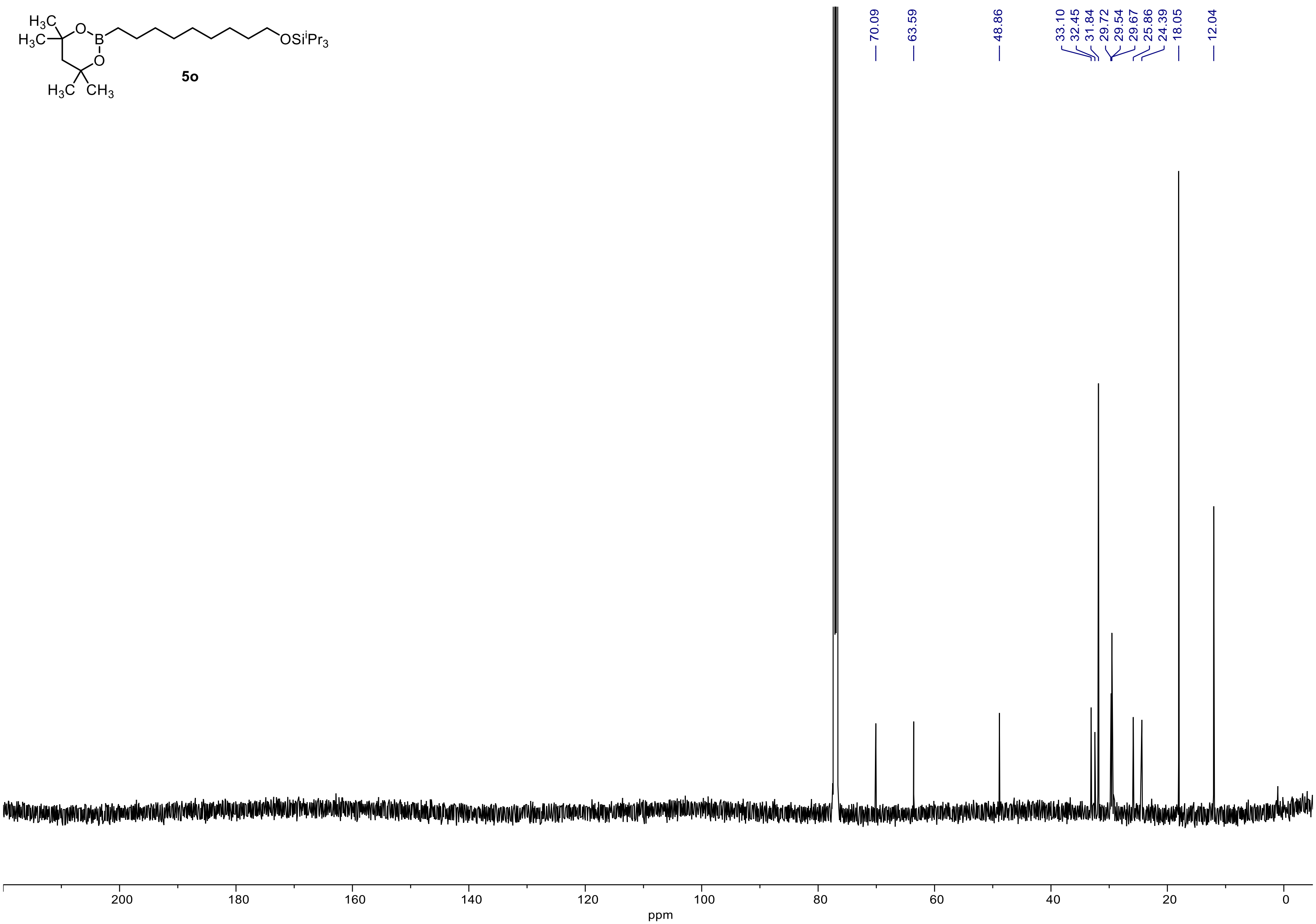

Figure S79. $^{13}C\{^1H\}$ NMR Spectrum of **5o** (150 MHz, $CDCl_3$).

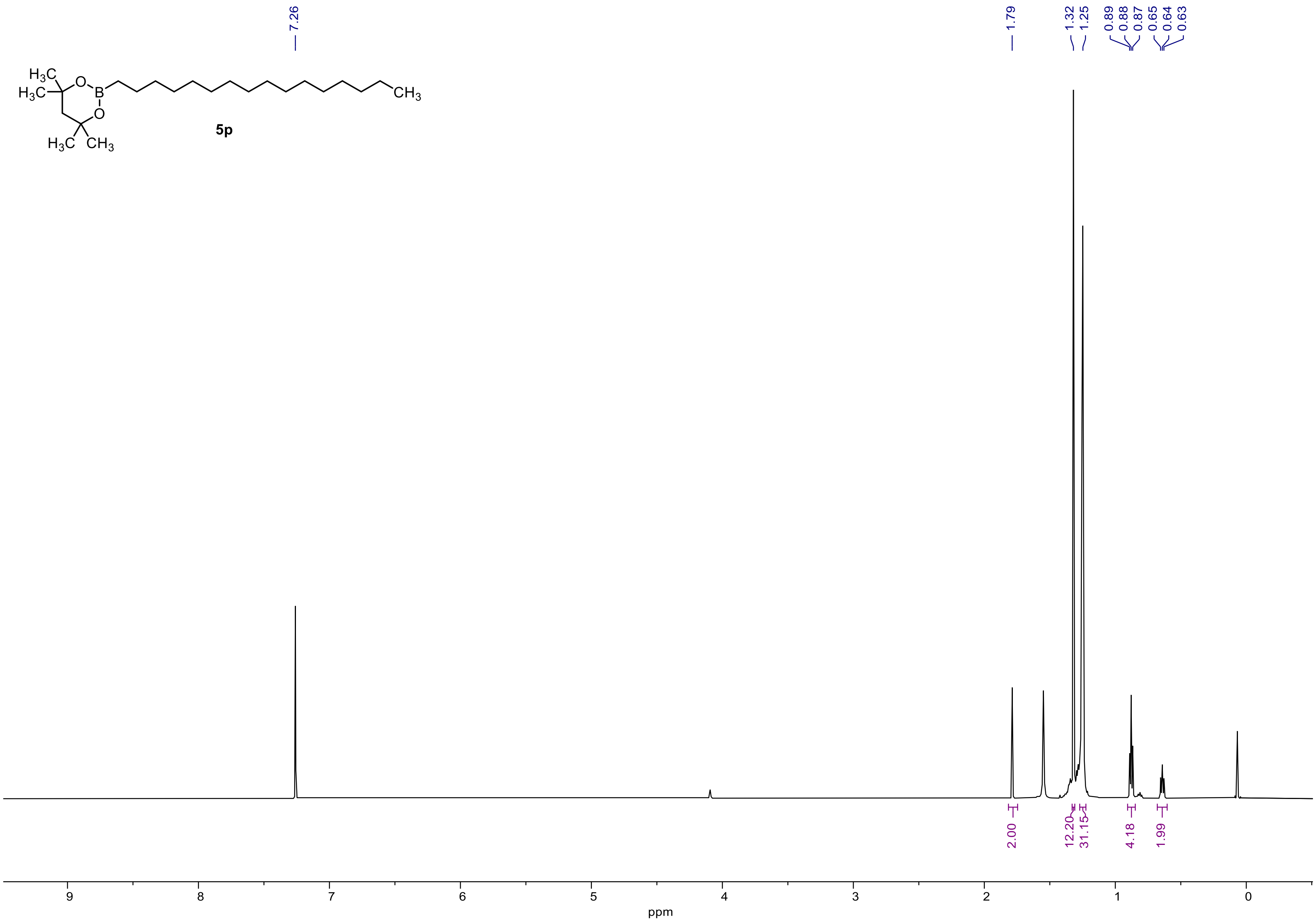

Figure S80. ^{1}H NMR Spectrum of **5p** (600 MHz, $CDCl_3$).

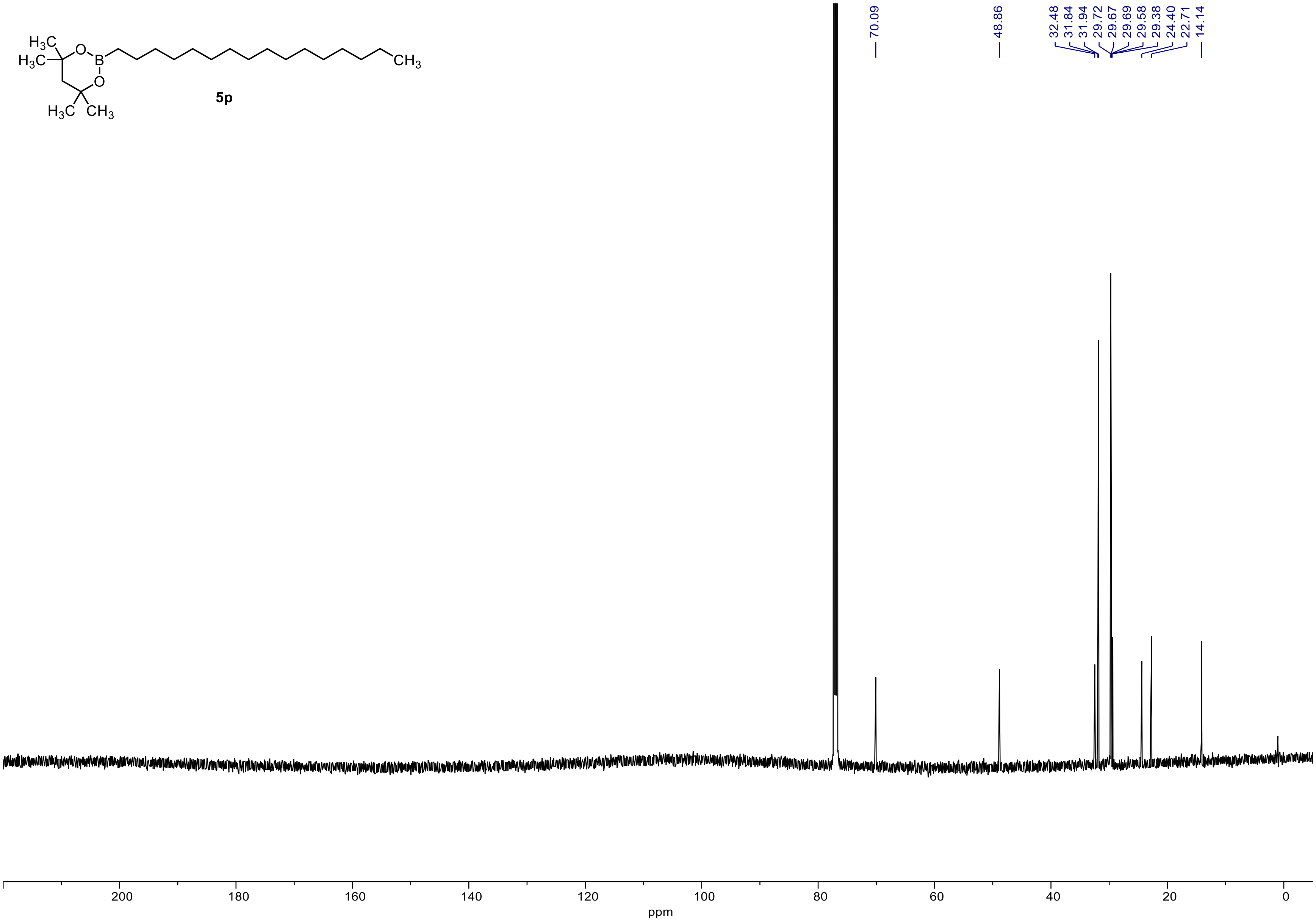

Figure S81. $^{13}C\{^{1}H\}$ NMR Spectrum of **5p** (150 MHz, $CDCl_3$).

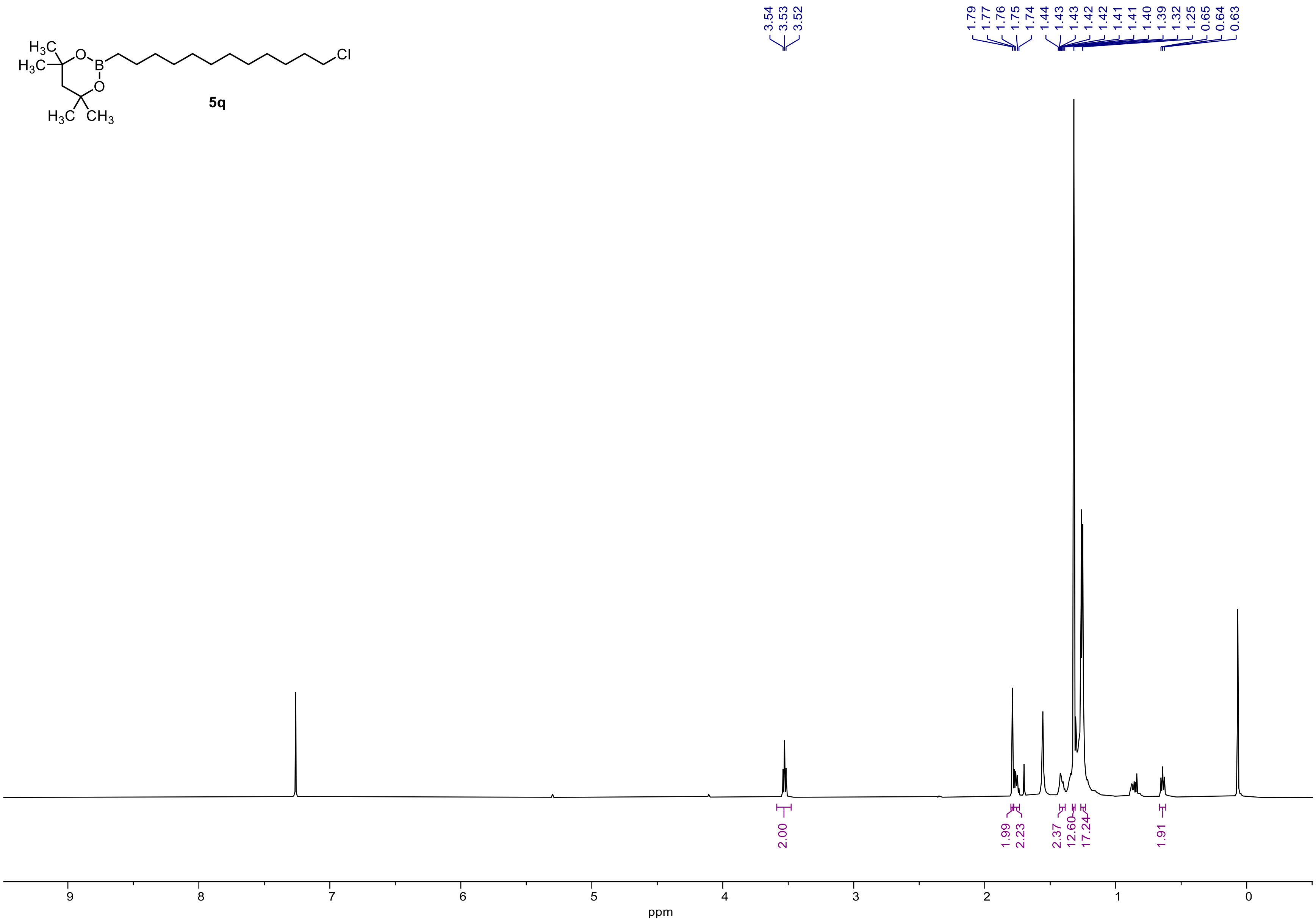

Figure S82. ^{1}H NMR Spectrum of **5q** (600 MHz, $CDCl_3$).

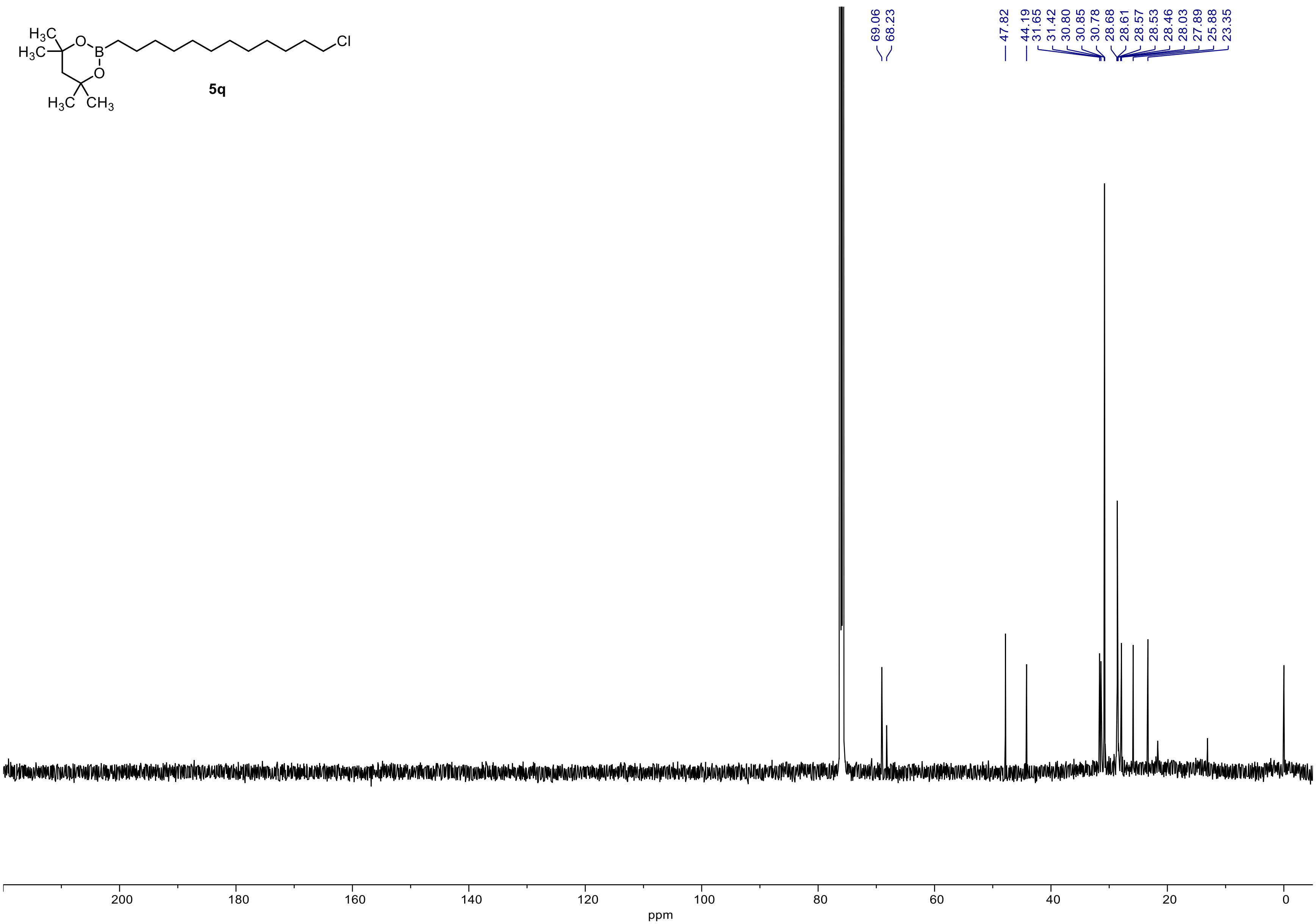

Figure S83. $^{13}C\{^1H\}$ NMR Spectrum of **5q** (150 MHz, $CDCl_3$).

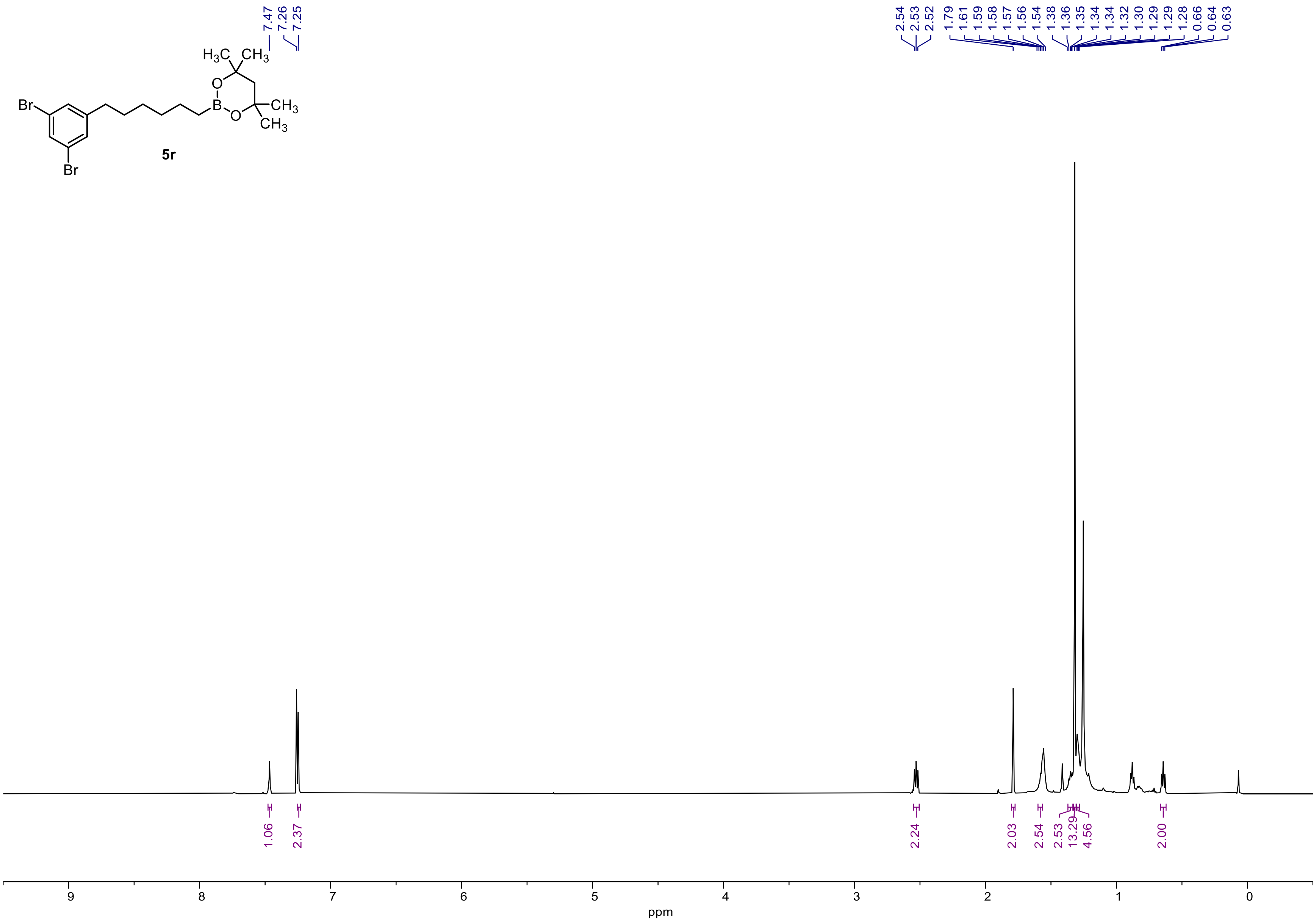

Figure S84. ^{1}H NMR Spectrum of **5r** (600 MHz, $CDCl_3$).

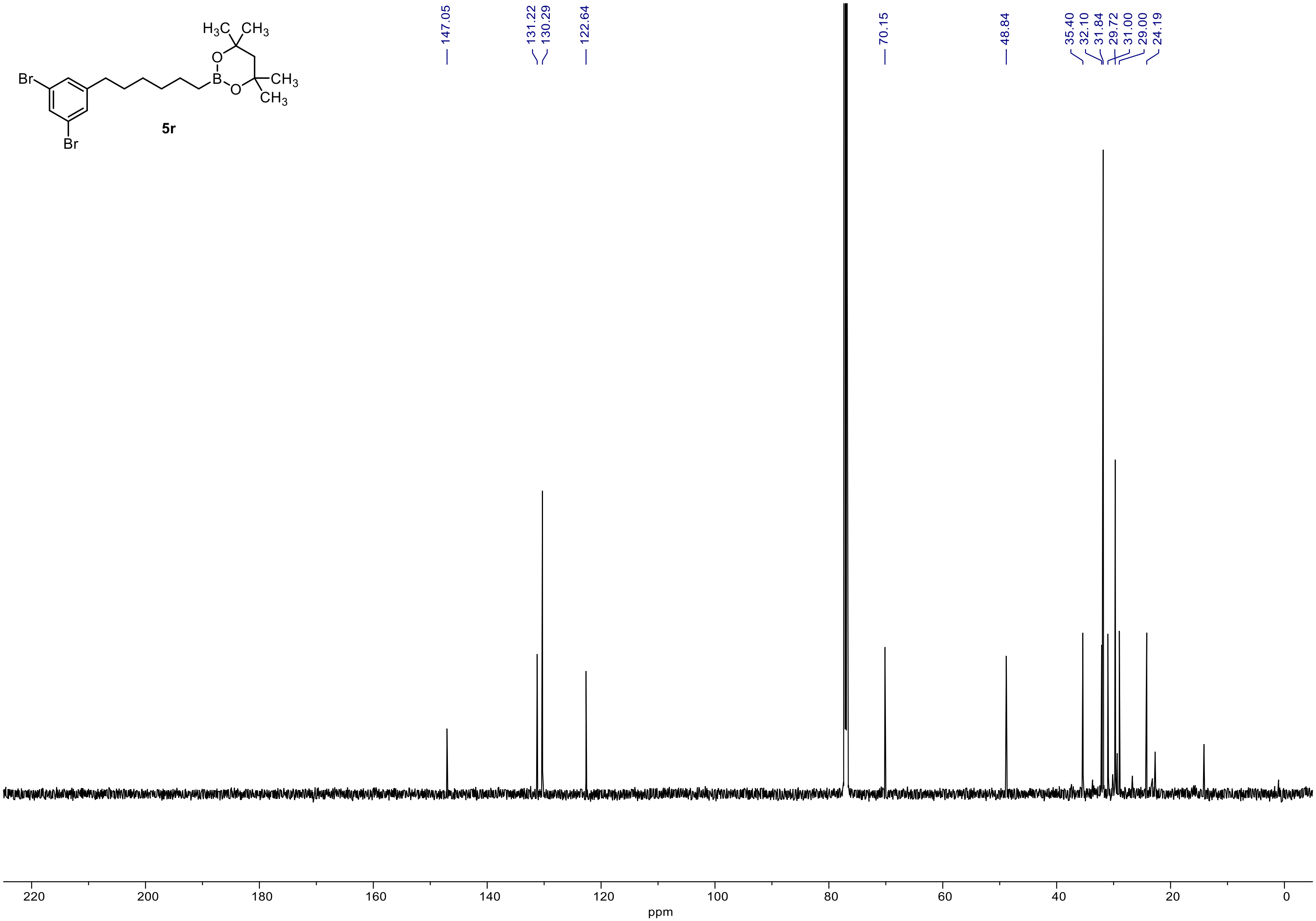

Figure S85. $^{13}C\{^{1}H\}$ NMR Spectrum of **5r** (150 MHz, $CDCl_3$).

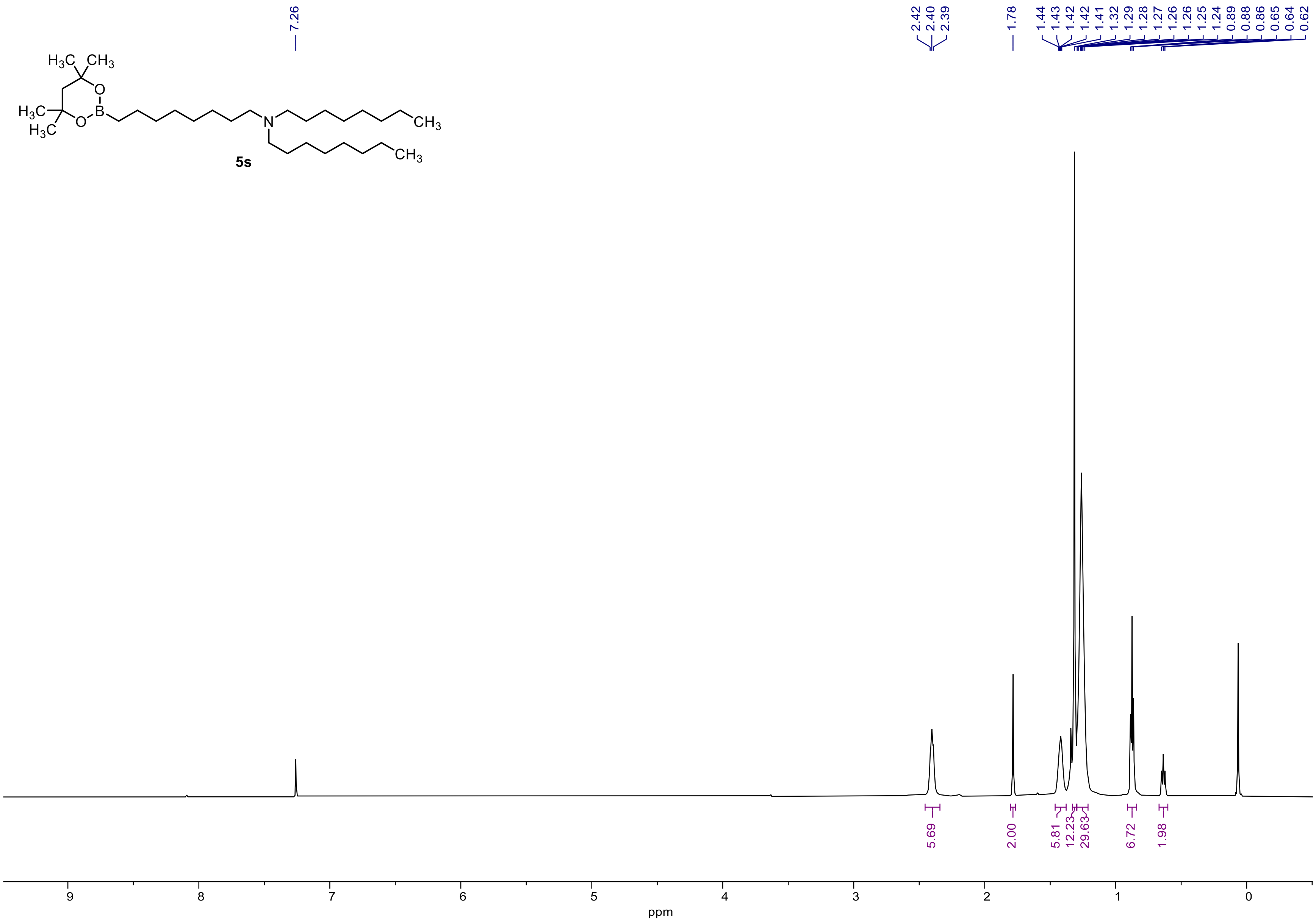

Figure S86. ^{1}H NMR Spectrum of **5s** (600 MHz, $CDCl_3$).

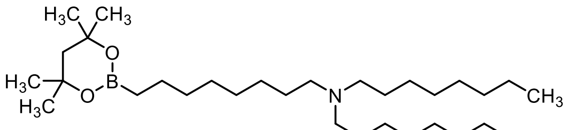

5s

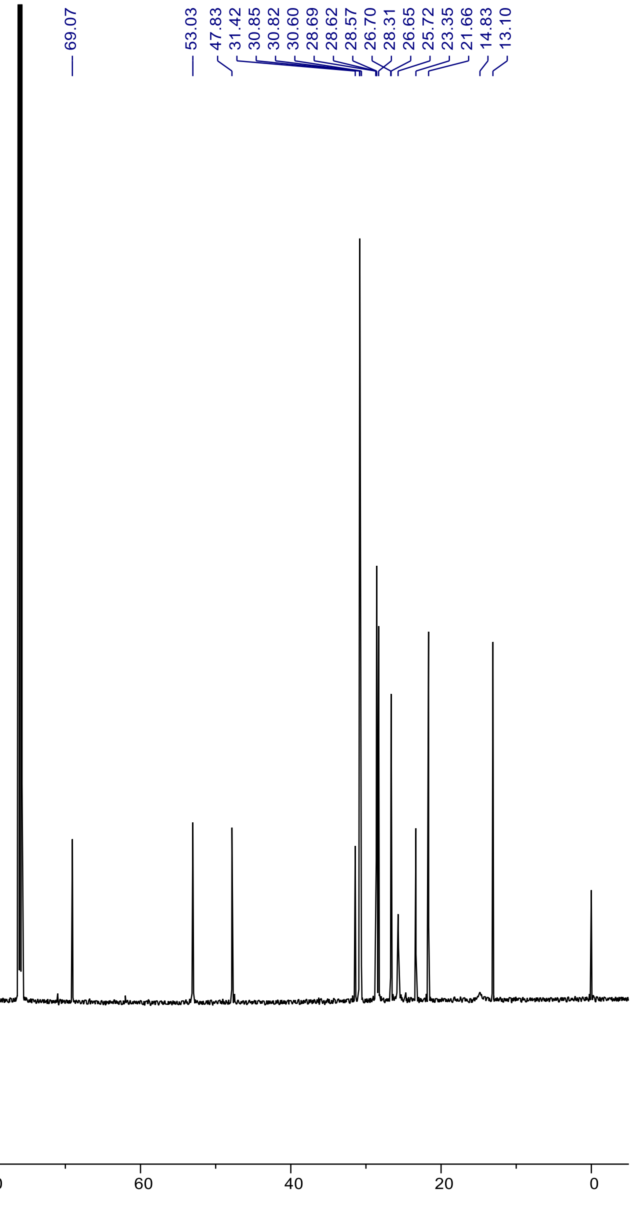

Figure S87. $^{13}C\{^{1}H\}$ NMR Spectrum of **5s** (150 MHz, $CDCl_3$).

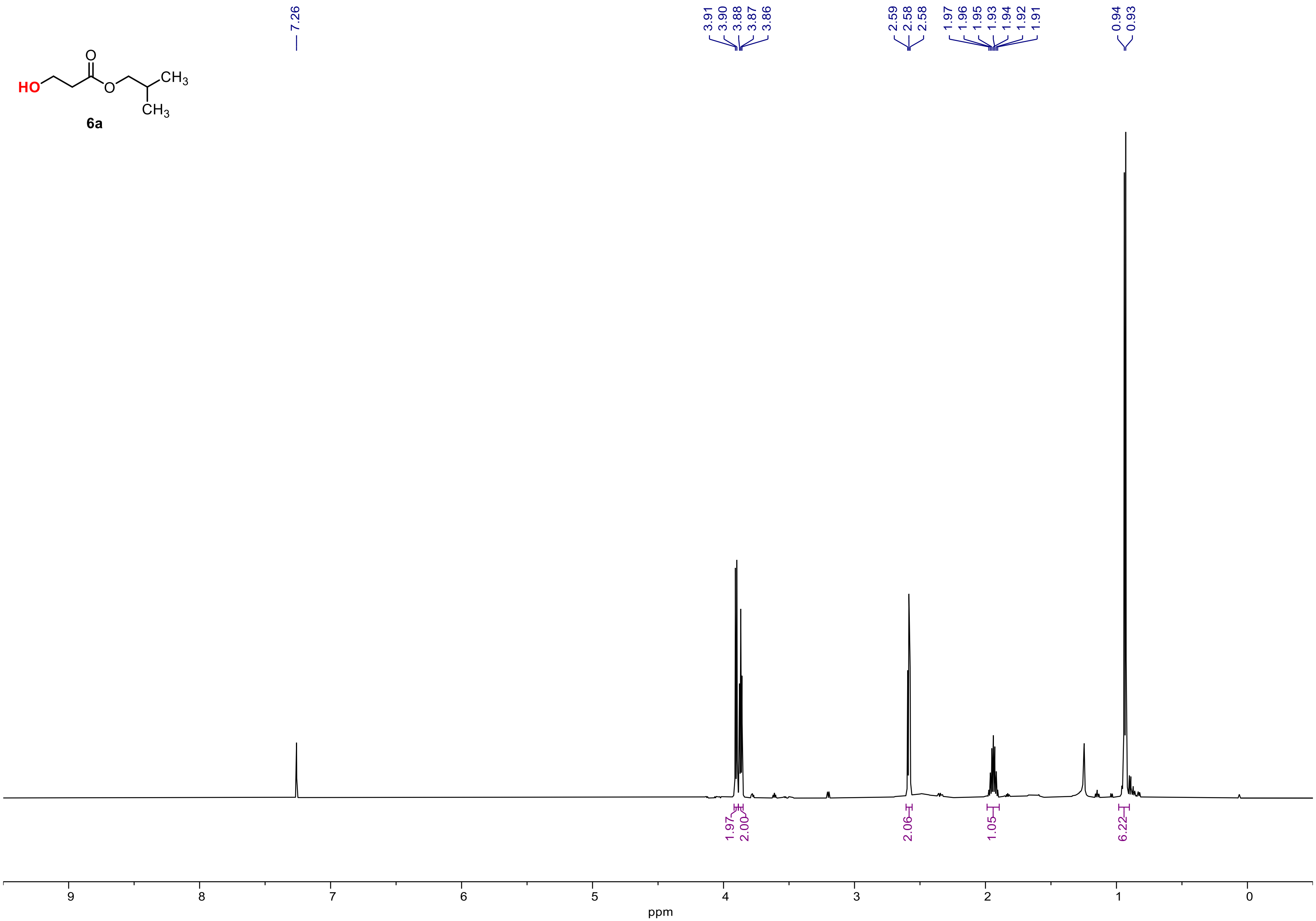

Figure S88. ^{1}H NMR Spectrum of **6a** (600 MHz, $CDCl_3$).

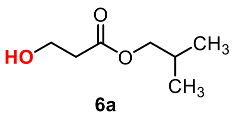

6a

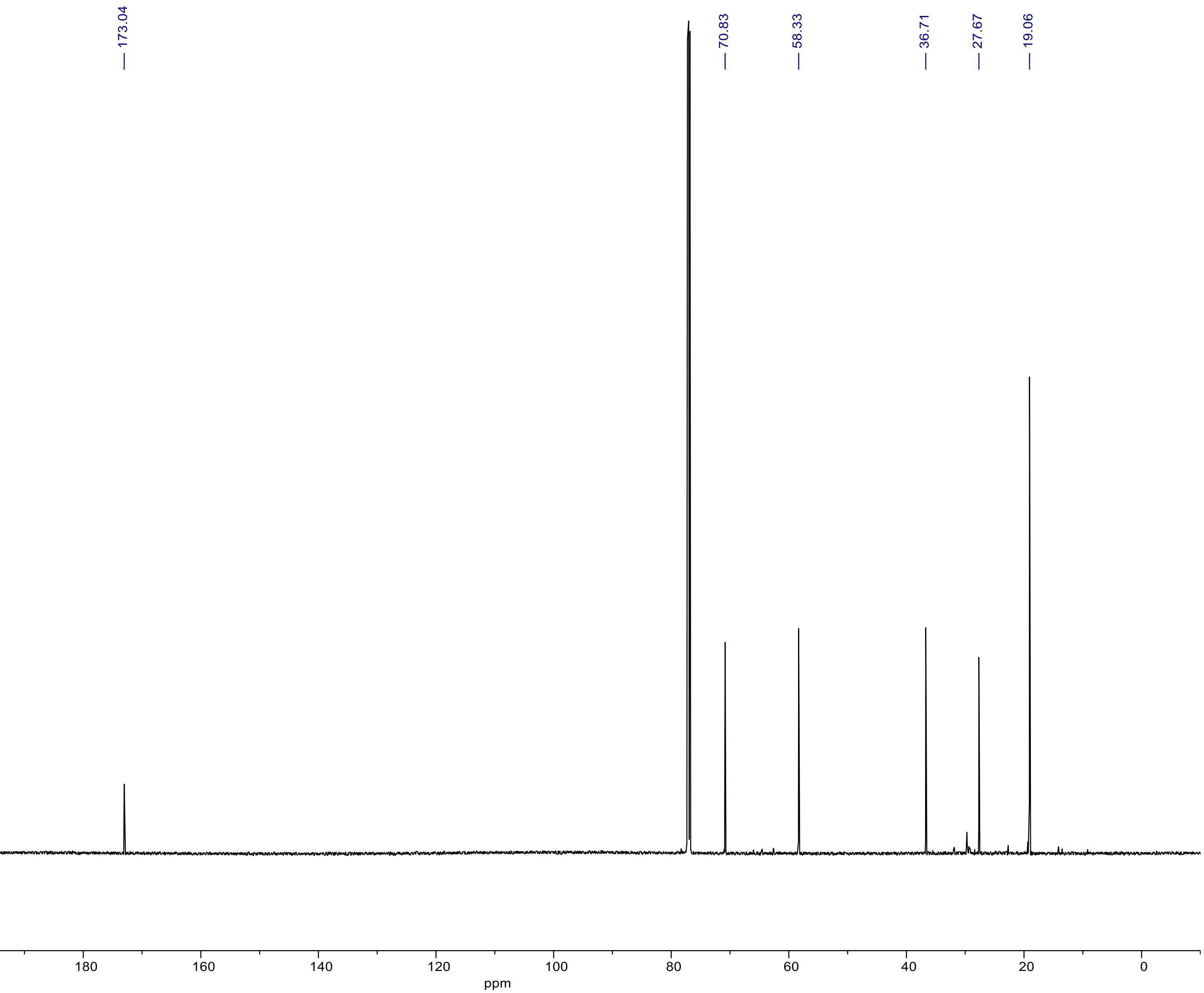

Figure S89. $^{13}C\{^{1}H\}$ NMR Spectrum of **6a** (150 MHz, $CDCl_3$).

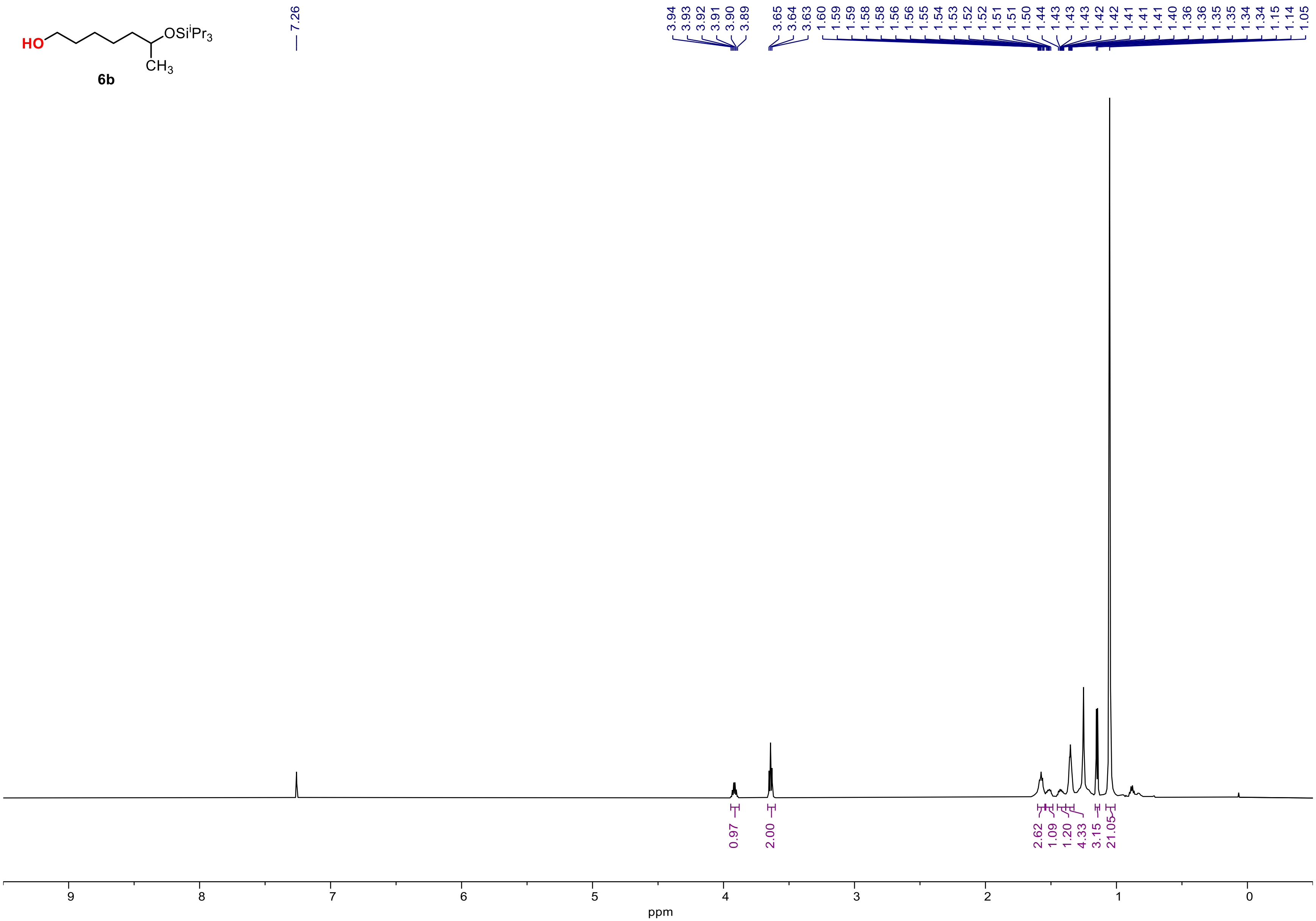

6b

Figure S90. ^{1}H NMR Spectrum of **6b** (600 MHz, $CDCl_3$).

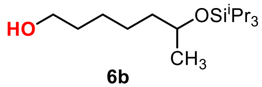

6b

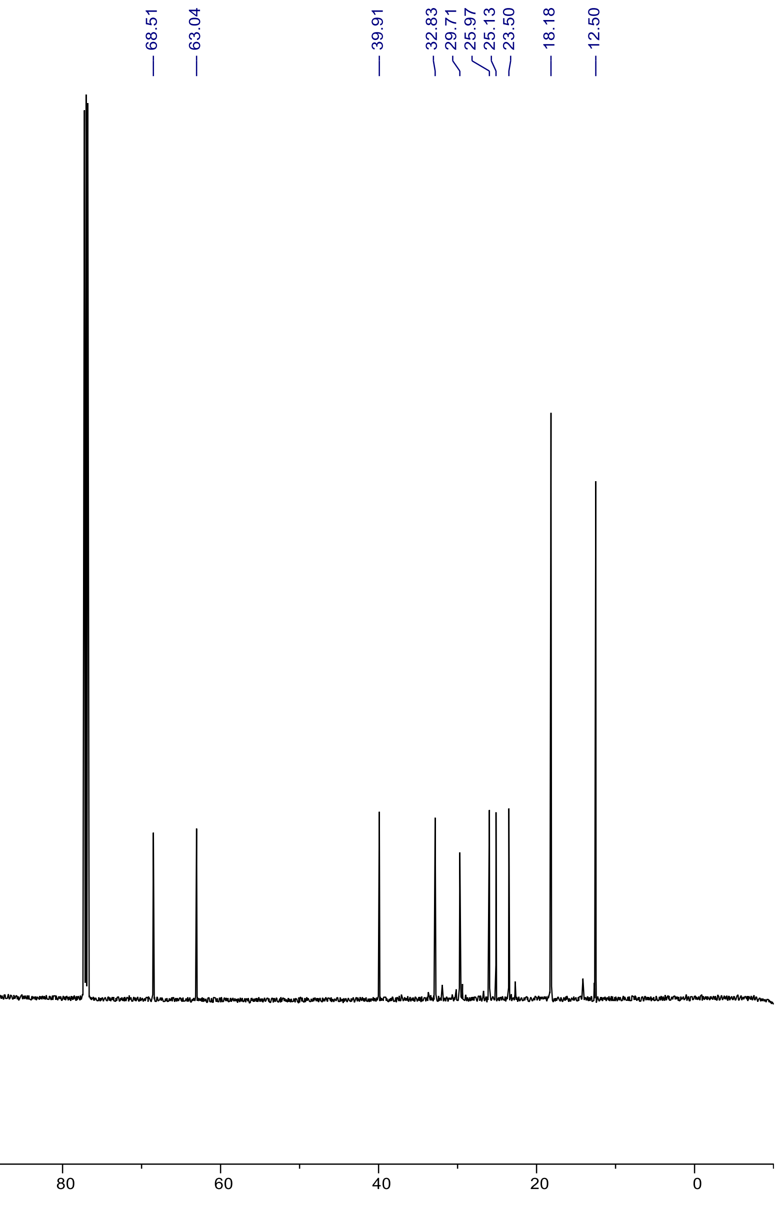

Figure S91. $^{13}C\{^{1}H\}$ NMR Spectrum of **6b** (150 MHz, $CDCl_3$).

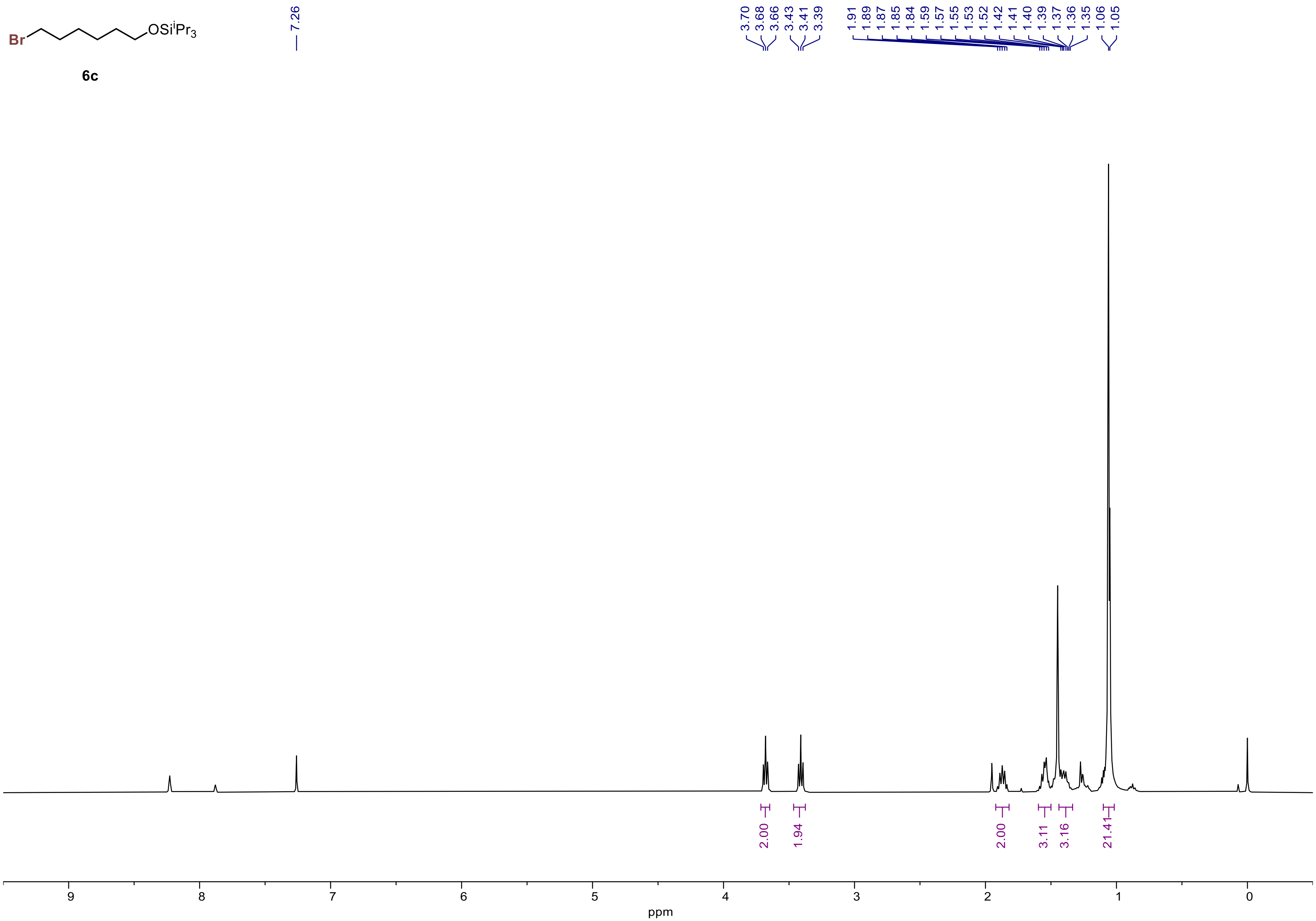

Figure S92. ^{1}H NMR Spectrum of **6c** (600 MHz, $CDCl_3$).

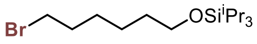

6c

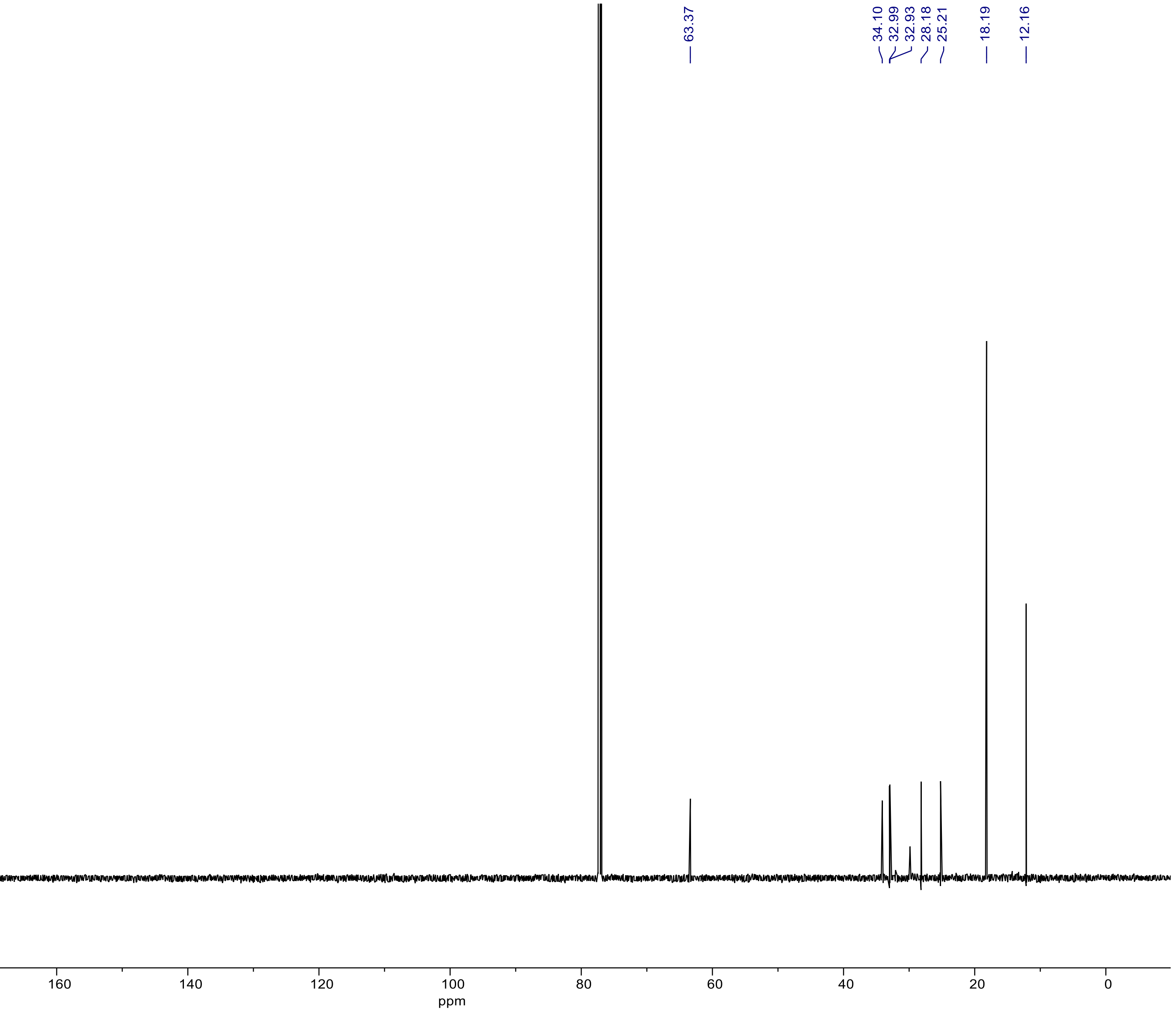

Figure S93. $^{13}C\{^1H\}$ NMR Spectrum of **6c** (150 MHz, $CDCl_3$).

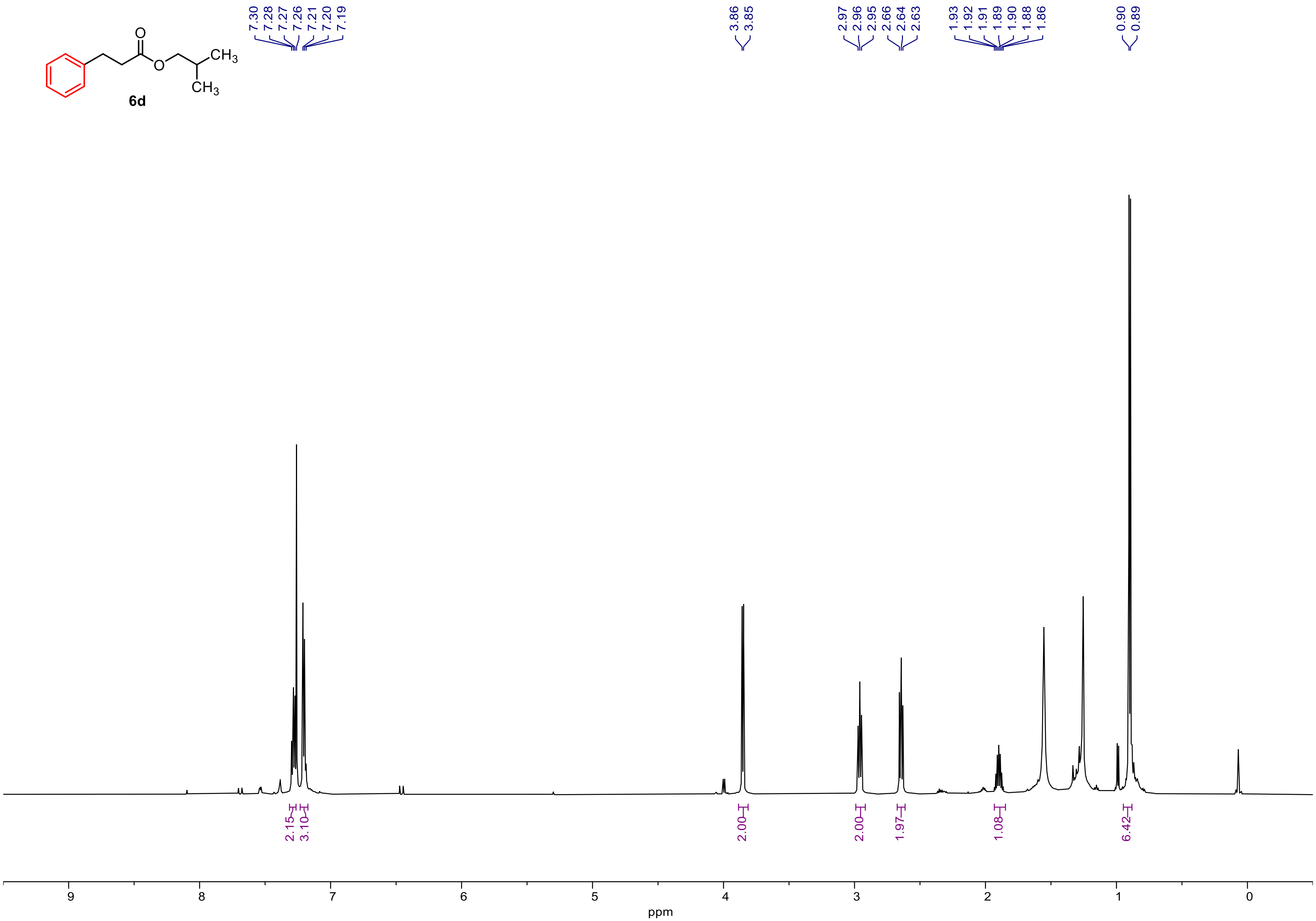

Figure S94. ^{1}H NMR Spectrum of **6d** (600 MHz, $CDCl_3$).

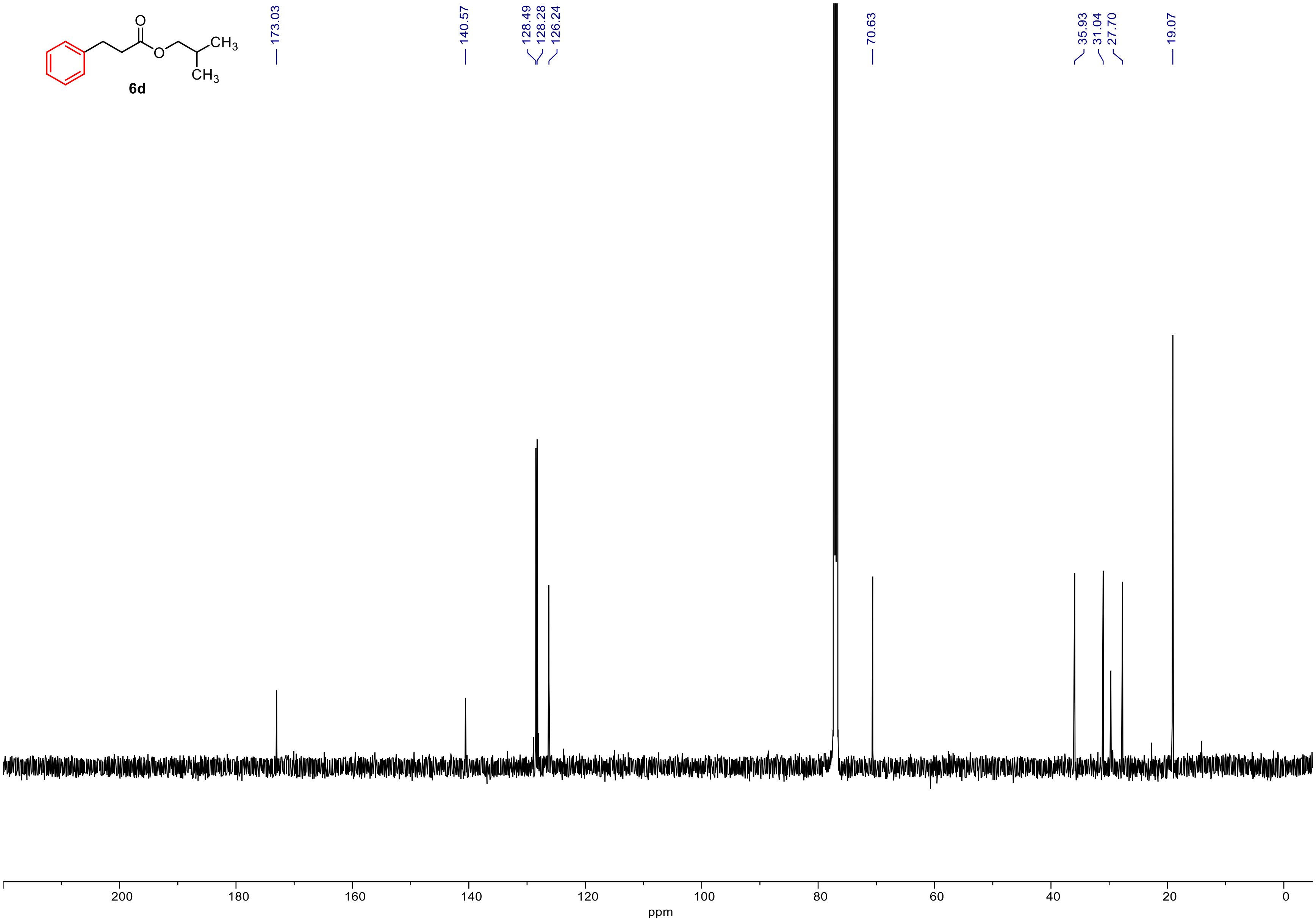

Figure S95. $^{13}C\{^{1}H\}$ NMR Spectrum of **6d** (150 MHz, $CDCl_3$).

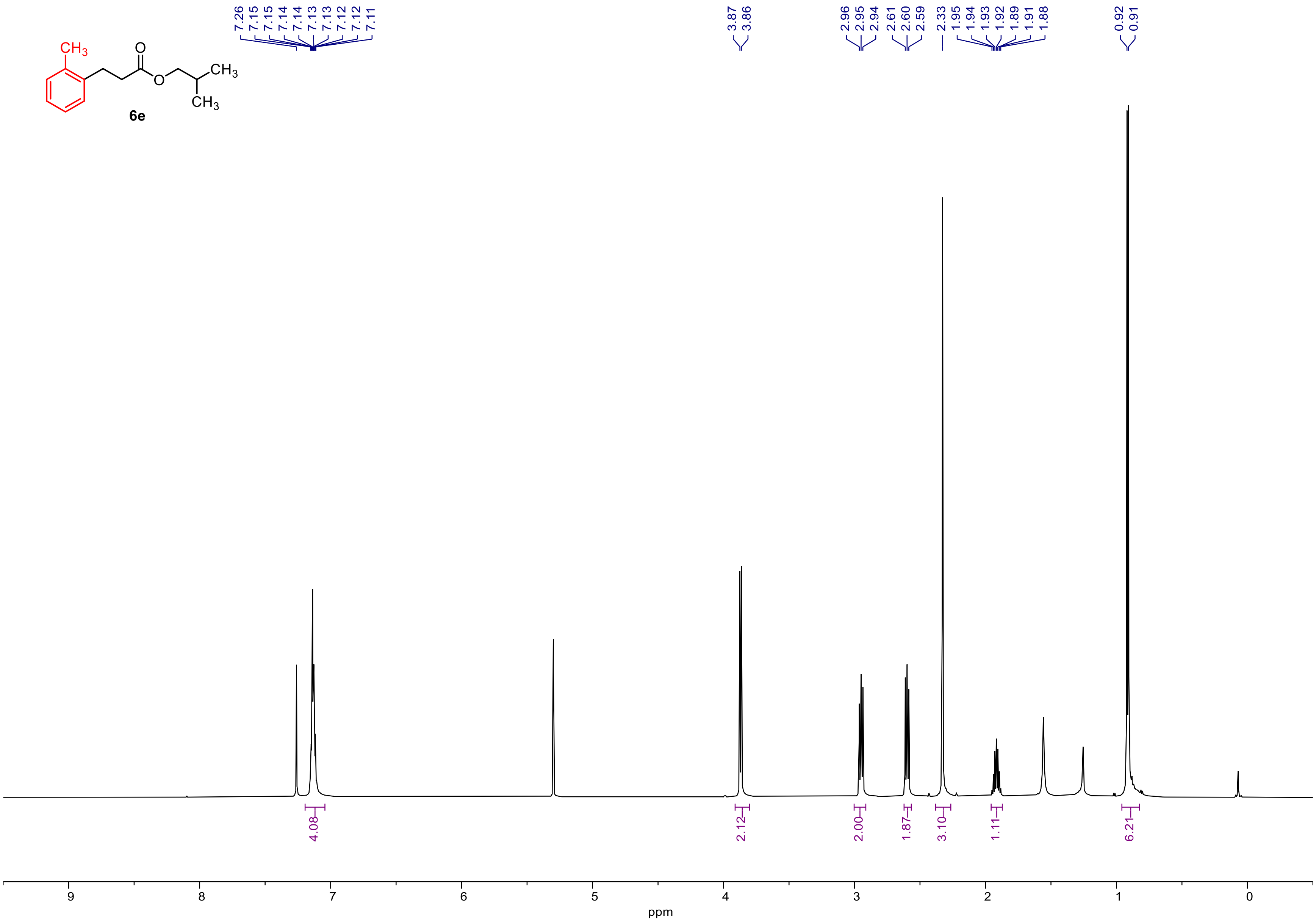

Figure S96. ^{1}H NMR Spectrum of **6e** (600 MHz, $CDCl_3$).

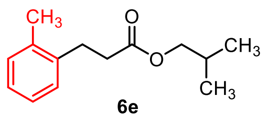

6e

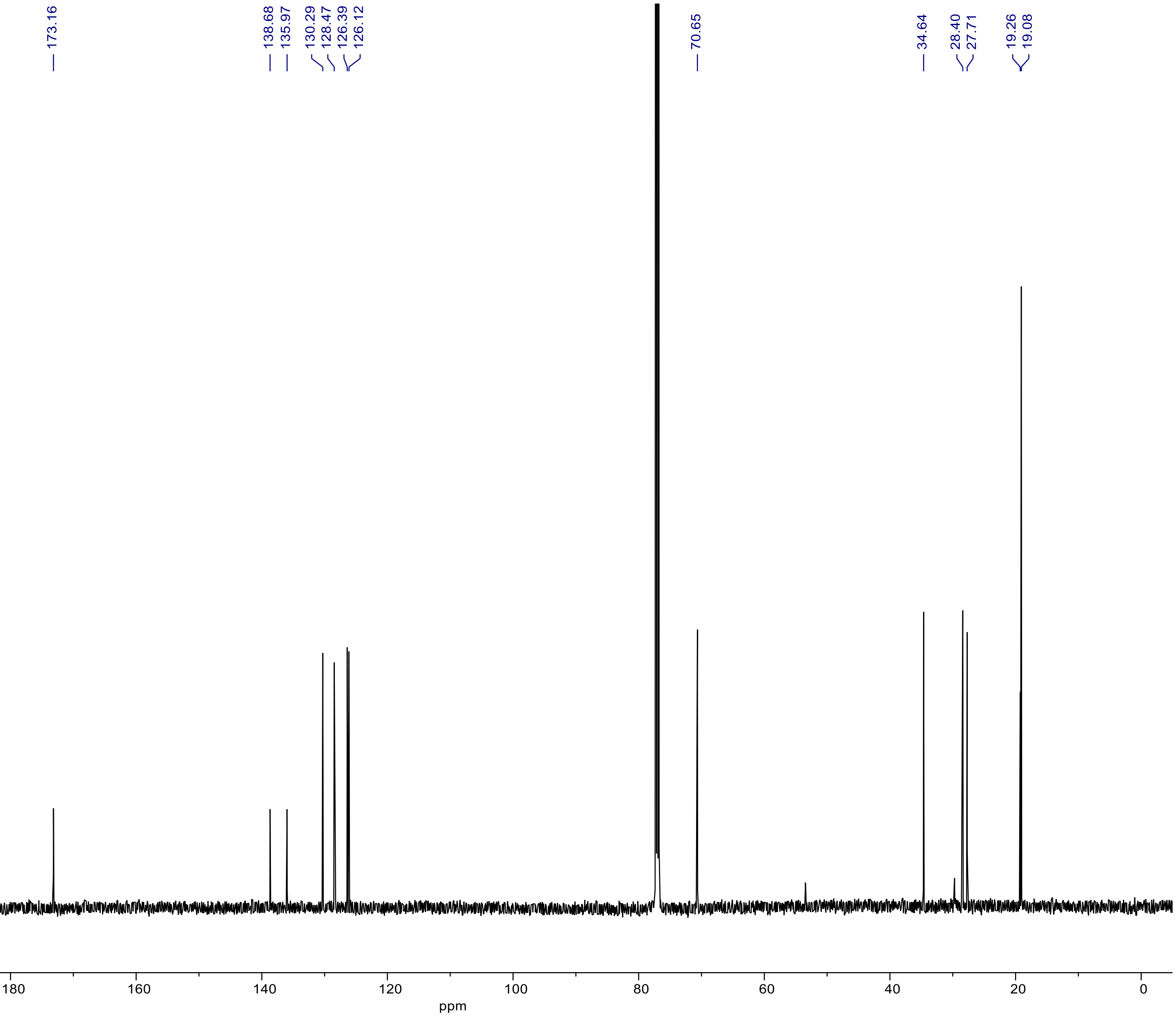

Figure S97. $^{13}C\{^{1}H\}$ NMR Spectrum of **6e** (150 MHz, $CDCl_3$).

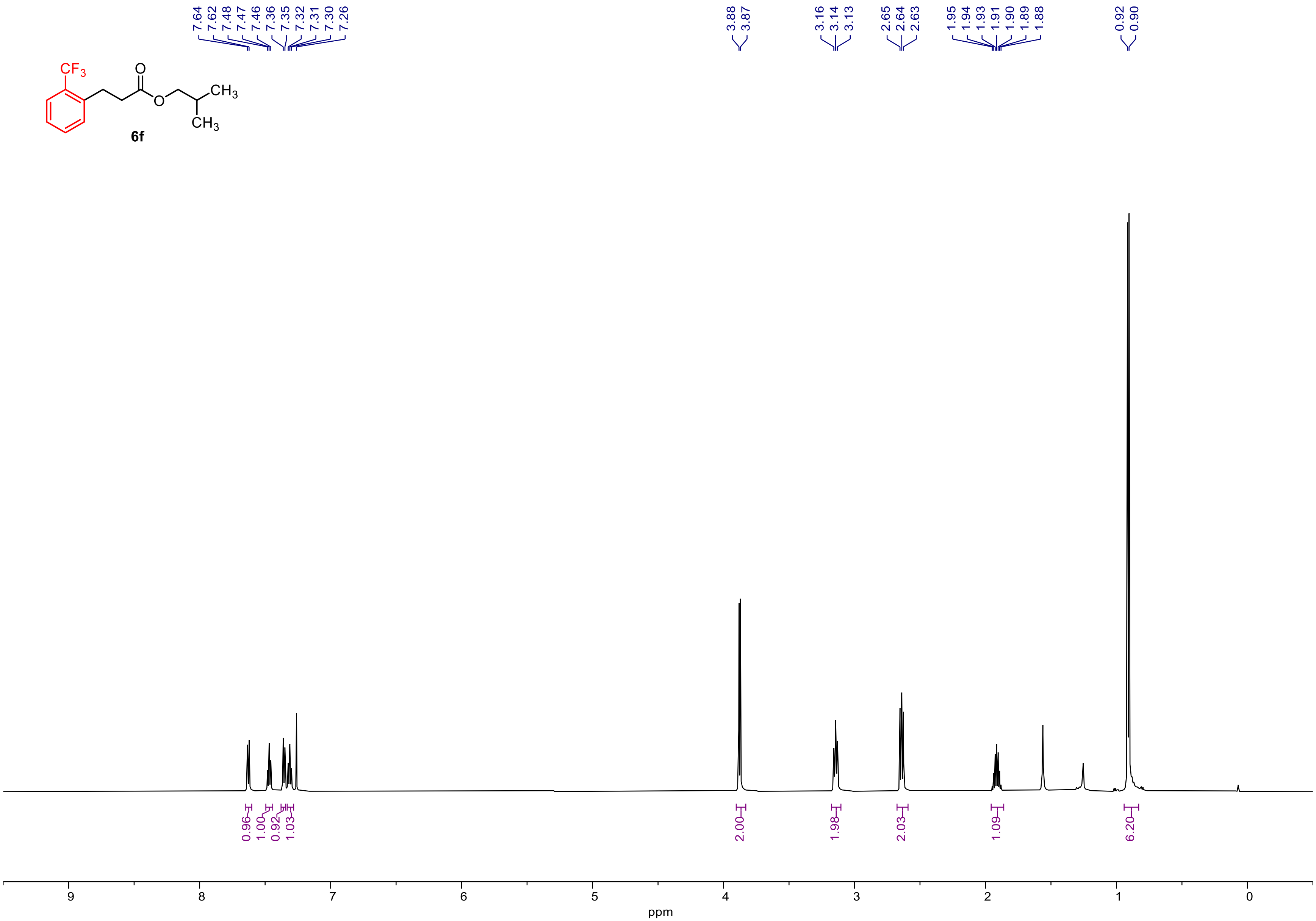

Figure S98. ^{1}H NMR Spectrum of **6f** (600 MHz, $CDCl_3$).

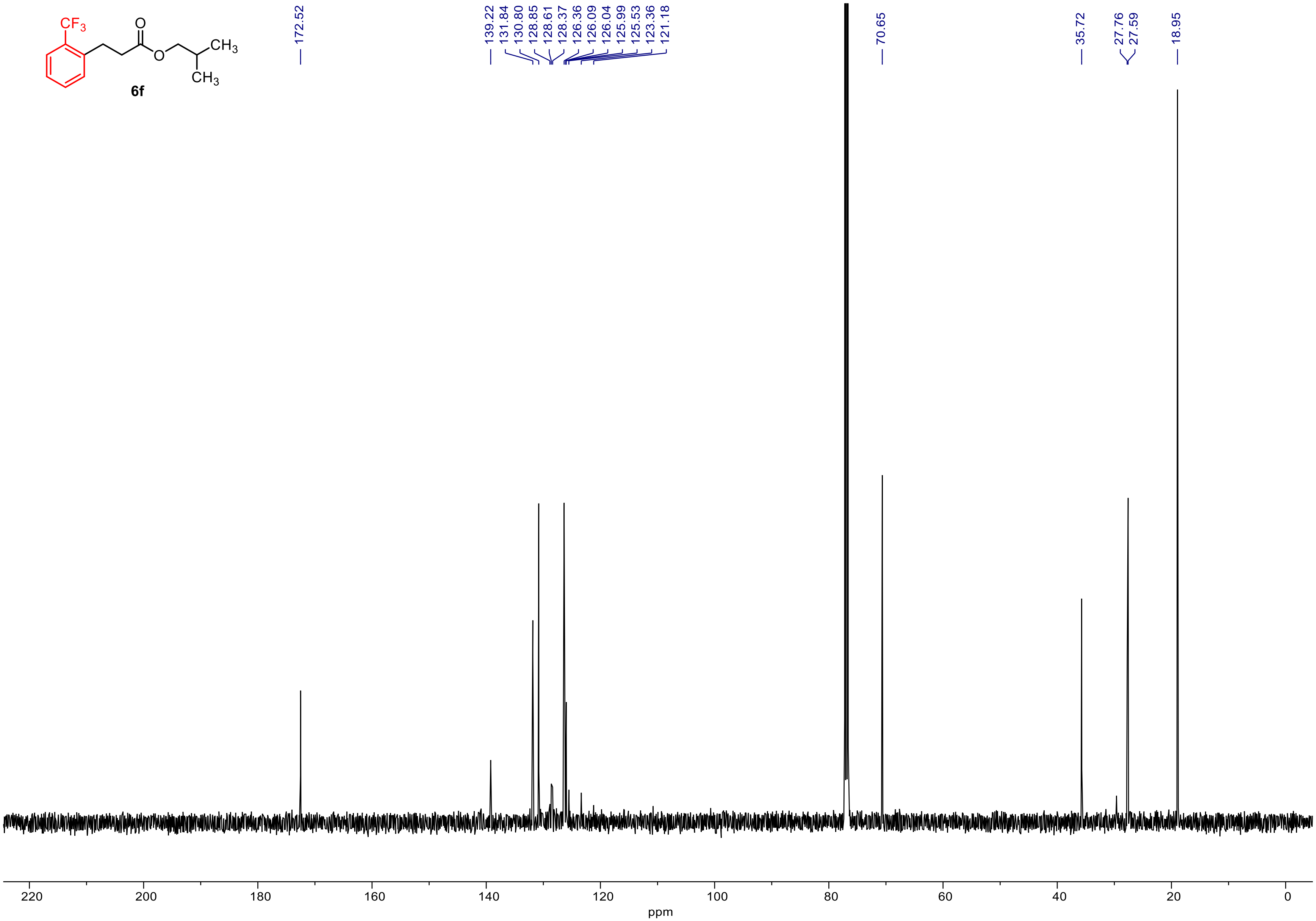

Figure S99. $^{13}C\{^1H\}$ NMR Spectrum of **6f** (150 MHz, $CDCl_3$).

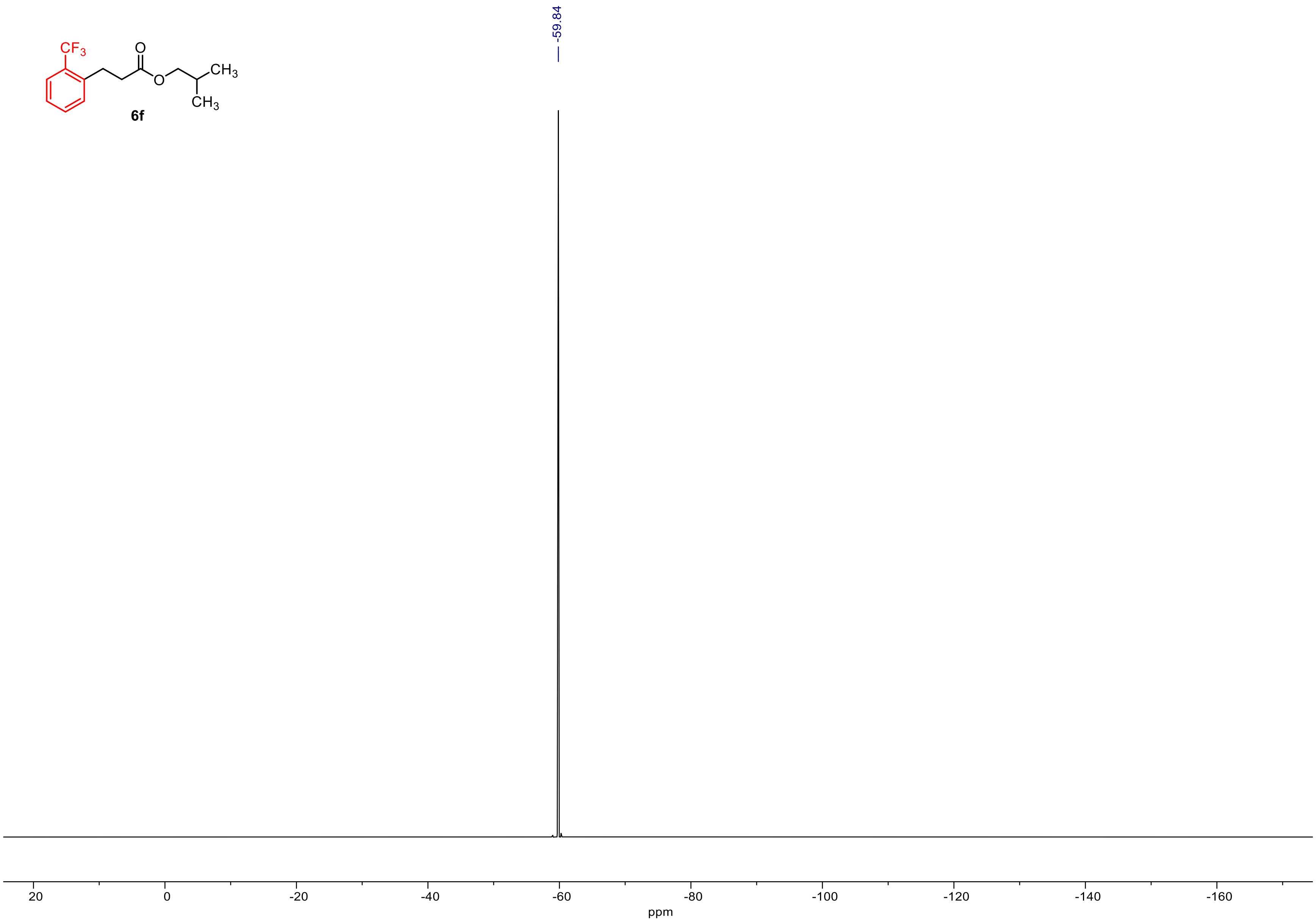

Figure S100. ^{19}F NMR Spectrum of **6f** (471 MHz, $CDCl_3$).

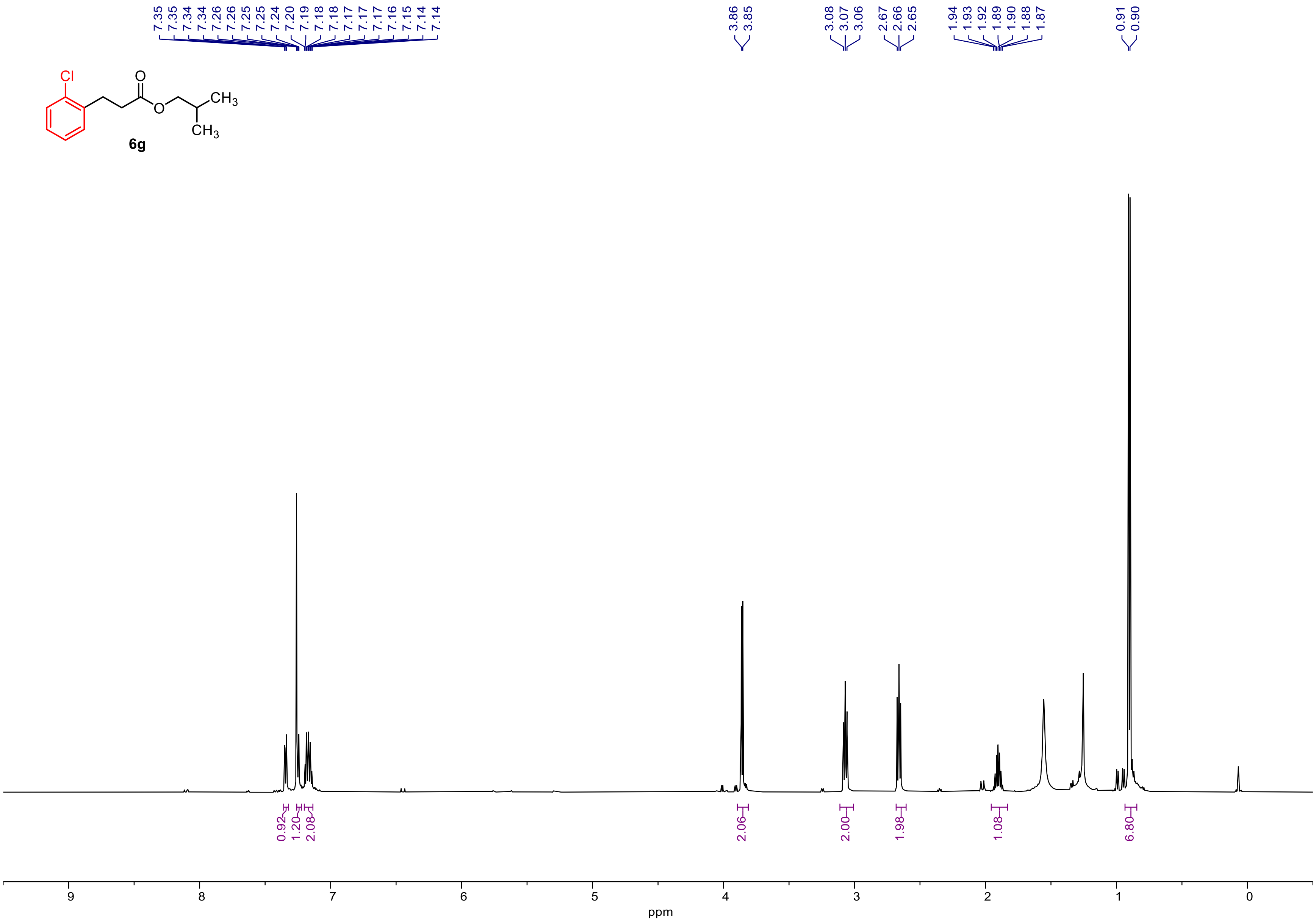

Figure S101. ^{1}H NMR Spectrum of **6g** (600 MHz, $CDCl_3$).

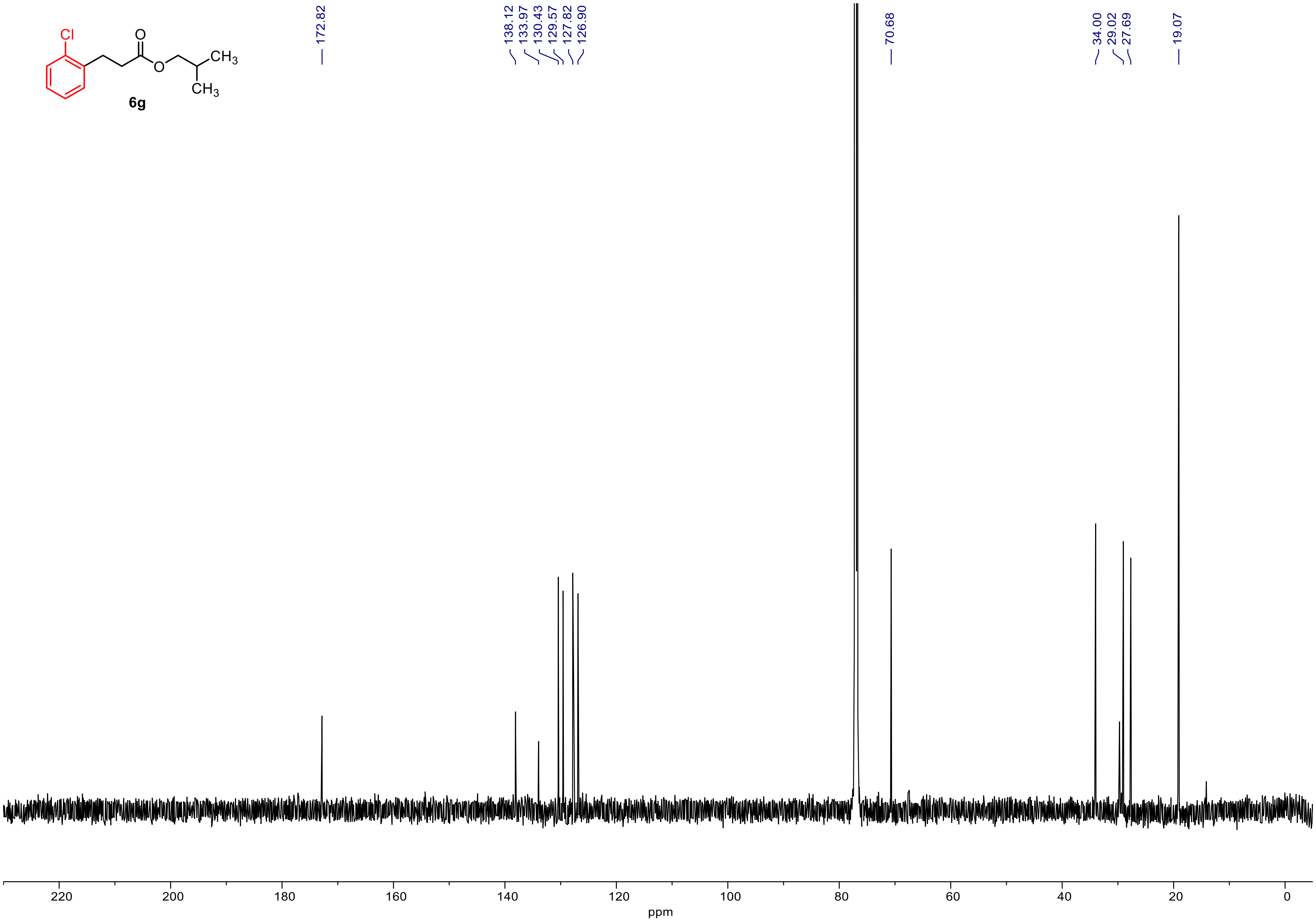

Figure S102. $^{13}C\{^{1}H\}$ NMR Spectrum of **6g** (150 MHz, $CDCl_3$).

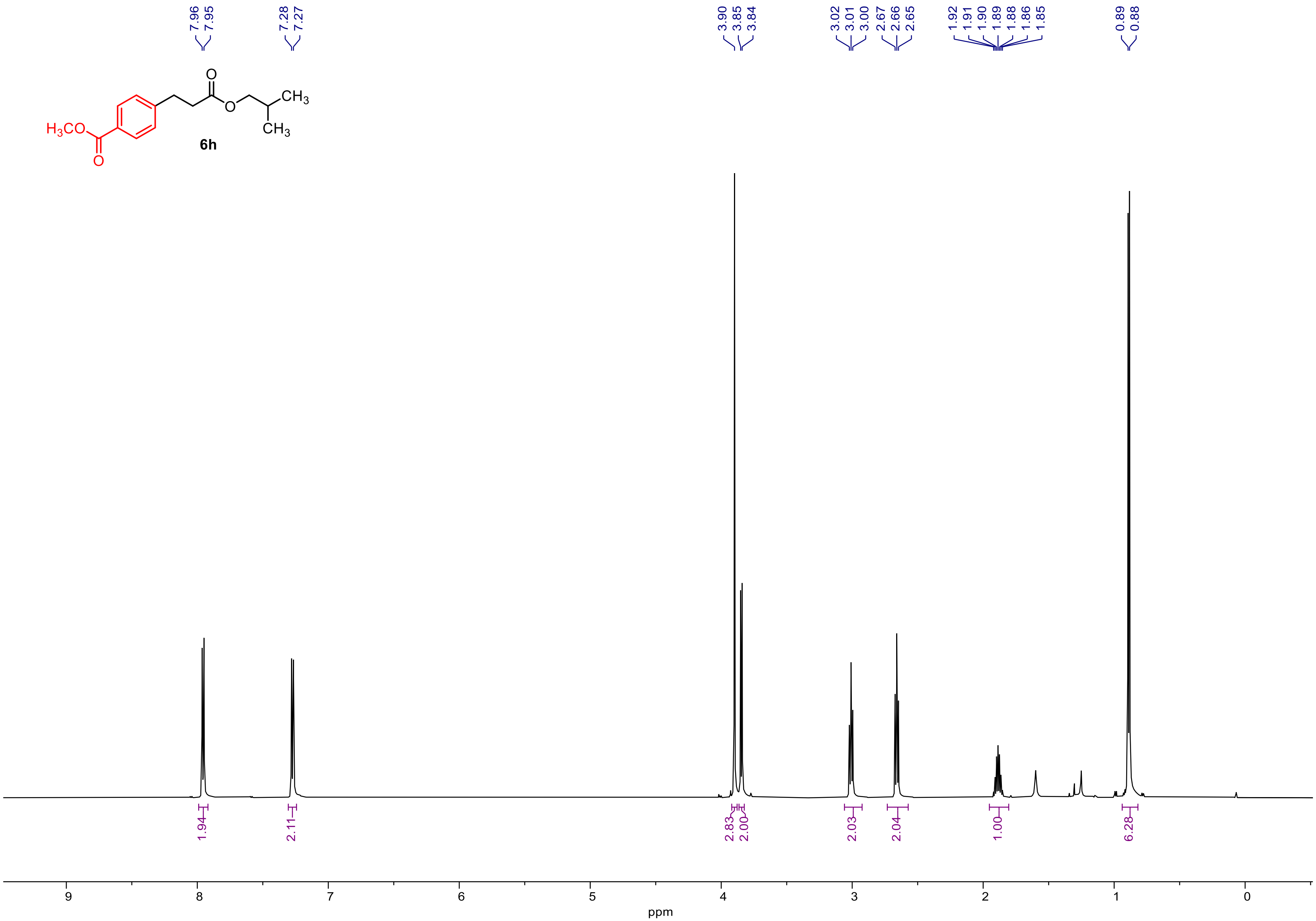

Figure S103. ^{1}H NMR Spectrum of **6h** (600 MHz, $CDCl_3$).

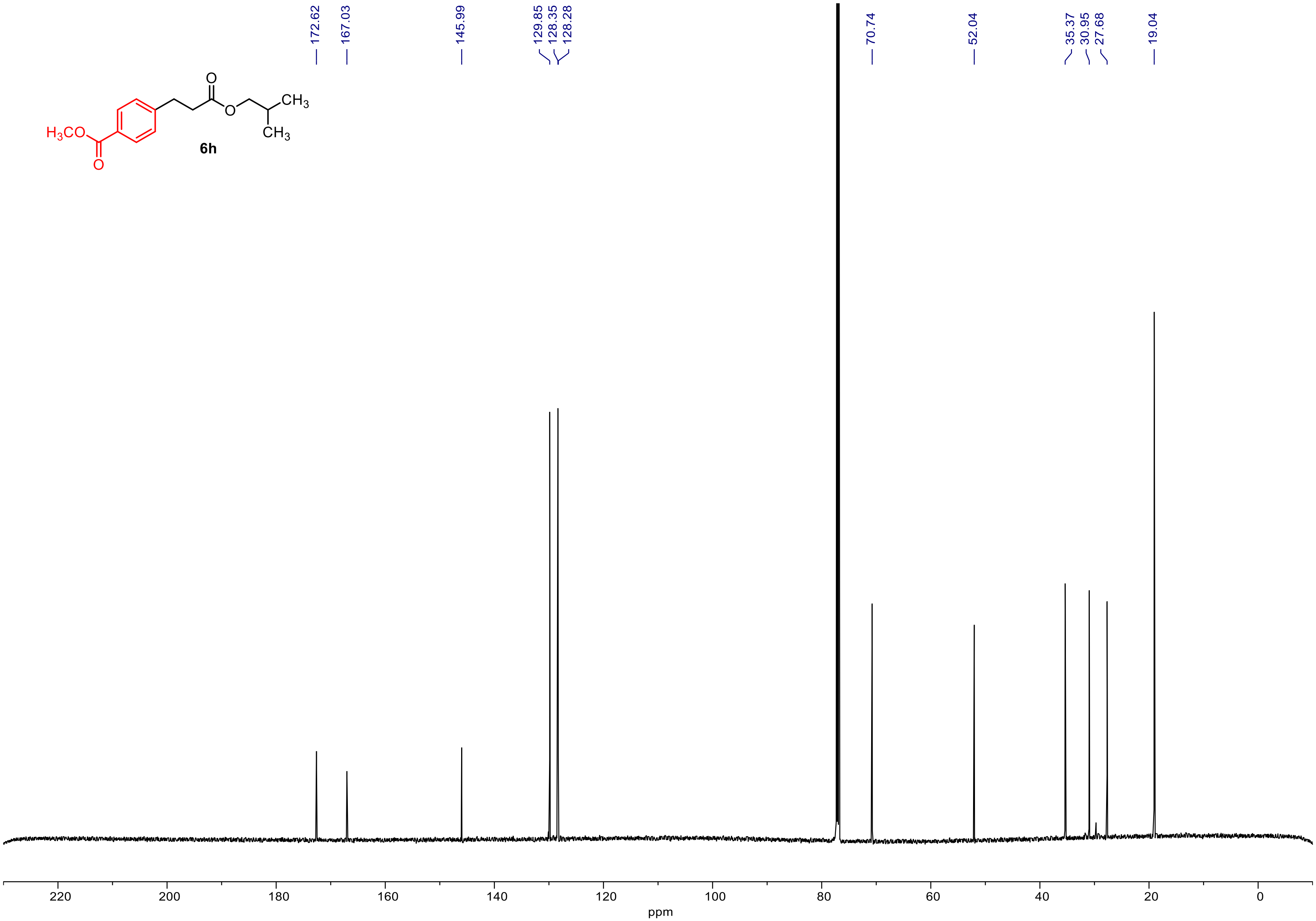

Figure S104. $^{13}C\{^{1}H\}$ NMR Spectrum of **6h** (150 MHz, $CDCl_3$).

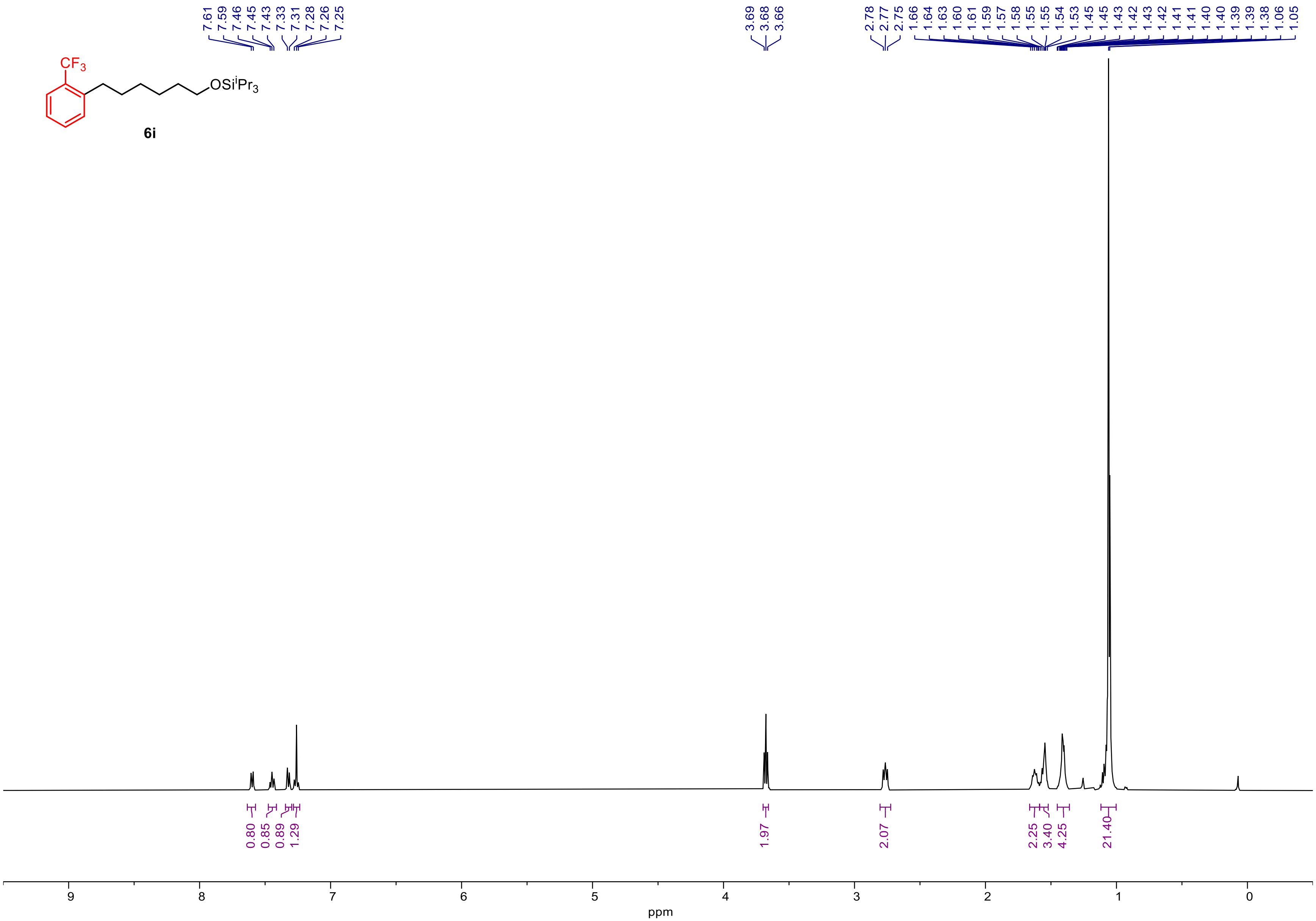

Figure S105. ^{1}H NMR Spectrum of **6i** (600 MHz, $CDCl_3$).

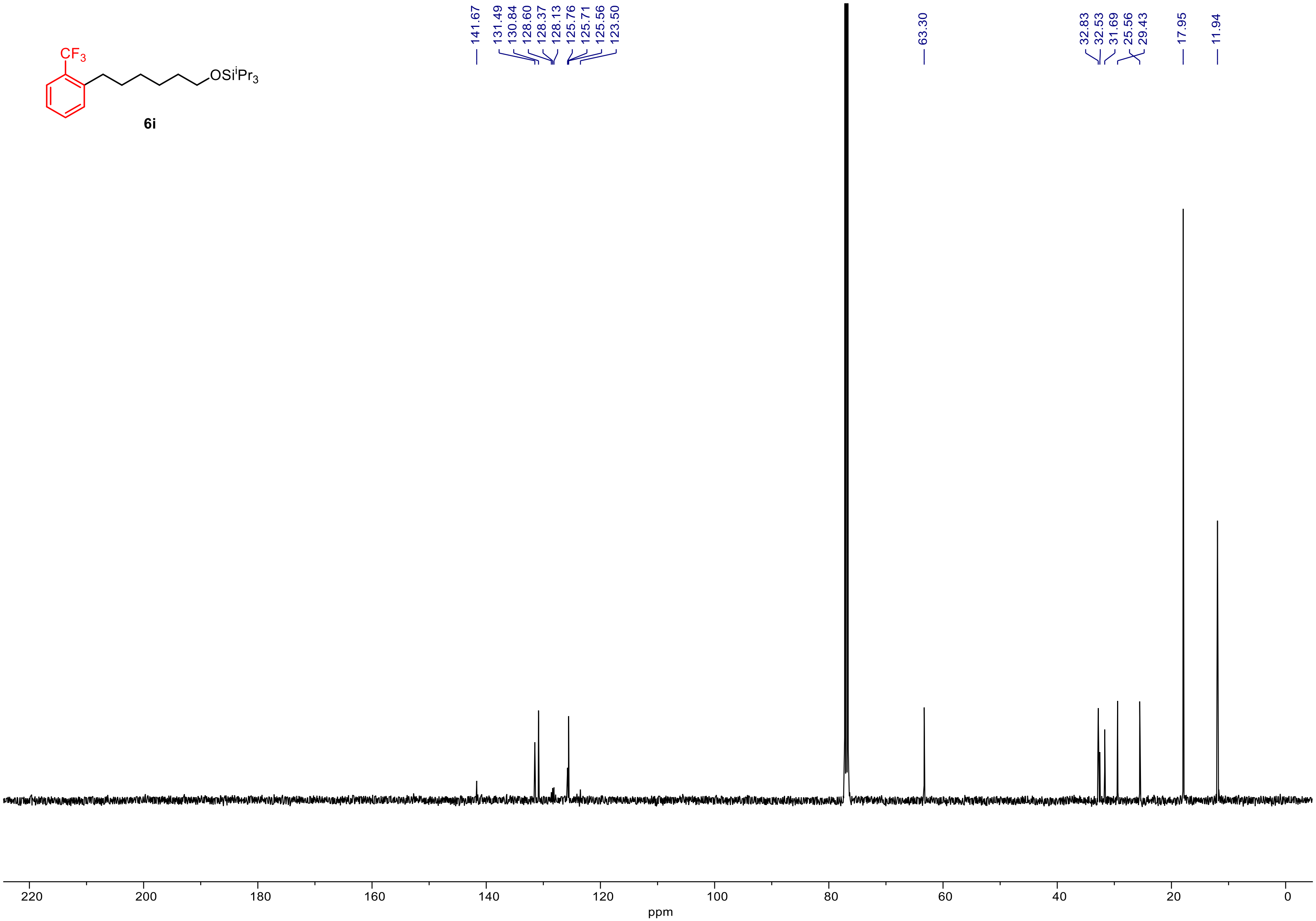

Figure S106. $^{13}C\{^{1}H\}$ NMR Spectrum of **6i** (150 MHz, $CDCl_3$).

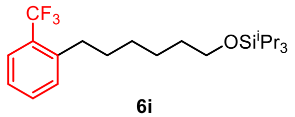

6i

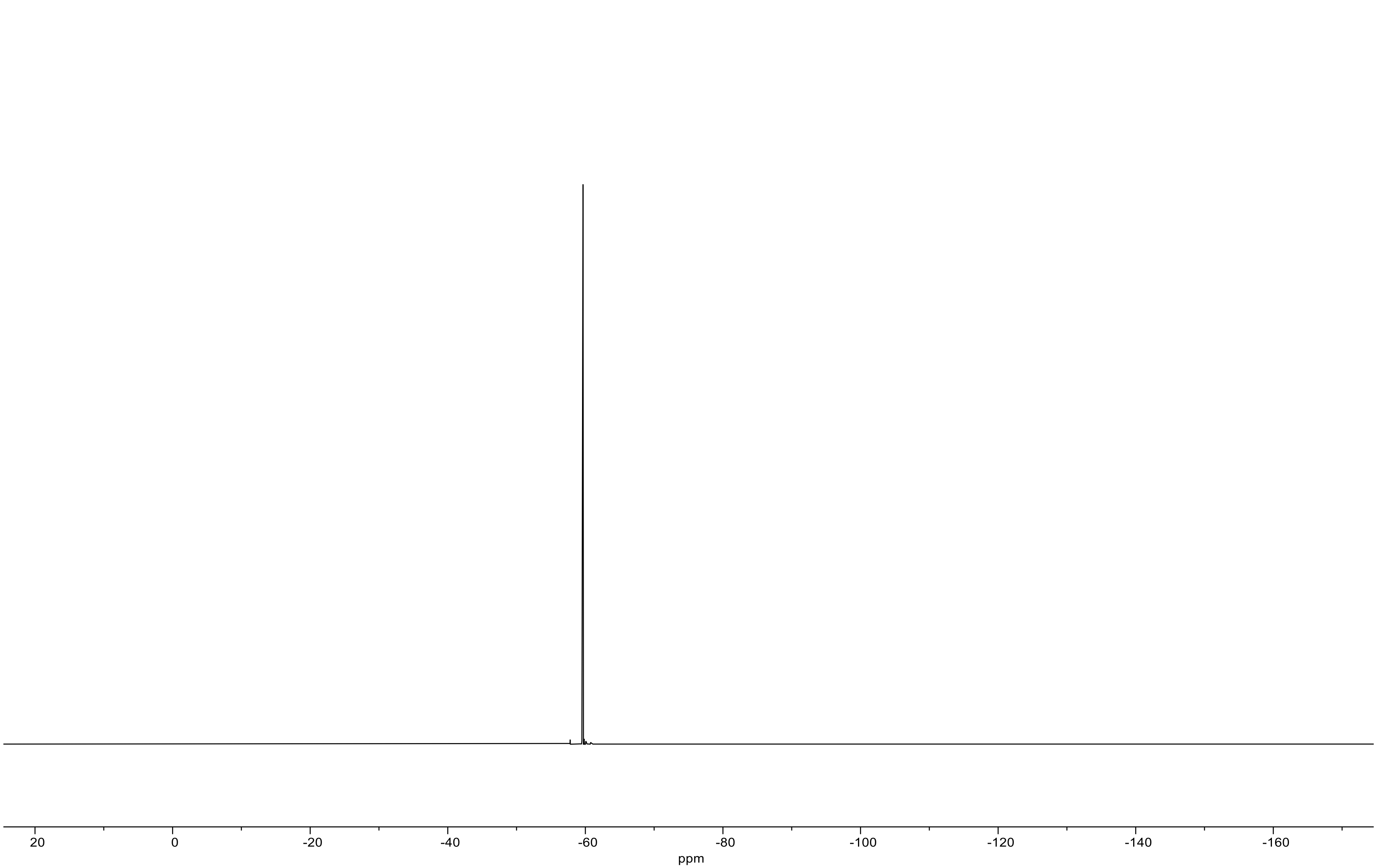

Figure S107. ^{19}F NMR Spectrum of **6i** (471 MHz, $CDCl_3$).

VII. X-ray Crystallographic Data

Details of crystallographic refinement

General Methods. A suitable crystal of each sample was selected for analysis and mounted in a polyimide loop. Crystal samples were handled under immersion oil and quickly transferred to a cold nitrogen stream. All measurements were made on a Rigaku Oxford Diffraction Supernova Eos CCD with filtered Cu-Kα radiation at a temperature of 100 K. Using Olex2,[9] the structure was solved with the ShelXT structure solution program using Direct Methods and refined with the ShelXL refinement package[10] using Least Squares minimization.

(κ^3–L2)IrH$_3$(SiEt$_3$) (7)

'The crystal was a merohedral twin related by the twin law [0 0 -1 0 -1 0 -1 0 0]. The metal hydrides were located in the difference map and their positions refined with Ir-H bond distances restrained to be equal and with atomic thermal parameters fixed to ride on the parent iridium atom.

Table S5. Crystal data and structure refinement for (κ^3–L2)IrH_3(SiEt$_3$)

Empirical formula	$C_{25}H_{34}FIrN_2Si$	
Formula weight	601.83	
Temperature	100.00(10) K	
Wavelength	1.54184 Å	
Crystal system	Monoclinic	
Space group	P 1 21/n 1	
Unit cell dimensions	a = 15.3832(5) Å	α = 90°
	b = 10.5957(3) Å	β = 106.763(3)°
	c = 15.4084(4) Å	γ = 90°
Volume	2404.78(13) Å^3	
Z	4	
Density (calculated)	1.662 Mg/m^3	
Absorption coefficient	11.391 mm^{-1}	
F(000)	1192	
Crystal size	0.102 x 0.074 x 0.021 mm^3	
Theta range for data collection	2.995 to 71.769°.	
Index ranges	-18<=h<=18, -10<=k<=12, -18<=l<=18	
Reflections collected	19153	
Independent reflections	4635 [R(int) = 0.0454]	
Completeness to theta = 67.684°	100.0 %	
Absorption correction	Gaussian	
Max. and min. transmission	0.822 and 0.478	
Refinement method	Full-matrix least-squares on F^2	
Data / restraints / parameters	4635 / 3 / 286	
Goodness-of-fit on F^2	1.021	
Final R indices [I>2sigma(I)]	R1 = 0.0391, wR2 = 0.1008	
R indices (all data)	R1 = 0.0448, wR2 = 0.1059	
Extinction coefficient	n/a	
Largest diff. peak and hole	1.783 and -2.075 e/Å^{-3}	

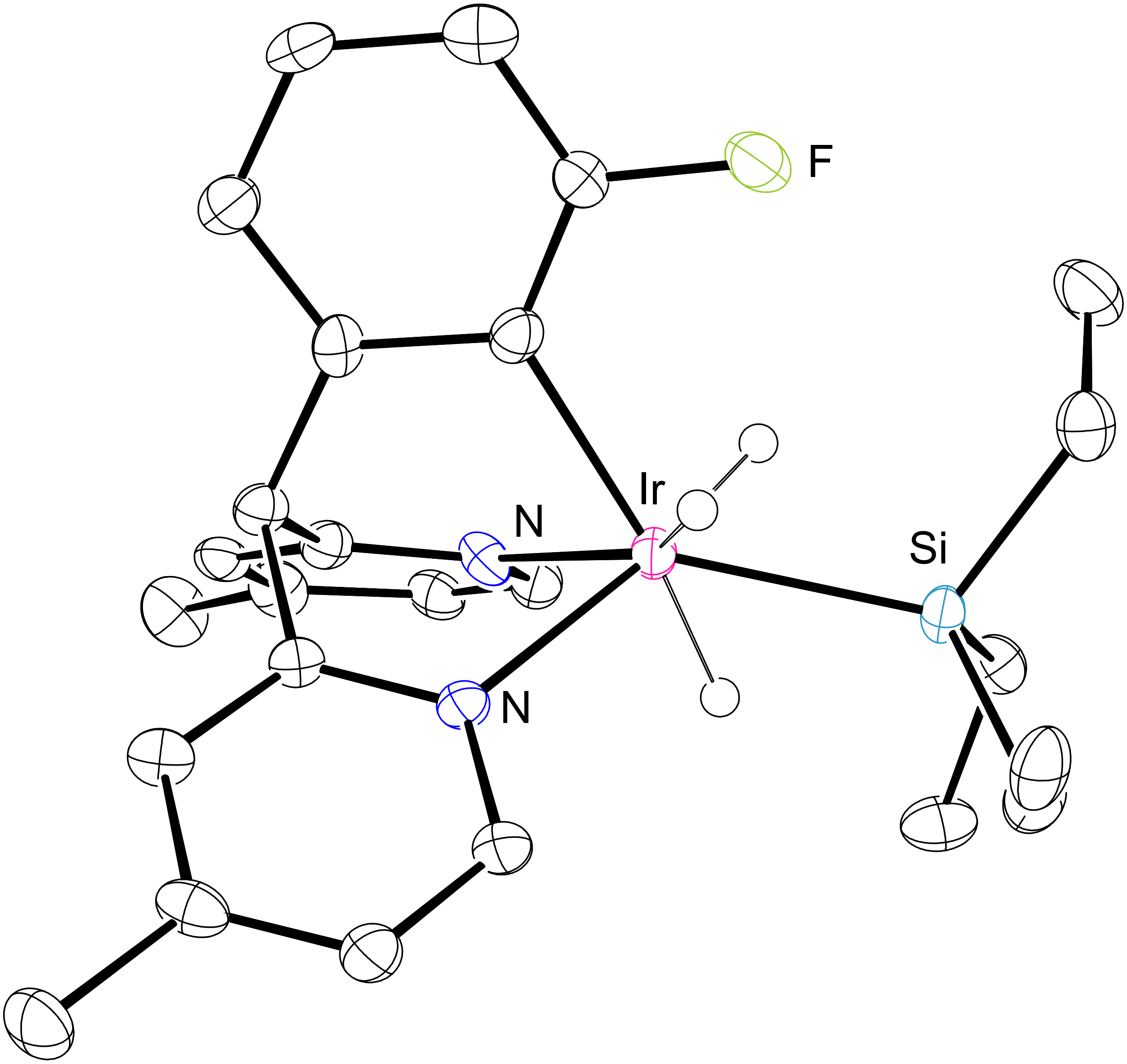

Figure S108. ORTEP of (κ^3–L2)IrH_3(SiEt$_3$). Hydrogen atoms bound to carbon are omitted for clarity.

VIII. Computational Methods

General Methods. Density functional theory (DFT) calculations were performed using Gaussian 16.[11]

Computational treatment of Compound 7. X-ray crystallographic data provided initial atomic coordinates for the iridium complex. A DFT optimization and frequency calculation was performed to compute the free energy of each complex and compound using the M06[12] functional with the following basis sets: (CHNF: def2SVP, IrSi: def2TZVP).[13] The ECP for Ir was retrieved from the EMSL basis set exchange (http://bse.pnl.gov/).[14]A dispersion correction was included.[15] The tabulated free energies were used to calculate ΔG° for the observed isomer of compound **7**.

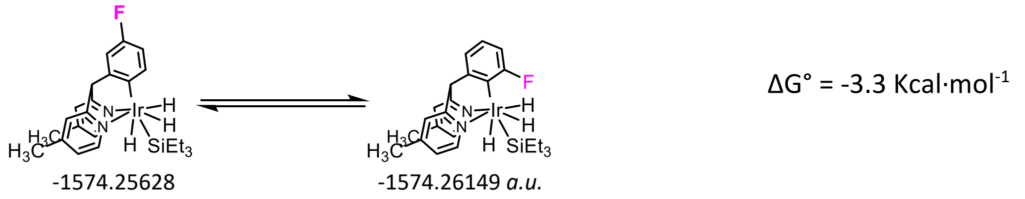

Figure S109. Computational treatment of potential isomerism in compound **7** .

Computational treatment of [Ir(μ-Cl)(H)$_2$(SiEt$_3$)$_2$]$_2$. The low-quality X-ray crystallographic data discussed above provided initial atomic coordinates. A DFT optimization and frequency calculation was performed to find the optimized structure using the M06[12] functional with the following basis sets: (CH: def2SVP, IrSiCl: def2TZVP)[13] and dispersion correction.[15] A stable structure consistent with the X-ray data could not be located if the hydrides were omitted in the calculation.

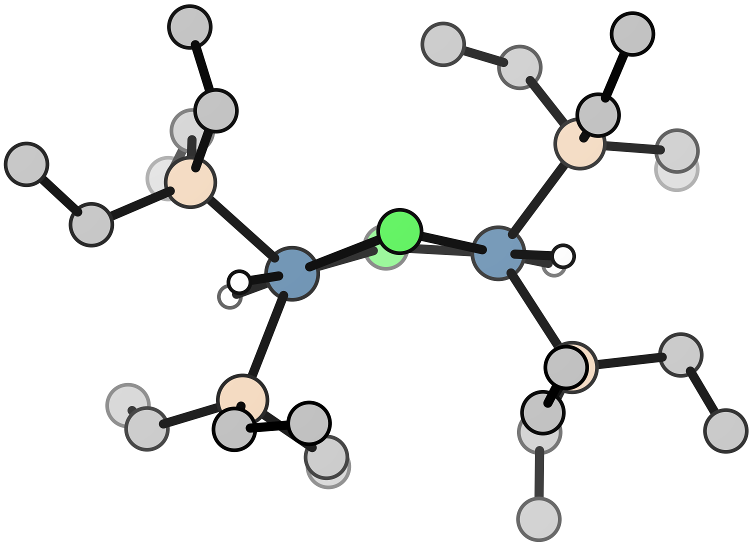

Figure S110. Computed structure of **[Ir(μ-Cl)(H)$_2$(SiEt$_3$)$_2$]$_2$.** Most hydrogen atoms are omitted for clarity.[16]

The supplemental file "calc_coords.xyz" contains the computed Cartesian coordinates of all of the molecules reported in this study. The file may be opened as a text file to read the coordinates, or opened directly by a molecular modeling program such as Mercury (version 3.3 or later, http://www.ccdc.cam.ac.uk/pages/Home.aspx) for visualization and analysis.

IX. References

(1) Rosenau, C. P.; Jelier, B. J.; Gossert, A. D.; Togni, A., Exposing the Origins of Irreproducibility in Fluorine NMR Spectroscopy. *Angew. Chem. Int. Ed.* **2018**, *57*, 9528-9533.
(2) Harris, R. K.; Becker, E. D.; Cabral de Menezes, S. M.; Granger, P.; Hoffman, R. E.; Zilm, K. W., Further conventions for NMR shielding and chemical shifts (IUPAC Recommendations 2008). *Pure Appl. Chem.* **2008**, *80*, 59-84.
(3) Chotana, G. A.; Vanchura, I. I. B. A.; Tse, M. K.; Staples, R. J.; Maleczka, J. R. E.; Smith, I. I. I. M. R., Getting the sterics just right: a five-coordinate iridium trisboryl complex that reacts with C–H bonds at room temperature. *Chem. Commun.* **2009**, 5731-5733.
(4) Ren, L.; Wang, L.; Lv, Y.; Li, G.; Gao, S., Synergistic H_4NI–AcOH Catalyzed Oxidation of the Csp^3–H Bonds of Benzylpyridines with Molecular Oxygen. *Org. Lett.* **2015**, *17*, 2078-2081.
(5) Jones, M. R. Advances in Iridium-Catalyzed C-H Borylation Enabled by 2,2′-Dipyridylarylmethane Ligands. Ph.D. Dissertation, Vanderbilt University, United States, Tennessee, 2021.
(6) Ji, X.; Huang, T.; Wu, W.; Liang, F.; Cao, S., LDA-Mediated Synthesis of Triarylmethanes by Arylation of Diarylmethanes with Fluoroarenes at Room Temperature. *Org. Lett.* **2015**, *17*, 5096-5099.
(7) Park, S.; Kim, B. G.; Göttker-Schnetmann, I.; Brookhart, M., Redistribution of Trialkyl Silanes Catalyzed by Iridium Silyl Complexes. *ACS Catalysis* **2012**, *2*, 307-316.
(8) Zhao, P.; Huang, J.; Li, J.; Zhang, K.; Yang, W.; Zhao, W., Ligand-controlled cobalt-catalyzed remote hydroboration and alkene isomerization of allylic siloxanes. *Chem. Commun.* **2022**, *58*, 302-305.
(9) Dolomanov, O. V.; Bourhis, L. J.; Gildea, R. J.; Howard, J. A. K.; Puschmann, H., OLEX2: a complete structure solution, refinement and analysis program. *J. Appl. Crystallogr.* **2009**, *42*, 339-341.
(10) Sheldrick, G., A short history of SHELX. *Acta Crystallogr., Sect. A* **2008**, *64*, 112-122.
(11) *Gaussian 16, Revision B.01*, M. J. Frisch, G. W. T., H. B. Schlegel, G. E. Scuseria, M. A. Robb, J. R. Cheeseman, G. Scalmani, V. Barone, G. A. Petersson, H. Nakatsuji, X. Li, M. Caricato, A. V. Marenich, J. Bloino, B. G. Janesko, R. Gomperts, B. Mennucci, H. P. Hratchian, J. V. Ortiz, A. F. Izmaylov, J. L. Sonnenberg, D. Williams-Young, F. Ding, F. Lipparini, F. Egidi, J. Goings, B. Peng, A. Petrone, T. Henderson, D. Ranasinghe, V. G. Zakrzewski, J. Gao, N. Rega, G. Zheng, W. Liang, M. Hada, M. Ehara, K. Toyota, R. Fukuda, J. Hasegawa, M. Ishida, T. Nakajima, Y. Honda, O. Kitao, H. Nakai, T. Vreven, K. Throssell, J. A. Montgomery, Jr., J. E. Peralta, F. Ogliaro, M. J. Bearpark, J. J. Heyd, E. N. Brothers, K. N. Kudin, V. N. Staroverov, T. A. Keith, R. Kobayashi, J. Normand, K.

Raghavachari, A. P. Rendell, J. C. Burant, S. S. Iyengar, J. Tomasi, M. Cossi, J. M. Millam, M. Klene, C. Adamo, R. Cammi, J. W. Ochterski, R. L. Martin, K. Morokuma, O. Farkas, J. B. Foresman, and D. J. Fox, Gaussian, Inc., Wallingford CT, 2016.
(12) Zhao, Y.; Truhlar, D. G., A new local density functional for main-group thermochemistry, transition metal bonding, thermochemical kinetics, and noncovalent interactions. *J. Chem. Phys.* **2006**, *125*, 194101.
(13) Weigend, F.; Ahlrichs, R., Balanced basis sets of split valence, triple zeta valence and quadruple zeta valence quality for H to Rn: Design and assessment of accuracy. *PCCP* **2005**, *7*, 3297-3305.
(14) Schuchardt, K. L.; Didier, B. T.; Elsethagen, T.; Sun, L.; Gurumoorthi, V.; Chase, J.; Li, J.; Windus, T. L., Basis Set Exchange: A Community Database for Computational Sciences. *J. Chem. Inf. Model.* **2007**, *47*, 1045-1052.
(15) Goerigk, L.; Grimme, S., A thorough benchmark of density functional methods for general main group thermochemistry, kinetics, and noncovalent interactions. *PCCP* **2011**, *13*, 6670-6688.
(16) *xyzrender: Publication-quality molecular graphics from the command line*, Goodfellow, A. S., 2026.
